# Supplementary material for: Silver/chiral pyrrolidinopyridine relay catalytic cycloisomerization/(2 + 3) cycloadditions of enynamides to asymmetrically synthesize bispirocyclopentenes as PDE1B inhibitors
Source: Commun Chem. 2023 Jun 19;6:128. doi: 10.1038/s42004-023-00921-6 (PMC10279699; doi:10.1038/s42004-023-00921-6)
Supplement: Supplementary file 2 — Supplementary Methods [file 42004_2023_921_MOESM2_ESM.pdf]

Silver/chiral pyrrolidinopyridine relay catalytic cycloisomerization/(2 + 3) cycloadditions of enynamides to asymmetrically synthesize bispirocyclopentenes as PDE1B inhibitors

Jing Jiang[1], Jin Zhou[1], Yang Li[1], Cheng Peng[1], Gu He[2], Wei Huang[1], Gu Zhan[1]*, and Bo, Han[1]*

[1]*State Key Laboratory of Southwestern Chinese Medicine Resources, School of Pharmacy, Chengdu University of Traditional Chinese Medicine, Chengdu 611137, China.*

[2]*State Key Laboratory of Biotherapy and Department of Pharmacy, West China Hospital Sichuan University, Chengdu 610041, China*

E-mail: zhangu@cdutcm.edu.cn; hanbo@cdutcm.edu.cn

Supplementary Methods

Table of Contents

1. General information S2

2. General procedure for the preparation of new chiral PPY catalysts S2

3. Optimization of the reaction conditions S3

4. Bioassay of phosphodiesterase PDE1 and other PDE subfamilies S4

5. General procedure for the cycloisomerization/(2 + 3) cycloaddition reaction S5

6. Control experiments S6

7. Scale-up synthesis of 3a S7

8. Derivatization of product 3a S7

9. Reaction with other substrate S9

10. Supplementary References S9

1. General information

High Performance Liquid Chromatography (HPLC) was analyzed by chiral column in comparison with authentic racemates, using a Daicel Chiralpak IE Column (250 x 4.6 mm), Daicel Chiralpak IF Column (250 x 4.6 mm) or Daicel Chiralpak IC Column (250 x 4.6 mm). UV detection was performed at 254 nm. Nuclear magnetic resonance (NMR) spectra were recorded in $CDCl_3$ and on Bruker 600, 700 MHz, or JEOL 600 NMR instrument for ^{1}H, and at 151 or 176 MHz for ^{13}C. Proton chemical shifts were reported in parts per million (δ scale). The ^{1}H NMR chemical shifts were reported in ppm with the internal TMS signal at 0.0 ppm as standard. The ^{13}C NMR chemical shifts were given by using $CDCl_3$ as the internal standard ($CDCl_3$: δ = 77.23 ppm). The ^{19}F NMR chemical shifts were given by using $CDCl_3$. Data were reported as follows: chemical shift [multiplicity (s = singlet, d = doublet, t = triplet, q = quartet, m = multiplet, dq = doublet of quartets, dd = doublet of doublets, td = triplet of doublets, dt = doublet of tripiets, ddd = doublet of doublet of doublets), coupling constant(s) (Hz), integration]. High-resolution mass spectra (HRMS) were obtained using Agilent P/N G1969-90010. High-resolution mass spectra were reported for the molecular ion $[M+Na]^+$ or $[M+H]^+$. Melting points were recorded on BUCHI Melting Point M-565 instrument. X-ray diffraction experiment was carried out on an Agilent Gemini and the data obtained were deposited at the Cambridge Crystallographic Data Centre. UV detection was performed at 254 nm. Column chromatography was performed on silica gel (300-400 mesh) using an eluent of ethyl acetate (EA), petroleum ether (PE) and dichloromethane (DCM). TLC was performed on glass-backed silica plates; products were visualized using UV light. Optical rotation values were measured with instruments operating at λ = 589 nm, corresponding to the sodium D line at 35 °C. All reagents and solvents were obtained from commercial sources and used without further purification. Enynamides **1**[1,2] and MBH carbonate **2**[3] were prepared according to the literature procedures. Oil baths were used as the heat source.

2. General procedure for the preparation of new chiral PPY catalysts

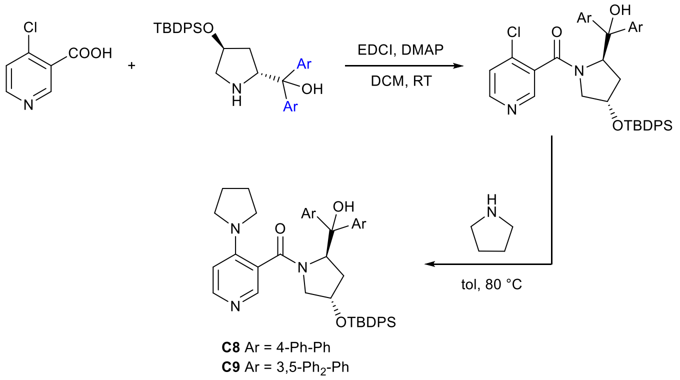

The new catalyst (**C8** or **C9**) was readily synthesized from corresponding chiral secondary amines and 4-chloronicotinic acid. To a solution of chiral secondary amines (0.45 mmol) in DCM (10 mL) was successively added 4-chloronicotinic acid (0.54 mmol), 1-ethyl-3-(3-dimethyllaminopropyl) carbodiimide hydrochloride (EDCI, 0.90 mmol) and 4-Dimethylaminopyridine (DMAP, 0.09 mmol). The mixture was stirred at room temperature for 5 h before being quenched with water (10 mL). The phases were separated and the aqueous phase was extracted with DCM (2 × 10 mL). The combined

organic phases were washed with brine (20 mL) before being dried (Na_2SO_4) and concentrated in vacuum. Purification by flash column chromatography on silica gel yielded the amide. To a solution of the amide (0.40 mmol) in toluene (15 mL) was added pyrrolidine (0.48 mmol). Then, the mixture was stirred at 80 °C overnight. After completion, the solvent was removed under reduced pressure and the residue was purified by flash column chromatography on silica gel (PE: EA = 1:1) to afford the catalyst. The corresponding chiral secondary amines were prepared according to the literature procedure[4].

3. Optimization of the reaction conditions

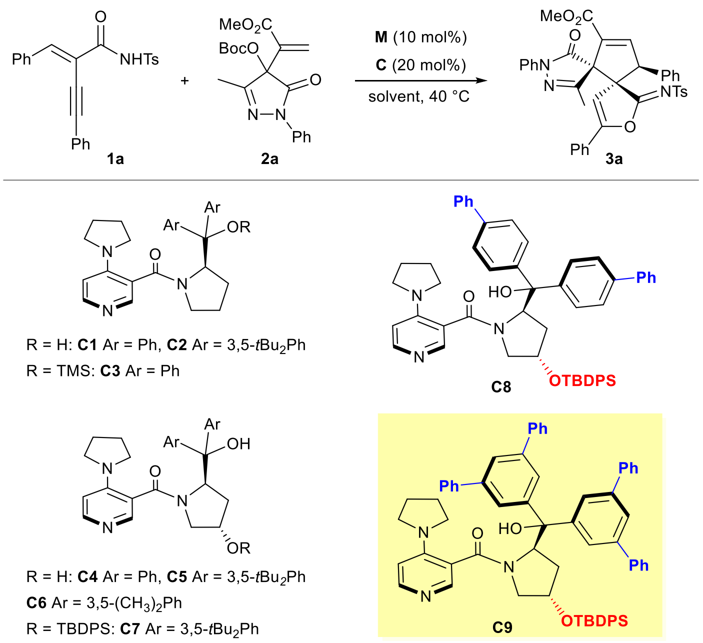

Table S1. Effect of metals/Lewis bases on the reaction[a]

Entry	**M**	**C**	Yield (%)	Er
1 [b]	PPh_3AuCl	PPh_3	N.R.	-
2 [b]	PPh_3AuCl	DABCO	N.R.	-
3 [b]	PPh_3AuCl	**C1**	49	76:24
4	AgOTf	**C1**	66	79:21
5	AgO	**C1**	66	76:24
6	AgF	**C1**	65	76:24
7	$AgSbF_6$	**C1**	37	77:23
8	$AgNTf_2$	**C1**	48	77:23
9	Ag_2CO_3	**C1**	56	72:28
10	AgOAc	**C1**	58	80:20
11	AgOAc	**C2**	26	74:26
12	AgOAc	**C3**	42	70:30
13	AgOAc	**C4**	37	56:43
14	AgOAc	**C5**	66	74:26

15	AgOAc	**C6**	22	83:17
16	AgOAc	**C7**	52	85:15
17	AgOAc	**C8**	86	86:14
18	AgOAc	**C9**	82	89:11

[a] Unless noted, reactions were performed with **1a** (0.10 mmol) and **M** (10 mol%) in $CHCl_3$ (1.0 mL) at 40 °C for 1 hour before **2a** (0.1 mmol) and **C** (20 mol%) was added. Then the mixture was stirred at 40 °C for 8 hours. [b] $AgSbF_6$ (10 mol%) as additive.

Table S2. Effect of solvents [a]

Entry	solvent 1	solvent 2	Yield(%)	Er
1	DCM	$CHCl_3$	81	88 : 12
2	THF	$CHCl_3$	52	89 : 11
3	Tol.	$CHCl_3$	69	88 : 12
4	1,4-dioxane	$CHCl_3$	61	88 : 12
5	$CHCl_3$	$CHCl_3$	82	89 : 11
6	$CHCl_3$	DCM	77	87 : 13
7	$CHCl_3$	DCE	78	85 : 15
8	$CHCl_3$	Tol	52	84 : 16
9	$CHCl_3$	THF	71	83 : 17
10	$CHCl_3$	DMF	58	73 : 27

[a] Unless noted, reactions were performed with **1a** (0.10 mmol) and AgOAc (10 mol%) in solvent 1 (1.0 mL) at 40 °C for 1 hour, and then solvent 1 was removed under reduced pressure. **2a** (0.10 mmol) and **C9** (20 mol%) in solvent 2 (1.0 mL) were added and stirred for 8 hours.

Table S3. Effect of temperature[a]

Entry	temperature (°C)	Yield(%)	Er
1	0	64	95 : 5
2	10	69	94 : 6
3	20	70	90 : 10
4	40	82	89 : 11
5	60	95	77 : 23
6[b]	40	80	95 : 5

[a] Unless noted, reactions were performed with **1a** (0.10 mmol) and AgOAc (10 mol%) in $CHCl_3$ (1.0 mL) for 1 hour before **2a** (0.1 mmol) and **C9** (20 mol%) was added. Then the mixture was stirred at the corresponding temperature. [b] After **2a** (0.1 mmol) and **C9** (20 mol%) were added, the reaction was stirred at 0 °C for 48 h and then at 40 °C for 3 h.

4. Bioassay of phosphodiesterase PDE1 and other PDE subfamilies

The PDE1B protein was purified according to the protocols described in previous report. PDE activity was measured by a scintillation proximity assay using a fixed amount of enzyme and substrate

concentrations. The phosphodiesterase (PDE) assays measure the conversion of H^3-cAMP for PDE 1A, 1B, 1C, 3A1, 4D2, 7A2, 8A2 and 10A1) or H^3-cGMP for PDE 2A, 5A1, 6C, 9A2 and 11A4, by the relevant PDE enzyme subtype. The scintillation proximity beads bind selectively to H^3-AMP or H^3-GMP, with the magnitude of radioactive counts being directly related to PDE enzymatic activity. In brief, 1 µL of test compound in dimethyl sulfoxide was added to each well. Enzyme solution was then added to each well in buffer (Trizma and $MgCl_2$) containing Brij 35 (0.01% (v/v)). For PDE1 subtype assays the buffer additionally included $CaCl_2$ (30 mM) and calmodulin (25 U ml^{-1}). Subsequently, 20 µL of H^3-cGMP (or 20 µL of H^3-cAMP) was added to each well to start the reaction and the plate was incubated for 30 minutes at 25 °C. Following an additional 8h incubation period the plates were read on a MicroBeta radioactive plate counter to determine radioactive counts per well.

Table S4. Inhibitory profiling of compound **3x** across representative PDE subtypes.

PDE subtype	IC_{50} (nM)	Selectivity (fold)
PDE1A	31.37	2.02
PDE1B	15.54	-
PDE1C	21.19	1.36
PDE2A	>10,000	>643
PDE3A	>10,000	>643
PDE4D2	1,237	79.6
PDE5A1	976	62.8
PDE6C	>10,000	>643
PDE7A1	>10,000	>643
PDE8A2	>10,000	>643
PDE9A2	>10,000	>643
PDE10A1	>10,000	>643
PDE11A4	>10,000	>643

5. General procedure for the cycloisomerization/(2 + 3) cycloaddition reaction

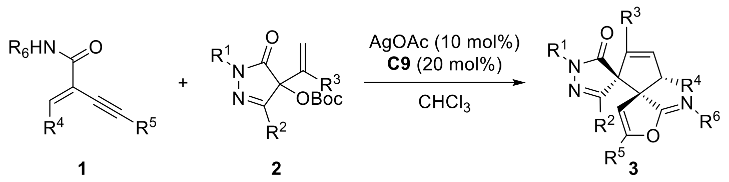

A mixture of enynamides **1** (0.10 mmol), AgOAc (1.7 mg, 0.01 mmol, 10 mol%) in $CHCl_3$ (0.5 mL) was stirred at 40 °C for 1 h, MBH carbonate **2** (0.10 mmol), **C9** (19.7 mg, 0.02 mmol, 20 mol%) in $CHCl_3$ (0.5 mL) were added to the above solution and stirred at 0 °C for 48 h, and at 40 °C for another 3 h until the reaction was complete (determined by TLC analysis). The mixture was concentrated under vacuum and purified by column chromatography on silica gel (PE: EA: DCM = 10:1:1 to 5:1:1) to afford the pure products **3**.

6. Control experiments

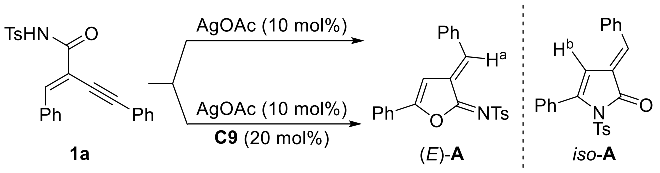

A mixture of **1a** (40.2 mg, 0.1 mmol), AgOAc (1.7, 0.01 mmol, 10 mol%) in $CHCl_3$ (1.0 mL) with or without catalyst **C9** (19.7 mg, 0.02 mmol, 20 mol%), and stirred at 40 °C for 1 h. Intermediate (*E*)-**A** was efficiently generated as the major product with a trace amount of *iso*-**A** in both reactions (unstable during isolation, monitored by NMR), showing that **C9** did not influence the cycloisomerization step. The ratio of intermediate (*E*)-**A** and *iso*-**A** was identified by ^{1}H NMR (about 25.4: 1).

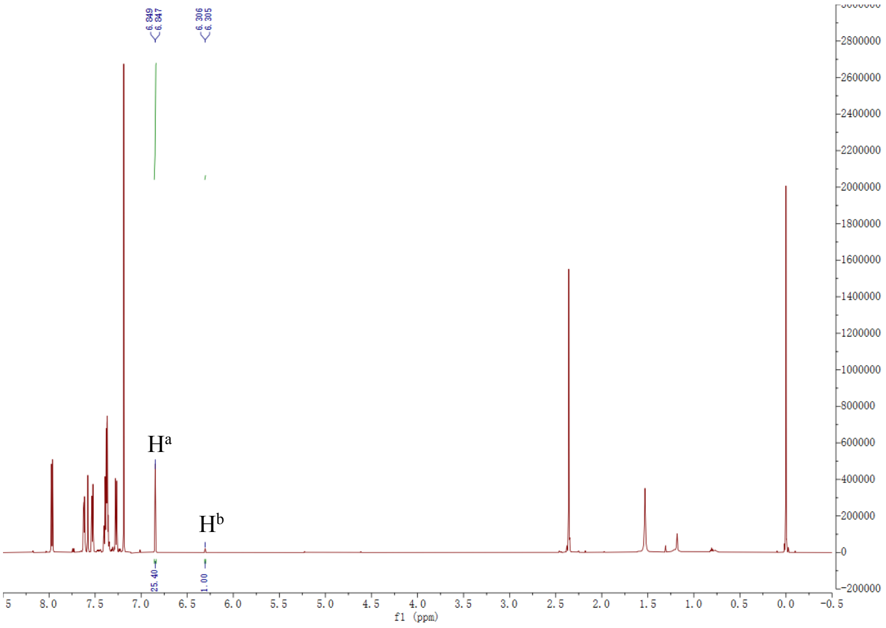

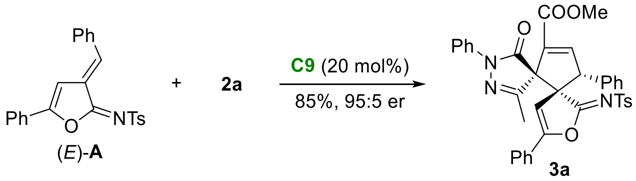

A mixture of **2a** (11.2 mg ,0.03 mmol), (*E*)-**A** (12.0 mg, 0.03 mmol), catalyst **C9** (5.9 mg, 0.006 mmol, 20 mol%) in $CHCl_3$ (0.5 mL) was stirred at 0 °C for 2 days. Then heated it to 40 °C for 3 hours until the reaction was complete (determined by TLC analysis). The mixture was concentrated under vacuum and purified by column chromatography on silica gel (PE: EA: DCM = 10:1:1) to afford the product **3a** (16.8 mg, 85% yield, 95:5 er) as a white solid.

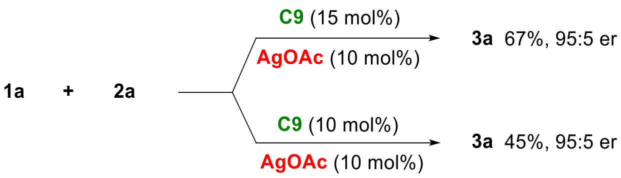

A mixture of **1a** (40.2 mg, 0.1 mmol), AgOAc (1.7 mg, 0.01 mmol, 10 mol%), in $CHCl_3$ (0.5 mL) was stirred at 40 °C for 1 hour, **2a** (37.4 mg, 0.1 mmol), catalyst **C9** (9.9 mg, 0.01 mmol, 10 mol% or 14.8

mg, 0.015 mmol, 15 mol%) in $CHCl_3$ (0.5 mL) were added to the above solution and stirred at 0 °C for 48 h, and then heated it to 40 °C for 3 h. The mixture was concentrated under vacuum and purified by column chromatography on silica gel (PE: EA: DCM = 10:1:1) to afford the product **3a** (29.8 mg, 45% yield, 95:5 er and 44.2 mg, 67% yield, 95:5 er, respectively) as a white solid.

7. Scale-up synthesis of 3a

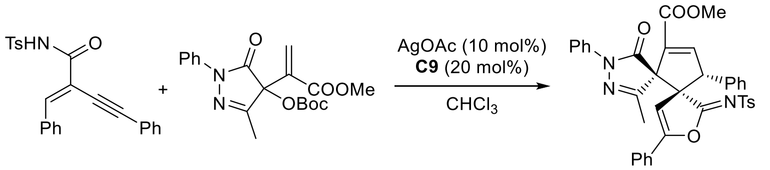

1a 1.0 mmol   **2a** 1.0 mmol   **3a** 71%, 464.9 mg, 95:5 er

A mixture of (E)-2-benzylidene-4-phenyl-N-tosylbut-3-ynamide **1a** (401.5 mg, 1.0 mmol), AgOAc (16.7 mg, 0.1 mmol, 10 mol%) in $CHCl_3$ (5 mL) was stirred at 40 °C for 1 hour, methyl 2-(4-((tert-butoxycarbonyl)oxy)-3-methyl-5-oxo-1-phenyl-4,5-dihydro-1H-pyrazol-4-yl)acrylate **2a** (374.4 mg, 1.0 mmol), catalyst **C9** (197.3 mg, 0.2 mmol, 20 mol%) in $CHCl_3$ (5 mL) were added to the above solution and stirred at 0 °C for 2 days, and then heated it to 40 °C for 3 hours until the reaction was complete (determined by TLC analysis). The mixture was concentrated under vacuum and purified by column chromatography on silica gel (PE: EA: DCM = 10:1:1) to afford the product **3a** (464.9 mg, 71% yield, 95:5 er) as a white solid.

8. Derivatization of product 3a

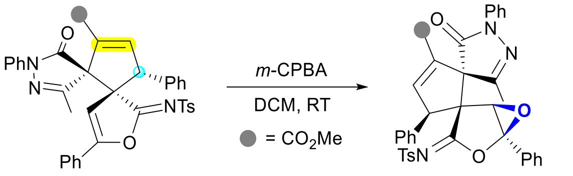

3a (95:5 er)   **4** 86%, >19:1 dr, 93:7 er

To a solution of **3a** (65.8 mg, 0.1 mmol) in DCM (2 mL) was added *m*-CPBA (34.5 mg, 0.2 mmol). The mixture was stirred at room temperature overnight. The reaction was then quenched with saturated $NaHCO_3$ solution. The organic phase was directly purified by silica gel column (PE: EA = 7:1) to give the desired product **4** as a white solid (57.6 mg, 86% yield, >19:1 dr, 93:7 er).

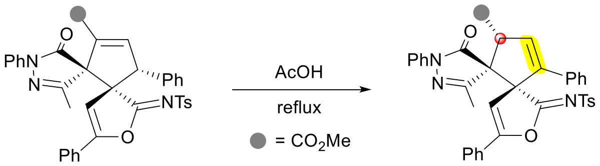

3a   **3a'** 78%, 94:6 er

A solution of **3a** (50.0 mg, 0.076 mmol) in AcOH (1 mL) was refluxed for 2 hours. The mixture was then quenched by water, and extracted with DCM. The organic phase was dried with anhydrous Na_2SO_4, and concentrated in vacuo. The crude mixture was purified with silica gel column (PE: EA = 8:1) to obtain the desired product **3a'** as a white solid (38.8 mg, 78% yield, 94:6 er).

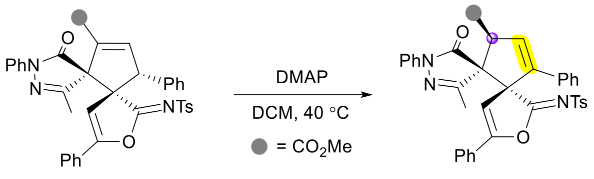

3a **5** 83%, 94: 6 er

To a solution of **3a** (30.0 mg, 0.046 mmol) in DCM (0.5 mL) was added DMAP (11.2 mg, 0.092 mmol). The reaction mixture was stirred at 40 ℃ overnight. The reaction solution was directly purified with silica gel column (PE: EA = 9:1) to obtain the desired product **5** as a white solid (24.9 mg, 83% yield, 94:6 er).

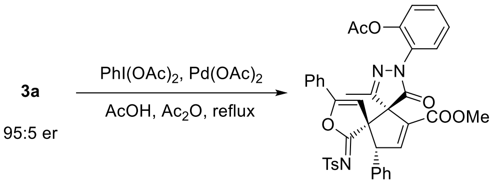

6 91%, 94:6 er

A solution containing **3a** (32.9 mg, 0.05 mmol), (diacetoxyiodo)benzene (19.3 mg, 0.06 mmol), and $Pd(OAc)_2$ (0.1 mg, 0.005 mmol) in AcOH (0.3 mL) and acetic anhydride (0.3 mL) was refluxed for 2 hours. The reaction mixture was then quenched by water, and extracted with DCM. The organic phase was dried with anhydrous Na_2SO_4, and concentrated in vacuo. The crude mixture was purified with silica gel column (PE: EA = 5:1) to obtain the desired product **6** as a white solid (29.9 mg, 91% yield, 94:6 er).

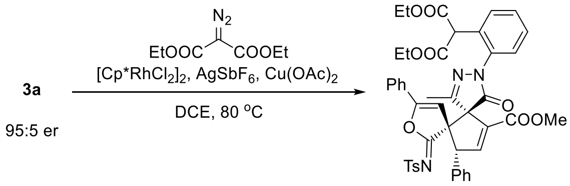

7 97%, 94:6 er

3a (32.9 mg, 0.05 mmol), diazo compound (14.0 mg, 0.075 mmol), $[Cp^*RhCl_2]_2$ (1.3 mg, 0.002 mmol), $AgSbF_6$ (3.4 mg, 0.01 mmol) and $Cu(OAc)_2$ (9.1 mg, 0.05 mmol) were dissolved in DCE (1 mL) under argon atmosphere. The mixture was stirred at 80 °C overnight. After that the solvent was removed under reduced pressure. The residue was purified by silica gel chromatography (PE: EA = 4:1) to afford compound **7** as a white solid (39.6 mg, 97% yield, 94:6 er). Diazo compound was prepared according to the literature procedures[5].

9. Reaction with other substrate

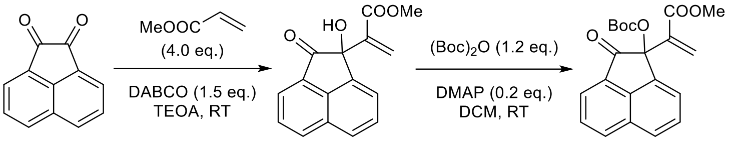

S1 **2b**

To a stirred solution of acenaphthylene-1,2-dione (182.2 mg, 1.0 mmol) and methyl acrylate (344.4 mg, 4.0 mmol) in triethanolamine (10 mL) was added DABCO (168.3 mg, 1.5 mmol), and the mixture was stirred at room temperature for 2 hours. After completion (monitored by TLC), the mixture was extracted with water and DCM. The organic phase was dried with anhydrous Na_2SO_4, and concentrated in vacuo. The residue was purified by flash column chromatography (PE: EA = 5:1) to give the product **S1**. $(Boc)_2O$ (0.28 mL, 1.2 mmol) was added to a stirred solution of **S1** (268.3 mg, 1.0 mmol) and DMAP (24.4 mg, 0.2 mmol) in DCM (5.0 mL) at room temperature for 2 hours. After completion (monitored by TLC), the mixture was concentrated under reduced pressure. The residue was purified by flash chromatography on silica gel (PE: EA = 15:1) to give the product **2b** as a red solid (332.6 mg, 90% yield).

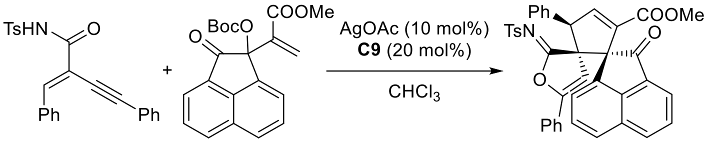

1a **2b** **3ba**
45%, 86:14 er

A mixture of (E)-2-benzylidene-4-phenyl-N-tosylbut-3-ynamide **1a** (40.2 mg, 0.1 mmol), AgOAc (1.7 mg, 0.01 mmol, 10 mol%) in $CHCl_3$ (0.5 mL) was stirred at 40 °C for 1 hour, methyl 2-(1-((tert-butoxycarbonyl)oxy)-2-oxo-1,2-dihydroacenaphthylen-1-yl)acrylate **2b** (36.8 mg, 0.1 mmol), catalyst **C9** (19.7 mg, 0.02 mmol, 20 mol%) in $CHCl_3$ (0.5 mL) were added to the above solution and stirred at 0 °C for 2 days, and then heated it to 40 °C for 3 hours until the reaction was complete (determined by TLC analysis). The mixture was concentrated under vacuum and purified by column chromatography on silica gel (PE: EA: DCM = 10:1:1) to afford the product **3ba** (29.1 mg, 45% yield, 86:14 er) as a pink solid.

10. Supplementary References

1. Rossi, R., Bellina, F., Bechini, C., Mannina, L. & Vergamini, P. Studies on the transition metal-catalyzed synthesis of variously substituted (E)-3-[1-(aryl)methylidene]- and (E)-3-(1-alkylidene)-3H-furan-2-ones. *Tetrahedron* **54**, 135-156 (1998).
2. Luo, Y. *et al.* Synthesis, biological evaluation, and molecular modeling of cinnamic acyl sulfonamide derivatives as novel antitubulin agents. *Bioorg. Med. Chem.* **19**, 4730-4738 (2011).
3. Yang, Z.-H. *et al.* A Double Deprotonation Strategy for Cascade Annulations of Palladium–Trimethylenemethanes and Morita–Baylis–Hillman Carbonates to Construct Bicyclo[3.1.0]hexane Frameworks. *Angew. Chem. Int. Ed.* **60**, 13913-13917 (2021).
4. Caruana, L., Kniep, F., Johansen, T. K., Poulsen, P. H. & Jørgensen, K. A. A New

Organocatalytic Concept for Asymmetric α-Alkylation of Aldehydes. *J. Am. Chem. Soc.* **136**, 15929-15932 (2014).

5. Friscourt, F., Fahrni, C. J. & Boons, G.-J. Fluorogenic Strain-Promoted Alkyne–Diazo Cycloadditions. *Chem. -Eur. J.* **21**, 13996-14001 (2015).
