# Supplementary material for: Silver/chiral pyrrolidinopyridine relay catalytic cycloisomerization/(2 + 3) cycloadditions of enynamides to asymmetrically synthesize bispirocyclopentenes as PDE1B inhibitors
Source: Commun Chem. 2023 Jun 19;6:128. doi: 10.1038/s42004-023-00921-6 (PMC10279699; doi:10.1038/s42004-023-00921-6)
Supplement: Supplementary file 3 — Description of Additional Supplementary Files [file 42004_2023_921_MOESM3_ESM.pdf]

Description of Additional Supplementary Files

File name: Supplementary Data 1

Description: Full characterization data of compounds

File name: Supplementary Data 2

Description: 1H, 13C NMR, 19F NMF spectra, and HPLC chromatograms

File name: Supplementary Data 3

Description: The CIF file of X-ray crystallographic coordinates for structure 3a

File name: Supplementary Data 4

Description: Computational chemistry details

File name: Supplementary Data 5

Description: The experimental procedures for the bioassay and uncropped images from western blots
