# Supplementary material for: Silver/chiral pyrrolidinopyridine relay catalytic cycloisomerization/(2 + 3) cycloadditions of enynamides to asymmetrically synthesize bispirocyclopentenes as PDE1B inhibitors
Source: Commun Chem. 2023 Jun 19;6:128. doi: 10.1038/s42004-023-00921-6 (PMC10279699; doi:10.1038/s42004-023-00921-6)
Supplement: Supplementary file 4 — Supplementary Data 1 [file 42004_2023_921_MOESM4_ESM.pdf]

Supplementary Data 1

Characterization of catalysts and products

((2*S*,4*R*)-4-((tert-butyldiphenylsilyl)oxy)-2-(di([1,1'-biphenyl]-4-yl)(hydroxy)methyl)pyrrolidin-1-yl)(4-(pyrrolidin-1-yl)pyridin-3-yl)methanone (C8)

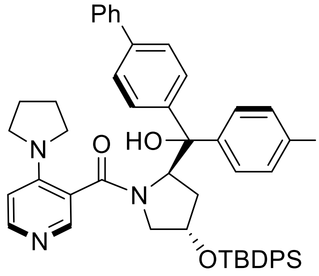

304.0 mg, 91% yield, white solid, m.p.: 172.2 – 176.8 ℃; $[\alpha]_D^{35}$ = - 82.899 (c = 0.035, DCM). **^{1}H NMR** (600 MHz, $CDCl_3$) δ 8.10 (d, *J* = 6.0 Hz, 1H), 7.63 – 7.60 (m, 3H), 7.61 – 7.56 (m, 4H), 7.54 – 7.50 (m, 4H), 7.48 – 7.39 (m, 9H), 7.38 (t, *J* = 7.8 Hz, 2H), 7.37 – 7.29 (m, 6H), 7.27 (t, *J* = 7.2 Hz, 2H), 6.45 (d, *J* = 6.0 Hz, 1H), 5.48 (t, *J* = 9.0 Hz, 1H), 4.14 (d, *J* = 3.0 Hz, 1H), 3.54 (d, *J* = 12.0 Hz, 1H), 3.50 (d, *J* = 7.8 Hz, 2H), 3.27 – 3.21 (m, 2H), 3.07 (dd, *J* = 12.0, 3.6 Hz, 1H), 2.06 (dd, *J* = 9.0, 3.0 Hz, 2H), 1.91 – 1.87 (m, 2H), 1.71 – 1.68 (m, 2H), 1.01 (s, 9H). **^{13}C NMR** (151 MHz, $CDCl_3$) δ 172.1, 150.0, 149.0, 149.0, 144.0, 141.5, 140.8, 140.6, 140.6, 140.3, 135.6, 135.5, 133.2, 133.0, 130.2, 130.2, 128.9, 128.2, 128.1, 128.0, 128.0, 127.5, 127.5, 127.3, 127.2, 126.9, 126.6, 116.1, 108.7, 81.6, 71.3, 68.0, 60.8, 49.3, 40.3, 27.1, 25.8, 19.2. **HRMS** (ESI-TOF) m/z: $[M+H]^+$ Calcd for $C_{55}H_{56}N_3O_3Si^+$ 834.4085, Found 834.4089.

((2*S*,4*R*)-4-((tert-butyldiphenylsilyl)oxy)-2-(di([1,1':3',1''-terphenyl]-5'-yl)(hydroxy)methyl)pyrrolidin-1-yl)(4-(pyrrolidin-1-yl)pyridin-3-yl)methanone (C9)

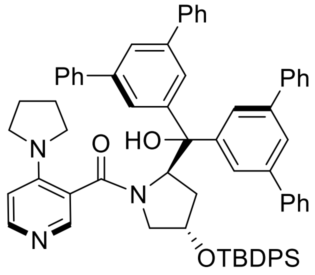

376.0 mg, 95% yield, white solid, m.p.: 172.0 ℃; $[\alpha]_D^{35}$ = + 282.500 (c = 0.015, DCM). **^{1}H NMR** (600 MHz, $CDCl_3$) δ 8.06 (d, *J* = 6.0 Hz, 1H), 7.99 (s, 1H), 7.84 (d, *J* = 1.8 Hz, 1H), 7.82 (d, *J* = 1.8 Hz, 2H), 7.73 (s, 3H), 7.61 – 7.57 (m, 8H), 7.47 (d, *J* = 7.2 Hz, 2H), 7.45 – 7.41 (m, 6H), 7.40 – 7.37 (m, 6H), 7.36 – 7.34 (m, 2H), 7.33 – 7.29 (m, 2H), 7.27 – 7.23 (m, 2H), 7.10 (t, *J* = 7.2 Hz, 2H), 6.98 (s, 1H), 6.43 (d, *J* = 6.6 Hz, 1H), 5.56 (t, *J* = 9.0 Hz, 1H), 4.16 (d, *J* = 3.0 Hz, 1H), 3.58 (d, *J* = 12.0 Hz, 1H), 3.51 (d, *J* = 8.4 Hz, 2H), 3.24 (d, *J* = 7.8 Hz, 2H), 3.18 (dd, *J* = 12.0, 3.6 Hz, 1H), 2.10 (dd, *J* = 9.0, 3.0 Hz, 2H), 1.93 (s, 2H), 1.73 (d, *J* = 7.8 Hz, 2H), 0.98 (s, 9H). **^{13}C NMR** (151 MHz, $CDCl_3$) δ 172.1, 149.9, 149.1, 148.7, 145.6, 143.3, 141.8, 141.3, 141.2, 135.5, 135.5, 132.9, 132.8, 130.3, 130.2, 129.0, 128.0, 128.0, 127.6, 127.6, 127.5, 126.1, 126.0, 125.7, 125.1, 116.0, 108.7, 82.1, 71.1, 69.5, 61.3, 49.5, 41.0, 27.1, 25.9, 19.2. **HRMS** (ESI-TOF) m/z: $[M+H]^+$ Calcd for $C_{67}H_{64}N_3O_3Si^+$ 986.4711, Found 986.4717.

N-((*Z*)-3-((*E*)-benzylidene)-5-phenylfuran-2(3*H*)-ylidene)-4-methylbenzenesulfonamide ((*E*)-A)

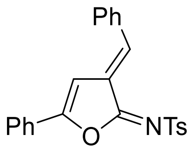

180.1 mg, 96% yield, yellow solid, m.p.:217.2 – 217.7 ℃. **^{1}H NMR** (600 MHz, $CDCl_3$) δ 7.97 (d, *J* = 8.4 Hz, 2H), 7.64 – 7.60 (m, 2H), 7.58 (s, 1H), 7.55 – 7.51 (m, 2H), 7.42 – 7.34 (m, 6H), 7.27 (d, *J* = 8.4 Hz, 2H), 6.85 (d, *J* = 1.2 Hz, 1H), 2.36 (s, 3H). **^{13}C NMR** (151 MHz, $CDCl_3$) δ 164.8, 158.7, 143.9, 138.6, 136.8, 135.1, 131.1, 131.0, 130.6, 129.6, 129.4, 129.2, 129.1, 127.9, 127.3, 125.7, 100.3, 21.8. **HRMS** (ESI-TOF) m/z: $[M+Na]^+$ Calcd for $C_{24}H_{19}NNaO_3S^+$ 424.0978, Found 424.0963.

(*E*)-3-benzylidene-5-phenyl-1-tosyl-1,3-dihydro-2*H*-pyrrol-2-one (*iso*-A)

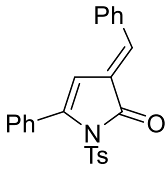

7.1 mg, 4% yield, yellow solid, m.p.: 177.9 ℃. **^{1}H NMR** (600 MHz, $CDCl_3$) δ 7.74 (d, *J* = 7.8 Hz, 2H), 7.50 – 7.45 (m, 2H), 7.47 – 7.43 (m, 2H), 7.40 – 7.35 (m, 3H), 7.33 – 7.29 (m, 4H), 7.23 (d, *J* = 7.8 Hz, 2H), 6.31 (d, *J* = 0.6 Hz, 1H), 2.35 (s, 3H).**^{13}C NMR** (151 MHz, $CDCl_3$) δ 168.8, 146.8, 145.4, 135.7, 135.4, 135.0, 132.3, 130.6, 130.6, 129.8, 129.8, 129.2, 128.5, 128.4, 128.1, 127.1, 108.6, 21.9. **HRMS** (ESI-TOF) m/z: $[M+Na]^+$ Calcd for $C_{24}H_{19}NNaO_3S^+$ 424.0978, Found 424.0962.

Methyl (5*R*,6*R*,11*R*,*E*)-1-methyl-4-oxo-3,9,11-triphenyl-7-(tosylimino)-8-oxa-2,3-diazadispiro[4.0.4^6.3^5]trideca-1,9,12-triene-13-carboxylate (3a)

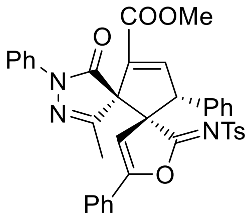

52.8 mg, 80% yield, white solid, m.p.: 228.4 ℃; **HPLC** (Daicel Chiralpak IE, n-hexane/2-propanol = 70:30, 1.0 mL/min, at 254 nm): t_R = 32.79 min (major), t_R = 47.74 min (minor); er = 95:5, $[\alpha]_D^{35}$ = + 954.737 (c = 0.015, DCM). **^{1}H NMR** (600 MHz, $CDCl_3$) δ 7.81 (d, *J* = 7.8 Hz, 2H), 7.51 (d, *J* = 2.4 Hz, 1H), 7.39 (d, *J* = 8.4 Hz, 2H), 7.33 (t, *J* = 7.8 Hz, 2H), 7.29 (t, *J* = 7.2 Hz, 1H), 7.21 (t, *J* = 7.8 Hz, 3H), 7.17 – 7.11 (m, 5H), 7.08 (d, *J* = 7.2 Hz, 2H), 7.04 (d, *J* = 7.2 Hz, 2H), 5.85 (s, 1H), 5.27 (d, *J* = 1.8 Hz, 1H), 3.70 (s, 3H), 2.33 (s, 3H), 2.18 (s, 3H). **^{13}C NMR** (151 MHz, $CDCl_3$) δ 171.0, 165.8, 162.7, 158.2, 156.2, 150.4, 144.0, 137.9, 137.6, 134.3, 133.8, 131.0, 129.6, 129.3, 129.1, 128.9, 128.9, 128.1, 127.7, 126.1, 125.8, 125.7, 119.4, 100.0, 71.8, 71.3, 56.0, 52.5, 21.8, 18.3. **HRMS** (ESI-TOF) m/z: $[M+Na]^+$ Calcd for $C_{38}H_{31}N_3NaO_6S^+$ 680.1826, Found 680.1825.

Methyl (5*R*,6*R*,11*R*,*E*)-3-(tert-butyl)-1-methyl-4-oxo-9,11-diphenyl-7-(tosylimino)-8-oxa-2,3-diazadispiro[4.0.4^6.3^5]trideca-1,9,12-triene-13-carboxylate (3b)

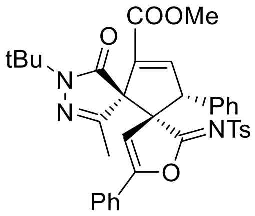

43.7 mg, 69% yield, white solid, m.p.: 229.7 ℃; **HPLC** (Daicel Chiralpak IE, n-hexane/2-propanol = 70:30, 1.0 mL/min, at 254 nm): t_R = 17.78 min (minor), t_R = 20.63 min (major); er = 97:3, $[\alpha]_D^{35}$ = + 87.949 (c = 0.015, DCM). **^{1}H NMR** (600 MHz, $CDCl_3$) δ 7.42 (d, *J* = 2.4 Hz, 1H), 7.36 (d, *J* = 8.4 Hz, 2H), 7.32 (tt, *J* = 7.2, 1.8 Hz, 1H), 7.26 (t, *J* = 7.2 Hz, 2H), 7.20 – 7.15 (m, 1H), 7.16 – 7.09 (m, 6H), 7.03 (d, *J* = 7.2 Hz, 2H), 5.90 (s, 1H), 5.18 (d, *J* = 2.4 Hz, 1H), 3.69 (s, 3H), 2.33 (s, 3H), 2.02 (s, 3H), 1.44 (s, 9H). **^{13}C NMR** (151 MHz, $CDCl_3$) δ 172.7, 166.1, 162.8, 155.5, 155.4, 149.9, 143.9, 138.0, 134.6, 134.2, 130.8, 129.5, 129.2, 129.0, 128.8, 128.0, 127.6, 126.4, 125.4, 100.8, 71.7, 70.7, 58.1, 55.8, 52.3, 28.3, 21.8, 18.1. **HRMS** (ESI-TOF) m/z: $[M+Na]^+$ Calcd for $C_{36}H_{35}N_3NaO_6S^+$ 660.2139, Found 660.2140.

Methyl (5*R*,6*R*,11*R*,*E*)-1,3-dimethyl-4-oxo-9,11-diphenyl-7-(tosylimino)-8-oxa-2,3-diazadispiro[4.0.4^6.3^5]trideca-1,9,12-triene-13-carboxylate (3c)

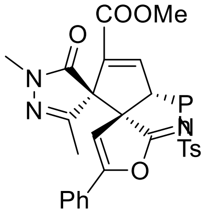

43.3 mg, 73% yield, white solid, m.p.: 187.4 ℃; **HPLC** (Daicel Chiralpak IE, n-hexane/2-propanol = 70:30, 1.0 mL/min, at 254 nm): t_R = 31.37 min (minor), t_R = 37.65 min (major); er = 91:9, $[\alpha]_D^{35}$ = + 69.126 (c = 0.015, DCM). **^{1}H NMR** (600 MHz, $CDCl_3$) δ 7.45 (d, *J* = 2.4 Hz, 1H), 7.38 (d, *J* = 8.4 Hz, 2H), 7.32 (tt, *J* = 7.2, 1.8 Hz, 1H), 7.27 (t, *J* = 7.8 Hz, 2H), 7.17 (d, *J* = 7.2 Hz, 1H), 7.17 – 7.12 (m, 4H), 7.14 – 7.10 (m, 2H), 7.02 (d, *J* = 7.2 Hz, 2H), 5.84 (s, 1H), 5.22 (d, *J* = 1.8 Hz, 1H), 3.71 (s, 3H), 3.25 (s, 3H), 2.33 (s, 3H), 2.06 (s, 3H). **^{13}C NMR** (151 MHz, $CDCl_3$) δ 172.6, 166.0, 162.7, 157.6, 155.9, 150.2, 144.0, 137.9, 134.4, 133.9, 131.0, 129.5, 129.2, 128.9, 128.8, 128.1, 127.7, 126.3, 125.6, 100.4, 70.8, 70.1, 56.0, 52.4, 31.7, 21.8, 18.2. **HRMS** (ESI-TOF) m/z: $[M+Na]^+$ Calcd for $C_{33}H_{29}N_3NaO_6S^+$ 618.1669, Found 618.1668.

Methyl (5*R*,6*R*,11*R*,*E*)-1-methyl-4-oxo-9,11-diphenyl-3-(p-tolyl)-7-(tosylimino)-8-oxa-2,3-diazadispiro[4.0.4^6.3^5]trideca-1,9,12-triene-13-carboxylate (3d)

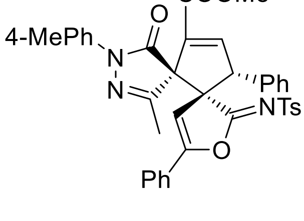

63.4 mg, 94% yield, white solid, m.p.: 237.8 – 240.5 ℃; **HPLC** (Daicel Chiralpak IE, n-hexane/2-propanol = 70:30, 1.0 mL/min, at 254 nm): t_R = 32.84 min (major), t_R = 47.06 min (minor); er = 93:7,

$[\alpha]_D^{35}$ = + 764.545 (c = 0.015, DCM). **^{1}H NMR** (600 MHz, $CDCl_3$) δ 7.67 (dt, *J* = 8.4, 2.4 Hz, 2H), 7.50 (d, *J* = 2.4 Hz, 1H), 7.40 (d, *J* = 7.8 Hz, 2H), 7.29 (t, *J* = 7.2 Hz, 1H), 7.24 – 7.18 (m, 3H), 7.17 – 7.11 (m, 6H), 7.08 (dd, *J* = 8.4, 1.2 Hz, 2H), 7.04 (dd, *J* = 7.2, 1.8 Hz, 2H), 5.85 (s, 1H), 5.27 (d, *J* = 1.8 Hz, 1H), 3.70 (s, 3H), 2.34 (s, 3H), 2.29 (s, 3H), 2.17 (s, 3H). **^{13}C NMR** (151 MHz, $CDCl_3$) δ 170.8, 165.9, 162.7, 158.1, 156.1, 150.4, 144.0, 138.0, 135.6, 135.2, 134.4, 133.9, 131.0, 129.7, 129.6, 129.3, 128.9, 128.9, 128.1, 127.7, 126.2, 125.7, 119.5, 100.1, 71.7, 71.4, 56.0, 52.5, 21.8, 21.2, 18.3. **HRMS** (ESI-TOF) m/z: $[M+Na]^+$ Calcd for $C_{39}H_{33}N_3NaO_6S^+$ 694.1982, Found 694.1985.

Methyl (5*R*,6*R*,11*R*,*E*)-1-methyl-4-oxo-9,11-diphenyl-3-(m-tolyl)-7-(tosylimino)-8-oxa-2,3-diazadispiro[4.0.4^6.3^5]trideca-1,9,12-triene-13-carboxylate (3e)

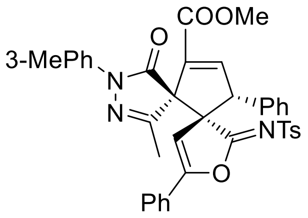

36.9 mg, 55% yield, white solid, m.p.: 323.4 – 323.5 ℃; **HPLC** (Daicel Chiralpak IC, n-hexane/2-propanol = 70:30, 1.0 mL/min, at 254 nm): t_R = 34.24 min (minor), t_R = 40.02 min (major); er = 99.5:0.5, $[\alpha]_D^{35}$ = + 83.333 (c = 0.015, DCM). **^{1}H NMR** (600 MHz, $CDCl_3$) δ 7.63 (d, *J* = 1.8 Hz, 1H), 7.61 (dd, *J* = 7.8, 1.8 Hz, 1H), 7.51 (d, *J* = 1.8 Hz, 1H), 7.40 (d, *J* = 8.4 Hz, 2H), 7.29 (t, *J* = 7.2 Hz, 1H), 7.21 (ddd, *J* = 13.8, 7.2, 3.0 Hz, 4H), 7.14 (td, *J* = 7.2, 6.0 Hz, 4H), 7.09 (d, *J* = 7.2 Hz, 2H), 7.04 (d, *J* = 7.2 Hz, 2H), 6.97 (d, *J* = 7.2 Hz, 1H), 5.85 (s, 1H), 5.28 (d, *J* = 2.4 Hz, 1H), 3.70 (s, 3H), 2.33 (s, 3H), 2.30 (s, 3H), 2.18 (s, 3H). **^{13}C NMR** (151 MHz, $CDCl_3$) δ 171.0, 165.8, 162.6, 158.1, 156.2, 150.4, 144.0, 139.1, 137.9, 137.5, 134.4, 133.9, 131.0, 129.6, 129.3, 129.0, 128.9, 128.9, 128.1, 127.7, 126.7, 126.1, 125.7, 120.0, 116.6, 100.0, 71.8, 71.4, 56.0, 52.5, 21.8, 21.8, 18.3. **HRMS** (ESI-TOF) m/z: $[M+Na]^+$ Calcd for $C_{39}H_{33}N_3NaO_6S^+$ 694.1982, Found 694.1984.

Methyl (5*R*,6*R*,11*R*,*E*)-3-(3-methoxyphenyl)-1-methyl-4-oxo-9,11-diphenyl-7-(tosylimino)-8-oxa-2,3-diazadispiro[4.0.4^6.3^5]trideca-1,9,12-triene-13-carboxylate (3f)

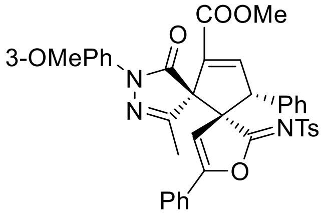

68.8 mg, 99% yield, white solid, m.p.: 221.2 ℃; **HPLC** (Daicel Chiralpak IE, n-hexane/2-propanol = 70:30, 1.0 mL/min, at 254 nm): t_R = 46.48 min (major), t_R = 68.34 min (minor); er = 90:10, $[\alpha]_D^{35}$ = + 837.778 (c = 0.015, DCM). **^{1}H NMR** (600 MHz, $CDCl_3$) δ 7.51 (d, *J* = 1.8 Hz, 1H), 7.45 – 7.42 (m, 2H), 7.39 (d, *J* = 8.4 Hz, 2H), 7.29 (t, *J* = 7.2 Hz, 1H), 7.26 – 7.18 (m, 4H), 7.14 (td, *J* = 7.8, 6.6 Hz, 4H), 7.08 (d, *J* = 7.2 Hz, 2H), 7.04 (d, *J* = 7.2 Hz, 2H), 6.71 – 6.69 (m, 1H), 5.84 (s, 1H), 5.27 (d, *J* = 2.4 Hz, 1H), 3.75 (s, 3H), 3.70 (s, 3H), 2.33 (s, 3H), 2.18 (s, 3H). **^{13}C NMR** (151 MHz, $CDCl_3$) δ 171.0, 165.8, 162.6, 160.2, 158.1, 156.2, 150.4, 144.1, 138.8, 137.9, 134.3, 133.8, 131.0, 129.9, 129.6, 129.3, 128.9, 128.9, 128.1, 127.7, 126.1, 125.7, 112.1, 111.5, 104.6, 99.9, 71.9, 71.4, 56.0, 55.6, 52.5, 21.8, 18.3. **HRMS** (ESI-TOF) m/z: $[M+Na]^+$ Calcd for $C_{39}H_{33}N_3NaO_7S^+$ 710.1931, Found 710.1928.

Methyl (5*R*,6*R*,11*R*,*E*)-1-methyl-4-oxo-9,11-diphenyl-3-(o-tolyl)-7-(tosylimino)-8-oxa-2,3-diazadispiro[4.0.4⁶.3⁵]trideca-1,9,12-triene-13-carboxylate (3g)

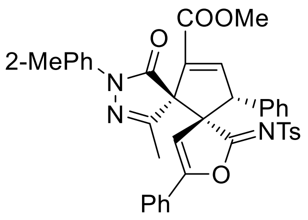

29.3mg, 44% yield, white solid, m.p.: 309.6 – 309.7 ℃; **HPLC** (Daicel Chiralpak IE, n-hexane/2-propanol = 70:30, 1.0 mL/min, at 254 nm): t_R = 27.44 min (minor), t_R = 33.16 min (major); er = 94:6, $[\alpha]_D^{35}$ = + 607.792 (c = 0.015, DCM). **^{1}H NMR** (600 MHz, $CDCl_3$) δ 7.50 (d, *J* = 1.8 Hz, 1H), 7.39 (d, *J* = 8.4 Hz, 2H), 7.37 – 7.31 (m, 1H), 7.28 (t, *J* = 7.8 Hz, 2H), 7.22 (d, *J* = 3.6 Hz, 2H), 7.20 – 7.18 (m, 2H), 7.18 – 7.16 (m, 3H), 7.16 – 7.12 (m, 4H), 7.04 (d, *J* = 7.2 Hz, 2H), 6.00 (s, 1H), 5.24 (d, *J* = 1.8 Hz, 1H), 3.75 (s, 3H), 2.34 (s, 3H), 2.19 (s, 3H), 2.15 (s, 3H). **^{13}C NMR** (151 MHz, $CDCl_3$) δ 171.9, 165.9, 162.7, 158.0, 155.9, 150.5, 144.0, 137.9, 135.5, 135.5, 134.3, 133.9, 131.4, 131.1, 129.6, 129.3, 129.1, 129.0, 128.9, 128.1, 127.7, 126.8, 126.8, 126.2, 125.5, 101.7, 70.8, 70.6, 56.0, 52.5, 21.8, 18.4, 18.2. **HRMS** (ESI-TOF) m/z: $[M+Na]^+$ Calcd for $C_{39}H_{33}N_3NaO_6S^+$ 694.1982, Found 694.1985.

Methyl (5*R*,6*R*,11*R*,*E*)-3-(2-bromophenyl)-1-methyl-4-oxo-9,11-diphenyl-7-(tosylimino)-8-oxa-2,3-diazadispiro[4.0.4⁶.3⁵]trideca-1,9,12-triene-13-carboxylate (3h)

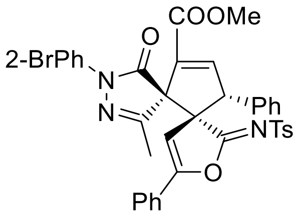

47.4 mg, 64% yield, white solid, m.p.: 303.1 – 303.2 ℃; **HPLC** (Daicel Chiralpak IE, n-hexane/2-propanol = 70:30, 1.0 mL/min, at 254 nm): t_R = 29.51 min (minor), t_R = 35.27 min (major); er = 94:6, $[\alpha]_D^{35}$ = + 175.850 (c = 0.015, DCM). **^{1}H NMR** (600 MHz, $CDCl_3$) δ 7.61 (dd, *J* = 8.4, 1.2 Hz, 1H), 7.50 (d, *J* = 2.4 Hz, 1H), 7.39 (d, *J* = 7.8 Hz, 2H), 7.33 (dd, J = 7.8, 6.6 Hz, 2H), 7.30 (dd, *J* = 7.8, 1.8 Hz, 1H), 7.27 (t, *J* = 7.8 Hz, 2H), 7.24 – 7.19 (m, 3H), 7.18 – 7.12 (m, 5H), 7.06 (d, *J* = 7.2 Hz, 2H), 6.14 (s, 1H), 5.22 (d, *J* = 1.8 Hz, 1H), 3.76 (s, 3H), 2.34 (s, 3H), 2.16 (s, 3H). **^{13}C NMR** (151 MHz, $CDCl_3$) δ 172.0, 165.9, 162.7, 158.3, 155.7, 150.4, 144.0, 137.9, 136.0, 134.3, 133.8, 133.7, 130.9, 130.5, 129.5, 129.2, 129.2, 128.9, 128.8, 128.5, 128.1, 127.6, 126.2, 125.6, 121.3, 101.0, 70.8, 70.3, 56.1, 52.5, 21.7, 18.3. **HRMS** (ESI-TOF) m/z: $[M+Na]^+$ Calcd for $C_{38}H_{30}BrN_3NaO_6S^+$ 758.0931, Found 758.0931.

Methyl (5*R*,6*R*,11*R*,*E*)-3-(2-chlorophenyl)-1-methyl-4-oxo-9,11-diphenyl-7-(tosylimino)-8-oxa-2,3-diazadispiro[4.0.4⁶.3⁵]trideca-1,9,12-triene-13-carboxylate (3i)

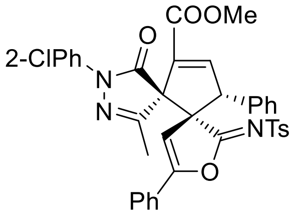

55.0 mg, 79% yield, white solid, m.p.: 231.2 – 238.2 ℃; **HPLC** (Daicel Chiralpak IE, n-hexane/2-propanol = 70:30, 1.0 mL/min, at 254 nm): t_R = 27.22 min (minor), t_R = 32.67 min (major); er = 93.5:6.5, $[\alpha]_D^{35}$ = + 196.667 (c = 0.015, DCM). **^{1}H NMR** (600 MHz, $CDCl_3$) δ 7.50 (d, *J* = 1.8 Hz, 1H), 7.45 – 7.41 (m, 1H), 7.39 (d, *J* = 8.4 Hz, 2H), 7.35 – 7.30 (m, 2H), 7.31 – 7.24 (m, 4H), 7.23 – 7.19 (m, 1H), 7.19 – 7.13 (m, 4H), 7.13 (d, *J* = 8.4 Hz, 2H), 7.06 (d, *J* = 6.6 Hz, 2H), 6.11 (s, 1H), 5.22 (d, *J* = 1.8 Hz, 1H), 3.75 (s, 3H), 2.34 (s, 3H), 2.16 (s, 3H). **^{13}C NMR** (151 MHz, CDCl3) δ 171.9, 165.9, 162.8, 158.4, 155.8, 150.6, 144.0, 138.0, 134.4, 134.2, 133.9, 131.6, 131.0, 130.6, 130.3, 129.6, 129.3, 128.9, 128.9, 128.9, 128.1, 127.9, 127.7, 126.3, 125.7, 100.9, 70.8, 70.4, 56.0, 52.5, 21.8, 18.3. **HRMS** (ESI-TOF) m/z: $[M+Na]^+$ Calcd for $C_{38}H_{30}ClN_3NaO_6S^+$ 714.1436, Found 714.1433.

Methyl (5*R*,6*R*,11*R*,*E*)-1-ethyl-4-oxo-3,9,11-triphenyl-7-(tosylimino)-8-oxa-2,3-diazadispiro[4.0.4^6.3^5]trideca-1,9,12-triene-13-carboxylate (3j)

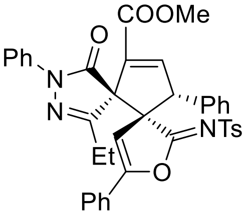

27.3 mg, 45% yield, white solid, m.p.: 237.3 – 237.4 ℃; **HPLC** (Daicel Chiralpak IE, n-hexane/2-propanol = 70:30, 1.0 mL/min, at 254 nm): t_R = 25.31 min (major), t_R = 28.72 min (minor); er = 91.5:8.5, $[\alpha]_D^{35}$ = + 50.303 (c = 0.015, DCM). **^{1}H NMR** (700 MHz, $CDCl_3$) δ 7.83 (dd, *J* = 8.4, 1.4 Hz, 2H), 7.49 (d, *J* = 2.1 Hz, 1H), 7.41 (d, *J* = 8.4 Hz, 2H), 7.34 (dd, *J* = 8.4, 7.7 Hz, 2H), 7.29 (tt, *J* = 7.0, 1.4 Hz, 1H), 7.22 (dd, *J* = 8.4, 7.0 Hz, 2H), 7.20 – 7.18 (m, 1H), 7.16 – 7.13 (m, 5H), 7.10 (d, *J* = 7.0 Hz, 2H), 7.05 (d, *J* = 7.0, 2H), 5.86 (s, 1H), 5.28 (d, *J* = 2.1 Hz, 1H), 3.68 (s, 3H), 2.50 (dq, *J* = 17.5, 7.0 Hz, 1H), 2.39 (dq, *J* = 17.5, 7.0 Hz, 1H), 2.34 (s, 3H), 1.16 (t, *J* = 7.0 Hz, 3H). **^{13}C NMR** (176 MHz, $CDCl_3$) δ 171.1, 166.0, 162.8, 162.1, 156.1, 150.1, 144.1, 137.9, 137.8, 134.7, 133.9, 131.0, 129.6, 129.3, 129.1, 128.9, 128.9, 128.9, 128.1, 127.7, 126.2, 125.8, 125.7, 119.4, 100.1, 71.8, 71.4, 56.0, 52.5, 25.6, 21.8, 10.7. **HRMS** (ESI-TOF) m/z: $[M+Na]^+$ Calcd for $C_{39}H_{33}N_3NaO_6S^+$ 694.1982, Found 694.1983.

Ethyl (5*R*,6*R*,11*R*,*E*)-1-methyl-4-oxo-3,9,11-triphenyl-7-(tosylimino)-8-oxa-2,3-diazadispiro[4.0.4^6.3^5]trideca-1,9,12-triene-13-carboxylate (3k)

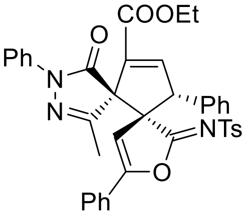

53.4 mg, 79% yield, white solid, m.p.: 205.6 – 205.7 ℃; **HPLC** (Daicel Chiralpak IE, n-hexane/2-propanol = 70:30, 1.0 mL/min, at 254 nm): t_R = 31.14 min (major), t_R = 36.26 min (minor); er = 92:8, $[\alpha]_D^{35}$ = + 663.623 (c = 0.015, DCM). **^{1}H NMR** (600 MHz, $CDCl_3$) δ 7.81 (dd, *J* = 9.0, 1.2 Hz, 2H), 7.50 (d, *J* = 2.4 Hz, 1H), 7.39 (d, *J* = 8.4 Hz, 2H), 7.33 (dd, *J* = 8.4, 7.2 Hz, 2H), 7.30 – 7.26 (m, 1H), 7.23 – 7.18 (m, 3H), 7.14 (td, *J* = 7.8, 6.0 Hz, 5H), 7.09 (d, *J* = 7.2 Hz, 2H), 7.05 (d, *J* = 7.2 Hz, 2H), 5.87 (s, 1H), 5.27 (d, *J* = 2.4 Hz, 1H), 4.19 – 4.07 (m, 2H), 2.33 (s, 3H), 2.18 (s, 3H), 1.18 (t, *J* = 7.2 Hz, 3H). **^{13}C NMR** (151 MHz, $CDCl_3$) δ 171.1, 165.8, 162.2, 158.3, 156.1, 150.2, 144.0, 137.9, 137.6, 134.6,

133.9, 131.0, 129.5, 129.3, 129.1, 128.9, 128.8, 128.1, 127.6, 126.1, 125.8, 125.7, 119.4, 100.0, 71.8, 71.3, 61.7, 56.0, 21.8, 18.3, 14.2. **HRMS** (ESI-TOF) m/z: $[M+Na]^+$ Calcd for $C_{39}H_{33}N_3NaO_6S^+$ 694.1982, Found 694.1986.

Methyl (5*R*,6*R*,11*R*,*E*)-1-methyl-7-(((4-nitrophenyl)sulfonyl)imino)-4-oxo-3,9,11-triphenyl-8-oxa-2,3-diazadispiro[4.0.4^6.3^5]trideca-1,9,12-triene-13-carboxylate (3l)

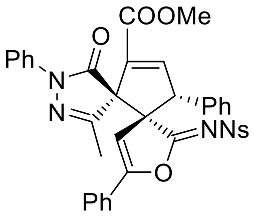

67.0 mg, 97% yield, white solid, m.p.: 236.5 ℃; **HPLC** (Daicel Chiralpak IF, n-hexane/2-propanol = 70:30, 1.0 mL/min, at 254 nm): t_R = 20.46 min (major), t_R = 37.98 min (minor); er = 95:5, $[\alpha]_D^{35}$ = + 649.000 (c = 0.015, DCM). **^{1}H NMR** (600 MHz, $CDCl_3$) δ 8.24 (d, *J* = 9.0 Hz, 2H), 7.88 (dd, *J* = 8.4, 1.2 Hz, 2H), 7.73 (d, *J* = 9.0 Hz, 2H), 7.59 (d, *J* = 1.8 Hz, 1H), 7.45 – 7.37 (m, 3H), 7.34 – 7.28 (m, 3H), 7.27 – 7.21 (m, 3H), 7.17 (d, *J* = 7.8 Hz, 2H), 7.11 (d, *J* = 7.2 Hz, 2H), 6.00 (s, 1H), 5.39 (d, *J* = 2.4 Hz, 1H), 3.78 (s, 3H), 2.27 (s, 3H). **^{13}C NMR** (151 MHz, $CDCl_3$) δ 170.7, 168.1, 162.5, 157.6, 156.3, 150.4, 150.0, 146.3, 137.5, 134.5, 134.0, 131.4, 129.2, 129.1, 129.0, 129.0, 128.9, 128.3, 126.0, 125.7, 125.6, 124.2, 119.4, 100.6, 71.7, 71.4, 55.8, 52.7, 18.2. **HRMS** (ESI-TOF) m/z: $[M+Na]^+$ Calcd for $C_{37}H_{28}N_4NaO_8S^+$ 711.1520, Found 711.1521.

Methyl (5*R*,6*R*,11*R*,*E*)-1-methyl-4-oxo-3,9,11-triphenyl-7-(tosylimino)-8-oxa-2,3-diazadispiro[4.0.4^6.3^5]trideca-1,9,12-triene-13-carboxylate (3m)

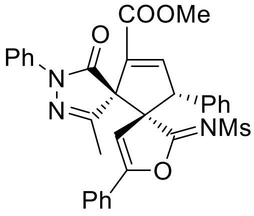

59.1 mg, 99% yield, white solid, m.p.: 212.6 – 215.9 ℃; **HPLC** (Daicel Chiralpak IF, n-hexane/2-propanol = 70:30, 1.0 mL/min, at 254 nm): t_R = 15.43 min (major), t_R = 25.96 min (minor); er = 93:7, $[\alpha]_D^{35}$ = + 960.556 (c = 0.015, DCM). **^{1}H NMR** (600 MHz, $CDCl_3$) δ 7.83 (dd, *J* = 8.4, 1.2 Hz, 2H), 7.56 (d, *J* = 2.4 Hz, 1H), 7.38 (d, *J* = 7.2 Hz, 2H), 7.35 (dt, *J* = 9.0, 7.2 Hz, 3H), 7.29 (t, *J* = 7.2 Hz, 2H), 7.27 – 7.20 (m, 3H), 7.16 (td, *J* = 8.4, 1.8 Hz, 3H), 5.95 (s, 1H), 5.33 (d, *J* = 1.8 Hz, 1H), 3.71 (s, 3H), 2.66 (s, 3H), 2.20 (s, 3H). **^{13}C NMR** (151 MHz, $CDCl_3$) δ 171.0, 167.8, 162.6, 157.9, 156.4, 150.0, 137.6, 134.7, 134.1, 131.3, 129.2, 129.1, 128.9, 128.5, 126.0, 125.9, 125.8, 119.4, 100.3, 71.6, 71.1, 56.2, 52.6, 41.9, 18.4. **HRMS** (ESI-TOF) m/z: $[M+Na]^+$ Calcd for $C_{32}H_{27}N_3NaO_6S^+$ 604.1513, Found 604.1519.

Methyl (5*R*,6*R*,11*R*,*E*)-3-(tert-butyl)-9-(4-chlorophenyl)-1-methyl-4-oxo-11-phenyl-7-(tosylimino)-8-oxa-2,3-diazadispiro[4.0.4^6.3^5]trideca-1,9,12-triene-13-carboxylate (3n)

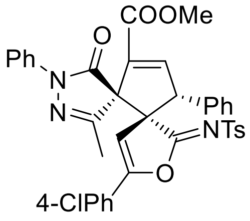

52.5 mg, 78% yield, white solid, m.p.: 211.6 ℃; **HPLC** (Daicel Chiralpak IF, n-hexane/2-propanol = 70:30, 1.0 mL/min, at 254 nm): t_R = 16.51 min (minor), t_R = 17.92 min (major); er = 97:3, $[\alpha]_D^{35}$ = + 577.292 (c = 0.020, DCM). **^{1}H NMR** (700 MHz, $CDCl_3$) δ 7.40 (d, *J* = 2.1 Hz, 1H), 7.37 (d, *J* = 8.4 Hz, 2H), 7.24 (d, *J* = 8.4 Hz, 2H), 7.18 (d, *J* = 7.7 Hz, 1H), 7.14 – 7.11 (m, 4H), 7.07 (d, *J* = 9.1 Hz, 2H), 7.00 (d, *J* = 7.0 Hz, 2H), 5.89 (s, 1H), 5.17 (d, *J* = 2.1 Hz, 1H), 3.69 (s, 3H), 2.34 (s, 3H), 2.01 (s, 3H), 1.43 (s, 9H). **^{13}C NMR** (176 MHz, $CDCl_3$) δ 172.6, 165.8, 162.7, 155.4, 154.5, 149.7, 144.0, 138.0, 137.0, 134.7, 134.1, 129.5, 129.3, 129.2, 128.8, 128.1, 127.6, 126.7, 124.8, 101.3, 71.6, 70.7, 58.1, 55.9, 52.3, 28.3, 21.8, 18.1. **HRMS** (ESI-TOF) m/z: $[M+Na]^+$ Calcd for $C_{36}H_{34}ClN_3NaO_6S^+$ 694.1749, Found 694.1747.

Methyl (5*R*,6*R*,11*R*,*E*)-3-(tert-butyl)-9-(4-methoxyphenyl)-1-methyl-4-oxo-11-phenyl-7-(tosylimino)-8-oxa-2,3-diazadispiro[4.0.4^6.3^5]trideca-1,9,12-triene-13-carboxylate (3o)

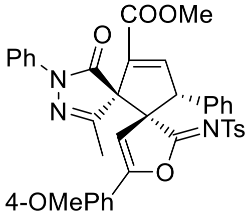

67.5 mg, 99% yield, white solid, m.p.: 352.1 ℃; **HPLC** (Daicel Chiralpak IC, n-hexane/2-propanol = 80:20, 1.0 mL/min, at 254 nm): t_R = 30.76 min (minor), t_R = 33.69 min (major); er = 94:6, $[\alpha]_D^{35}$ = + 537.333 (c = 0.020, DCM). **^{1}H NMR** (700 MHz, $CDCl_3$) δ 7.48 (d, *J* = 2.1 Hz, 1H), 7.43 (d, *J* = 7.7 Hz, 2H), 7.24 (t, *J* = 7.0 Hz, 1H), 7.22 – 7.16 (m, 4H), 7.14 (d, *J* = 8.4 Hz, 2H), 7.09 (d, *J* = 7.0 Hz, 2H), 6.83 (d, *J* = 9.1 Hz, 2H), 5.80 (s, 1H), 5.22 (d, *J* = 2.1 Hz, 1H), 3.82 (s, 3H), 3.76 (s, 3H), 2.40 (s, 3H), 2.08 (s, 3H), 1.51 (s, 9H). **^{13}C NMR** (151 MHz, $CDCl_3$) δ 172.8, 166.5, 162.8, 161.6, 155.5, 155.4, 149.9, 143.8, 138.1, 134.6, 134.3, 129.5, 129.3, 128.8, 127.9, 127.6, 127.1, 119.0, 114.4, 98.3, 71.7, 70.8, 58.1, 55.8, 55.6, 52.3, 28.3, 21.8, 18.1. **HRMS** (ESI-TOF) m/z: $[M+Na]^+$ Calcd for $C_{37}H_{37}N_3NaO_7S^+$ 690.2244, Found 690.2246.

Methyl (5*R*,6*R*,11*R*,*E*)-9-(3-chlorophenyl)-1-methyl-4-oxo-3,11-diphenyl-7-(tosylimino)-8-oxa-2,3-diazadispiro[4.0.4^6.3^5]trideca-1,9,12-triene-13-carboxylate (3p)

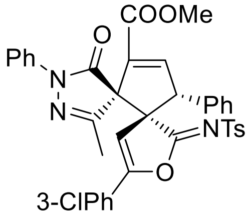

42.6 mg, 62% yield, white solid, m.p.: 225.7 ℃; **HPLC** (Daicel Chiralpak IE, n-hexane/2-propanol = 70:30, 1.0 mL/min, at 254 nm): t_R = 29.86 min (major), t_R = 36.56 min (minor); er = 95:5, $[\alpha]_D^{35}$ = +

654.286 (c = 0.020, DCM). **^{1}H NMR** (600 MHz, $CDCl_3$) δ 7.81 (dd, J = 8.7, 1.1 Hz, 2H), 7.51 (d, J = 2.4 Hz, 1H), 7.33 (dd, J = 15.0, 8.4 Hz, 4H), 7.26 – 7.21 (m, 2H), 7.20 (t, J = 7.2 Hz, 2H), 7.18 – 7.11 (m,4H), 7.06 (d, J = 6.6 Hz, 2H), 7.02 (dt, J = 7.8, 1.2 Hz, 1H), 6.83 (t, J = 1.8 Hz, 1H), 5.89 (s, 1H), 5.28 (d, J = 2.4 Hz, 1H), 3.70 (s, 3H), 2.33 (s, 3H), 2.20 (s, 3H). **^{13}C NMR** (151 MHz, $CDCl_3$) δ 170.9, 164.8, 162.6, 158.1, 154.7, 150.3, 144.2, 137.8, 137.6, 135.1, 134.4, 133.8, 131.0, 130.2, 129.7, 129.3, 129.2, 129.0, 128.2, 127.7, 127.5, 125.9, 125.5, 123.8, 119.4, 101.5, 71.8, 71.1, 56.1, 52.5, 21.8, 18.3. **HRMS** (ESI-TOF) m/z: $[M+Na]^+$ Calcd for $C_{38}H_{30}ClN_3NaO_6S^+$ 714.1436, Found 714.1436.

Methyl (5*R*,6*R*,11*R*,*E*)-9-(3-methoxyphenyl)-1-methyl-4-oxo-3,11-diphenyl-7-(tosylimino)-8-oxa-2,3-diazadispiro[4.0.4^6.3^5]trideca-1,9,12-triene-13-carboxylate (3q)

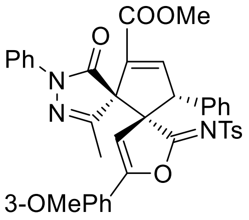

54.6 mg, 79% yield, white solid, m.p.: 201.8 ℃; **HPLC** (Daicel Chiralpak IE, n-hexane/2-propanol = 70:30, 1.0 mL/min, at 254 nm): t_R = 36.31 min (major), t_R = 51.52 min (minor); er = 92:8, $[\alpha]_D^{35}$ = + 683.571 (c = 0.020, DCM). **^{1}H NMR** (600 MHz, $CDCl_3$) δ 7.80 (dd, J = 8.4, 1.2 Hz, 2H), 7.51 (d, J = 2.4 Hz, 1H), 7.36 (d, J = 8.4 Hz, 2H), 7.32 (td, J = 7.2, 1.8 Hz, 2H), 7.19 (t, J = 7.2 Hz, 1H), 7.15 (t, J = 7.2 Hz, 3H), 7.11 (dt, J = 8.4, 4.2 Hz, 3H), 7.04 (d, J = 7.2 Hz, 2H), 6.81 (ddd, J = 8.4, 2.4, 1.2 Hz, 1H), 6.68 (dt, J = 7.8, 1.2 Hz, 1H), 6.53 (dd, J = 2.4, 1.8 Hz, 1H), 5.84 (s, 1H), 5.26 (d, J = 1.8 Hz, 1H), 3.69 (s, 3H), 3.65 (s, 3H), 2.32 (s, 3H), 2.18 (s, 3H). **^{13}C NMR** (151 MHz, $CDCl_3$) δ 171.0, 165.6, 162.6, 159.9, 158.2, 156.0, 150.4, 144.1, 137.8, 137.6, 134.3, 133.8, 130.0, 129.6, 129.2, 129.1, 128.9, 128.1, 127.6, 127.3, 125.8, 119.4, 118.1, 116.4, 111.3, 100.3, 71.8, 71.3, 55.9, 55.5, 52.5, 21.7, 18.3. **HRMS** (ESI-TOF) m/z: $[M+Na]^+$ Calcd for $C_{39}H_{33}N_3NaO_7S^+$ 710.1931, Found 710.1931.

Methyl (5*R*,6*R*,11*R*,*E*)-9-(2-fluorophenyl)-1-methyl-4-oxo-3,11-diphenyl-7-(tosylimino)-8-oxa-2,3-diazadispiro[4.0.4^6.3^5]trideca-1,9,12-triene-13-carboxylate (3r)

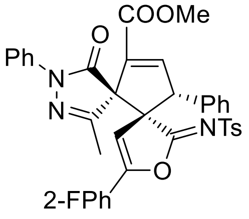

38.9 mg, 56% yield, white solid, m.p.: 216.2 °C; **HPLC** (Daicel Chiralpak IF, n-hexane/2-propanol = 70:30, 1.0 mL/min, at 254 nm): t_R = 24.79 min (major), t_R = 46.46 min (minor); er = 93.5:6.5, $[\alpha]_D^{35}$ = + 664.167 (c = 0.020, DCM). **^{1}H NMR** (600 MHz, Chloroform-*d*) δ 7.78 (dd, J = 8.4, 1.2 Hz, 2H), 7.50 (d, J = 2.4 Hz, 1H), 7.42 (d, J = 8.4 Hz, 2H), 7.34 (dd, J = 8.4, 7.2 Hz, 2H), 7.27 (dddd, J = 9.0, 7.8, 5.4, 1.8 Hz, 1H), 7.21 – 7.19 (m, 1H), 7.17 – 7.13 (m, 5H), 7.05 (d, J = 7.2 Hz, 2H), 7.01 – 6.96 (m, 2H), 6.93 (td, J = 7.8, 1.8 Hz, 1H), 6.13 (d, J = 2.4 Hz, 1H), 5.30 (d, J = 1.8 Hz, 1H), 3.70 (s, 3H), 2.34 (s, 3H), 2.18 (s, 3H). **^{13}C NMR** (151 MHz, $CDCl_3$) δ 170.9, 165.2, 162.7, 160.6 (d, $^1J_{CF}$ = 255.2 Hz), 159.8, 158.0, 150.5, 150.2 (d, $^3J_{CF}$ = 3.0 Hz), 144.1, 137.9, 137.5, 134.3, 133.8, 132.2 (d, $^3J_{CF}$ = 9.1 Hz), 129.6, 129.3, 129.1, 128.9, 128.2, 127.8, 127.6, 125.9, 124.5 (d, $^3J_{CF}$ = 3.0 Hz), 119.7, 116.3 (d, $^2J_{CF}$ = 21.1 Hz),

114.8 (d, $^2J_{CF}$ = 10.6 Hz), 105.8 (d, $^4J_{CF}$ = 1.5 Hz), 71.7, 71.6, 56.0, 52.5, 21.8, 18.3. **HRMS** (ESI-TOF) m/z: $[M+Na]^+$ Calcd for $C_{38}H_{30}FN_3NaO_6S^+$ 698.1732, Found 698.1736.

Methyl (5*R*,6*R*,11*R*,*E*)-1-methyl-4-oxo-3,11-diphenyl-9-(o-tolyl)-7-(tosylimino)-8-oxa-2,3-diazadispiro[4.0.4⁶.3⁵]trideca-1,9,12-triene-13-carboxylate (3s)

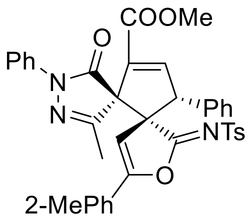

36.3 mg, 54% yield, white solid, m.p.: 212.0 – 222.9 ℃; **HPLC** (Daicel Chiralpak IF, n-hexane/2-propanol = 70:30, 1.0 mL/min, at 254 nm): t_R = 21.34 min (major), t_R = 43.61 min (minor); er = 93:7, $[\alpha]_D^{35}$ = + 545.385 (c = 0.020, DCM). **¹H NMR** (600 MHz, $CDCl_3$) δ 7.90 (d, *J* = 8.4 Hz, 2H), 7.60 (d, *J* = 2.4 Hz, 1H), 7.45 (d, *J* = 8.4 Hz, 2H), 7.41 (t, *J* = 7.8 Hz, 2H), 7.30 (t, *J* = 7.2 Hz, 1H), 7.27 (s, 1H), 7.26 – 7.17 (m, 5H), 7.14 (dd, *J* = 7.2, 2.4 Hz, 3H), 7.07 (t, *J* = 7.8 Hz, 1H), 6.80 (dd, *J* = 7.8, 1.2 Hz, 1H), 5.74 (s, 1H), 5.35 (d, *J* = 2.4 Hz, 1H), 3.78 (s, 3H), 2.42 (s, 3H), 2.26 (s, 3H), 2.11 (s, 3H). **¹³C NMR** (151 MHz, $CDCl_3$) δ 171.0, 166.0, 162.7, 158.3, 155.9, 150.6, 144.0, 137.9, 137.6, 137.2, 134.2, 133.9, 131.4, 130.7, 129.5, 129.3, 129.1, 128.9, 128.4, 128.2, 127.7, 126.1, 125.9, 125.8, 119.2, 104.7, 72.0, 71.6, 55.6, 52.5, 21.8, 21.5, 18.2. **HRMS** (ESI-TOF) m/z: $[M+Na]^+$ Calcd for $C_{39}H_{33}N_3NaO_6S^+$ 694.1982, Found 694.1984.

Methyl (5*R*,6*S*,11*S*,*E*)-1-methyl-4-oxo-3,9-diphenyl-11-(thiophen-2-yl)-7-(tosylimino)-8-oxa-2,3-diazadispiro[4.0.4⁶.3⁵]trideca-1,9,12-triene-13-carboxylate (3t)

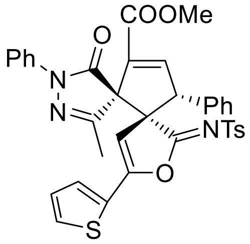

49.1 mg, 74% yield, white solid, m.p.: 221.0 – 221.2 ℃; **HPLC** (Daicel Chiralpak IF, n-hexane/2-propanol = 70:30, 1.0 mL/min, at 254 nm): t_R = 29.87 min (major), t_R = 65.90 min (minor); er = 91:9, $[\alpha]_D^{35}$ = + 645.208 (c = 0.015, DCM). **¹H NMR** (700 MHz, $CDCl_3$) δ 7.79 (d, *J* = 8.4 Hz, 2H), 7.49 (d, *J* = 2.1 Hz, 1H), 7.41 (d, *J* = 7.7 Hz, 2H), 7.33 (t, *J* = 7.7 Hz, 2H), 7.27 (t, *J* = 3.5 Hz, 1H), 7.19 – 7.16 (m, 1H), 7.16 – 7.13 (m, 3H), 7.12 (d, *J* = 7.7 Hz, 2H), 7.04 (d, *J* = 7.0 Hz, 2H), 6.88 (d, *J* = 2.8 Hz, 2H), 5.68 (s, 1H), 5.24 (d, *J* = 2.1 Hz, 1H), 3.69 (s, 3H), 2.32 (s, 3H), 2.17 (s, 3H). **¹³C NMR** (176 MHz, $CDCl_3$) δ 171.0, 165.5, 162.6, 158.1, 151.1, 150.4, 144.1, 137.6, 137.6, 134.2, 133.8, 129.5, 129.2, 129.1, 128.9, 128.4, 128.2, 128.1, 128.0, 127.8, 125.9, 119.5, 98.8, 71.8, 71.4, 56.0, 52.5, 21.8, 18.3. **HRMS** (ESI-TOF) m/z: $[M+Na]^+$ Calcd for $C_{36}H_{29}N_3NaO_6S_2^+$ 686.1390, Found 686.1398.

Methyl (5*R*,6*R*,11*R*,*E*)-11-(tert-butyl)-1-methyl-4-oxo-3,9-diphenyl-7-(tosylimino)-8-oxa-2,3-diazadispiro[4.0.4⁶.3⁵]trideca-1,9,12-triene-13-carboxylate (3u)

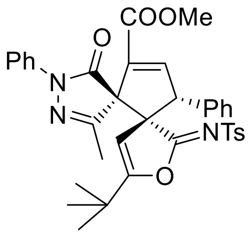

33.7 mg, 53% yield, white solid, m.p.: 221.0 – 229.0 ℃; **HPLC** (Daicel Chiralpak IF, n-hexane/2-propanol = 70:30, 1.0 mL/min, at 254 nm): t_R = 18.07 min (major), t_R = 29.73 min (minor); er = 84:16, $[\alpha]_D^{35}$ = + 120.909 (c = 0.015, DCM). **^{1}H NMR** (600 MHz, $CDCl_3$) δ 7.77 (dd, *J* = 9.0, 1.2 Hz, 2H), 7.50 (d, *J* = 1.8 Hz, 1H), 7.34 (dd, *J* = 8.4, 7.2 Hz, 2H), 7.27 (d, *J* = 8.4 Hz, 2H), 7.23 – 7.20 (m, 3H), 7.15 (tt, *J* = 7.2, 1.2 Hz, 1H), 7.06 – 7.04 (m, 4H), 5.15 (s, 1H), 5.14 (d, *J* = 1.8 Hz, 1H), 3.70 (s, 3H), 2.29 (s, 3H), 2.15 (s, 3H), 0.74 (s, 9H). **^{13}C NMR** (151 MHz, $CDCl_3$) δ 171.0, 168.1, 166.8, 162.7, 158.3, 150.5, 143.8, 137.9, 137.6, 134.2, 134.1, 129.4, 129.2, 129.1, 128.7, 128.0, 127.7, 125.9, 119.5, 98.8, 71.6, 70.5, 55.0, 52.5, 32.4, 26.9, 21.7, 18.1. **HRMS** (ESI-TOF) m/z: $[M+Na]^+$ Calcd for $C_{36}H_{35}N_3NaO_6S^+$ 660.2139, Found 660.2145.

Methyl (5*R*,6*R*,11*S*,*E*)-11-butyl-1-methyl-4-oxo-3,9-diphenyl-7-(tosylimino)-8-oxa-2,3-diazadispiro[4.0.4^6.3^5]trideca-1,9,12-triene-13-carboxylate (3v)

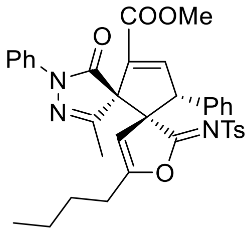

32.4 mg, 51% yield, white solid, m.p.: 227.5 – 232.1 ℃; **HPLC** (Daicel Chiralpak IF, n-hexane/2-propanol = 70:30, 1.0 mL/min, at 254 nm): t_R = 19.93 min (major), t_R = 29.01 min (minor); er = 87 : 13, $[\alpha]_D^{35}$ = + 443.667 (c = 0.020, DCM). **^{1}H NMR** (600 MHz, $CDCl_3$) δ 7.82 (d, *J* = 7.2 Hz, 2H), 7.46 (d, *J* = 2.4 Hz, 1H), 7.38 (d, *J* = 8.4 Hz, 2H), 7.34 (dd, *J* = 8.4, 7.2 Hz, 2H), 7.24 – 7.18 (m, 1H), 7.20 – 7.15 (m, 2H), 7.15 (t, *J* = 6.6 Hz, 1H), 7.09 (d, *J* = 7.8 Hz, 2H), 7.02 (d, *J* = 6.6 Hz, 2H), 5.23 (s, 1H), 5.14 (d, *J* = 1.8 Hz, 1H), 3.68 (s, 3H), 2.33 (s, 3H), 2.15 (s, 3H), 2.05 – 1.99 (m, 2H), 1.12 – 1.00 (m, 4H), 0.70 (t, *J* = 7.2 Hz, 3H). **^{13}C NMR** (151 MHz, $CDCl_3$) δ 171.0, 167.3, 162.7, 160.2, 158.2, 150.7, 143.9, 137.7, 137.7, 134.2, 133.9, 129.4, 129.3, 129.1, 128.7, 128.1, 127.8, 125.7, 119.2, 101.9, 71.5, 70.8, 55.2, 52.5, 27.7, 27.1, 21.8, 21.7, 18.2, 13.6. **HRMS** (ESI-TOF) m/z: $[M+Na]^+$ Calcd for $C_{36}H_{35}N_3NaO_6S^+$ 660.2139, Found 660.2147.

Methyl (5*R*,6*R*,11*R*,*E*)-1-methyl-11-(naphthalen-1-yl)-4-oxo-3,9-diphenyl-7-(tosylimino)-8-oxa-2,3-diazadispiro[4.0.4^6.3^5]trideca-1,9,12-triene-13-carboxylate (3w)

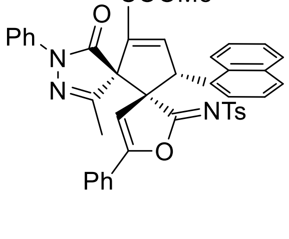

37.5 mg, 53% yield, white solid, m.p.: 166.1 – 175.7 ℃; **HPLC** (Daicel Chiralpak IF, n-hexane/2-propanol = 70:30, 1.0 mL/min, at 254 nm): t_R = 37.27 min (major), t_R = 72.57 min (minor); er = 86:14, $[\alpha]_D^{35}$ = + 140.980 (c = 0.015, DCM). **^{1}H NMR** (600 MHz, $CDCl_3$) δ 7.85 – 7.79 (m, 3H), 7.73 – 7.67 (m, 2H), 7.50 (d, *J* = 2.4 Hz, 1H), 7.47 (d, *J* = 8.4 Hz, 2H), 7.45 (dd, *J* = 7.2, 1.2 Hz, 1H), 7.35 (dd, *J* = 8.4, 7.2 Hz, 2H), 7.30 (dd, *J* = 8.4, 7.2 Hz, 1H), 7.29 – 7.24 (m, 2H), 7.23 – 7.18 (m, 1H), 7.16 (t, *J* = 7.2 Hz, 1H), 7.14 – 7.10 (m, 4H), 6.98 (d, *J* = 7.2 Hz, 2H), 6.16 (d, *J* = 2.4 Hz, 1H), 6.01 (s, 1H), 3.72 (s, 3H), 2.32 (s, 3H), 2.18 (s, 3H). **^{13}C NMR** (151 MHz, $CDCl_3$) δ 171.4, 165.6, 162.7, 158.2, 155.7, 152.1, 144.2, 137.9, 137.6, 134.2, 133.8, 131.8, 130.8, 130.1, 129.6, 129.3, 129.2, 129.2, 129.0, 128.7, 127.7, 126.1, 126.1, 125.9, 125.8, 125.6, 125.5, 123.4, 119.6, 100.7, 71.9, 71.5, 52.6, 52.6, 21.8, 18.4. **HRMS** (ESI-TOF) m/z: $[M+Na]^+$ Calcd for $C_{42}H_{33}N_3NaO_6S^+$ 730.1982, Found 730.1981.

Methyl (5*R*,6*R*,11*R*,*E*)-11-(4-chlorophenyl)-1-methyl-4-oxo-3,9-diphenyl-7-(tosylimino)-8-oxa-2,3-diazadispiro[4.0.4^6.3^5]trideca-1,9,12-triene-13-carboxylate (3x)

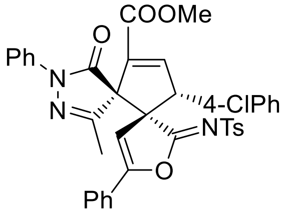

57.2mg, 83% yield, white solid, m.p.: 220.8 – 220.9 ℃; **HPLC** (Daicel Chiralpak IF, n-hexane/2-propanol = 70:30, 1.0 mL/min, at 254 nm): t_R = 23.75 min (major), t_R = 42.61 min (minor); er = 94:6, $[\alpha]_D^{35}$ = + 791.333 (c = 0.020, DCM). **^{1}H NMR** (600 MHz, $CDCl_3$) δ 7.80 (dd, *J* = 8.4, 1.2 Hz, 2H), 7.46 (d, *J* = 8.4 Hz, 2H), 7.43 (d, *J* = 1.8 Hz, 1H), 7.32 (dd, *J* = 8.4, 7.2 Hz, 2H), 7.32 – 7.28 (m, 1H), 7.23 (t, *J* = 7.8 Hz, 2H), 7.17 – 7.12 (m, 5H), 7.01 (d, *J* = 8.4 Hz, 2H), 6.93 (d, *J* = 8.4 Hz, 2H), 5.84 (s, 1H), 5.22 (d, *J* = 2.4 Hz, 1H), 3.69 (s, 3H), 2.35 (s, 3H), 2.21 (s, 3H). **^{13}C NMR** (151 MHz, $CDCl_3$) δ 170.8, 165.9, 162.5, 158.0, 156.4, 149.5, 144.5, 137.5, 137.4, 134.7, 134.2, 132.4, 131.2, 130.4, 129.6, 129.1, 129.0, 128.9, 127.8, 125.9, 125.8, 125.7, 119.4, 99.7, 71.8, 71.3, 55.2, 52.5, 21.8, 18.3. **HRMS** (ESI-TOF) m/z: $[M+Na]^+$ Calcd for $C_{38}H_{30}ClN_3NaO_6S^+$ 714.1436, Found 714.1436.

Methyl (5*R*,6*R*,11*R*,*E*)-11-(3-fluorophenyl)-1-methyl-4-oxo-3,9-diphenyl-7-(tosylimino)-8-oxa-2,3-diazadispiro[4.0.4^6.3^5]trideca-1,9,12-triene-13-carboxylate(3y)

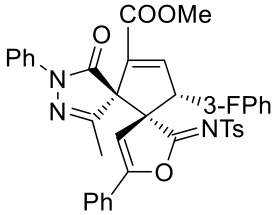

48.6 mg, 72% yield, white solid, m.p.: 231.7 – 231.8 °C; **HPLC** (Daicel Chiralpak IE, n-hexane/2-propanol = 70:30, 1.0 mL/min, at 254 nm): t_R = 28.31 min (major), t_R = 36.63 min (minor); er = 92:8, $[\alpha]_D^{35}$ = + 536.212 (c = 0.020, DCM). **^{1}H NMR** (600 MHz, $CDCl_3$) δ 7.80 (dd, *J* = 8.4, 1.2 Hz, 2H), 7.52 (d, *J* = 8.4 Hz, 2H), 7.42 (d, *J* = 1.8 Hz, 1H), 7.33 (dd, *J* = 8.4, 7.2 Hz, 2H), 7.30 (d, *J* = 7.2 Hz, 1H), 7.24 (dd, *J* = 8.4, 7.2 Hz, 2H), 7.23 – 7.18 (m, 2H), 7.18 (s, 1H), 7.16 (s, 1H), 7.14 (t, *J* = 8.4 Hz, 1H), 7.08 (td, *J* = 7.8, 6.0 Hz, 1H), 6.85 (td, *J* = 8.4, 2.4 Hz, 1H), 6.80 (d, *J* = 7.8 Hz, 1H), 6.74 (dt, *J* = 9.6, 2.4 Hz, 1H), 5.85 (s, 1H), 5.24 (d, *J* = 1.8 Hz, 1H), 3.68 (s, 3H), 2.34 (s, 3H), 2.18 (s, 3H). **^{13}C NMR**

(151 MHz, $CDCl_3$) δ 170.8, 166.0, 162.5, 162.2 (d, $^1J_{CF}$ = 247.6 Hz), 157.9, 156.5, 149.5, 144.3, 137.6, 137.6, 136.3 (d, $^3J_{CF}$ = 7.6 Hz), 134.7, 131.2, 130.4 (d, $^3J_{CF}$ = 7.6 Hz), 129.6, 129.1, 129.0, 127.7, 126.0, 125.9, 125.8, 124.7 (d, $^4J_{CF}$ = 3.0 Hz), 119.4, 116.3 (d, $^2J_{CF}$ = 22.7 Hz), 115.2 (d, $^2J_{CF}$ = 21.1 Hz), 99.6, 71.7, 71.2, 55.4, 52.5, 21.8, 18.2. **HRMS** (ESI-TOF) m/z: $[M+Na]^+$ Calcd for $C_{38}H_{30}FN_3NaO_6S^+$ 698.1732, Found 698.1732.

Methyl (5*R*,6*R*,11*R*,*E*)-11-(3-chlorophenyl)-1-methyl-4-oxo-3,9-diphenyl-7-(tosylimino)-8-oxa-2,3-diazadispiro[4.0.4^6.3^5]trideca-1,9,12-triene-13-carboxylate (3z)

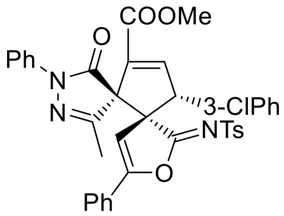

53.0 mg, 77% yield, white solid, m.p.: 216.7 ℃; **HPLC** (Daicel Chiralpak IF, n-hexane/2-propanol = 70:30, 1.0 mL/min, at 254 nm): t_R = 23.93 min (major), t_R = 49.92 min (minor); er = 90:10, $[\alpha]_D^{35}$ = + 490.864 (c = 0.020, DCM). **^{1}H NMR** (600 MHz, $CDCl_3$) δ 7.88 (dd, *J* = 8.4, 1.2 Hz, 2H), 7.60 (d, *J* = 8.4 Hz, 2H), 7.52 (d, *J* = 2.4 Hz, 1H), 7.41 (dd, *J* = 8.4, 7.2 Hz, 2H), 7.39 – 7.37 (m, 1H), 7.32 (td, *J* = 7.2, 1.8 Hz, 2H), 7.28 – 7.26 (m, 2H), 7.26 – 7.24 (m, 2H), 7.24 – 7.21 (m, 2H), 7.14 (t, *J* = 1.8 Hz, 1H), 7.12 (t, *J* = 7.8 Hz, 1H), 6.98 (d, *J* = 7.8 Hz, 1H), 5.92 (s, 1H), 5.31 (d, *J* = 1.8 Hz, 1H), 3.76 (s, 3H), 2.42 (s, 3H), 2.25 (s, 3H). **^{13}C NMR** (151 MHz, $CDCl_3$) δ 170.8, 166.0, 162.5, 157.9, 156.6, 149.3, 144.2, 137.7, 137.6, 136.0, 134.7, 131.2, 130.1, 129.7, 129.3, 129.1, 129.0, 128.5, 127.7, 127.1, 126.0, 125.9, 125.8, 119.4, 99.7, 71.7, 71.1, 55.4, 52.6, 21.8, 18.2. **HRMS** (ESI-TOF) m/z: $[M+Na]^+$ Calcd for $C_{38}H_{30}ClN_3NaO_6S^+$ 714.1436, Found 714.1437.

Methyl (5*R*,6*R*,11*S*,*E*)-11-(2-chlorophenyl)-1-methyl-4-oxo-3,9-diphenyl-7-(tosylimino)-8-oxa-2,3-diazadispiro[4.0.4^6.3^5]trideca-1,9,12-triene-13-carboxylate (3aa)

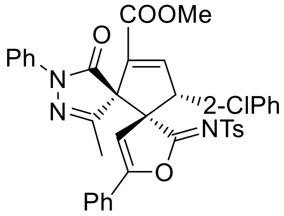

52.5 mg, 76% yield, white solid, m.p.: 197.7 – 206.2 ℃; **HPLC** (Daicel Chiralpak IE, n-hexane/2-propanol = 70:30, 1.0 mL/min, at 254 nm): t_R = 24.61 min (major), t_R = 34.49 min (minor); er = 95:5, $[\alpha]_D^{35}$ = + 605.119 (c = 0.015, DCM). **^{1}H NMR** (600 MHz, $CDCl_3$) δ 7.79 (dd, *J* = 9.0, 1.2 Hz, 2H), 7.56 (d, *J* = 8.4 Hz, 2H), 7.34 (dd, *J* = 7.2, 2.4 Hz, 1H), 7.34 – 7.29 (m, 3H), 7.30 – 7.24 (m, 1H), 7.21 (t, *J* = 7.8 Hz, 2H), 7.18 (d, *J* = 1.2 Hz, 1H), 7.17 – 7.08 (m, 7H), 5.94 (s, 1H), 5.83 (d, *J* = 2.4 Hz, 1H), 3.68 (s, 3H), 2.33 (s, 3H), 2.14 (s, 3H). **^{13}C NMR** (151 MHz, $CDCl_3$) δ 170.9, 165.9, 162.5, 157.7, 154.7, 150.8, 144.3, 137.9, 137.6, 134.6, 134.5, 133.4, 131.6, 130.8, 129.8, 129.8, 129.7, 129.1, 128.8, 127.7, 127.0, 126.3, 125.8, 125.7, 119.4, 101.5, 71.7, 71.6, 53.2, 52.5, 21.8, 18.3. **HRMS** (ESI-TOF) m/z: $[M+Na]^+$ Calcd for $C_{38}H_{30}FN_3NaO_6S^+$ 714.1436, Found 714.1436.

Methyl (5*R*,6*R*,11*R*,*E*)-11-(2-methoxyphenyl)-1-methyl-4-oxo-3,9-diphenyl-7-(tosylimino)-8-oxa-2,3-diazadispiro[4.0.4^6.3^5]trideca-1,9,12-triene-13-carboxylate (3ab)

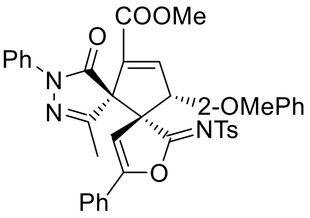

36.2 mg, 53% yield, white solid, m.p.: 212.5 – 212.6 ℃; **HPLC** (Daicel Chiralpak IE, n-hexane/2-propanol = 70:30, 1.0 mL/min, at 254 nm): t_R = 33.12 min (major), t_R = 47.54 min (minor); er = 92:8, $[\alpha]_D^{35}$ = + 64.133 (c = 0.015, DCM). **^{1}H NMR** (600 MHz, $CDCl_3$) δ 7.67 (dd, *J* = 8.4, 1.2 Hz, 2H), 7.44 (d, *J* = 8.4 Hz, 2H), 7.39 (d, *J* = 2.4 Hz, 1H), 7.32 (dd, *J* = 7.8, 1.8 Hz, 2H), 7.30 – 7.26 (m, 5H), 7.24 (td, *J* = 7.8, 1.8 Hz,1H), 7.10 (tt, *J* = 7.8, 1.2 Hz, 2H), 7.05 (d, *J* = 7.8 Hz, 2H), 6.85 (td, *J* = 7.8, 1.2 Hz, 1H), 6.75 (dd, *J* = 8.4, 1.2 Hz, 1H), 6.16 (s, 1H), 5.22 (d, *J* = 2.4 Hz, 1H), 3.68 (s, 3H), 3.56 (s, 3H), 2.26 (s, 3H), 1.93 (s, 3H). **^{13}C NMR** (151 MHz, $CDCl_3$) δ 171.1, 167.3, 162.5, 158.6, 157.4, 152.6, 150.1, 143.8, 138.0, 137.8, 134.3, 130.6, 130.4, 129.8, 129.4, 129.0, 128.9, 127.5, 126.9, 125.6, 125.4, 122.8, 120.5, 119.7, 110.4, 105.5, 71.6, 69.1, 55.3, 53.9, 52.6, 21.7, 18.5. **HRMS** (ESI-TOF) m/z: $[M+Na]^+$ Calcd for $C_{39}H_{33}N_3NaO_7S^+$ 710.1931, Found 710.1933.

Methyl (5*R*,6*R*,11*S*,*E*)-11-(2,4-dichlorophenyl)-1-methyl-4-oxo-3,9-diphenyl-7-(tosylimino)-8-oxa-2,3-diazadispiro[4.0.4^6.3^5]trideca-1,9,12-triene-13-carboxylate (3ac)

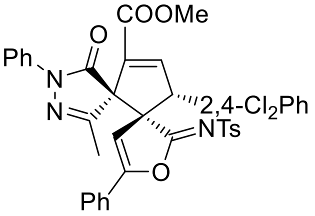

58.4 mg, 80% yield, white solid, m.p.: 162.4 ℃; **HPLC** (Daicel Chiralpak IF, n-hexane/2-propanol = 70:30, 1.0 mL/min, at 254 nm): t_R = 16.47 min (major), t_R = 35.01 min (minor); er = 96:4, $[\alpha]_D^{35}$ = + 589.048 (c = 0.020, DCM). **^{1}H NMR** (600 MHz, $CDCl_3$) δ 7.79 (dd, *J* = 8.4, 1.2 Hz, 2H), 7.61 (d, *J* = 8.4 Hz, 2H), 7.32 (td, *J* = 7.2, 1.2 Hz, 2H), 7.32 – 7.27 (m, 1H), 7.28 – 7.19 (m, 8H), 7.16 – 7.13 (m, 2H), 6.94 (dd, *J* = 8.4, 2.4 Hz, 1H), 5.91 (s, 1H), 5.77 (d, *J* = 2.4 Hz, 1H), 3.70 (s, 3H), 2.37 (s, 3H), 2.17 (s, 3H). **^{13}C NMR** (151 MHz, $CDCl_3$) δ 170.8, 166.0, 162.4, 157.6, 155.0, 150.1, 144.8, 137.5, 137.5, 135.1, 135.0, 134.0, 131.0, 130.2, 129.8, 129.5, 129.1, 129.0, 127.9, 127.2, 126.2, 125.9, 125.8, 119.5, 101.2, 71.7, 71.7, 52.6, 52.6, 21.9, 18.4. **HRMS** (ESI-TOF) m/z: $[M+Na]^+$ Calcd for $C_{38}H_{29}Cl_2N_3NaO_6S^+$ 748.1046, Found 748.1046.

Methyl (1*R*,5*S*,5'*R*,*E*)-3''-methyl-5''-oxo-1'',5,5'-triphenyl-3-(tosylimino)-1'',5''-dihydro-4,6-dioxadispiro[bicyclo[3.1.0]hexane-2,1'-cyclopentane-2',4''-pyrazol]-3'-ene-3'-carboxylate (4)

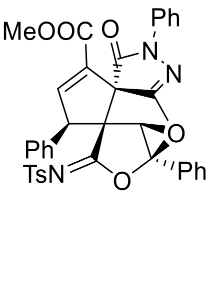

57.6 mg, 86% yield, white solid, m.p.: 208.0 °C; **HPLC** (Daicel Chiralpak IF, n-hexane/2-propanol = 70:30, 1.0 mL/min, at 254 nm): t_R = 20.88 min (major), t_R = 36.32 min (minor); er = 93:7, $[\alpha]_D^{35}$ = + 426.833 (c = 0.015, DCM). **^{1}H NMR** (600 MHz, $CDCl_3$) δ 7.89 (d, *J* = 7.8 Hz, 2H), 7.47 (d, *J* = 8.4 Hz, 2H), 7.38 – 7.35 (m, 3H), 7.31 (dt, *J* = 20.4, 7.2 Hz, 2H), 7.20 (dd, *J* = 15.6, 8.4 Hz, 4H), 7.16 (d, *J* = 7.2 Hz, 1H), 7.11 (d, *J* = 7.8 Hz, 2H), 6.97 (d, *J* = 7.2 Hz, 2H), 6.76 (d, *J* = 7.8 Hz, 2H), 5.22 (d, *J* = 2.4 Hz, 1H), 4.27 (s, 1H), 3.68 (s, 3H), 2.34 (s, 3H), 2.14 (s, 3H). **^{13}C NMR** (151 MHz, $CDCl_3$) δ 171.0, 164.3, 162.3, 159.0, 148.5, 144.2, 137.8, 137.6, 135.7, 133.6, 130.8, 129.5, 129.2, 129.2, 129.1, 128.7, 128.6, 128.2, 127.5, 126.3, 125.9, 119.7, 90.8, 69.7, 68.3, 64.5, 55.2, 52.5, 21.8, 19.0. **HRMS** (ESI-TOF) m/z: $[M+Na]^+$ Calcd for $C_{38}H_{31}N_3NaO_7S^+$ 696.1775, Found 696.1778.

Methyl (5*S*,6*S*,13*R*,*E*)-1-methyl-4-oxo-3,9,11-triphenyl-7-(tosylimino)-8-oxa-2,3-diazadispiro[4.0.4^6.3^5]trideca-1,9,11-triene-13-carboxylate (3a')

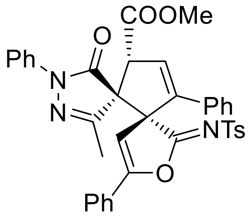

38.8 mg, 78% yield, white solid, m.p.: 226.2 ℃; **HPLC** (Daicel Chiralpak IE, n-hexane/2-propanol = 70:30, 1.0 mL/min, at 254 nm): t_R = 32.81 min (major), t_R = 45.21 min (minor); er = 94:6, $[\alpha]_D^{35}$ = + 159.074 (c = 0.015, DCM). **^{1}H NMR** (700 MHz, $CDCl_3$) δ 7.83 (d, *J* = 8.4 Hz, 2H), 7.54 (dd, *J* = 8.4, 1.4 Hz, 2H), 7.35 (d, *J* = 2.1 Hz, 1H), 7.25 (dd, *J* = 8.4, 7.7 Hz, 2H), 7.21 (t, *J* = 7.7 Hz, 1H), 7.19 – 7.14 (m, 4H), 7.12 (t, *J* = 7.7 Hz, 1H), 7.12 – 7.05 (m, 5H), 7.02 (d, *J* = 7.7 Hz, 2H), 5.92 (s, 1H), 4.85 (d, *J* = 2.1 Hz, 1H), 3.67 (s, 3H), 2.27 (s, 3H), 2.23 (s, 3H). **^{13}C NMR** (176 MHz, $CDCl_3$) δ 170.3, 170.1, 162.2, 157.9, 151.9, 148.5, 144.4, 137.8, 137.5, 135.0, 134.9, 130.4, 129.6, 129.2, 128.9, 128.7, 128.7, 127.8, 126.6, 125.6, 125.5, 119.7, 104.5, 71.5, 71.3, 61.6, 52.8, 21.8, 17.6. **HRMS** (ESI-TOF) m/z: $[M+Na]^+$ Calcd for $C_{38}H_{31}N_3NaO_6S^+$ 680.1826, Found 680.1827.

Methyl (5*S*,6*S*,13*S*,*E*)-1-methyl-4-oxo-3,9,11-triphenyl-7-(tosylimino)-8-oxa-2,3-diazadispiro[4.0.4^6.3^5]trideca-1,9,11-triene-13-carboxylate (5)

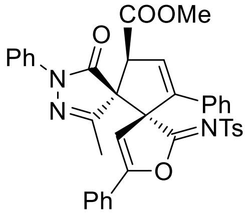

24.9 mg, 83% yield, white solid, m.p.: 192.6 – 194.0 °C; **HPLC** (Daicel Chiralpak IF, n-hexane/2-propanol = 70:30, 1.0 mL/min, at 254 nm): t_R = 22.97 min (major), t_R = 51.40 min (minor); er = 94:6, $[\alpha]_D^{35}$ = + 807.778 (c = 0.015, DCM). **^{1}H NMR** (700 MHz, $CDCl_3$) δ 7.79 (d, *J* = 8.4 Hz, 2H), 7.70 (dd, *J* = 8.4, 1.4 Hz, 2H), 7.36 – 7.30 (m, 3H), 7.31 – 7.26 (m, 4H), 7.22 (dd, *J* = 9.1, 0.7 Hz, 2H), 7.15 (tt, *J* = 8.4, 1.4 Hz, 1H), 7.11 (tt, *J* = 7.7, 1.4 Hz, 1H), 7.08 (t, *J* = 7.7 Hz, 2H), 6.91 (dd, *J* = 8.4, 1.4 Hz, 2H), 6.49 (d, *J* = 2.1 Hz, 1H), 5.64 (s, 1H), 4.56 (d, *J* = 2.1 Hz, 1H), 3.60 (s, 3H), 2.36 (s, 3H), 2.27 (s, 3H). **^{13}C NMR** (176 MHz, $CDCl_3$) δ 170.7, 169.1, 168.4, 155.3, 155.2, 143.4, 141.8, 136.4, 136.1, 132.3, 129.9, 128.5, 128.2, 127.9, 127.8, 127.5, 127.4, 126.6, 125.3, 125.0, 124.6, 124.5, 118.2, 100.2, 70.4,

67.2, 51.9, 51.7, 20.6, 15.5. **HRMS** (ESI-TOF) m/z: $[M+Na]^+$ Calcd for $C_{38}H_{31}N_3NaO_6S^+$ 680.1826, Found 680.1824.

Methyl (5*R*,6*R*,11*R*,*E*)-3-(2-acetoxyphenyl)-1-methyl-4-oxo-9,11-diphenyl-7-(tosylimino)-8-oxa-2,3-diazadispiro[4.0.4^6.3^5]trideca-1,9,12-triene-13-carboxylate (6)

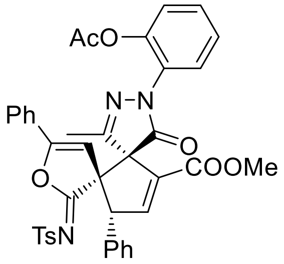

29.9 mg, 91% yield, white solid, m.p.: 174.8 ℃; **HPLC** (Daicel Chiralpak IF, n-hexane/2-propanol = 70:30, 1.0 mL/min, at 254 nm): t_R = 38.69 min (major), t_R = 72.40 min (minor); er = 94:6, $[\alpha]_D^{35}$ = + 842.857 (c = 0.015, DCM). **^{1}H NMR** (600 MHz, $CDCl_3$) δ 7.50 (d, *J* = 1.8 Hz, 1H), 7.41 – 7.39 (m, 3H), 7.34 (t, *J* = 7.2 Hz, 1H), 7.31 – 7.29 (m, 1H), 7.28 – 7.26 (m, 2H), 7.23 (td, *J* = 8.4, 1.2 Hz, 3H), 7.19 – 7.16 (m, 1H), 7.15 – 7.11 (m, 5H), 7.02 (d, *J* = 7.2 Hz, 2H), 5.97 (s, 1H), 5.23 (d, *J* = 1.8 Hz, 1H), 3.73 (s, 3H), 2.34 (s, 3H), 2.14 (s, 3H), 1.86 (s, 3H). **^{13}C NMR** (151 MHz, $CDCl_3$) δ 171.5, 168.9, 165.9, 162.6, 158.2, 156.0, 150.6, 144.8, 144.1, 137.9, 134.2, 133.8, 131.1, 129.6, 129.2, 129.0, 128.9, 128.8, 128.1, 127.6, 126.5, 126.4, 126.2, 125.8, 124.5, 100.4, 71.1, 70.5, 55.9, 52.5, 21.8, 20.8, 18.1. **HRMS** (ESI-TOF) m/z: $[M+Na]^+$ Calcd for $C_{40}H_{33}N_3NaO_8S^+$ 738.1881, Found 738.1881.

Diethyl 2-(2-((5*R*,6*R*,11*R*,*E*)-13-(methoxycarbonyl)-4-methyl-1-oxo-9,11-diphenyl-7-(tosylimino)-8-oxa-2,3-diazadispiro[4.0.4^6.3^5]trideca-3,9,12-trien-2-yl)phenyl)malonate (7)

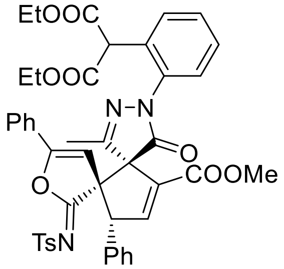

39.6 mg, 97% yield, white solid, m.p.: 92.4 – 115.2 ℃; **HPLC** (Daicel Chiralpak IF, n-hexane/2-propanol = 70:30, 1.0 mL/min, at 254 nm): t_R = 32.04 min (major), t_R = 36.47 min (minor); er = 94:6, $[\alpha]_D^{35}$ = + 618.125 (c = 0.015, DCM). **^{1}H NMR** (700 MHz, $CDCl_3$) δ 7.57 – 7.54 (m, 1H), 7.48 (d, *J* = 2.1 Hz, 1H), 7.39 (d, *J* = 8.4 Hz, 2H), 7.38 – 7.31 (m, 3H), 7.29 – 7.24 (m, 5H), 7.22 – 7.17 (m, 1H), 7.14 (dd, *J* = 9.8, 7.7 Hz, 4H), 7.04 (d, *J* = 7.0 Hz, 2H), 6.25 (s, 1H), 5.20 (d, *J* = 2.1 Hz, 1H), 4.70 (s, 1H), 4.22 (dq, *J* = 11.2, 7.0 Hz, 1H), 4.16 (dq, *J* = 11.2, 7.0 Hz, 1H), 4.01 （ddq, J = 32.9, 11.2, 7.0 Hz, 2H) , 3.75 (s, 3H), 2.34 (s, 3H), 2.16 (s, 3H), 1.22 (t, *J* = 7.0 Hz, 3H), 1.01 (t, *J* = 7.0 Hz, 3H). **^{13}C NMR** (176 MHz, $CDCl_3$) δ 172.7, 168.1, 167.9, 165.9, 162.6, 158.5, 156.3, 150.6, 144.1, 137.9, 135.6, 134.2, 133.8, 131.0, 130.5, 130.2, 129.6, 129.2, 129.2, 129.2, 128.9, 128.9, 128.1, 127.7, 126.6, 126.2, 125.7, 100.4, 70.7, 70.5, 62.2, 62.0, 56.2, 53.3, 52.5, 21.8, 18.3, 14.3, 14.1. **HRMS** (ESI-TOF) m/z: $[M+Na]^+$ Calcd for $C_{45}H_{41}N_3NaO_{10}S^+$ 838.2405, Found 838.2410.

Methyl 2-(1-((tert-butoxycarbonyl)oxy)-2-oxo-1,2-dihydroacenaphthylen-1-yl)acrylate (2b)

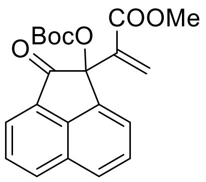

332.6 mg, 90% yield, red solid, m.p.: 187.0 ℃. **^{1}H NMR** (600 MHz, $CDCl_3$) δ 8.01 (dd, *J* = 8.4, 0.6 Hz, 1H), 7.96 (dd, *J* = 7.2, 0.6 Hz, 1H), 7.83 (dd, *J* = 8.4, 0.6 Hz, 1H), 7.68 (dd, *J* = 8.4, 7.2 Hz, 1H), 7.53 (dd, *J* = 8.4, 6.6 Hz, 1H), 7.43 (dd, *J* = 6.6, 0.6 Hz, 1H), 6.56 (d, *J* = 1.2 Hz, 1H), 6.52 (d, *J* = 0.6 Hz, 1H), 3.28 (s, 3H), 1.13 (s, 9H). **^{13}C NMR** (151 MHz, $CDCl_3$) δ 198.4, 164.7, 150.6, 142.3, 138.0, 136.6, 133.8, 131.0, 130.4, 128.4, 128.3, 128.3, 126.7, 121.5, 120.6, 83.7, 52.1, 27.6. **HRMS** (ESI-TOF) m/z: $[M+Na]^+$ Calcd for $C_{21}H_{20}NaO_6^+$ 391.1152, Found 391.1136.

Methyl (1*R*,1'*R*,5'*R*,*E*)-2-oxo-5',5''-diphenyl-2''-(tosylimino)-2*H*,2''*H*-dispiro[acenaphthylene-1,2'-cyclopentane-1',3''-furan]-3'-ene-3'-carboxylate (3ba)

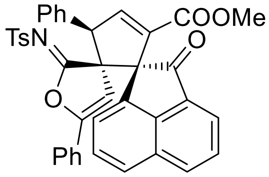

29.1 mg, 45% yield, pink solid, m.p.: 250.0 – 250.1 ℃; **HPLC** (Daicel Chiralpak IF, n-hexane/2-propanol = 70:30, 1.0 mL/min, at 254 nm): t_R = 42.81 min (major), t_R = 63.54 min (minor); er = 86:14, $[\alpha]_D^{35}$ = + 180.769 (c = 0.015, DCM). **^{1}H NMR** (600 MHz, $CDCl_3$) δ 7.99 (d, *J* = 7.8 Hz, 1H), 7.95 (d, *J* = 7.2 Hz, 1H), 7.92 (d, *J* = 7.2 Hz, 1H), 7.74 (d, *J* = 8.4 Hz, 1H), 7.62 (dd, *J* = 8.4, 7.2 Hz, 1H), 7.52 (dd, *J* = 8.4, 7.2 Hz, 1H), 7.47 (d, *J* = 2.4 Hz, 1H), 7.41 (d, *J* = 8.4 Hz, 2H), 7.20 – 7.17 (m, 1H), 7.18 – 7.08 (m, 7H), 7.05 (t, *J* = 7.8 Hz, 2H), 6.79 (d, *J* = 7.2 Hz, 2H), 5.77 (s, 1H), 5.17 (d, *J* = 1.8 Hz, 1H), 3.41 (s, 3H), 2.32 (s, 3H). **^{13}C NMR** (151 MHz, $CDCl_3$) δ 202.6, 167.3, 163.5, 154.9, 147.9, 143.7, 143.6, 138.4, 137.8, 135.2, 135.1, 132.9, 131.2, 130.8, 130.4, 129.5, 129.3, 129.3, 128.8, 128.6, 128.0, 127.9, 127.7, 126.3, 125.8, 125.3, 125.0, 122.6, 101.4, 72.9, 71.1, 56.8, 51.9, 21.7. **HRMS** (ESI-TOF) m/z: $[M+Na]^+$ Calcd for $C_{40}H_{29}NNaO_6S^+$ 674.1608, Found 674.1608.
