# Supplementary material for: Silver/chiral pyrrolidinopyridine relay catalytic cycloisomerization/(2 + 3) cycloadditions of enynamides to asymmetrically synthesize bispirocyclopentenes as PDE1B inhibitors
Source: Commun Chem. 2023 Jun 19;6:128. doi: 10.1038/s42004-023-00921-6 (PMC10279699; doi:10.1038/s42004-023-00921-6)
Supplement: Supplementary file 5 — Supplementary Data 2 [file 42004_2023_921_MOESM5_ESM.pdf]

Supplementary Data 2

NMR and HPLC spectra

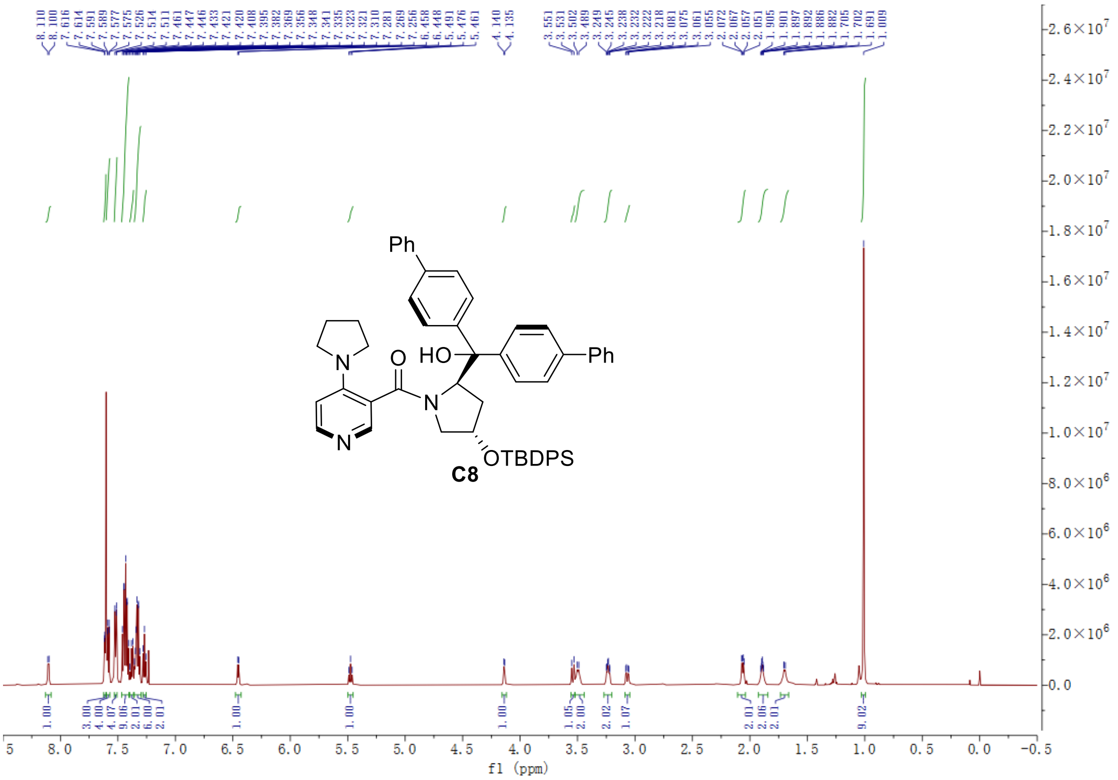

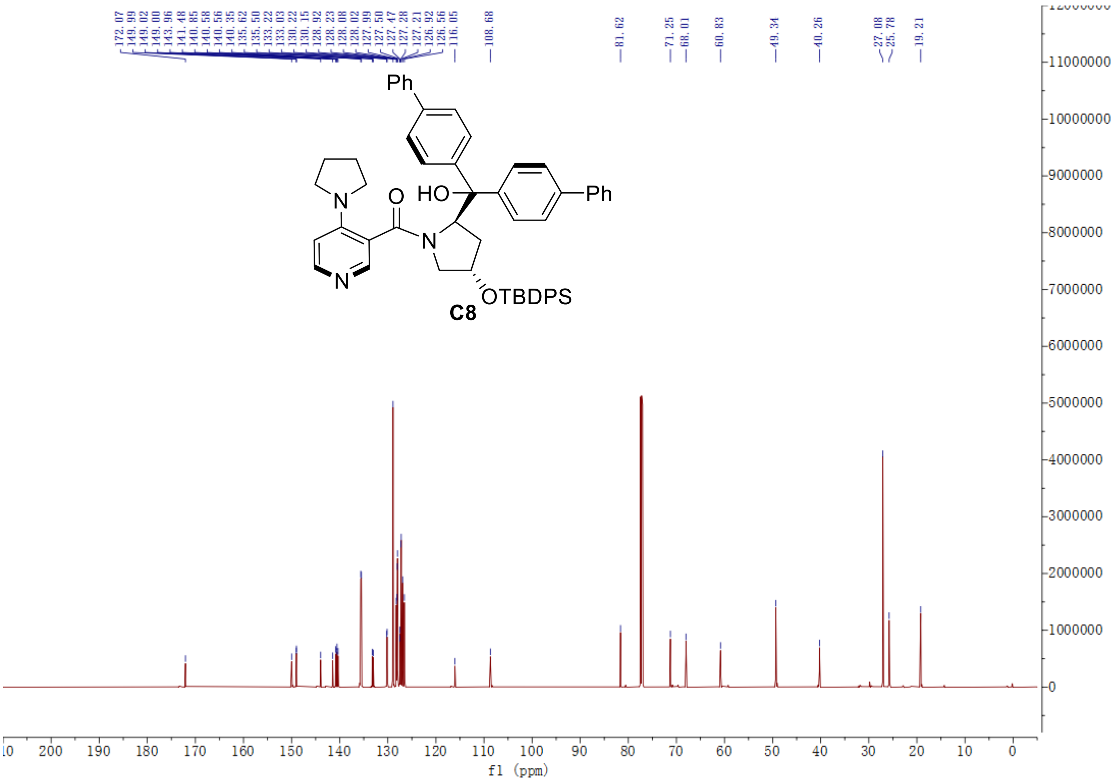

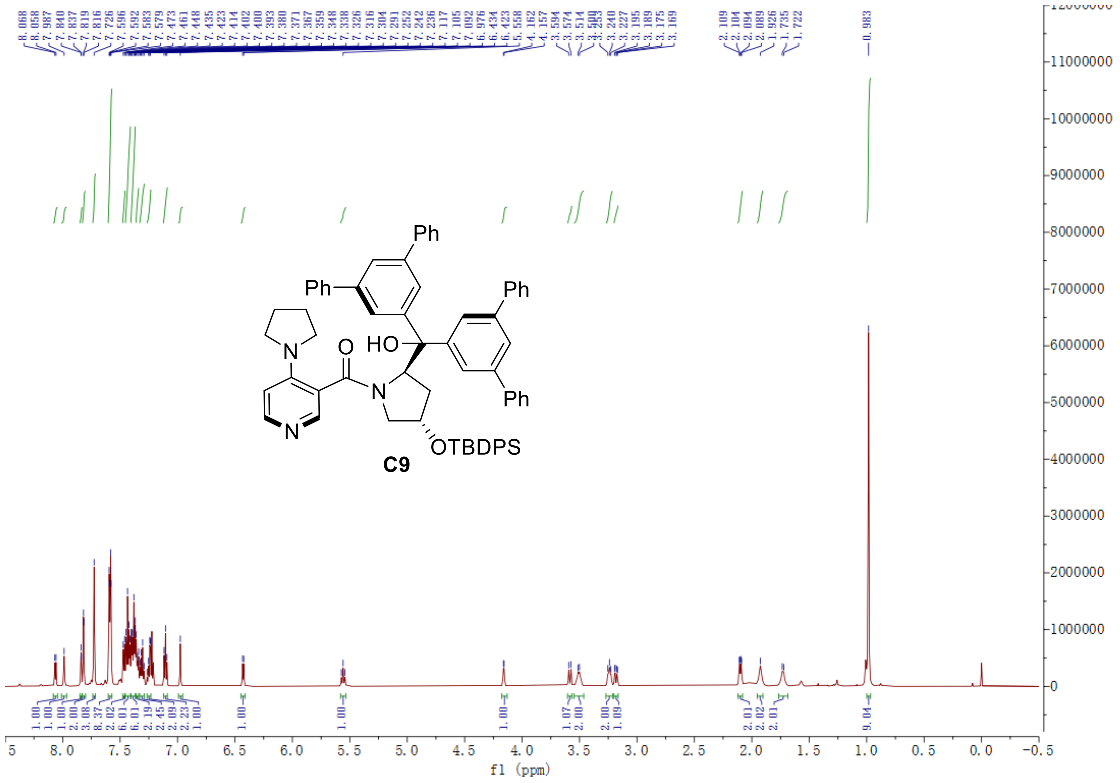

C9
Ph
HO
O
N
OTBDPS
f1 (ppm)

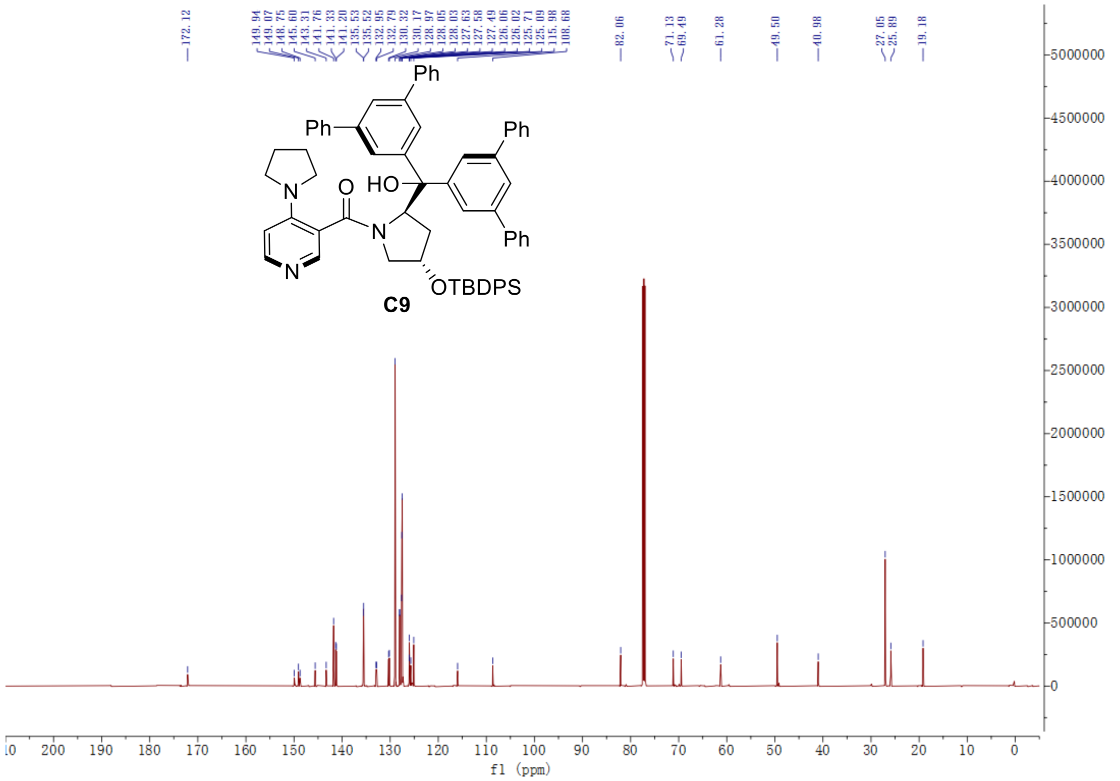

172.12
149.94
149.07
148.75
145.60
143.31
141.76
141.33
141.20
135.53
135.52
132.95
132.79
130.32
130.17
128.97
128.05
128.03
127.63
127.58
127.49
127.06
126.02
125.71
125.09
115.98
108.68
82.06
71.13
69.49
61.28
49.50
40.98
27.05
25.89
19.18
C9
Ph
HO
O
N
OTBDPS
f1 (ppm)

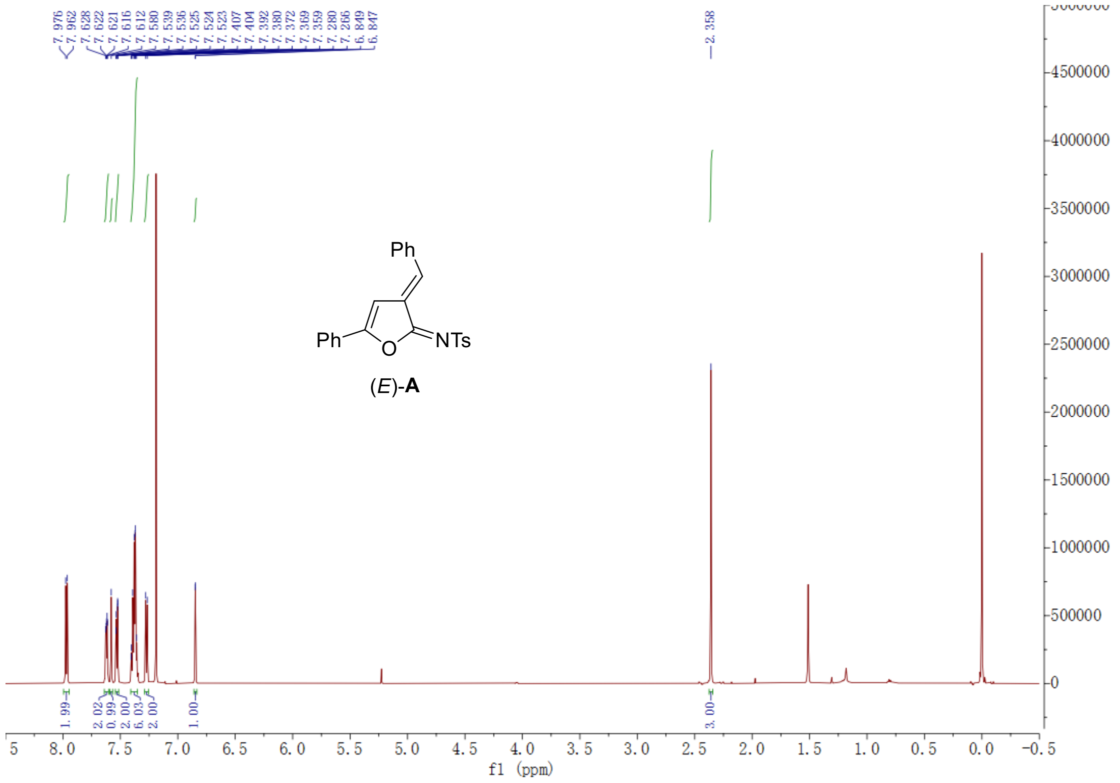
7.976
7.962
7.628
7.622
7.621
7.616
7.612
7.580
7.539
7.536
7.525
7.524
7.523
7.407
7.404
7.392
7.380
7.372
7.369
7.359
7.280
7.266
6.849
6.847
2.358
Ph
Ph
O
NTs
(E)-A
1.99
2.02
0.99
0.99
2.00
6.03
2.00
1.00
3.00
f1 (ppm)

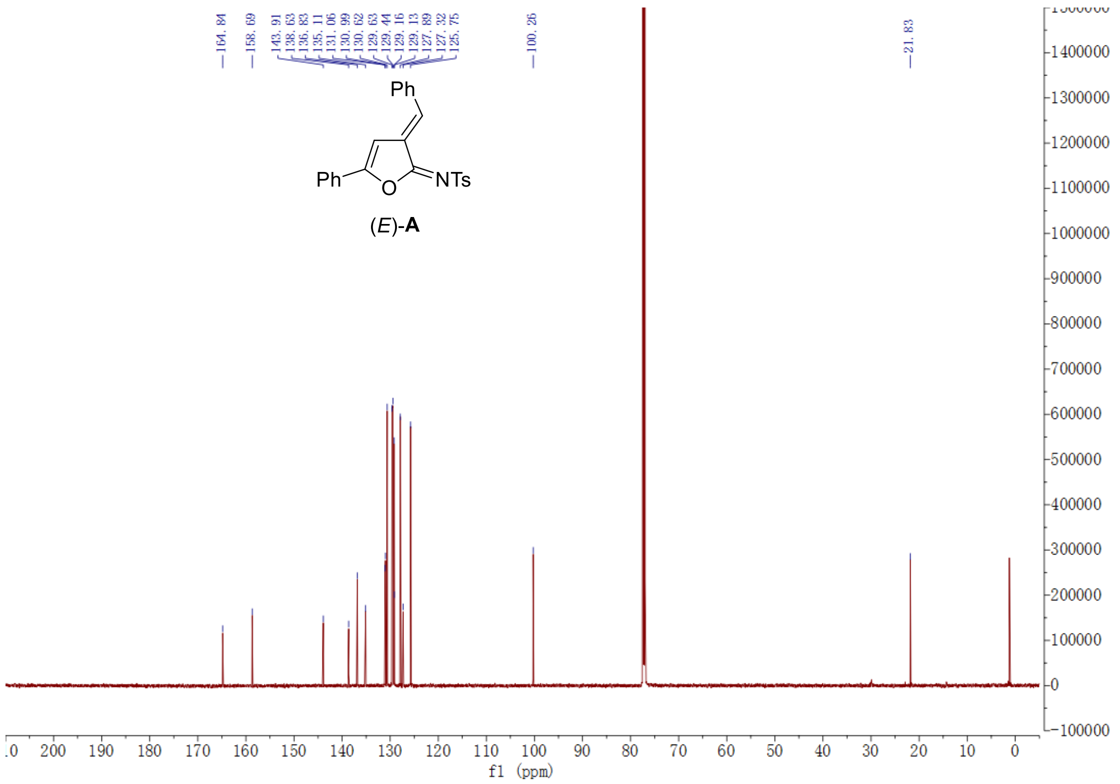
164.84
158.69
143.91
138.63
136.83
135.11
131.06
130.99
130.62
129.63
129.44
129.16
129.13
127.89
127.32
125.75
100.26
21.83
Ph
Ph
O
NTs
(E)-A
f1 (ppm)

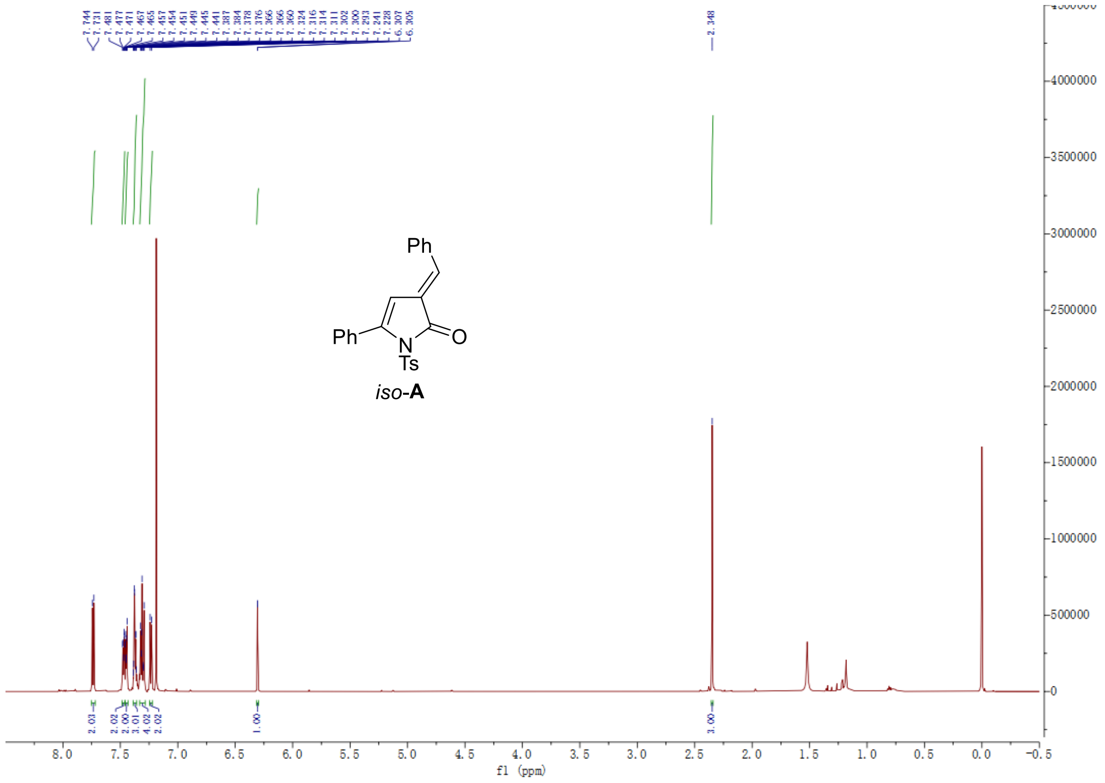
Ph
Ph
N
O
Ts
iso-A
f1 (ppm)

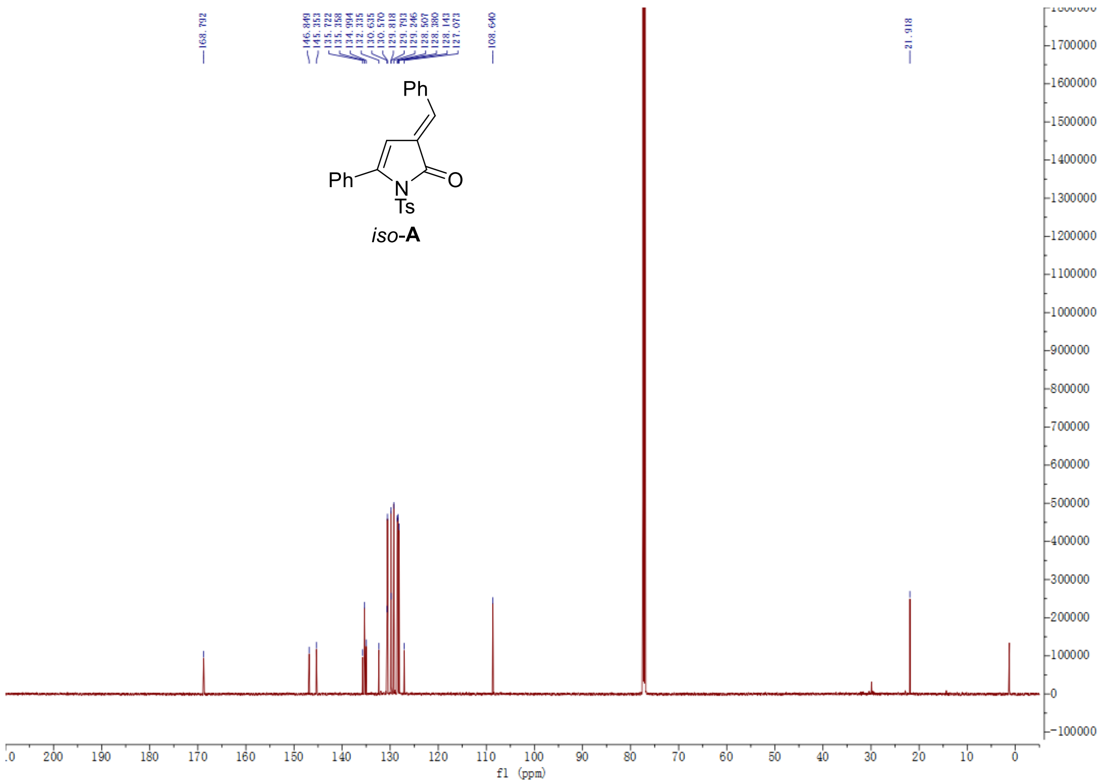
Ph
Ph
N
O
Ts
iso-A
f1 (ppm)

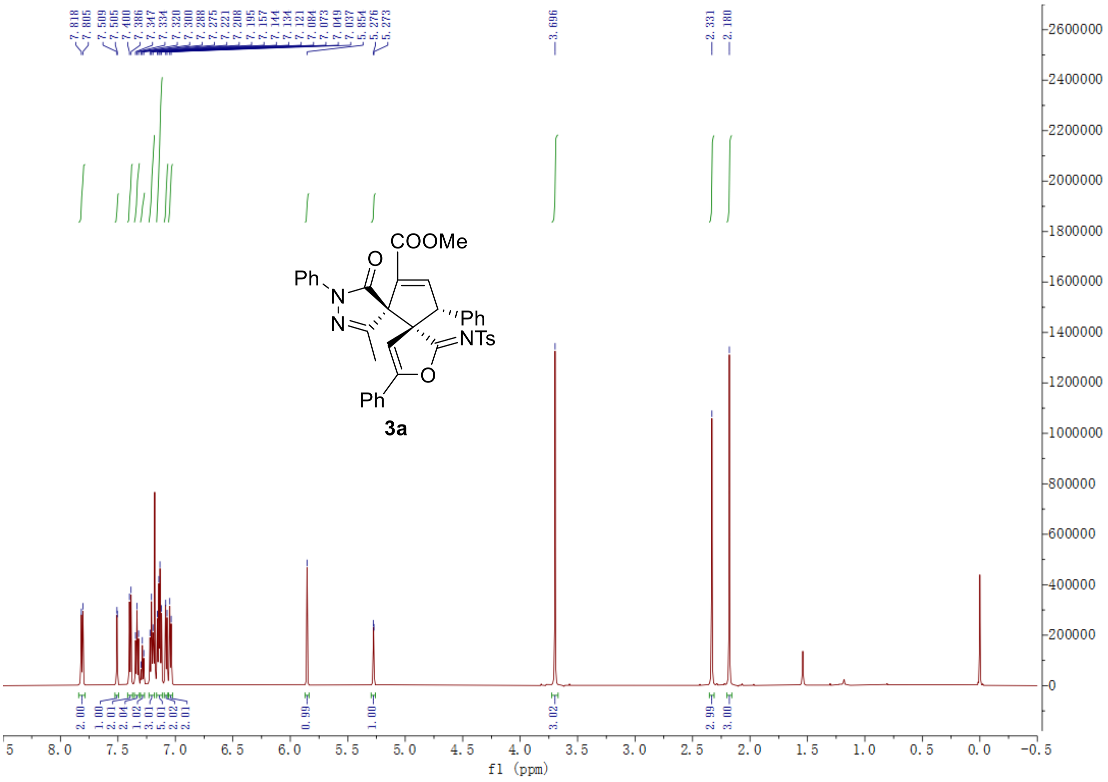

7.818
7.805
7.509
7.505
7.400
7.386
7.347
7.334
7.320
7.300
7.288
7.275
7.221
7.208
7.195
7.157
7.144
7.134
7.121
7.084
7.073
7.049
7.037
5.854
5.276
5.273
3.696
2.331
2.180
COOMe
O
Ph
N
N
Ph
NTs
O
Ph
3a
2.00
1.00
2.01
2.04
1.02
3.01
5.01
2.02
2.01
0.99
1.00
3.02
2.99
3.00
f1 (ppm)

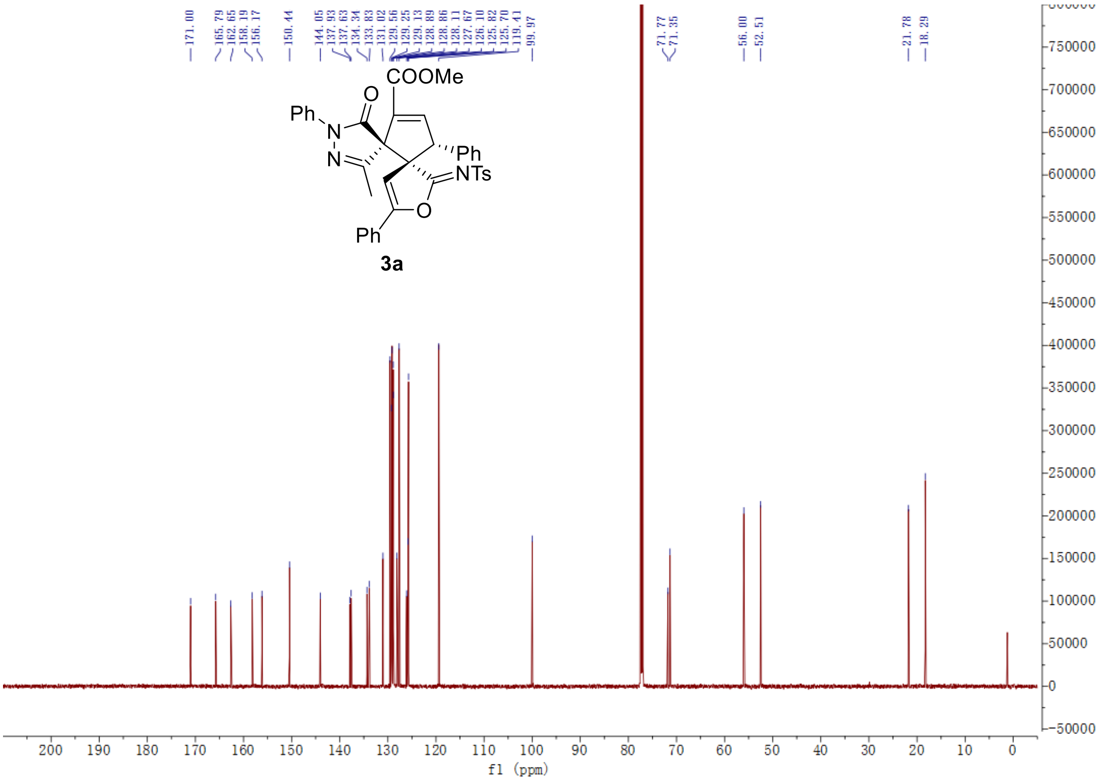

171.00
165.79
162.65
158.19
156.17
150.44
144.05
137.93
137.63
134.34
133.83
131.02
129.56
129.25
129.13
128.86
128.89
128.11
127.67
126.10
125.82
125.70
119.41
99.97
71.77
71.35
56.00
52.51
21.78
18.29
COOMe
O
Ph
N
N
Ph
NTs
O
Ph
3a
f1 (ppm)

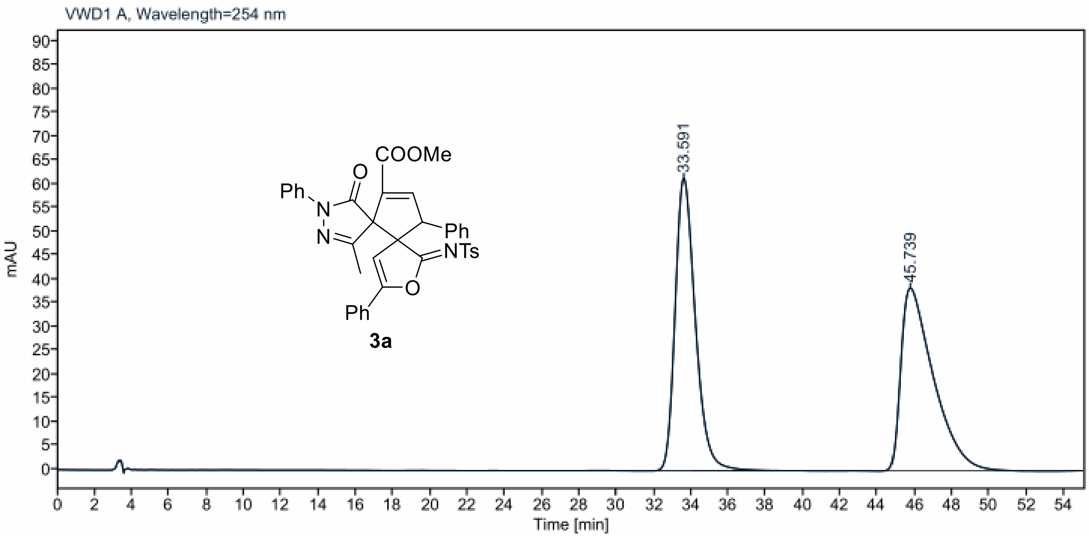

Signal: VWD1 A, Wavelength=254 nm

RT [min]	Type	Width [min]	Area	Height	Area%	Name
33.591	BB	1.1552	4653.1636	61.5384	49.8299	
45.739	BB	1.7470	4684.9395	38.4922	50.1701	
		Sum	9338.1030			

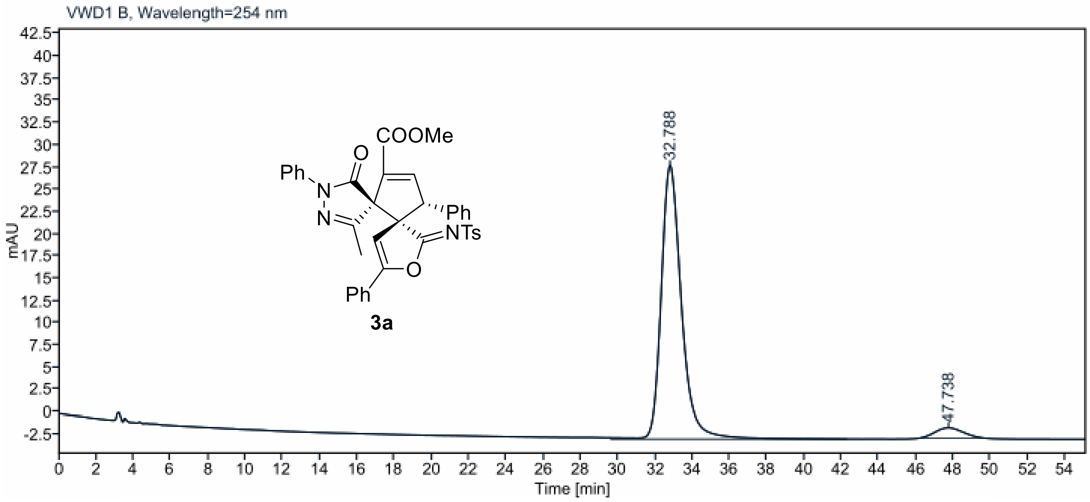

Signal: VWD1 B, Wavelength=254 nm

RT [min]	Type	Width [min]	Area	Height	Area%	Name
32.788	MM	1.2698	2342.4099	30.7456	94.6693	
47.738	MM	1.8285	131.8988	1.2023	5.3307	
		Sum	2474.3087			

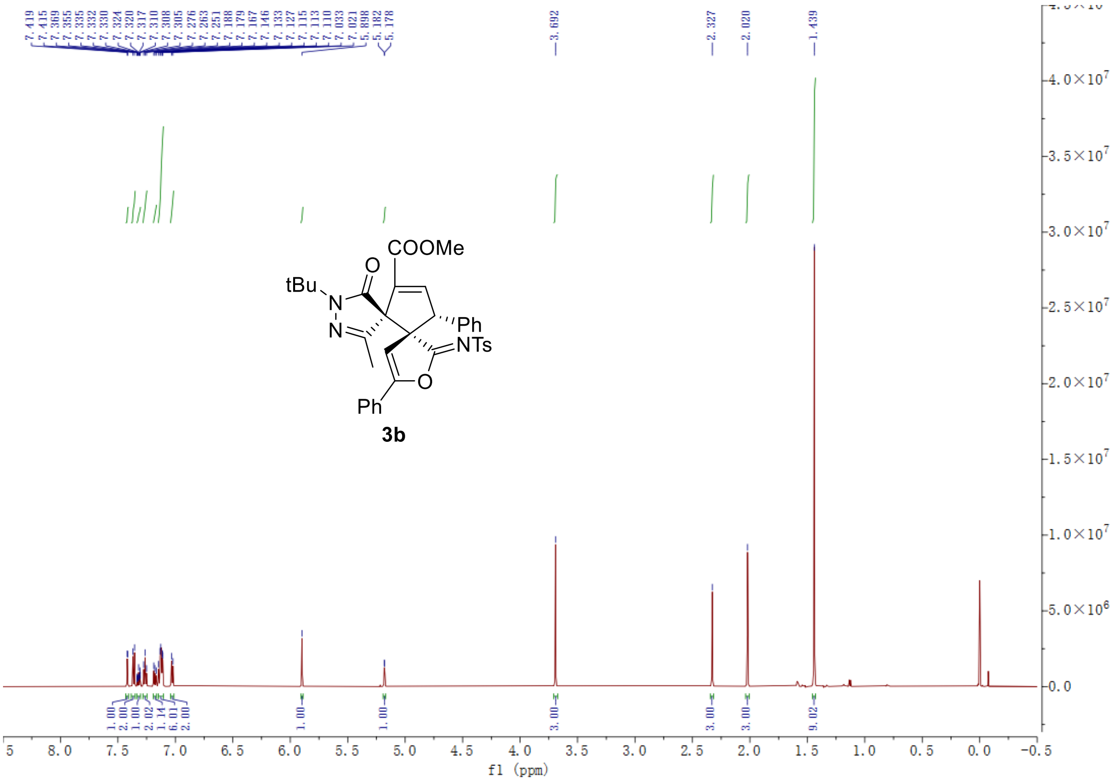
7.419 7.415 7.369 7.355 7.335 7.332 7.330 7.324 7.320 7.317 7.310 7.308 7.305 7.276 7.263 7.251 7.188 7.179 7.167 7.146 7.133 7.127 7.115 7.113 7.110 7.033 7.021 5.898 5.182 5.178
3.692
2.327
2.020
1.439
COOMe
tBu
O
N
N
Ph
NTs
O
Ph
3b
1.00 2.00 1.00 1.00 2.02 1.14 6.01 2.00
1.00
1.00
3.00
3.00
3.00
9.02
f1 (ppm)

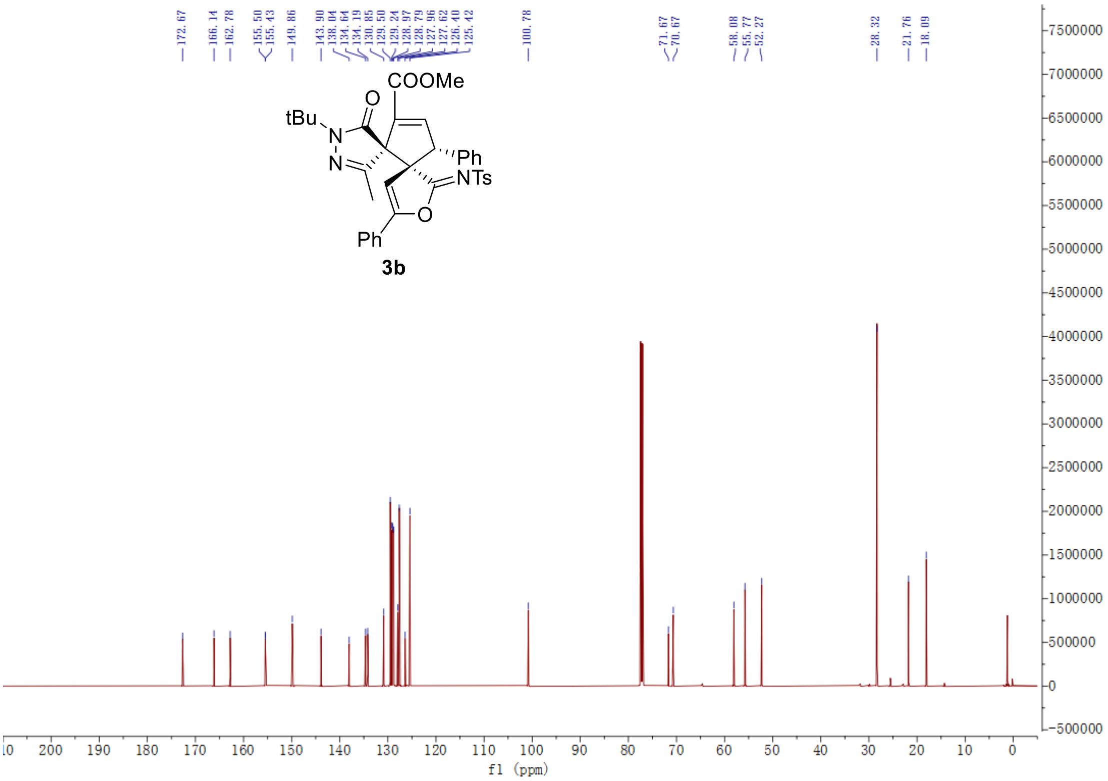
172.67
166.14
162.78
155.50 155.43
149.86
143.90 138.04 134.64 134.19 130.85 129.50 129.24 128.97 128.79 127.96 127.62 126.40 125.42
100.78
71.67 70.67
58.08 55.77 52.27
28.32
21.76
18.09
COOMe
tBu
O
N
N
Ph
NTs
O
Ph
3b
f1 (ppm)

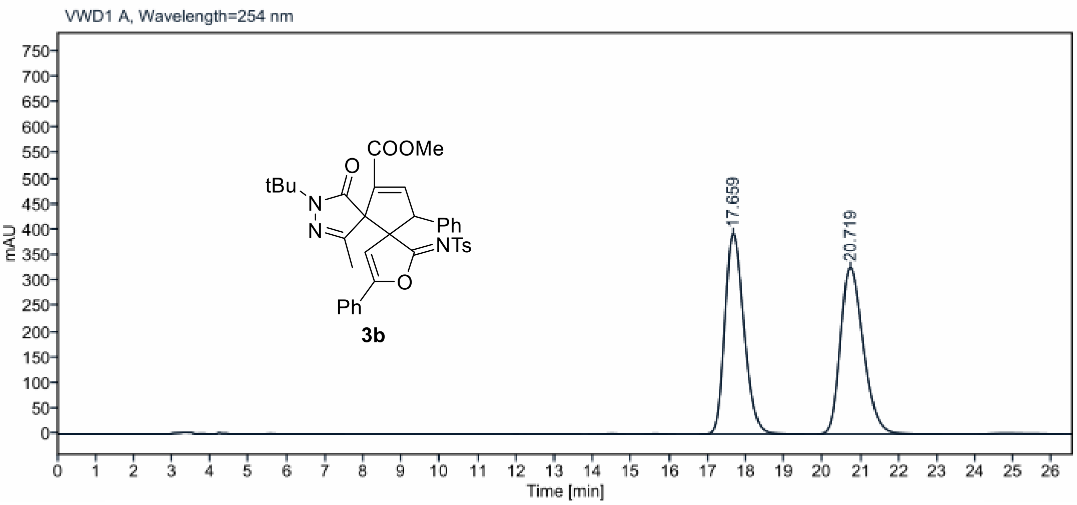

Signal: VWD1 A, Wavelength=254 nm

RT [min]	Type	Width [min]	Area	Height	Area%	Name
17.659	MM	0.5760	13586.3936	393.1182	50.0217	
20.719	MM	0.6918	13574.5996	327.0233	49.9783	
		Sum	27160.9932			

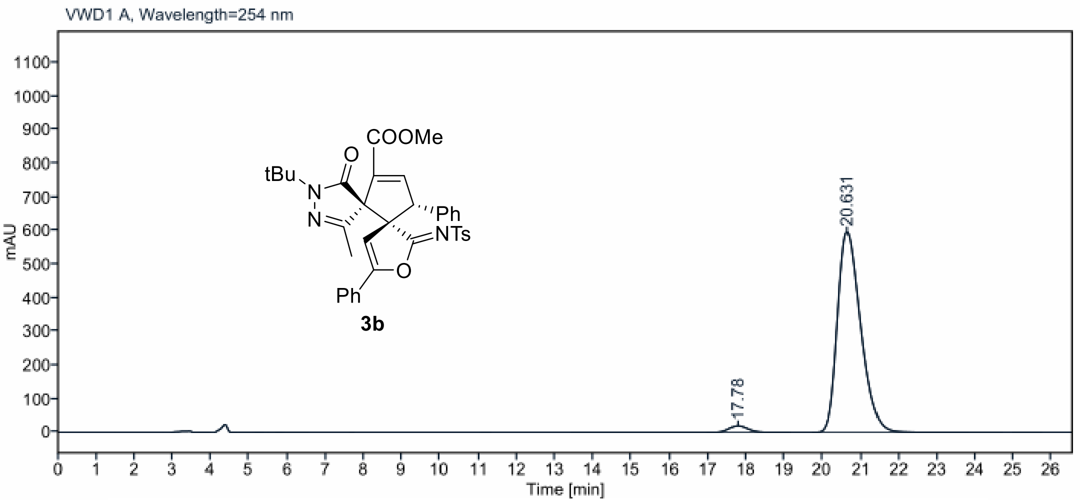

Signal: VWD1 A, Wavelength=254 nm

RT [min]	Type	Width [min]	Area	Height	Area%	Name
17.780	MM	0.5949	715.6263	20.0494	2.7515	
20.631	MM	0.7062	25292.8320	596.8898	97.2485	
		Sum	26008.4583			

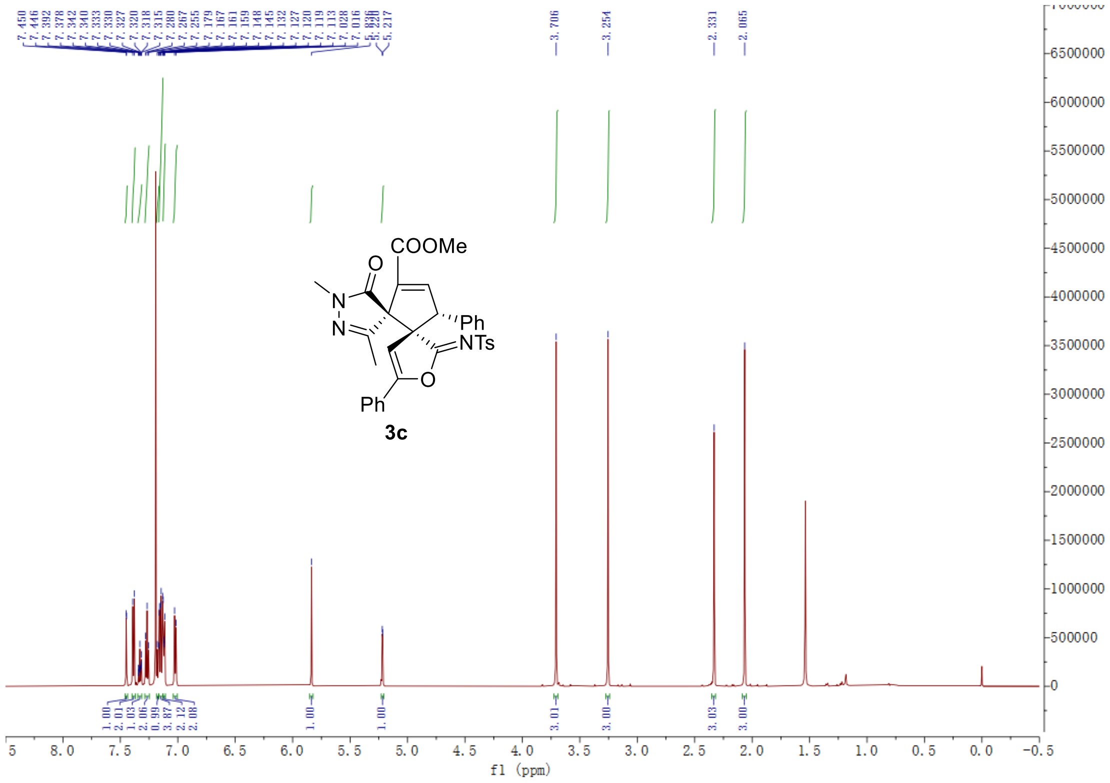
7.450
7.446
7.392
7.378
7.342
7.340
7.333
7.330
7.327
7.320
7.318
7.315
7.280
7.267
7.255
7.179
7.167
7.161
7.159
7.148
7.145
7.132
7.127
7.120
7.119
7.113
7.028
7.016
5.217
3.706
3.254
2.331
2.065
COOMe
O
N
N
Ph
NTs
O
Ph
3c
1.00
2.01
1.03
2.06
0.99
3.87
2.12
2.08
1.00
1.00
3.01
3.00
3.03
3.00
f1 (ppm)

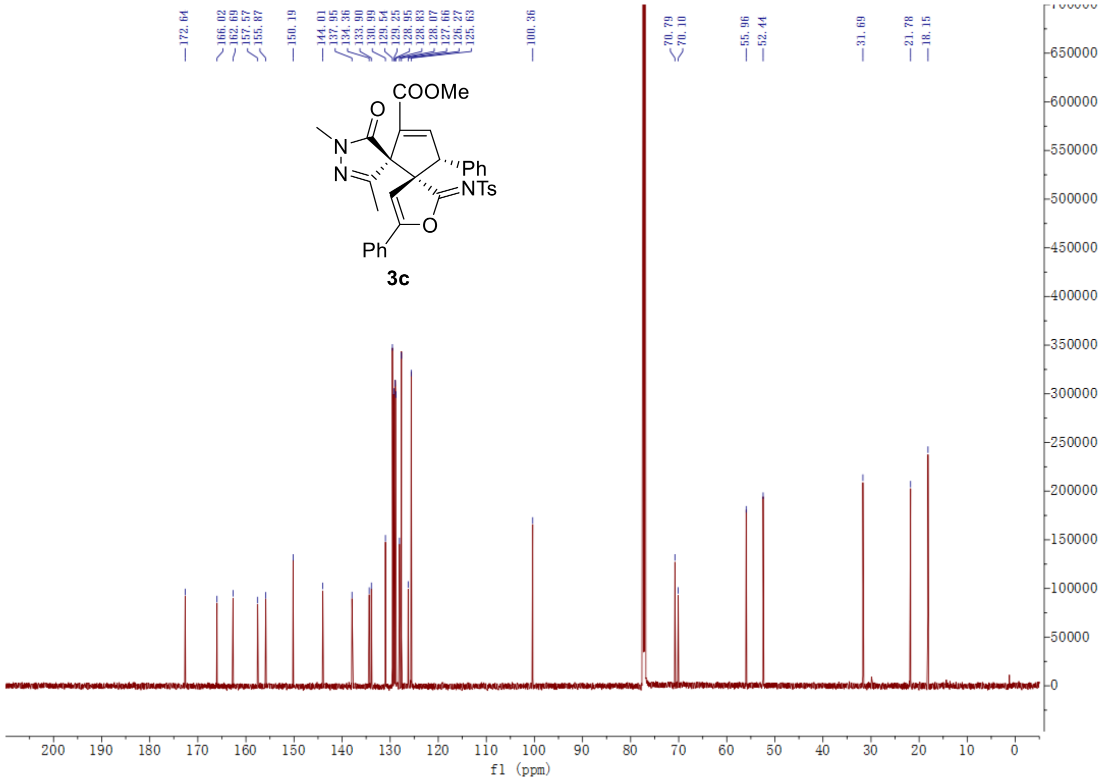
172.64
166.02
162.69
157.57
155.87
150.19
144.01
137.95
134.36
133.90
130.99
129.54
129.25
128.95
128.83
128.07
127.66
126.27
125.63
100.36
70.79
70.10
55.96
52.44
31.69
21.78
18.15
COOMe
O
N
N
Ph
NTs
O
Ph
3c
f1 (ppm)

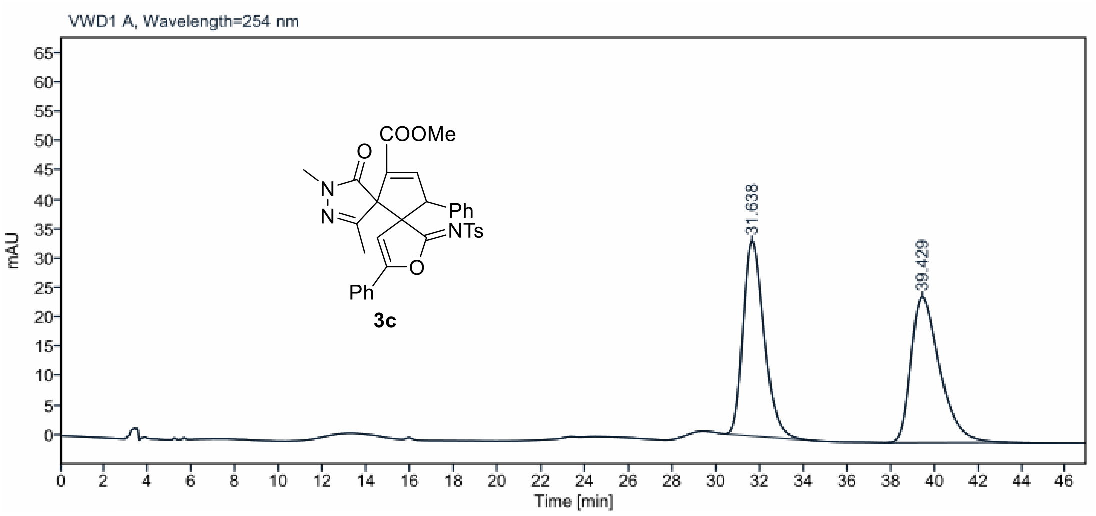

Signal: VWD1 A, Wavelength=254 nm

RT [min]	Type	Width [min]	Area	Height	Area%	Name
31.638	BB	1.0358	2241.4316	33.1956	49.5621	
39.429	MM	1.5335	2281.0391	24.7909	50.4379	
		Sum	4522.4707			

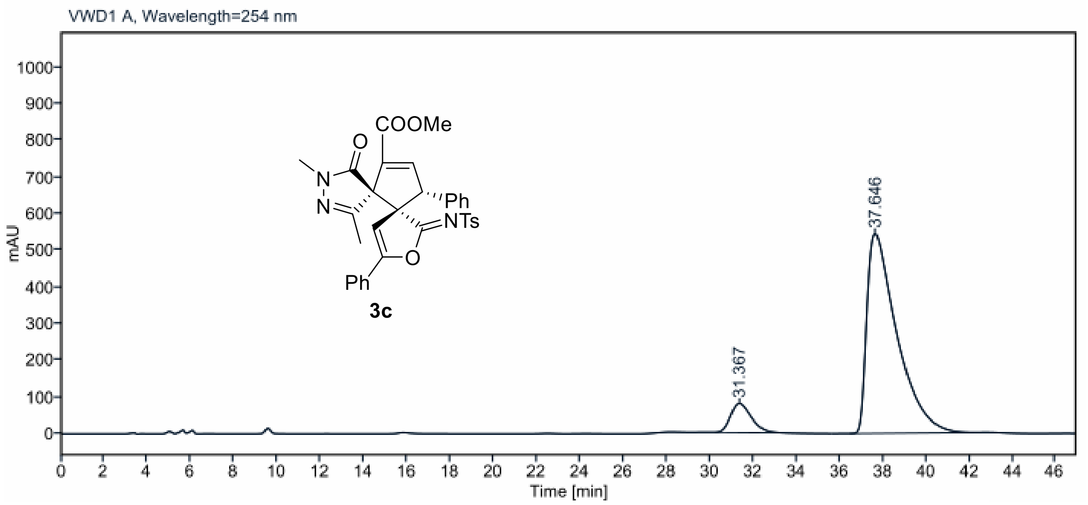

Signal: VWD1 A, Wavelength=254 nm

RT [min]	Type	Width [min]	Area	Height	Area%	Name
31.367	MM	1.1092	5259.6343	79.0273	9.1372	
37.646	BB	1.4158	52303.0156	544.4389	90.8628	
		Sum	57562.6499			

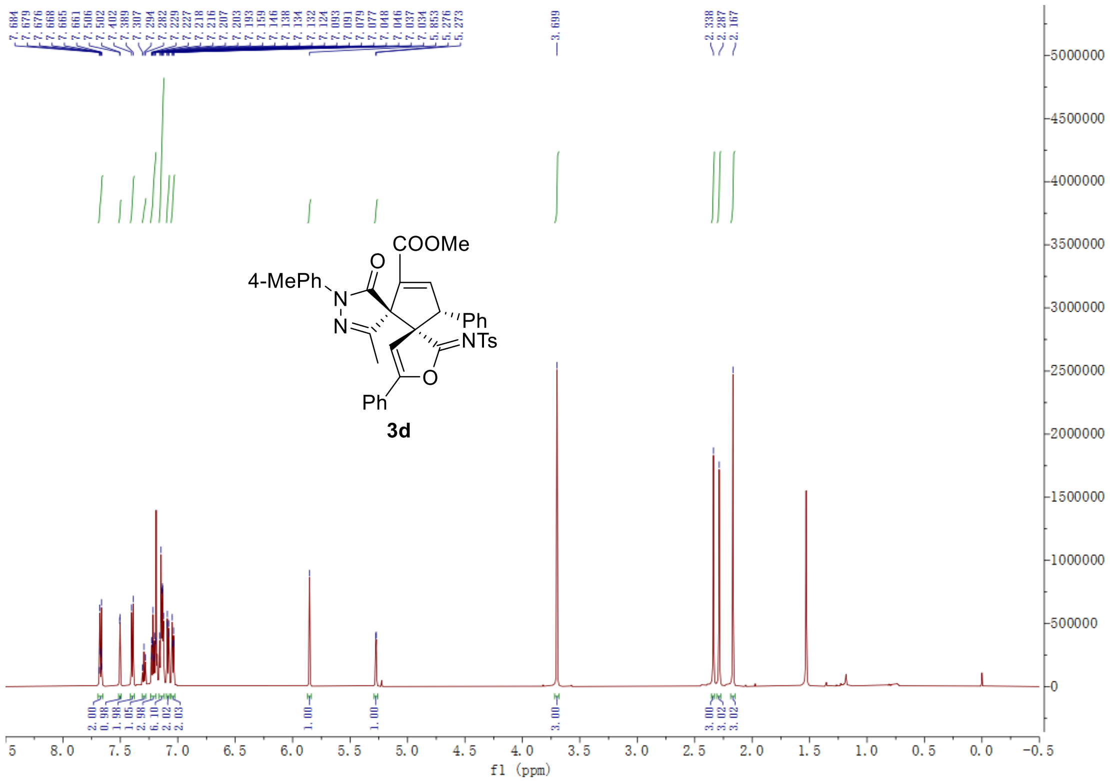

7.684
7.679
7.676
7.668
7.665
7.661
7.506
7.502
7.402
7.389
7.307
7.294
7.282
7.229
7.227
7.218
7.216
7.207
7.203
7.193
7.159
7.146
7.138
7.134
7.132
7.124
7.093
7.091
7.079
7.077
7.048
7.046
7.037
7.034
5.853
5.276
5.273
3.699
2.338
2.287
2.167
COOMe
4-MePh
O
N
N
Ph
NTs
O
Ph
3d
2.00
0.98
1.98
1.05
2.98
6.10
2.02
2.03
1.00
1.00
3.00
3.00
3.02
3.02
f1 (ppm)

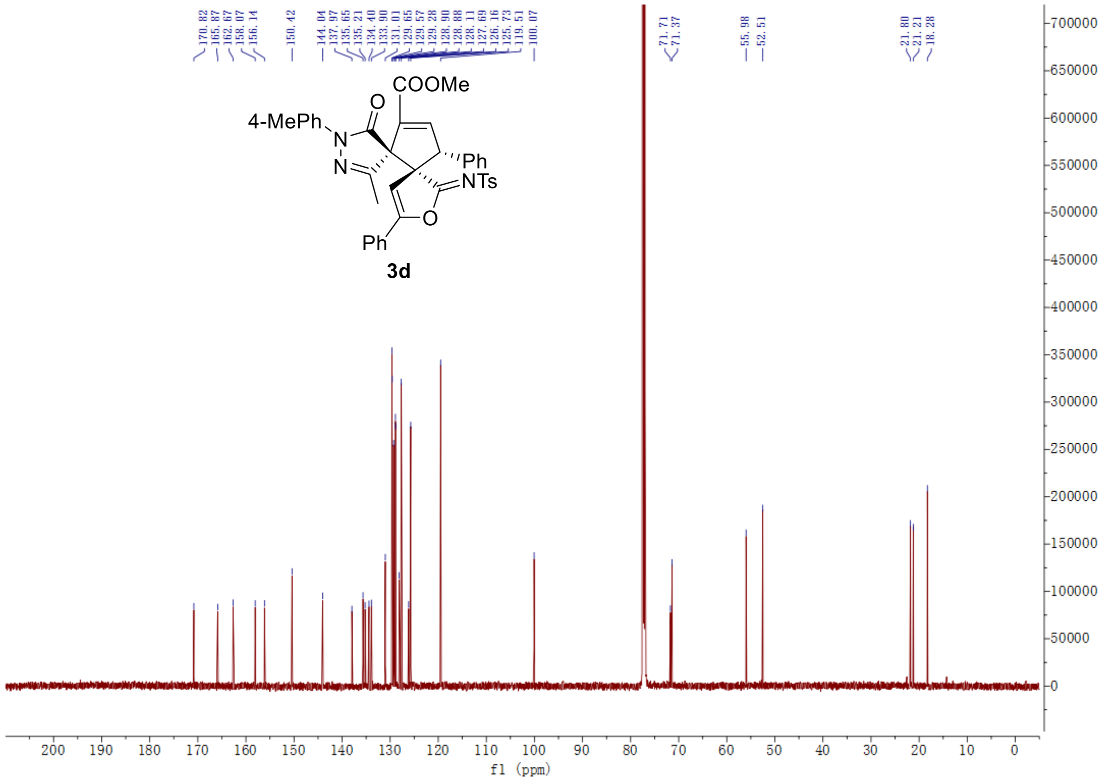

170.82
165.87
162.67
158.07
156.14
150.42
144.04
137.97
135.65
135.21
134.40
133.90
131.01
129.65
129.57
129.28
128.90
128.88
128.11
127.69
126.16
125.73
119.51
100.07
71.71
71.37
55.98
52.51
21.80
21.21
18.28
COOMe
4-MePh
O
N
N
Ph
NTs
O
Ph
3d
f1 (ppm)

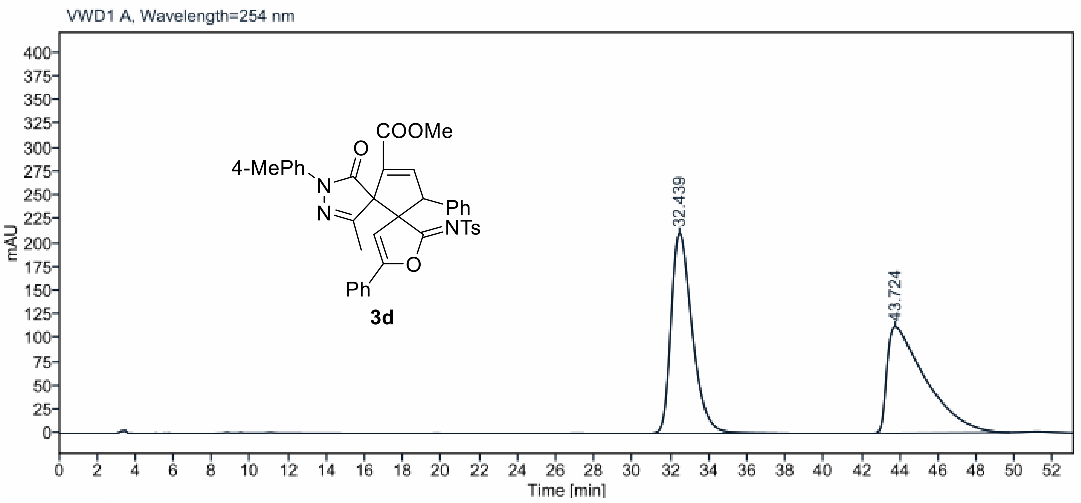

Signal: VWD1 A, Wavelength=254 nm

RT [min]	Type	Width [min]	Area	Height	Area%	Name
32.439	MM	1.2755	16100.4912	210.3792	50.6472	
43.724	BB	1.9404	15689.0137	111.9228	49.3528	
		Sum	31789.5049			

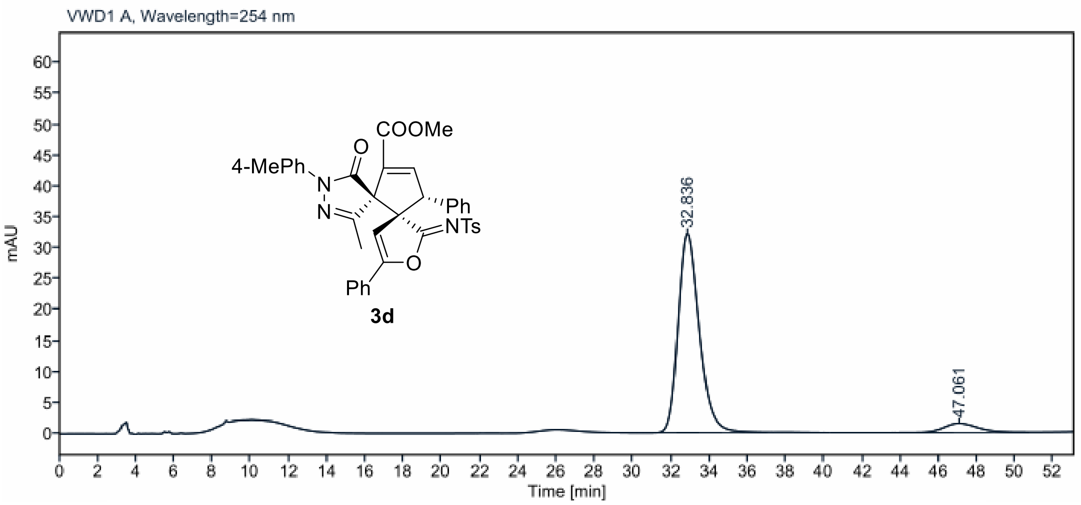

Signal: VWD1 A, Wavelength=254 nm

RT [min]	Type	Width [min]	Area	Height	Area%	Name
32.836	BB	1.1732	2487.8767	32.2087	93.3030	
47.061	MM	2.0313	178.5726	1.4652	6.6970	
		Sum	2666.4493			

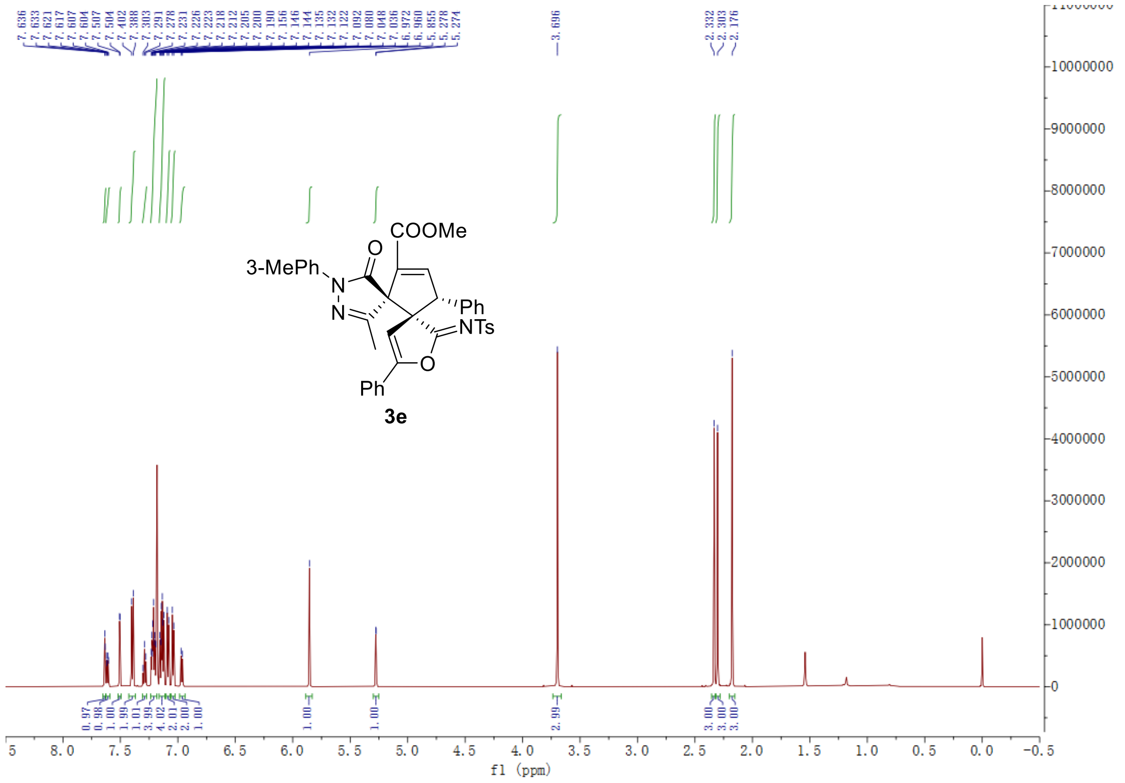

3e

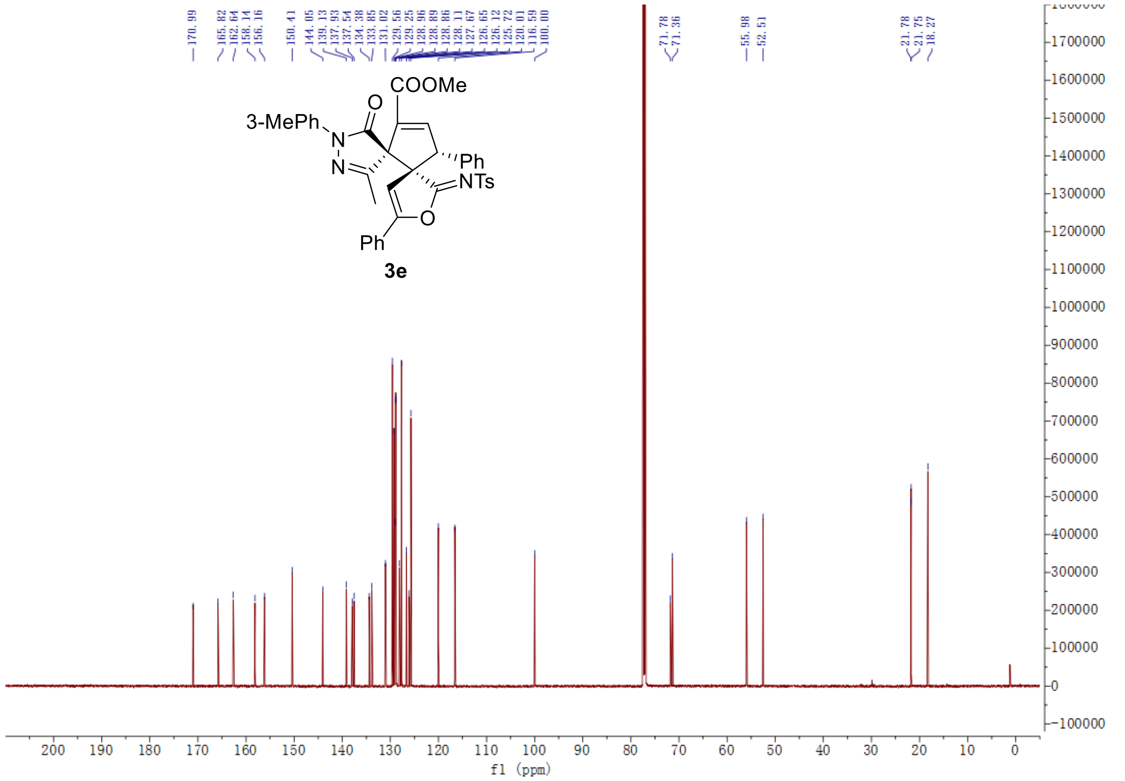

3e

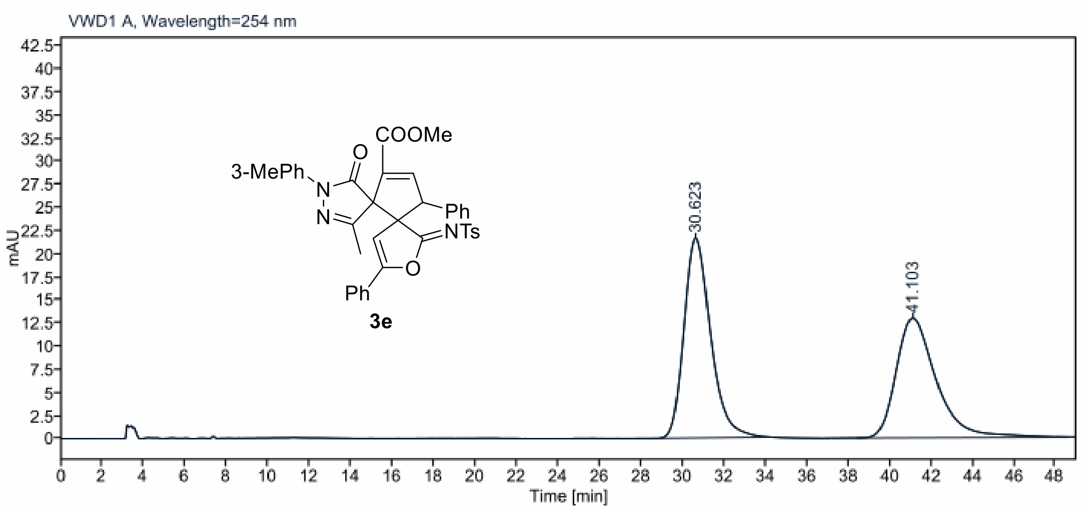

Signal: VWD1 A, Wavelength=254 nm

RT [min]	Type	Width [min]	Area	Height	Area%	Name
30.623	MM	1.4973	1941.2158	21.6082	52.5996	
41.103	MM	2.2521	1749.3358	12.9457	47.4004	
		Sum	3690.5516			

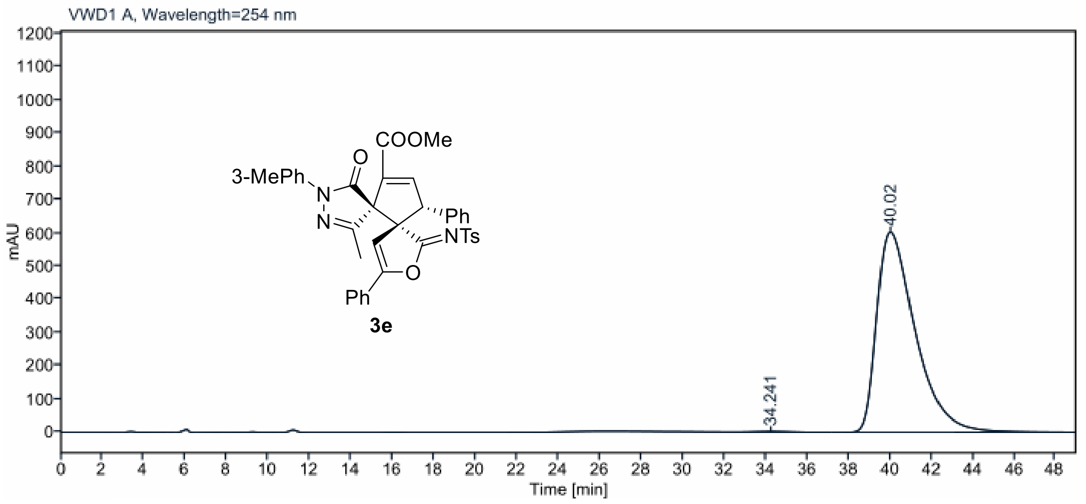

Signal: VWD1 A, Wavelength=254 nm

RT [min]	Type	Width [min]	Area	Height	Area%	Name
34.241	BB	1.3557	252.3979	2.1785	0.3234	
40.020	MM	2.1515	77801.8750	602.6919	99.6766	
		Sum	78054.2729			

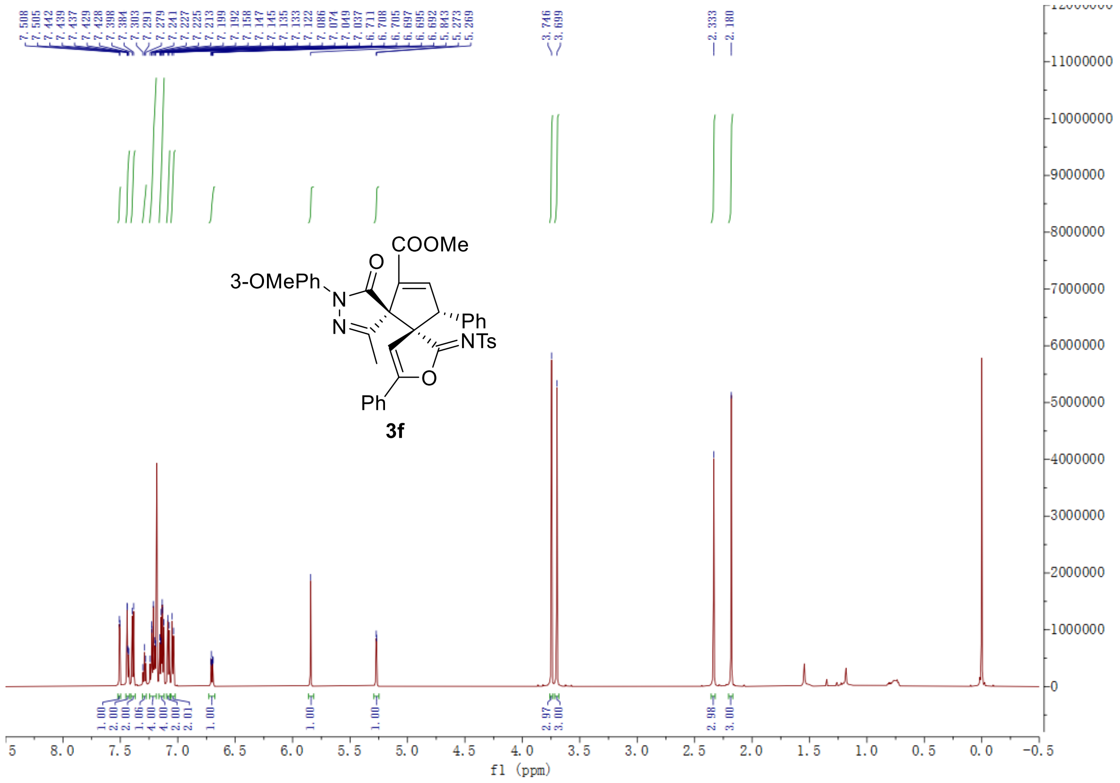
7.508
7.505
7.442
7.439
7.437
7.429
7.428
7.398
7.384
7.303
7.291
7.279
7.241
7.227
7.225
7.213
7.199
7.192
7.158
7.147
7.145
7.135
7.133
7.122
7.086
7.074
7.049
7.037
6.711
6.708
6.705
6.697
6.695
6.692
5.843
5.273
5.269
3.746
3.699
2.333
2.180
COOMe
O
3-OMePh
N
N
Ph
NTs
O
Ph
3f
1.00
2.03
2.00
1.06
4.00
4.00
2.00
2.01
1.00
1.00
1.00
2.97
3.00
2.98
3.00
f1 (ppm)

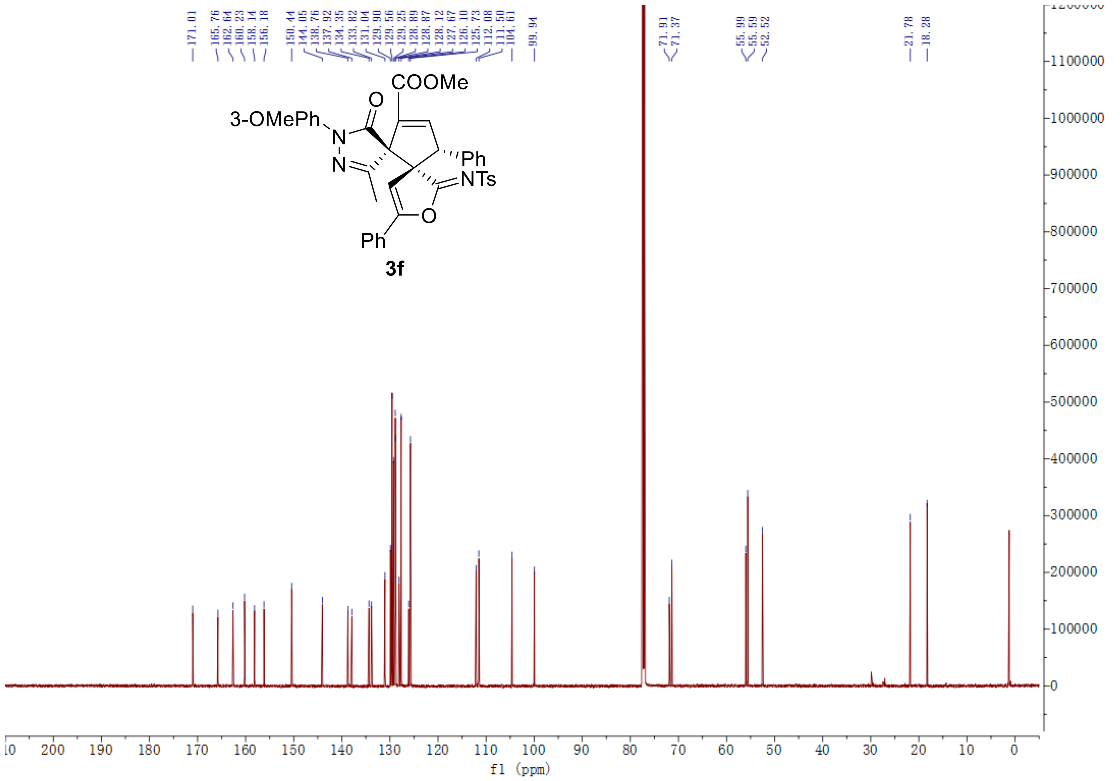
171.01
165.76
162.64
160.23
158.14
156.18
150.44
144.05
138.76
137.92
134.35
133.82
131.04
129.90
129.56
129.25
128.89
128.87
128.12
127.67
126.10
125.73
112.08
111.50
104.61
99.94
71.91
71.37
55.99
55.59
52.52
21.78
18.28
COOMe
O
3-OMePh
N
N
Ph
NTs
O
Ph
3f
f1 (ppm)

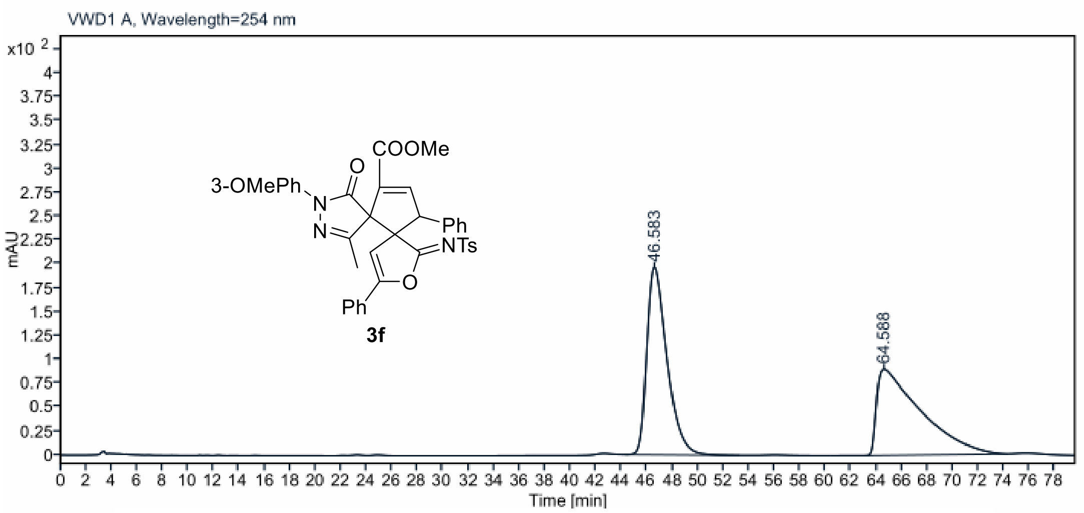

Signal: VWD1 A, Wavelength=254 nm

RT [min]	Type	Width [min]	Area	Height	Area%	Name
46.583	BB	1.6733	21724.5273	196.2782	50.1217	
64.588	BB	3.1605	21619.0508	90.3215	49.8783	
		Sum	43343.5781			

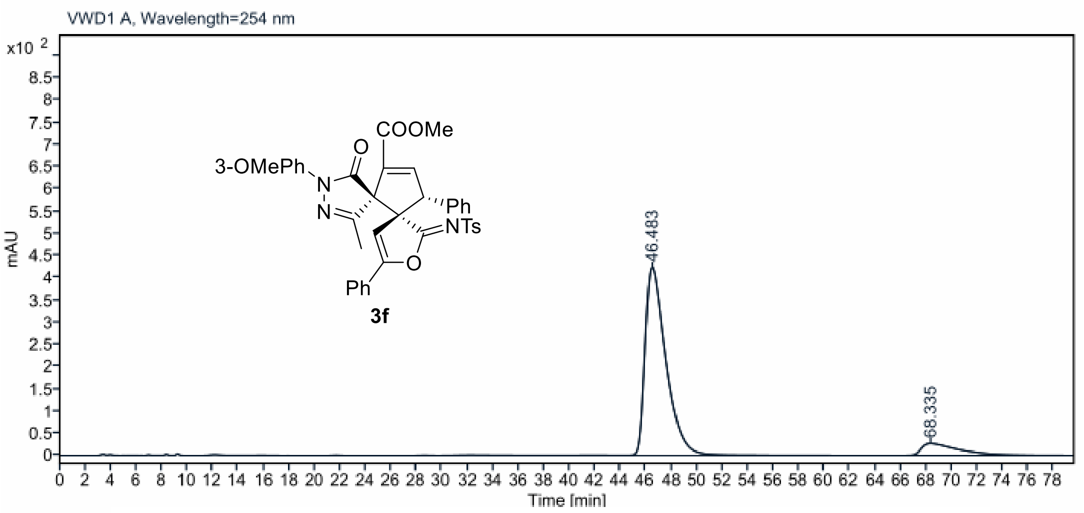

Signal: VWD1 A, Wavelength=254 nm

RT [min]	Type	Width [min]	Area	Height	Area%	Name
46.483	MM	1.8879	47956.7813	423.3804	89.7054	
68.335	MM	3.3724	5503.5205	27.1986	10.2946	
		Sum	53460.3018			

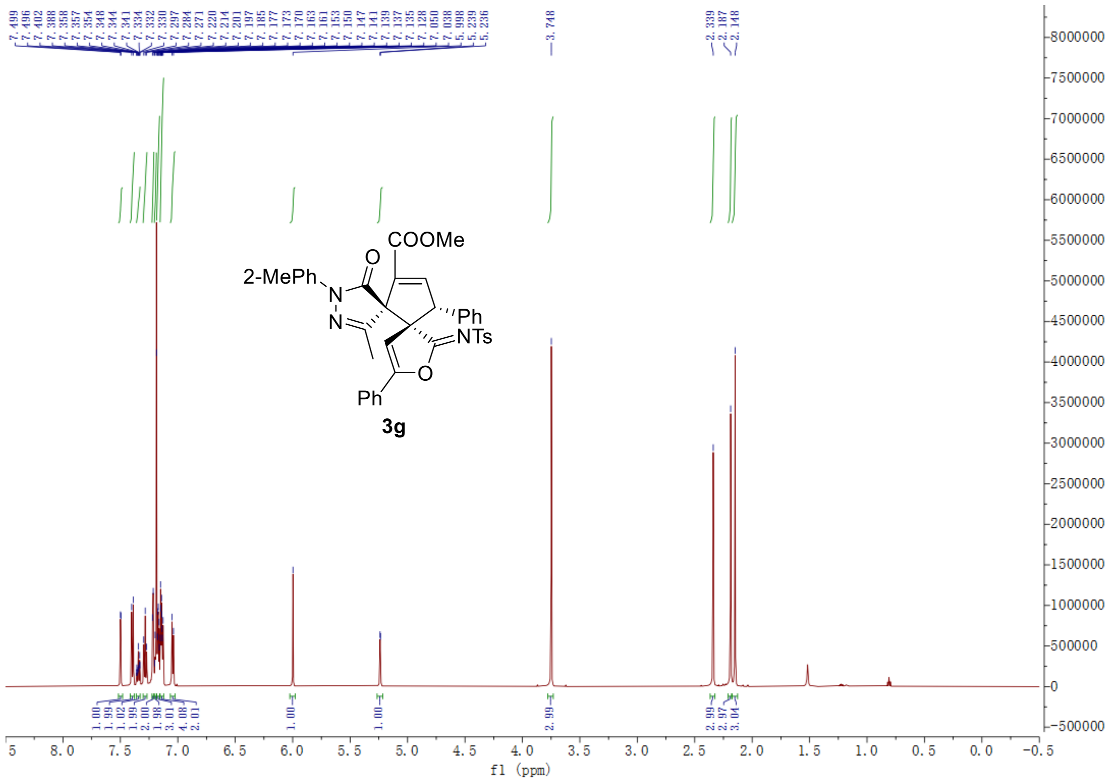

COOMe
2-MePh
N
N
O
Ph
NTs
O
Ph
3g
fl (ppm)

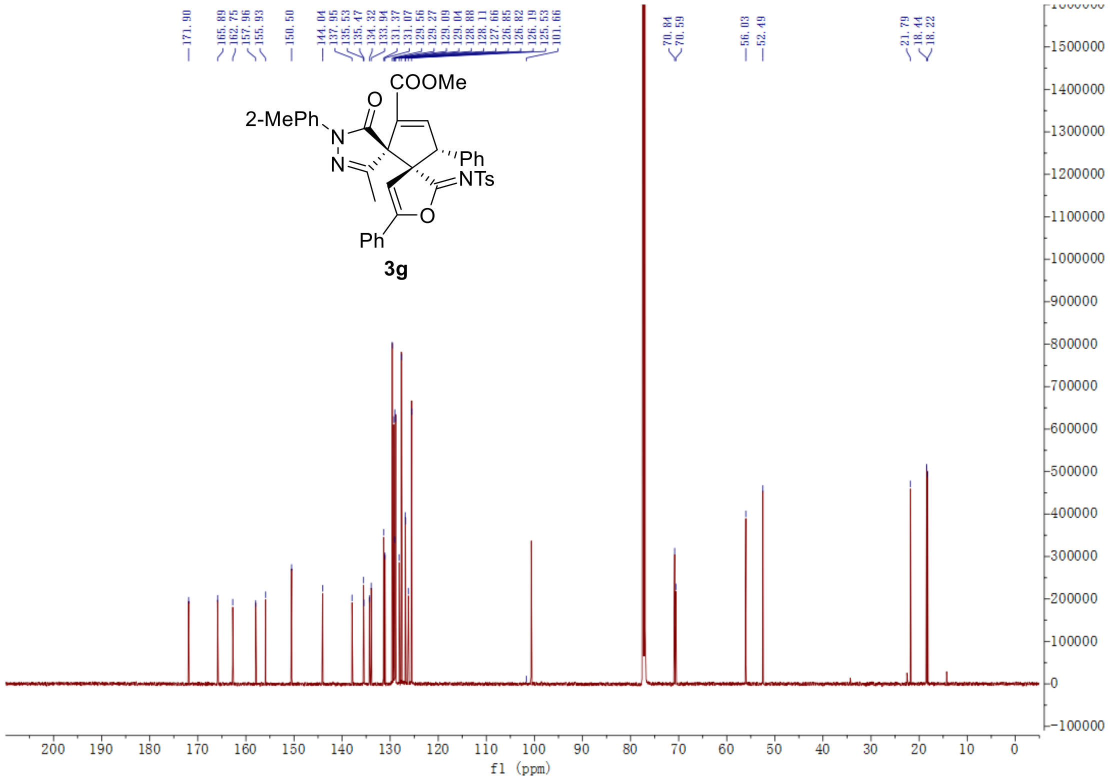

COOMe
2-MePh
N
N
O
Ph
NTs
O
Ph
3g
fl (ppm)

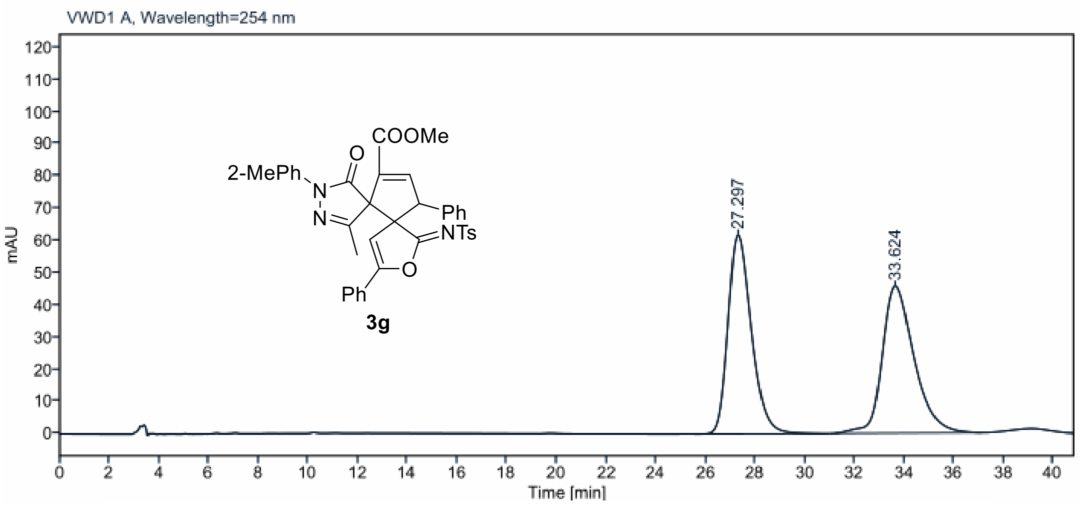

Signal: VWD1 A, Wavelength=254 nm

RT [min]	Type	Width [min]	Area	Height	Area%	Name
27.297	BB	1.0040	4036.0615	61.7929	49.8845	
33.624	MM	1.4803	4054.7510	45.6511	50.1155	
		Sum	8090.8125			

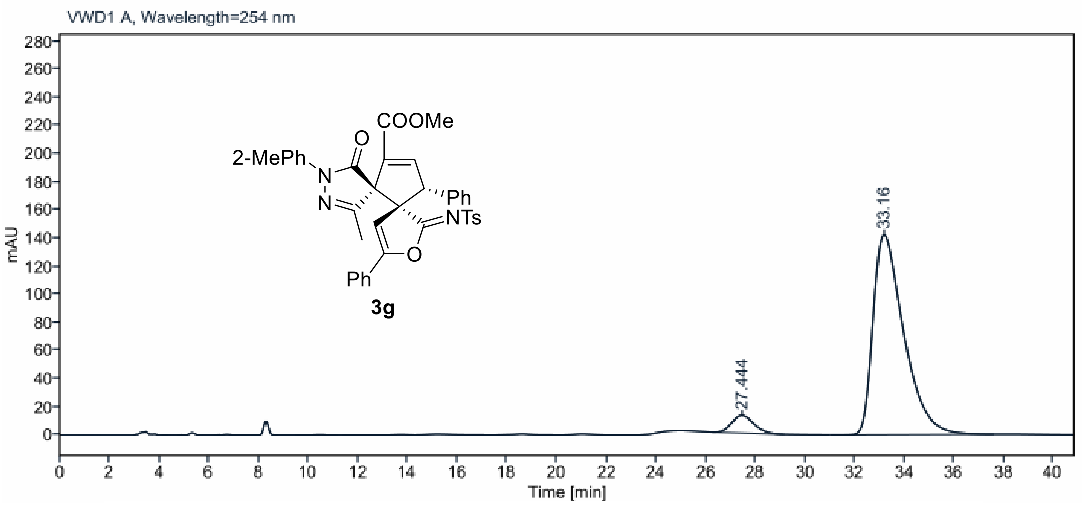

Signal: VWD1 A, Wavelength=254 nm

RT [min]	Type	Width [min]	Area	Height	Area%	Name
27.444	MM	1.0536	789.9116	12.4958	6.0735	
33.160	BB	1.2933	12215.9248	142.1882	93.9265	
		Sum	13005.8364			

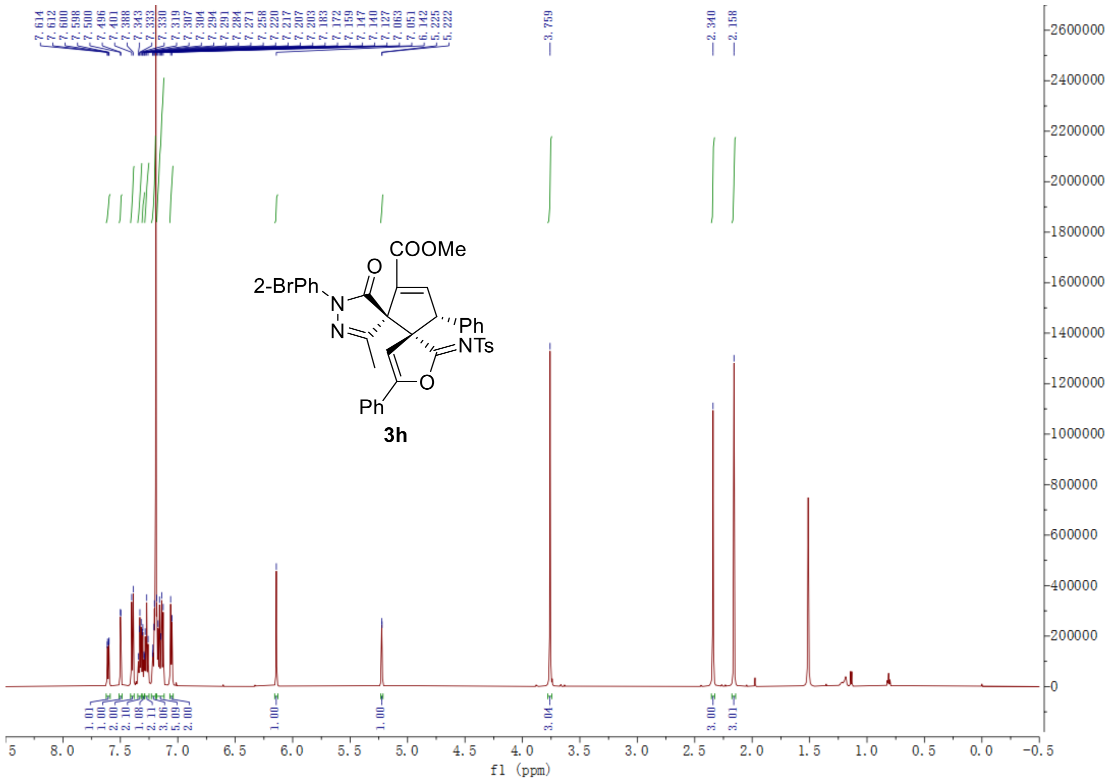
7.614
7.612
7.600
7.598
7.500
7.496
7.401
7.388
7.343
7.333
7.330
7.319
7.307
7.304
7.294
7.291
7.284
7.271
7.258
7.220
7.217
7.207
7.203
7.183
7.172
7.159
7.147
7.140
7.127
7.063
7.051
6.142
5.225
5.222
3.759
2.340
2.158
COOMe
O
2-BrPh
N
N
Ph
NTs
O
Ph
3h
f1 (ppm)

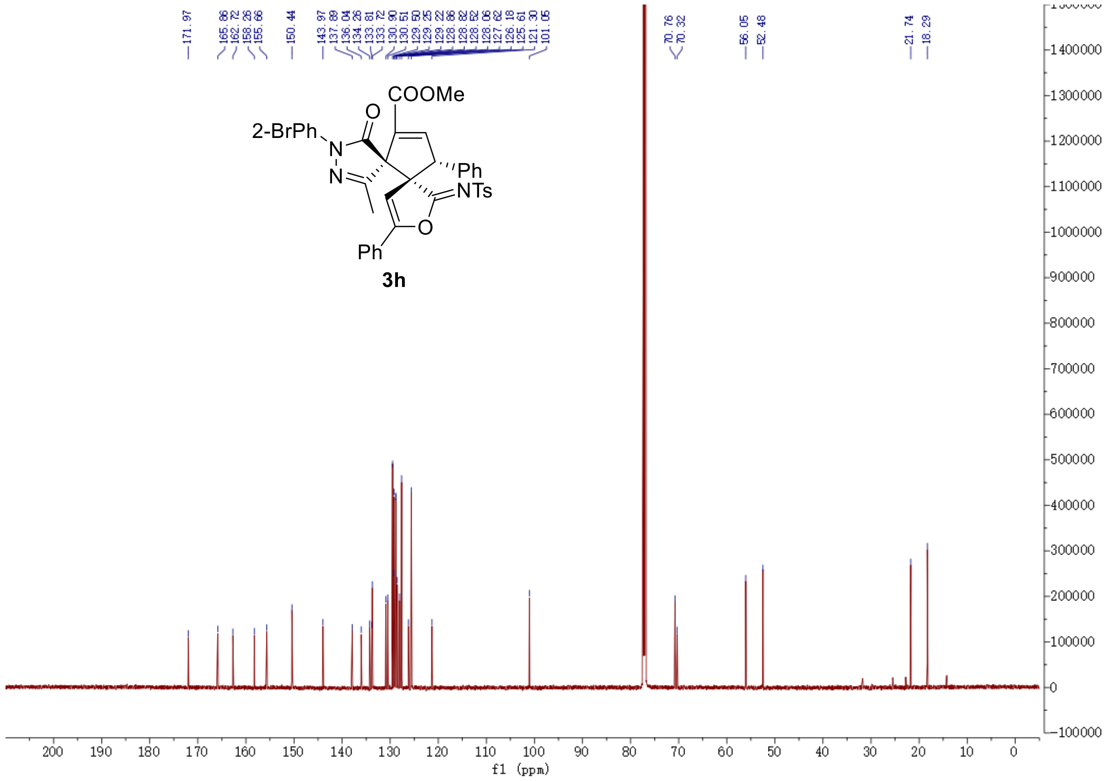
171.97
165.86
162.72
158.28
155.66
150.44
143.97
137.89
136.04
134.26
133.81
133.72
130.90
130.51
129.50
129.25
129.22
128.86
128.82
128.52
128.06
127.62
126.18
125.61
121.30
101.05
70.76
70.32
56.05
52.48
21.74
18.29
COOMe
O
2-BrPh
N
N
Ph
NTs
O
Ph
3h
f1 (ppm)

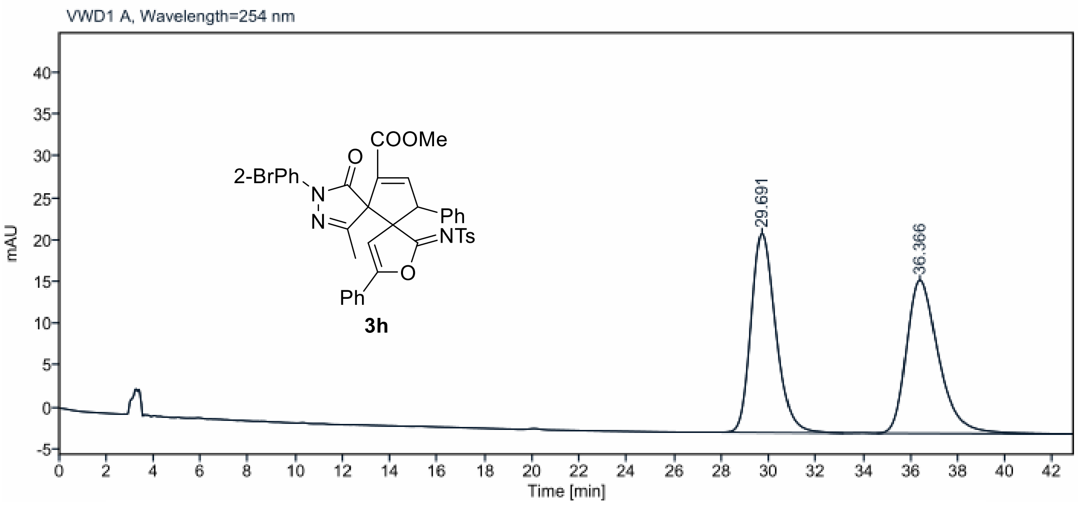

Signal: VWD1 A, Wavelength=254 nm

RT [min]	Type	Width [min]	Area	Height	Area%	Name
29.691	BB	1.1058	1728.3892	23.8127	50.0770	
36.366	BB	1.4025	1723.0747	18.2995	49.9230	
		Sum	3451.4639			

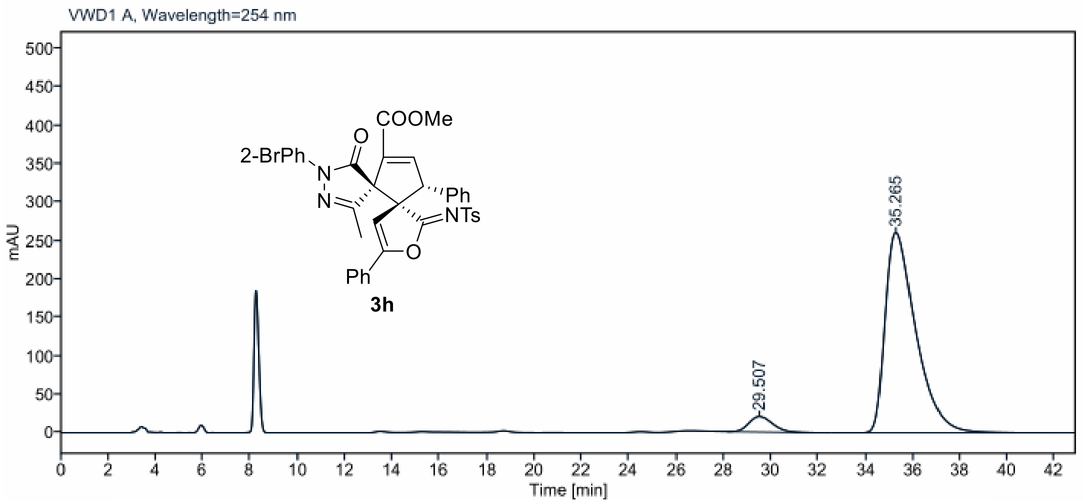

Signal: VWD1 A, Wavelength=254 nm

RT [min]	Type	Width [min]	Area	Height	Area%	Name
29.507	MM	1.1616	1428.7961	20.5006	5.7136	
35.265	MM	1.5062	23578.1855	260.9058	94.2864	
		Sum	25006.9817			

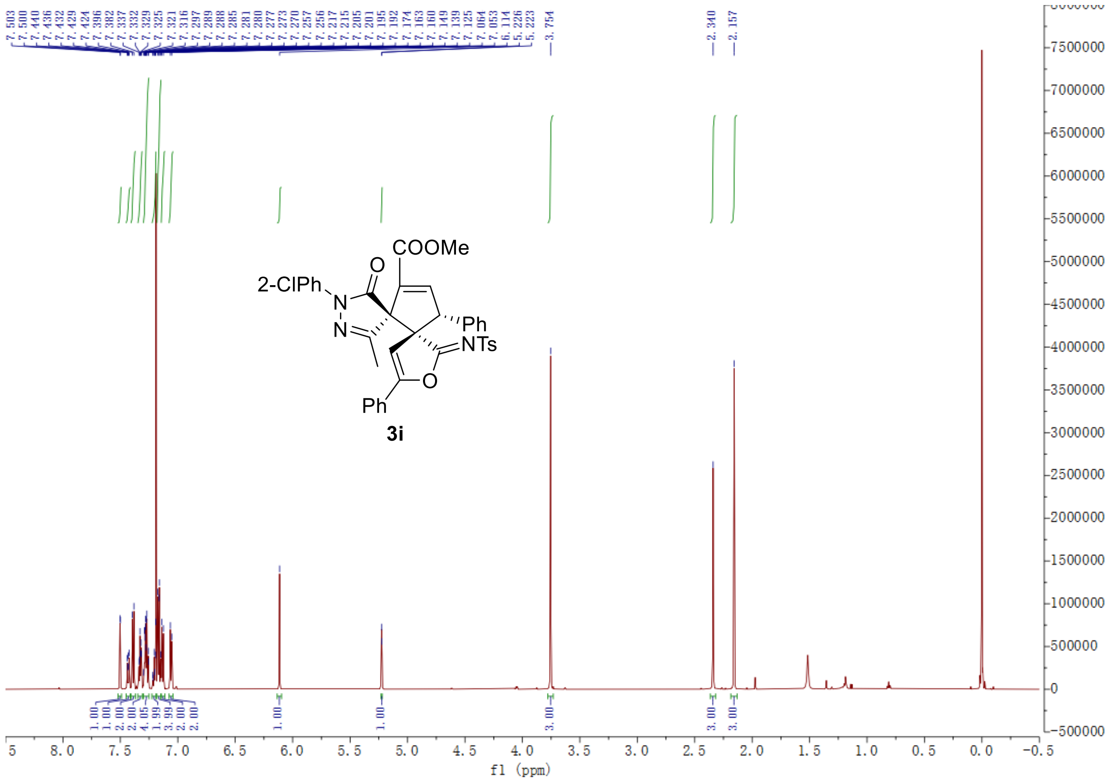
COOMe
2-ClPh
N
N
O
Ph
NTs
Ph
3i
f1 (ppm)

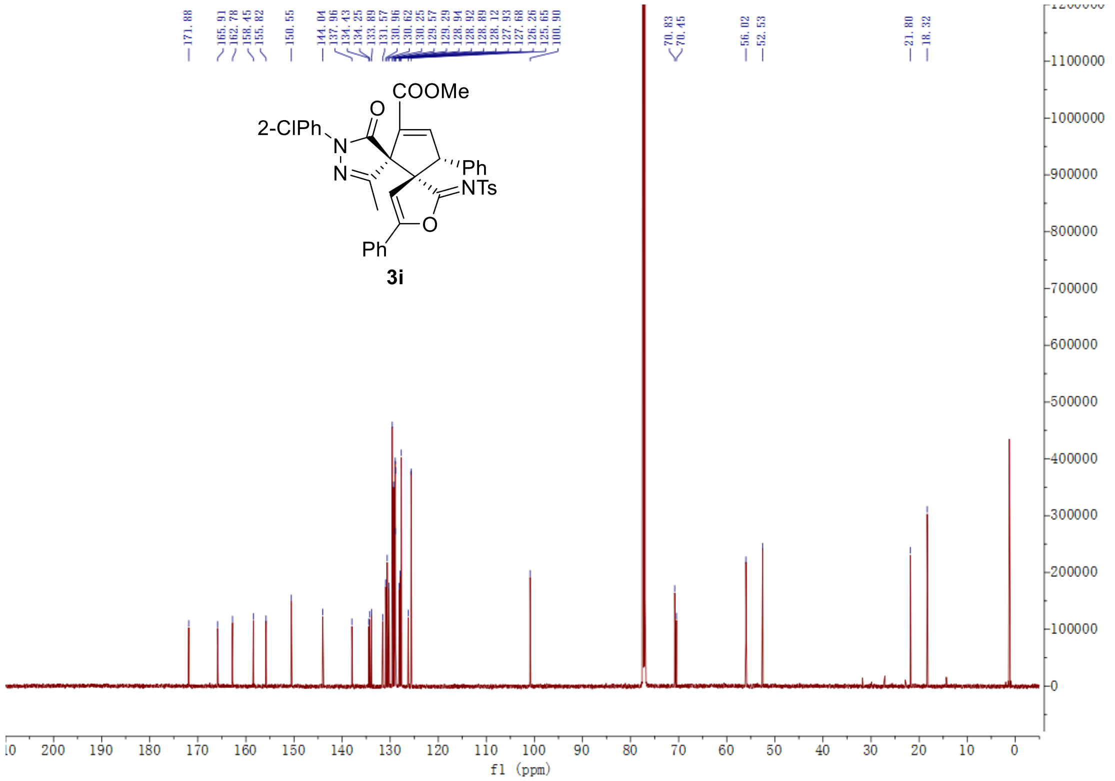
COOMe
2-ClPh
N
N
O
Ph
NTs
Ph
3i
f1 (ppm)

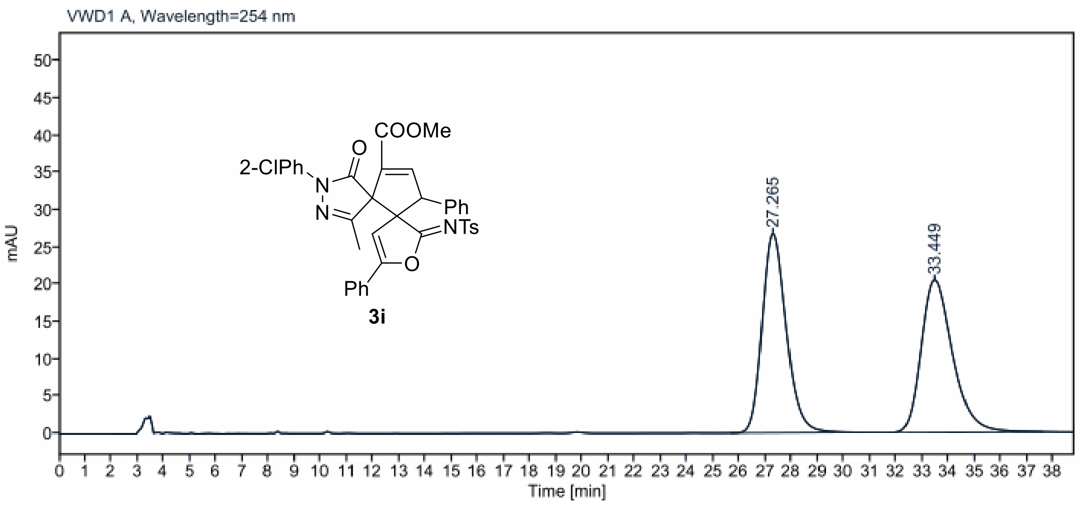

Signal: VWD1 A, Wavelength=254 nm

RT [min]	Type	Width [min]	Area	Height	Area%	Name
27.265	BB	0.9849	1717.4866	26.7215	50.1115	
33.449	BB	1.2482	1709.8457	20.4131	49.8885	
		Sum	3427.3323			

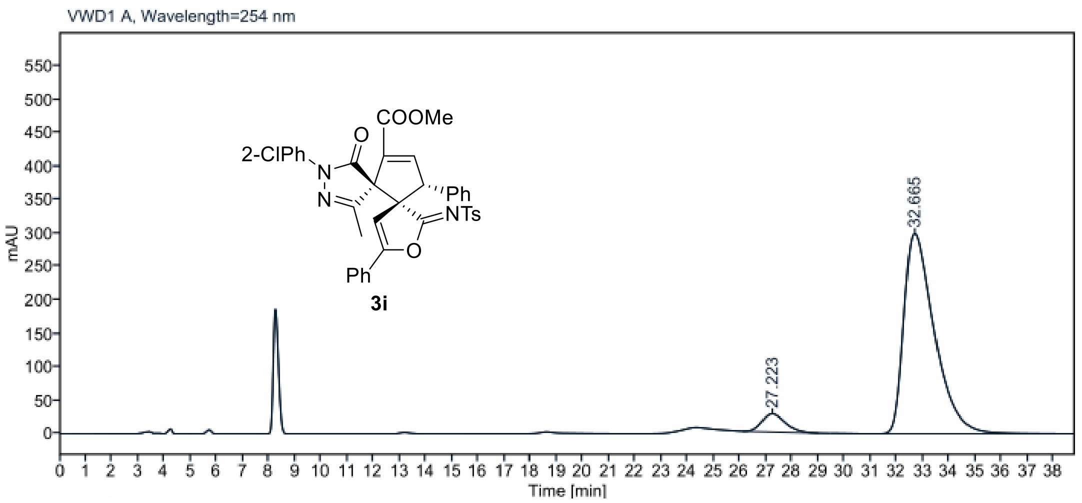

Signal: VWD1 A, Wavelength=254 nm

RT [min]	Type	Width [min]	Area	Height	Area%	Name
27.223	MM	1.0445	1701.3143	27.1484	6.5242	
32.665	MM	1.3569	24375.5469	299.3999	93.4758	
		Sum	26076.8612			

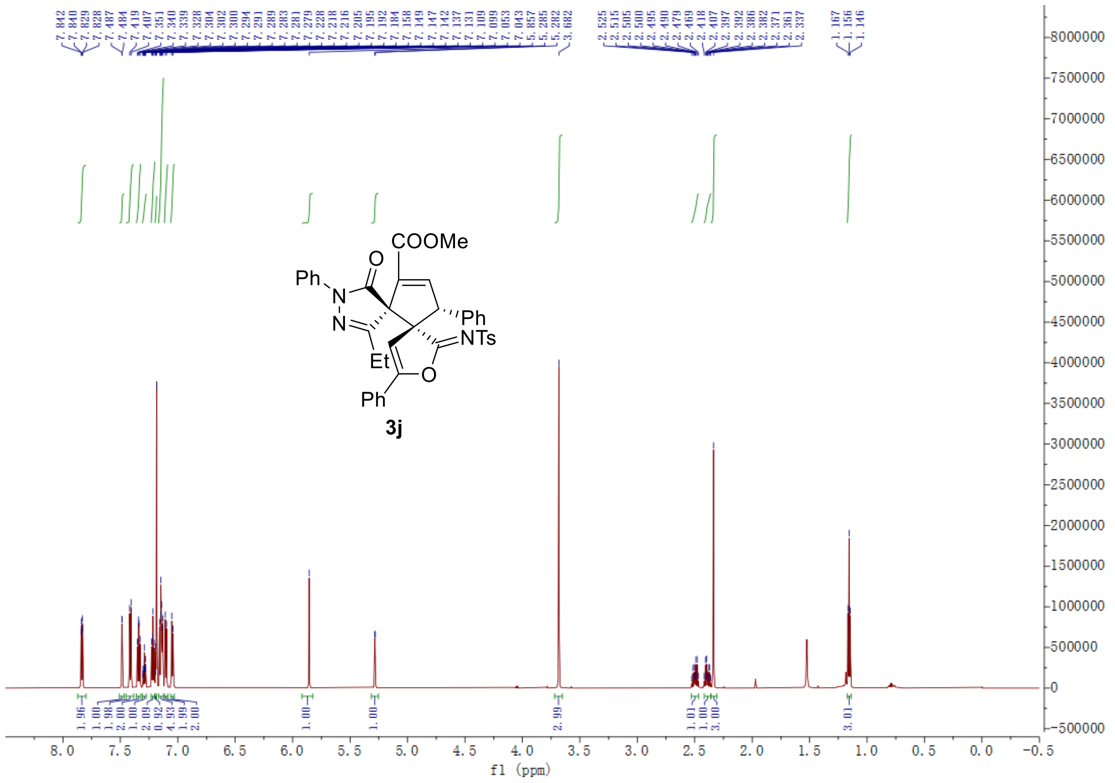

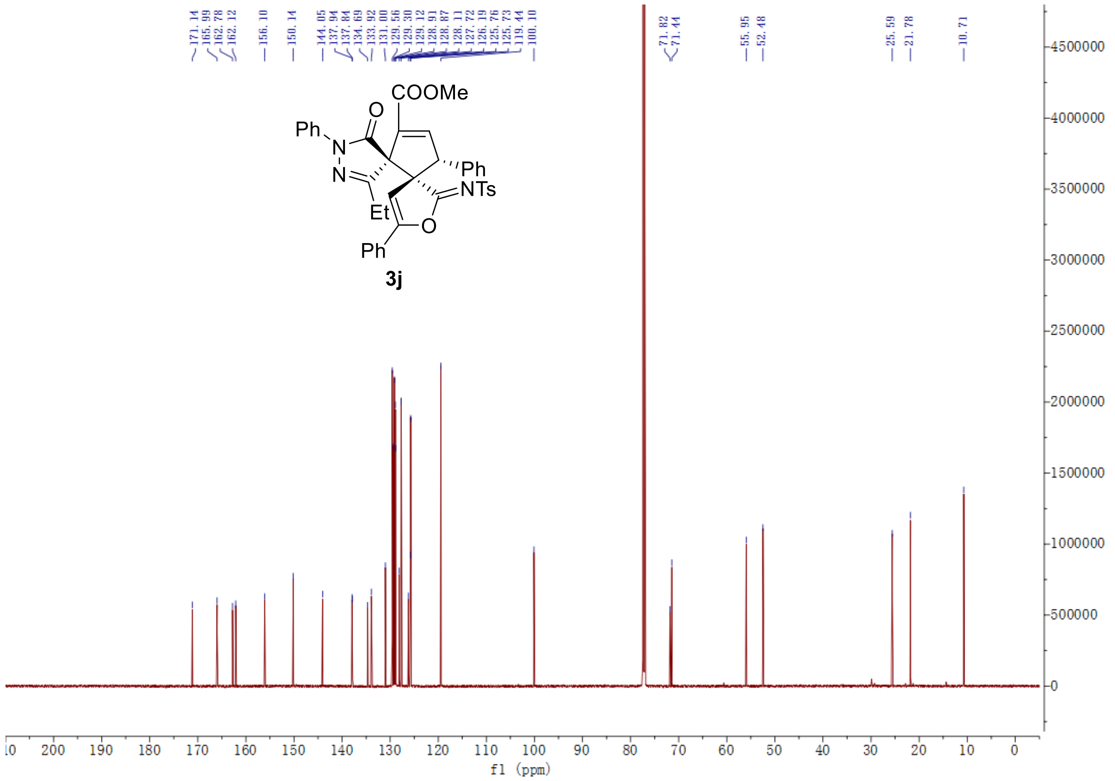

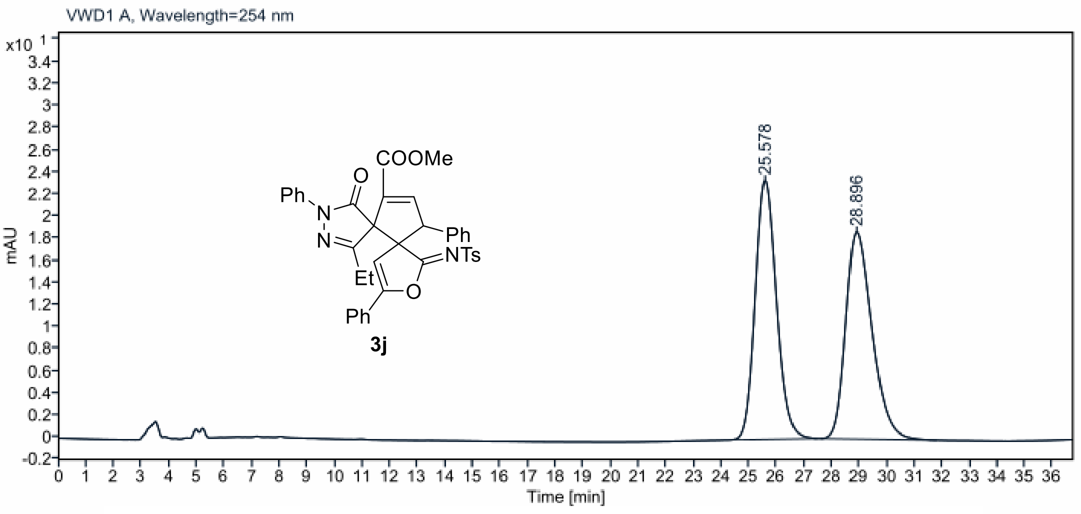

Signal: VWD1 A, Wavelength=254 nm

RT [min]	Type	Width [min]	Area	Height	Area%	Name
25.578	MM	0.8915	1251.6298	23.3987	50.1024	
28.896	MM	1.1099	1246.5138	18.7189	49.8976	
		Sum	2498.1436			

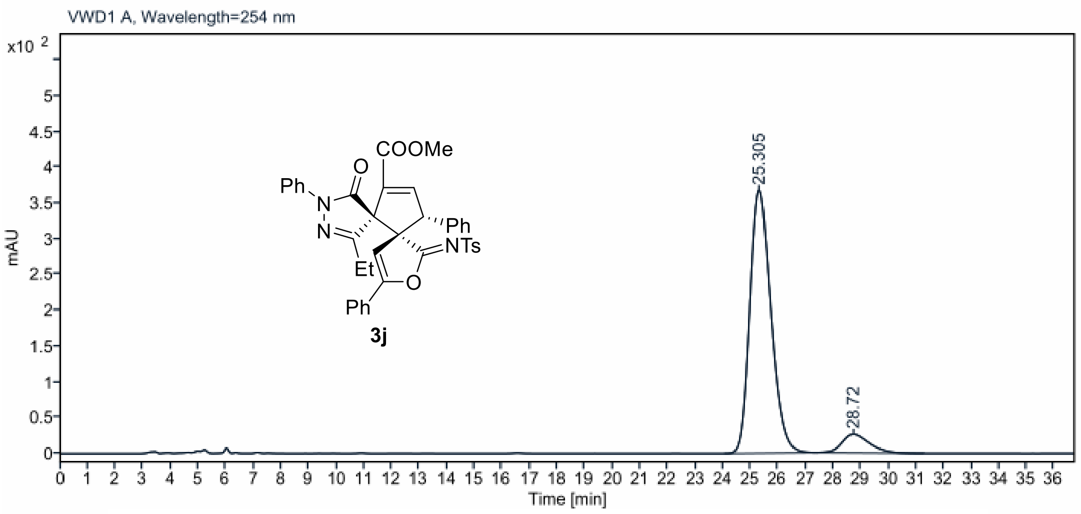

Signal: VWD1 A, Wavelength=254 nm

RT [min]	Type	Width [min]	Area	Height	Area%	Name
25.305	BB	0.8245	19552.9727	367.6474	91.5361	
28.720	BB	1.0544	1807.9575	26.1565	8.4639	
		Sum	21360.9302			

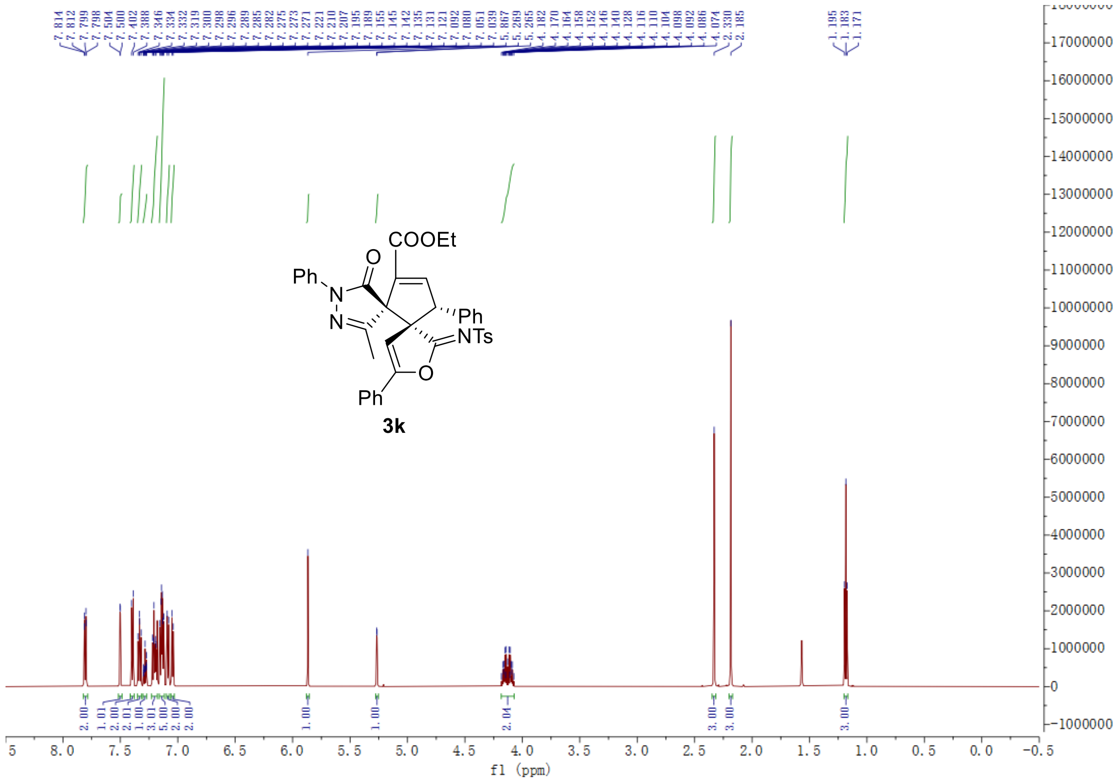
COOEt
Ph
N
N
O
Ph
NTs
O
Ph
3k
f1 (ppm)

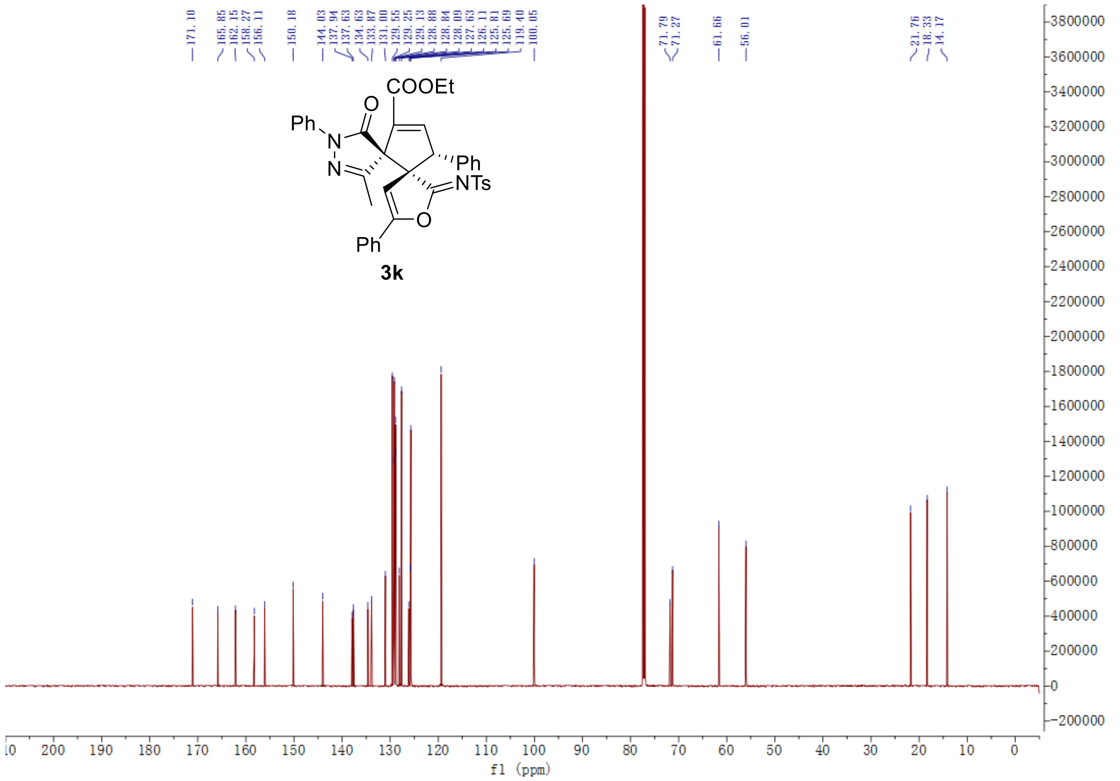
COOEt
Ph
N
N
O
Ph
NTs
O
Ph
3k
f1 (ppm)

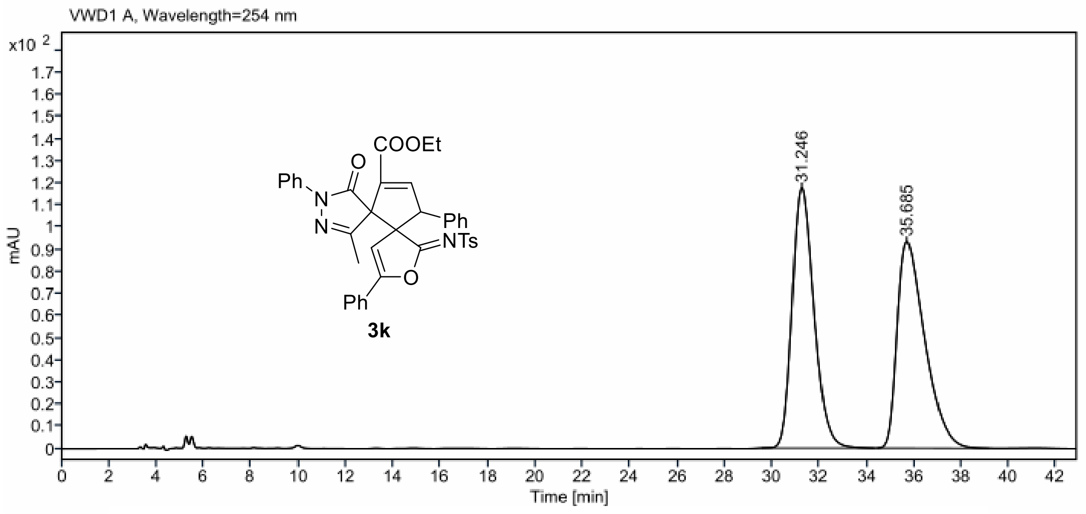

Signal: VWD1 A, Wavelength=254 nm

RT [min]	Type	Width [min]	Area	Height	Area%	Name
31.246	MM	1.0720	7567.2686	117.6493	49.9010	
35.685	MM	1.3564	7597.2832	93.3508	50.0990	
		Sum	15164.5518			

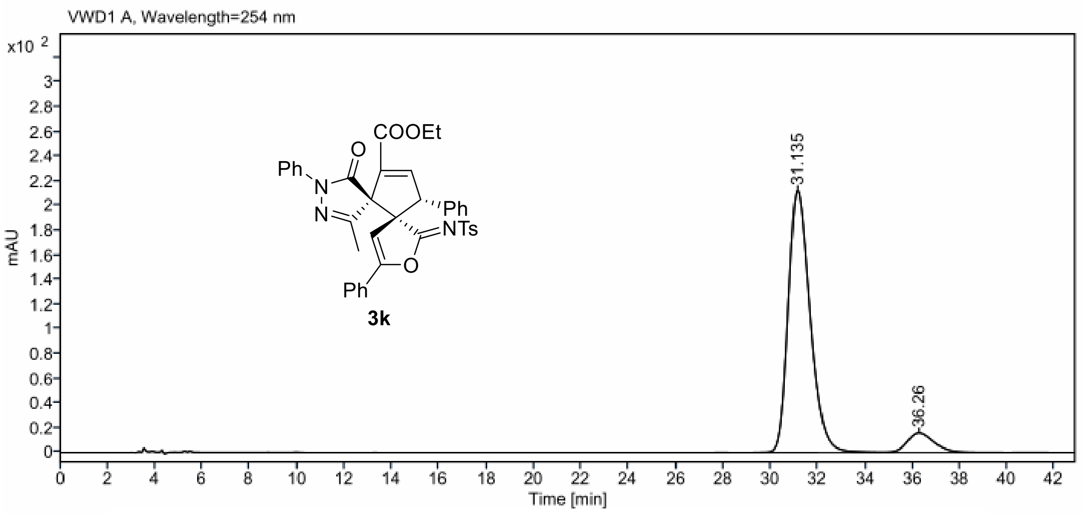

Signal: VWD1 A, Wavelength=254 nm

RT [min]	Type	Width [min]	Area	Height	Area%	Name
31.135	MM	1.0686	13606.3496	212.2161	91.8274	
36.260	MM	1.2876	1210.9546	15.6750	8.1726	
		Sum	14817.3042			

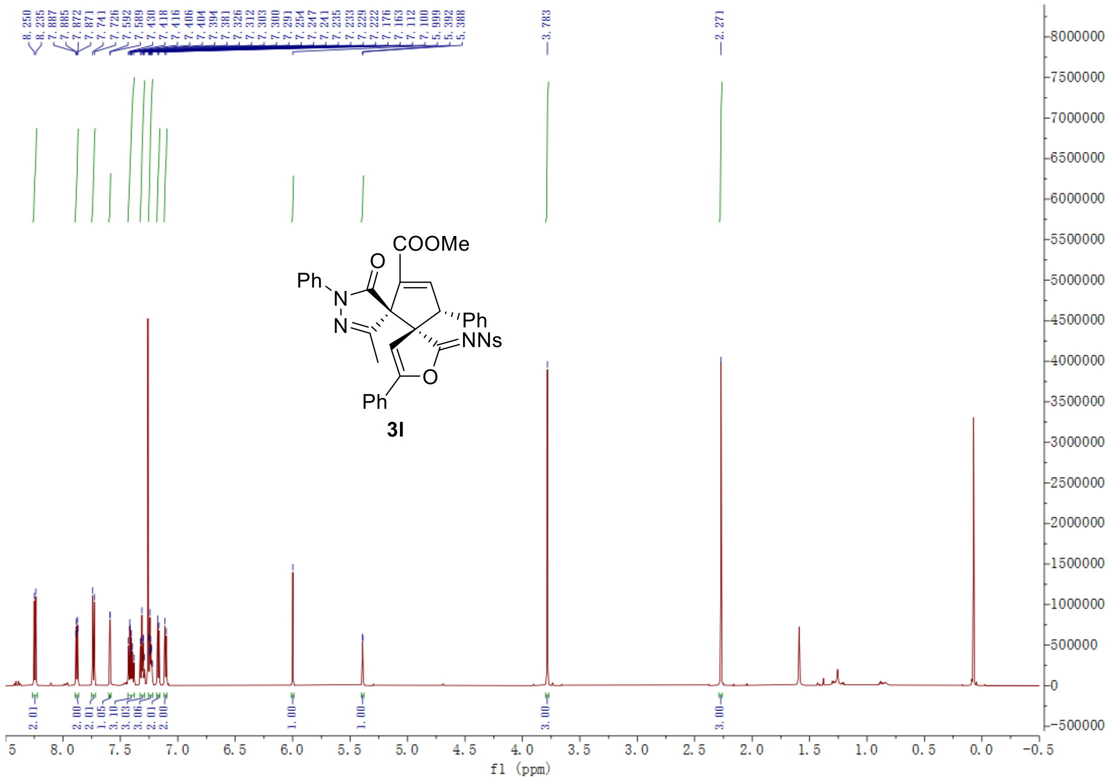
8.250
8.235
7.887
7.885
7.872
7.871
7.741
7.726
7.592
7.589
7.430
7.418
7.416
7.406
7.404
7.394
7.381
7.326
7.312
7.303
7.300
7.291
7.254
7.247
7.241
7.235
7.233
7.229
7.222
7.176
7.163
7.112
7.100
5.999
5.392
5.388
3.783
2.271
COOMe
O
Ph
N
N
Ph
NNs
O
Ph
3l
2.01
2.00
2.01
1.05
3.10
3.03
3.06
2.01
2.00
1.00
1.00
3.00
3.00
f1 (ppm)

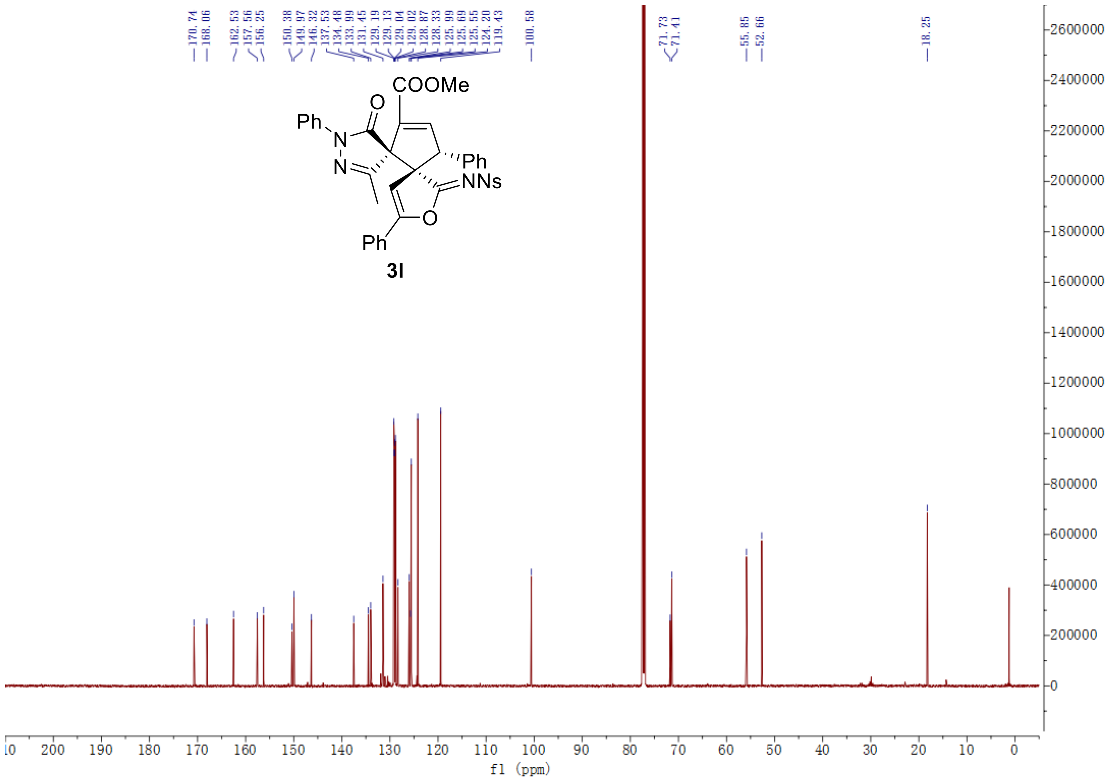
170.74
168.06
162.53
157.56
156.25
150.38
149.97
146.32
137.53
134.48
133.99
131.45
129.19
129.13
129.04
129.02
128.87
128.33
125.99
125.55
124.20
119.43
100.58
71.73
71.41
55.85
52.66
18.25
COOMe
O
Ph
N
N
Ph
NNs
O
Ph
3l
f1 (ppm)

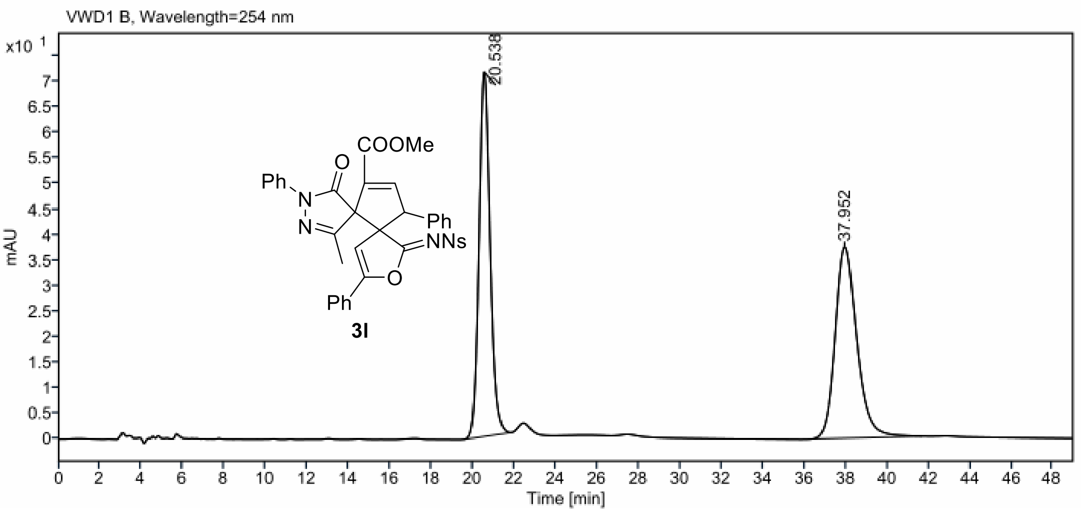

Signal: VWD1 B, Wavelength=254 nm

RT [min]	Type	Width [min]	Area	Height	Area%	Name
20.538	BB	0.5773	2687.2197	71.3700	49.6956	
37.952	MM	1.2123	2720.1365	37.3976	50.3044	
		Sum	5407.3562			

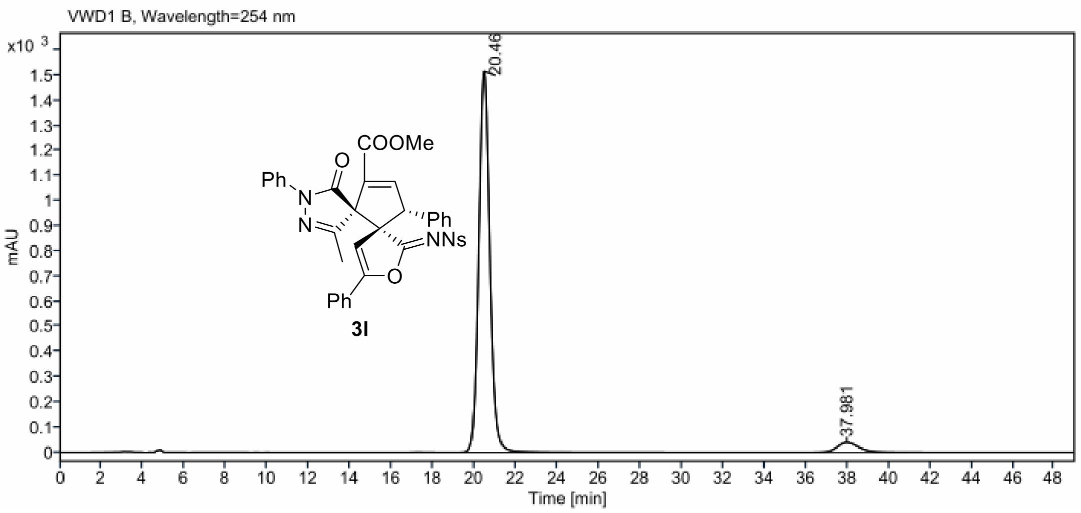

Signal: VWD1 B, Wavelength=254 nm

RT [min]	Type	Width [min]	Area	Height	Area%	Name
20.460	MM	0.6261	56958.5078	1516.2987	95.1942	
37.981	MM	1.2071	2875.4863	39.7017	4.8058	
		Sum	59833.9941			

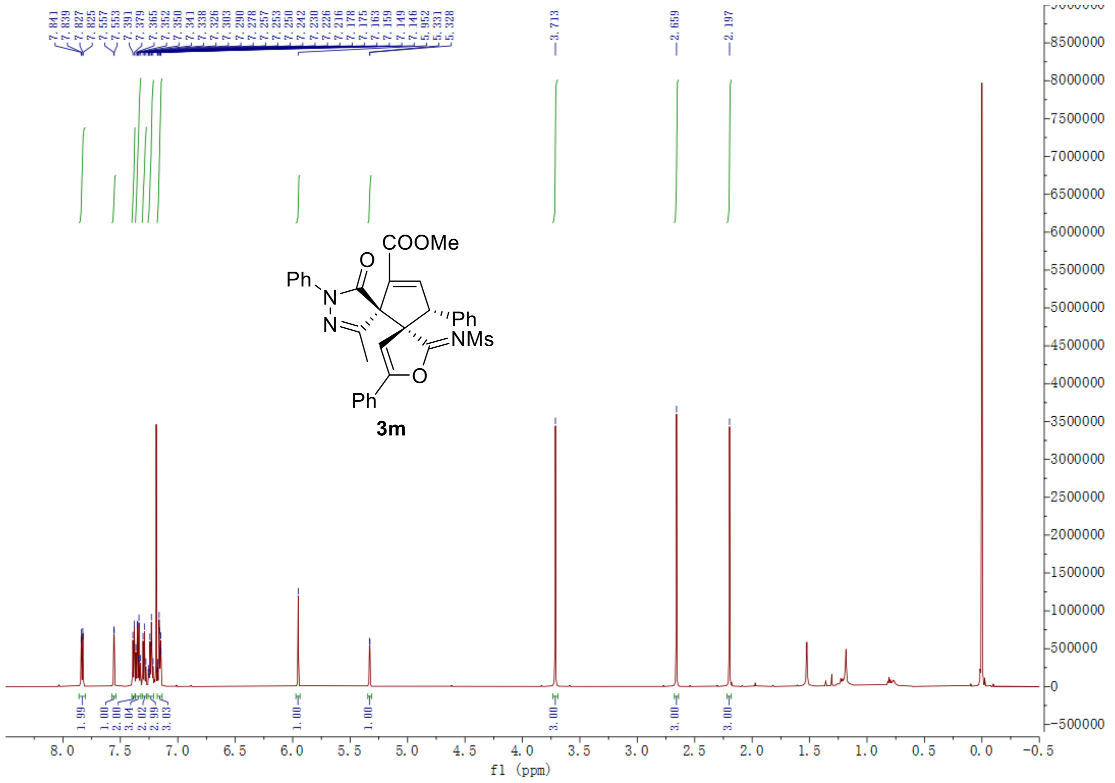
COOMe
Ph
N
N
O
Ph
NMs
O
Ph
3m
f1 (ppm)

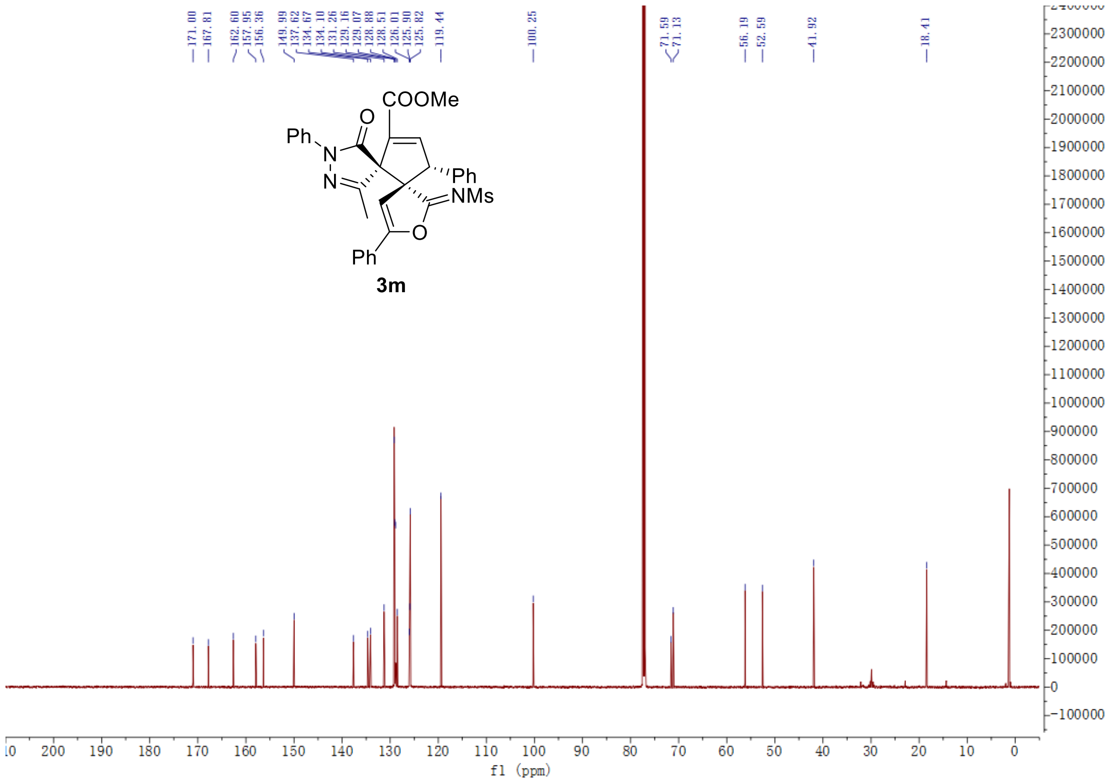
COOMe
Ph
N
N
O
Ph
NMs
O
Ph
3m
f1 (ppm)

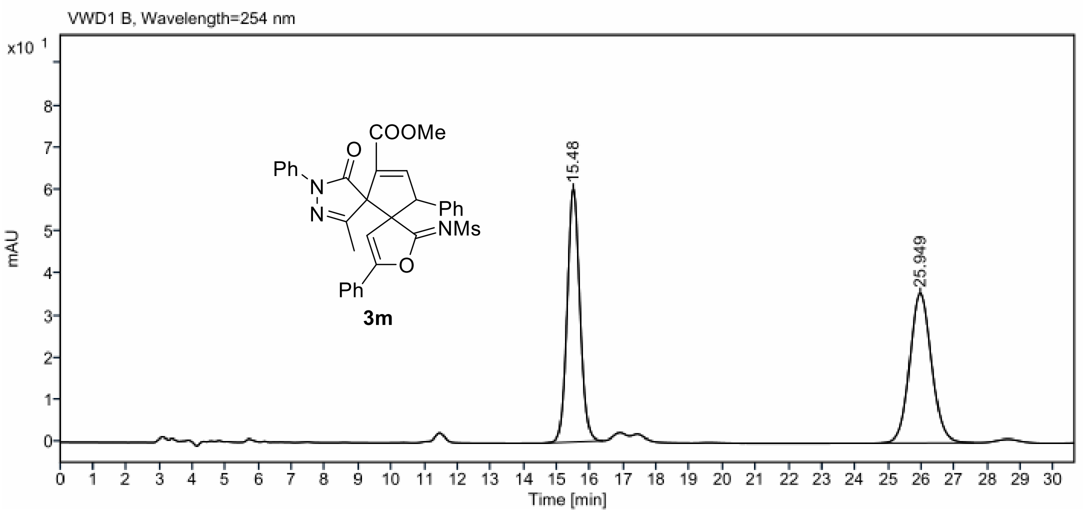

Signal: VWD1 B, Wavelength=254 nm

RT [min]	Type	Width [min]	Area	Height	Area%	Name
15.480	BB	0.4164	1623.7627	60.2538	49.7972	
25.949	MM	0.7655	1636.9896	35.6414	50.2028	
		Sum	3260.7523			

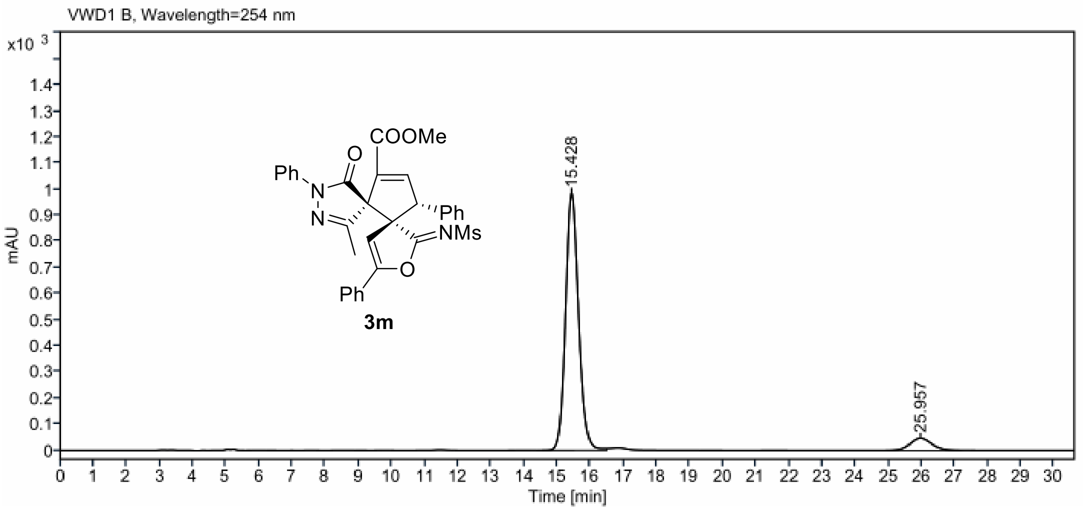

Signal: VWD1 B, Wavelength=254 nm

RT [min]	Type	Width [min]	Area	Height	Area%	Name
15.428	MM	0.4599	27192.8691	985.4598	92.7887	
25.957	MM	0.7627	2113.3542	46.1794	7.2113	
		Sum	29306.2234			

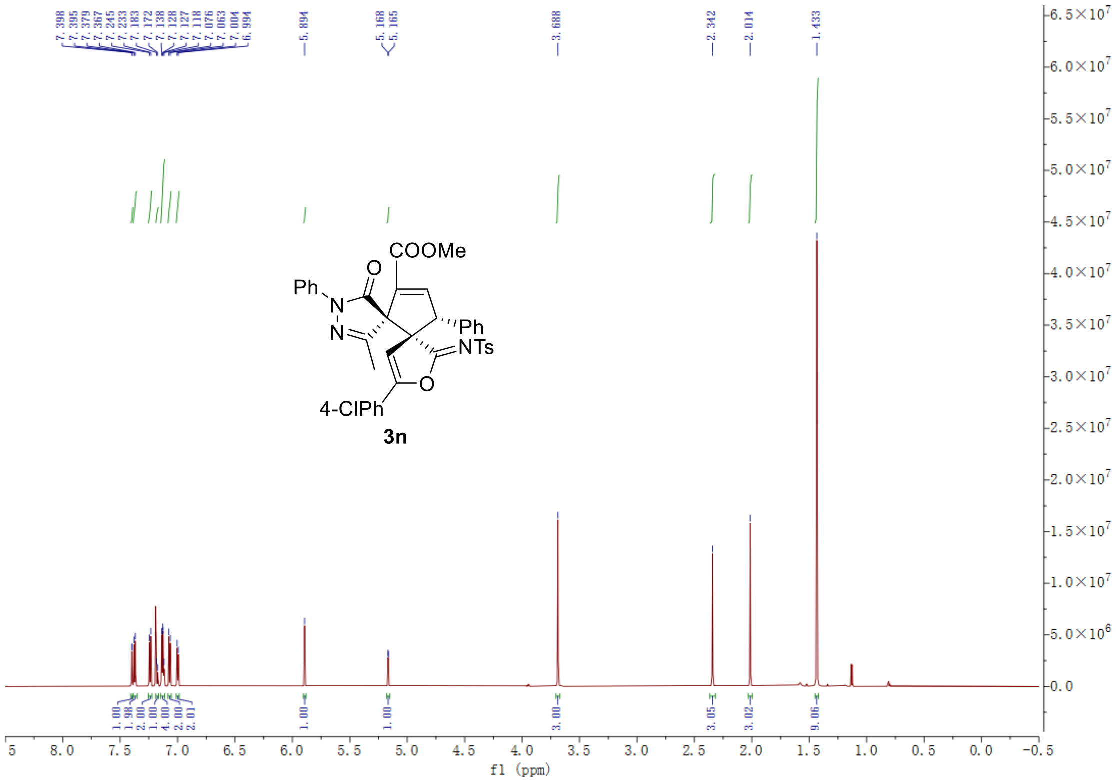
7.398
7.395
7.379
7.367
7.245
7.233
7.183
7.172
7.138
7.128
7.127
7.118
7.076
7.063
7.004
6.994
5.894
5.168
5.165
3.688
2.342
2.014
1.433
COOMe
O
Ph
N
N
Ph
NTs
O
4-ClPh
3n
1.00
1.98
2.00
1.00
1.00
4.00
2.00
2.01
1.00
1.00
3.00
3.05
3.02
9.06
f1 (ppm)

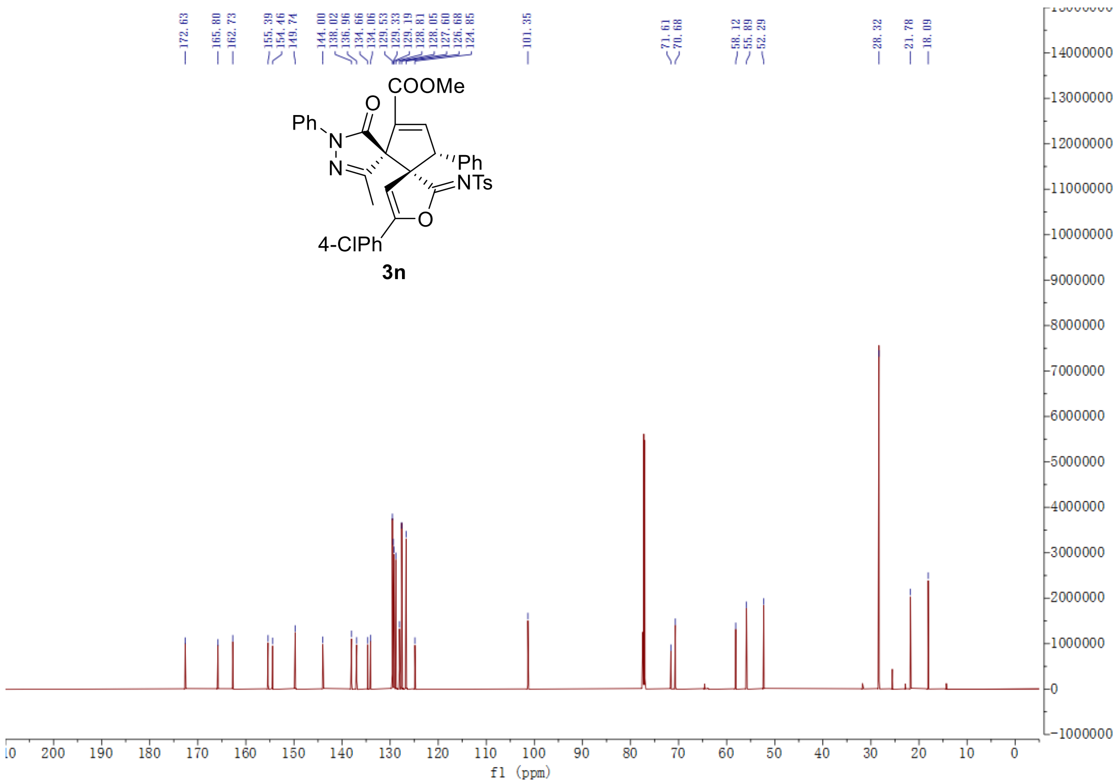
172.63
165.80
162.73
155.39
154.46
149.74
144.00
138.02
136.96
134.66
134.06
129.53
129.33
129.19
128.81
128.05
127.60
126.68
124.85
101.35
71.61
70.68
58.12
55.89
52.29
28.32
21.78
18.09
COOMe
O
Ph
N
N
Ph
NTs
O
4-ClPh
3n
f1 (ppm)

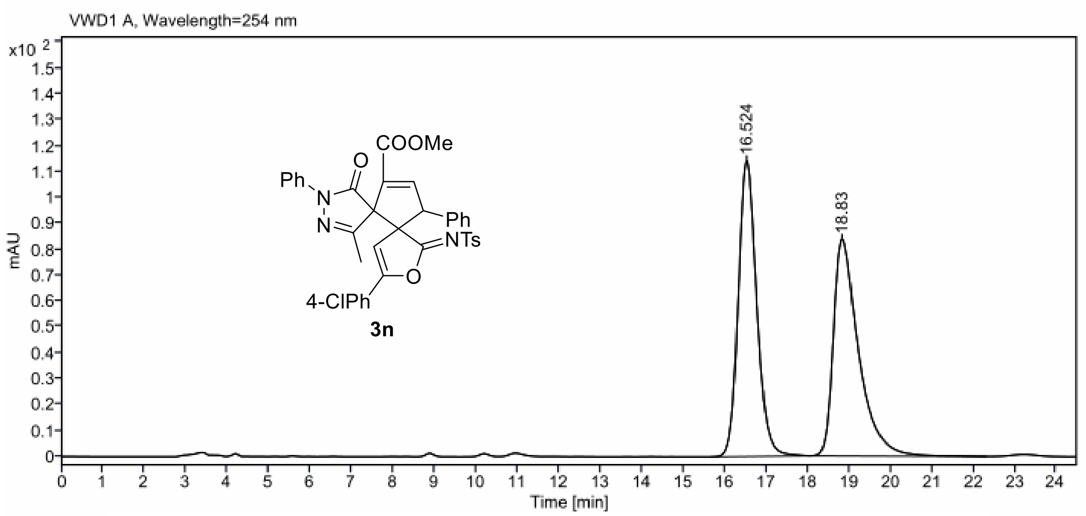

Signal: VWD1 A, Wavelength=254 nm

RT [min]	Type	Width [min]	Area	Height	Area%	Name
16.524	BB	0.4767	3552.6104	114.2804	50.1078	
18.830	BB	0.6275	3537.3184	83.6217	49.8922	
		Sum	7089.9287			

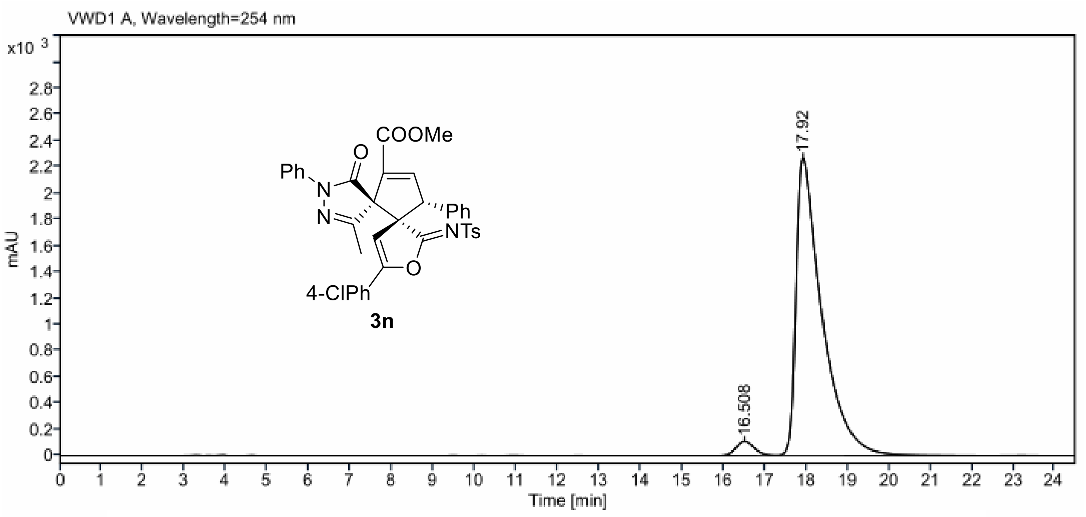

Signal: VWD1 A, Wavelength=254 nm

RT [min]	Type	Width [min]	Area	Height	Area%	Name
16.508	MM	0.5061	3240.5046	106.7060	3.1902	
17.920	MM	0.7224	98337.6016	2268.7041	96.8098	
		Sum	101578.1062			

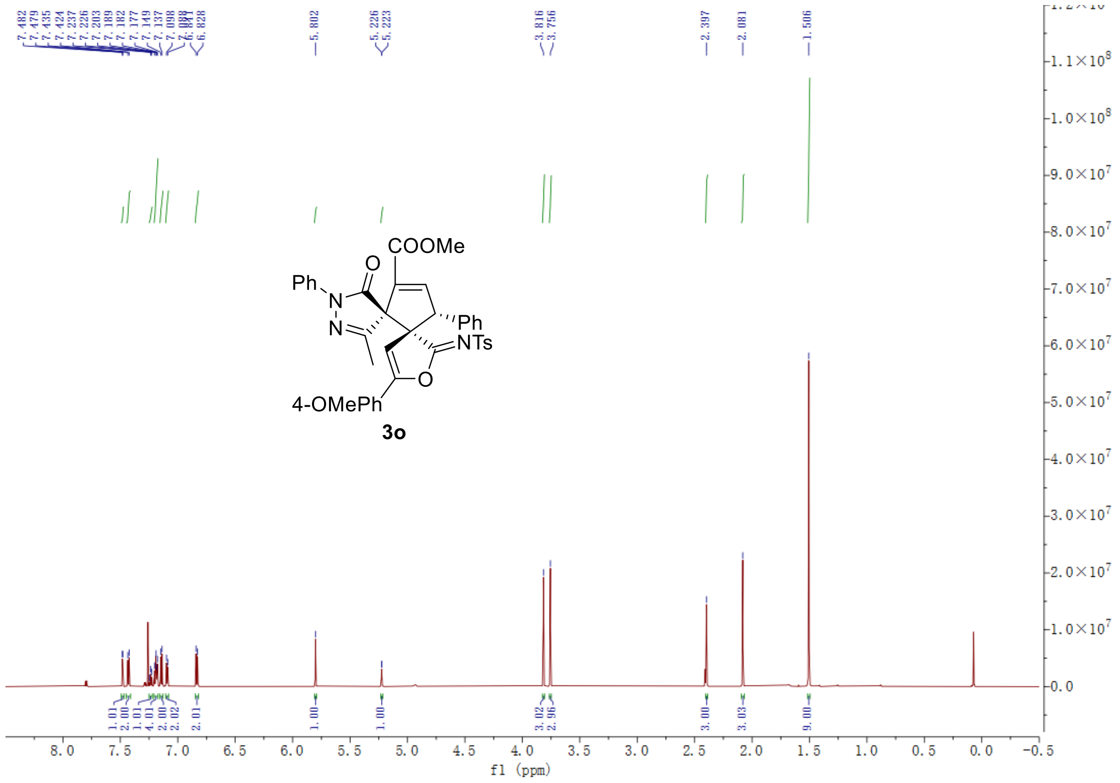

7.482
7.479
7.435
7.424
7.237
7.226
7.203
7.189
7.182
7.177
7.149
7.137
6.828
5.802
5.226
5.223
3.816
3.756
2.397
2.081
1.506
COOMe
O
Ph
N
N
Ph
NTs
O
4-OMePh
3o
1.01
2.00
1.01
1.01
4.01
2.00
2.02
2.01
1.00
1.00
3.02
2.96
3.00
3.03
9.00
f1 (ppm)

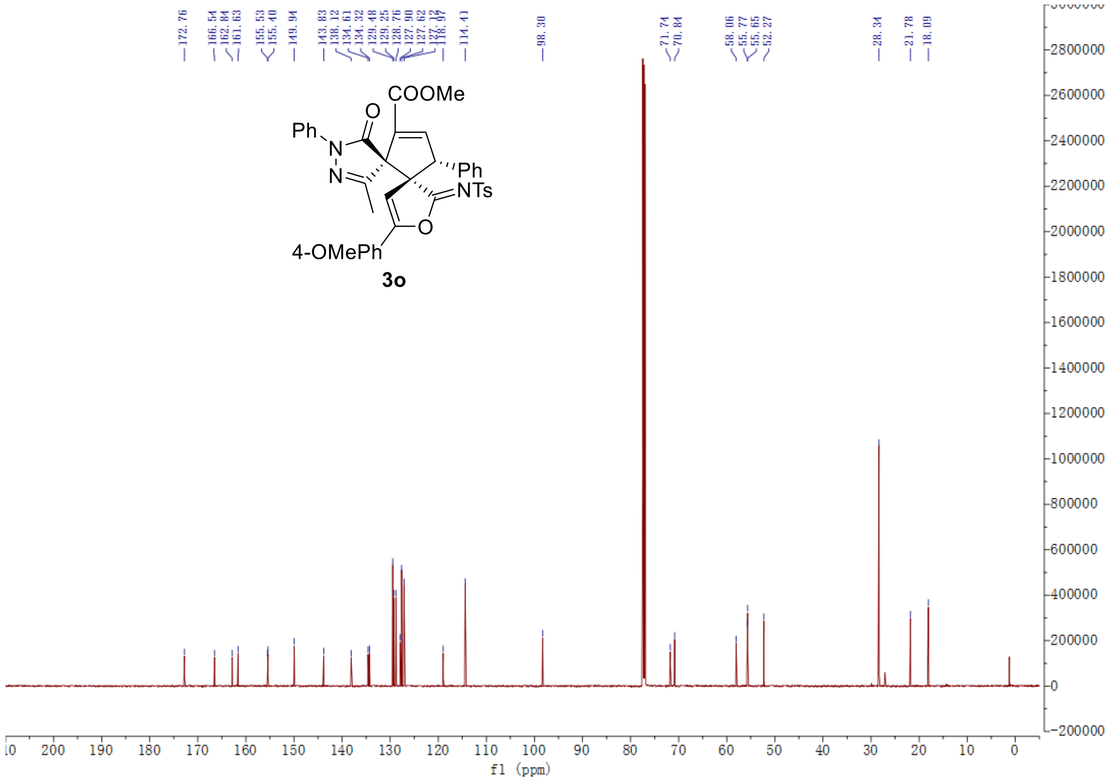

172.76
166.54
162.84
161.63
155.53
155.40
149.94
143.83
138.12
134.61
134.32
129.48
129.25
128.76
127.90
127.62
118.97
114.41
98.30
71.74
70.84
58.06
55.77
55.65
52.27
28.34
21.78
18.09
COOMe
O
Ph
N
N
Ph
NTs
O
4-OMePh
3o
f1 (ppm)

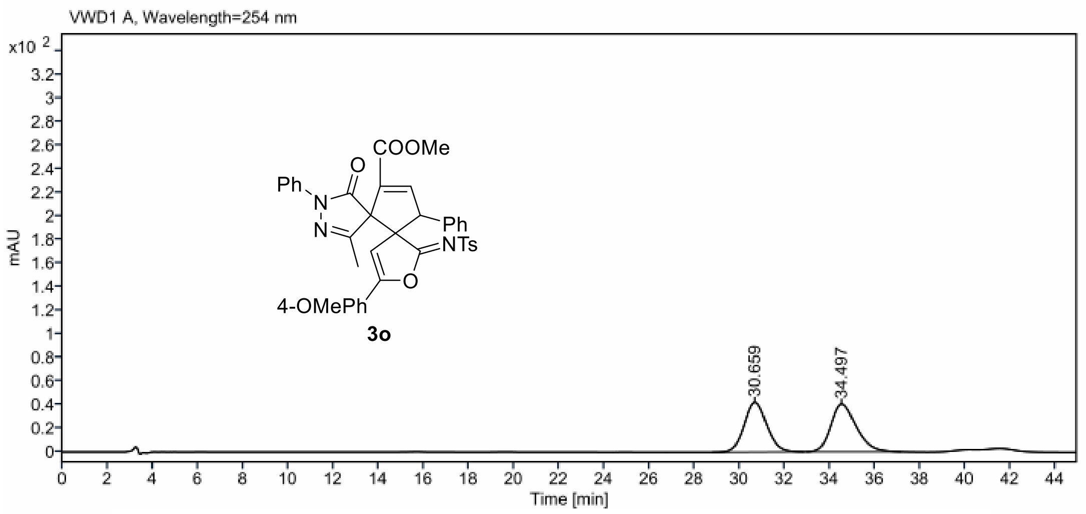

Signal: VWD1 A, Wavelength=254 nm

RT [min]	Type	Width [min]	Area	Height	Area%	Name
30.659	BB	1.0837	2941.6187	41.9714	48.1717	
34.497	MM	1.3093	3164.9048	40.2872	51.8283	
		Sum	6106.5234			

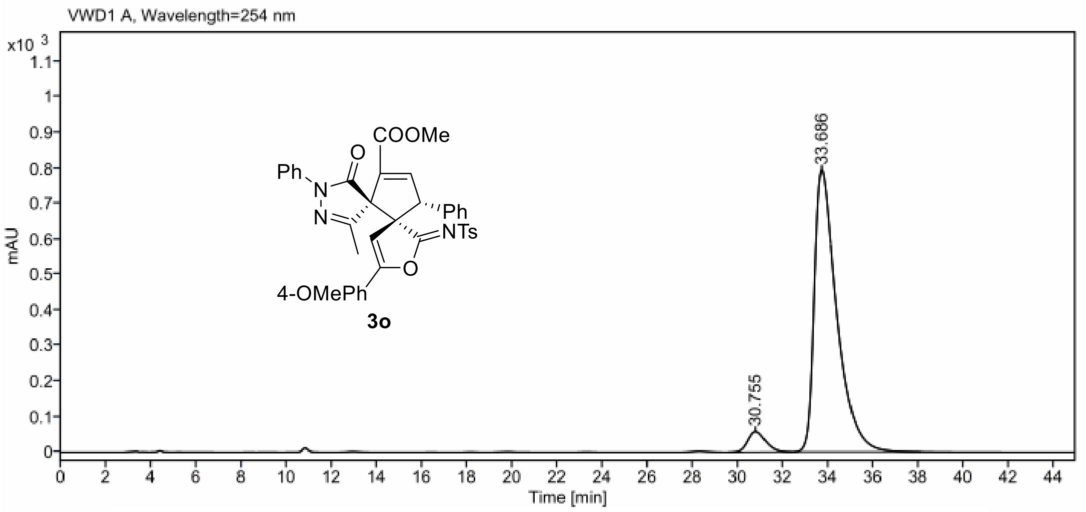

Signal: VWD1 A, Wavelength=254 nm

RT [min]	Type	Width [min]	Area	Height	Area%	Name
30.755	MM	0.9284	3236.8923	58.1084	5.5709	
33.686	MM	1.1538	54866.4883	792.5590	94.4291	
		Sum	58103.3806			

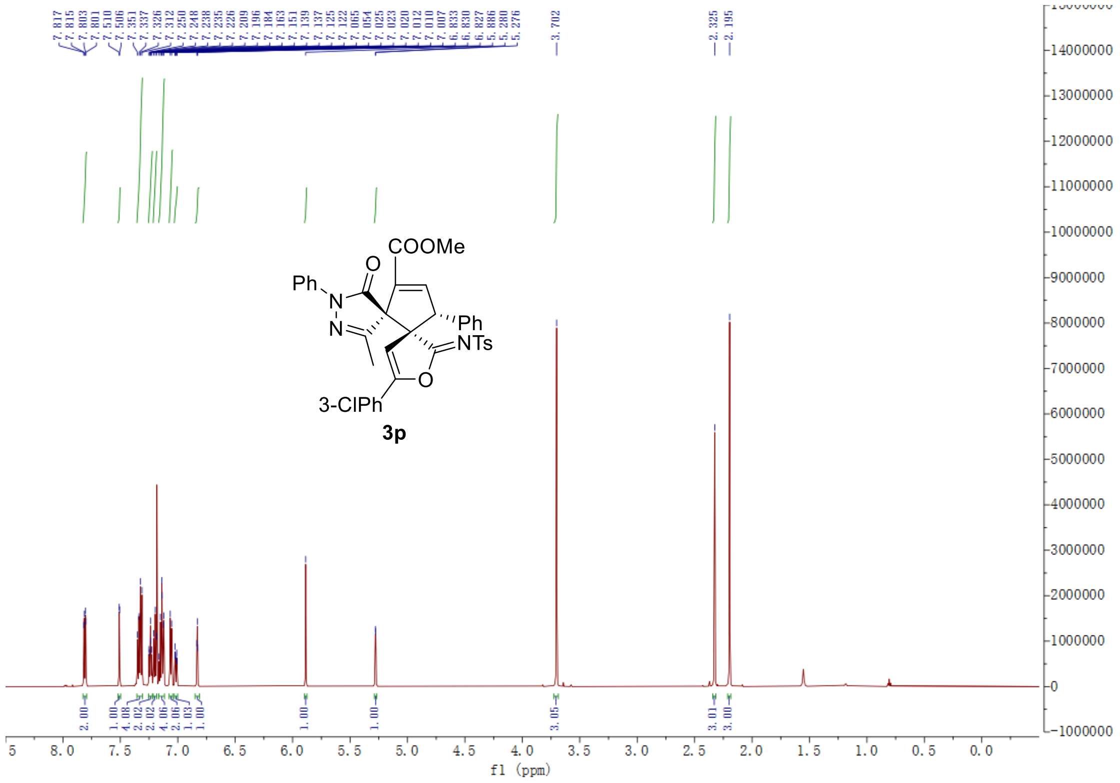
7.817 7.815 7.803 7.801 7.510 7.506 7.351 7.337 7.326 7.312 7.250 7.248 7.238 7.235 7.226 7.209 7.196 7.184 7.163 7.151 7.139 7.137 7.125 7.122 7.065 7.054 7.025 7.023 7.020 7.012 7.010 7.007 6.833 6.830 6.827 5.286 5.280 5.276
3.702
2.325 2.195
COOMe
O
Ph
N
N
Ph
NTs
O
3-ClPh
3p
2.00 1.00 4.08 2.02 2.02 4.06 2.06 1.03 1.00 1.00 1.00 3.05 3.01 3.00
f1 (ppm)

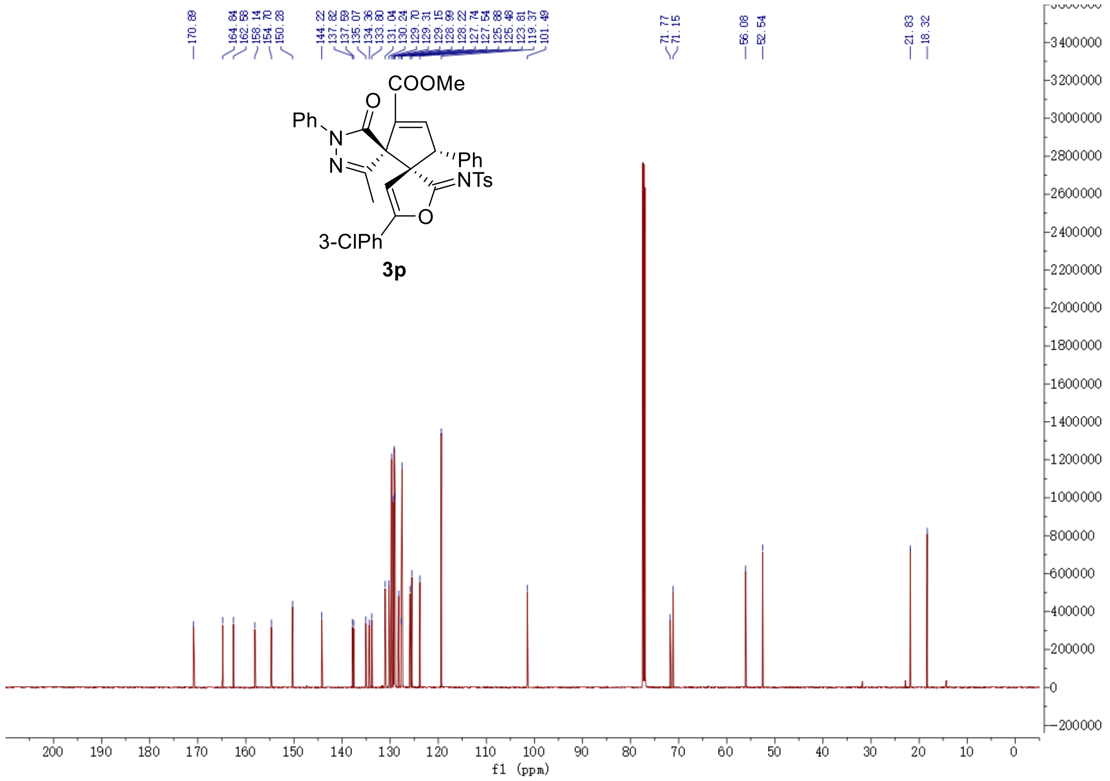
170.89
164.84 162.58 158.14 154.70 150.28
144.22 137.82 137.59 135.07 134.36 133.80 131.04 130.24 129.70 129.31 129.15 128.99 128.22 127.74 127.54 125.88 125.48 123.81 119.37 101.49
71.77 71.15
56.08 52.54
21.83 18.32
COOMe
O
Ph
N
N
Ph
NTs
O
3-ClPh
3p
f1 (ppm)

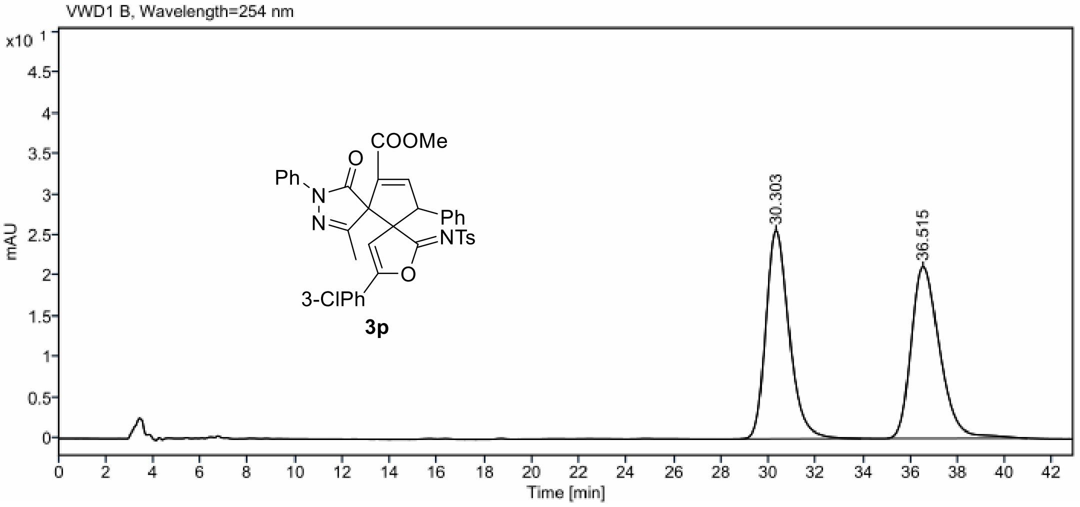

Signal: VWD1 B, Wavelength=254 nm

RT [min]	Type	Width [min]	Area	Height	Area%	Name
30.303	MM	1.1443	1759.1243	25.6218	49.8587	
36.515	MM	1.3961	1769.0967	21.1191	50.1413	
		Sum	3528.2209			

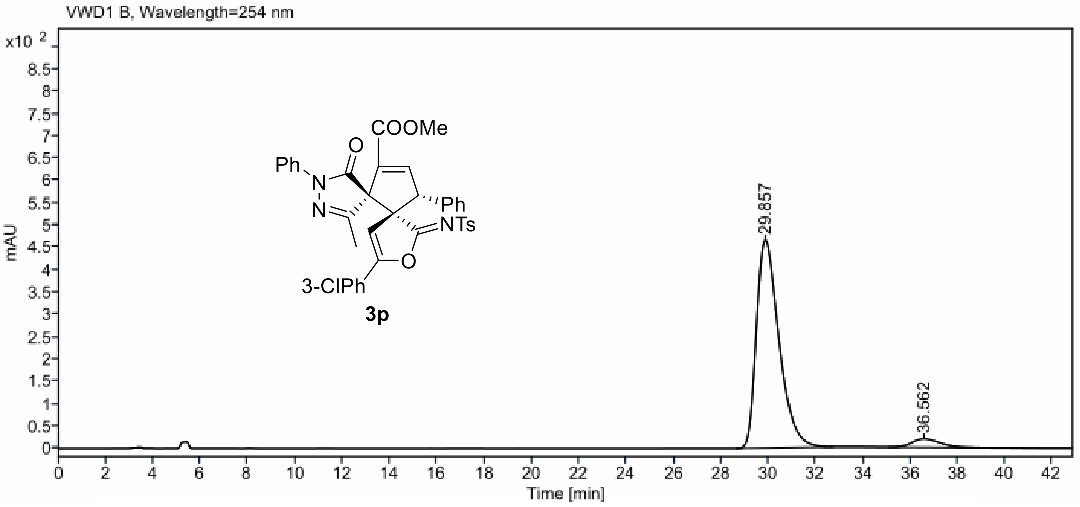

Signal: VWD1 B, Wavelength=254 nm

RT [min]	Type	Width [min]	Area	Height	Area%	Name
29.857	BB	1.0127	30857.1934	465.9211	95.2531	
36.562	BBA	1.2595	1537.7601	18.9340	4.7469	
		Sum	32394.9535			

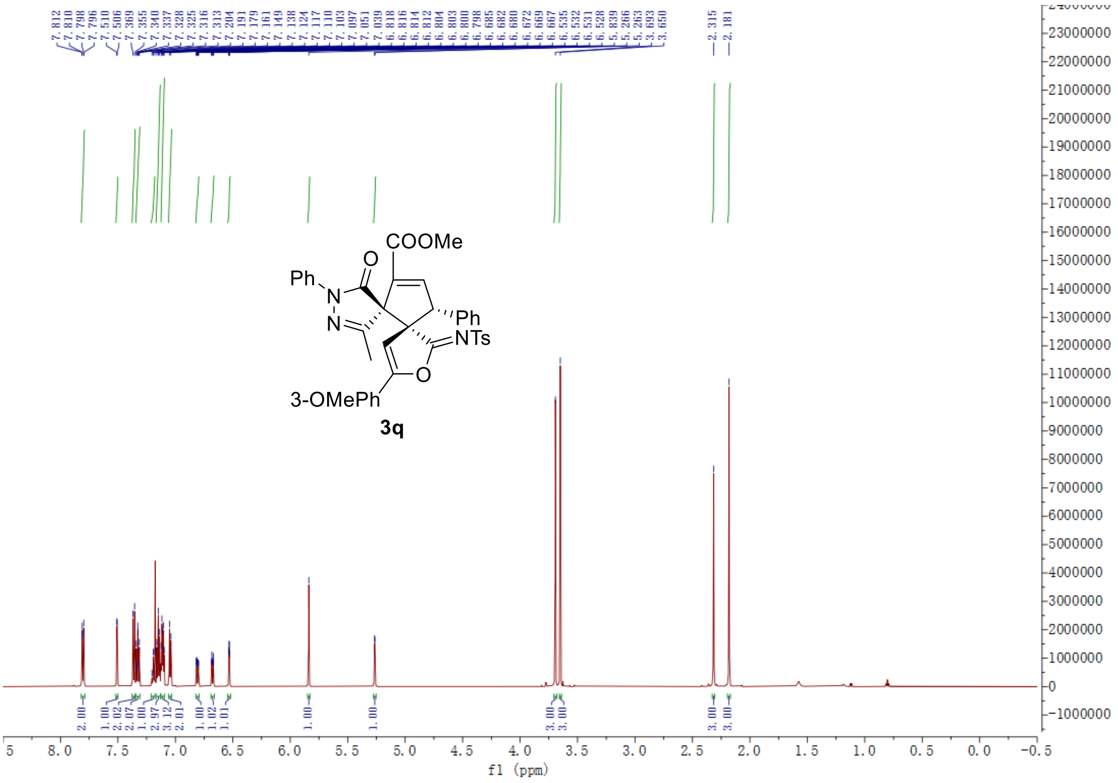
COOMe
Ph
N
N
O
Ph
NTs
O
3-OMePh
3q
f1 (ppm)

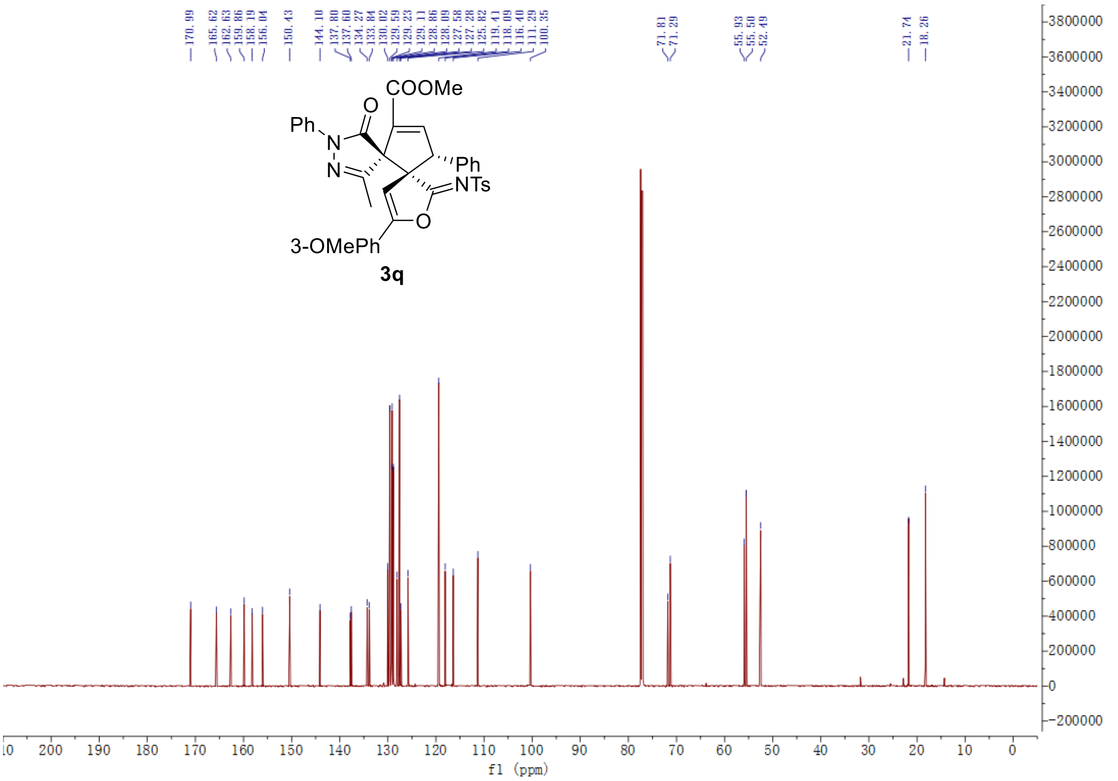
COOMe
Ph
N
N
O
Ph
NTs
O
3-OMePh
3q
f1 (ppm)

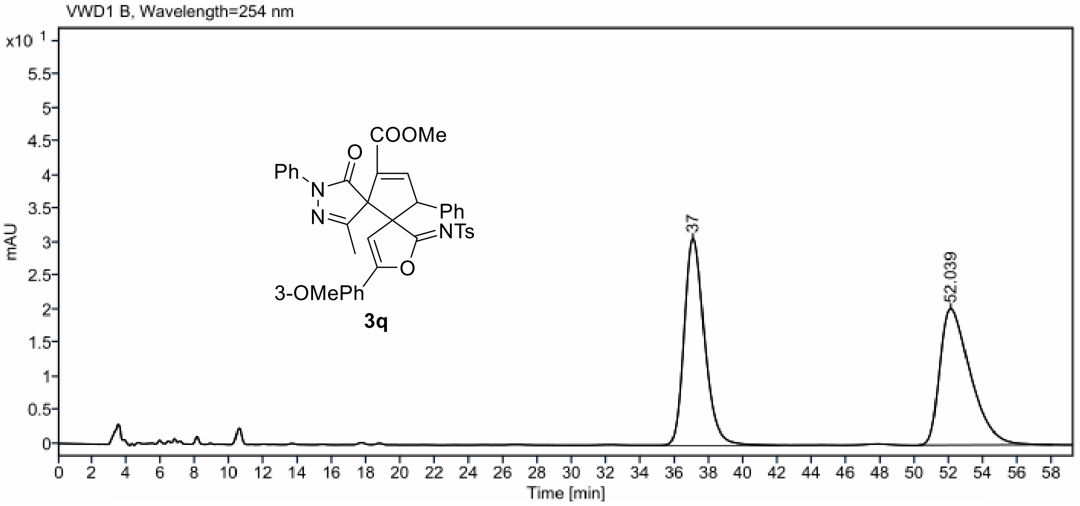

Signal: VWD1 B, Wavelength=254 nm

RT [min]	Type	Width [min]	Area	Height	Area%	Name
37.000	BB	1.2608	2540.4326	30.8464	50.0453	
52.039	MM	2.0794	2535.8311	20.3252	49.9547	
		Sum	5076.2637			

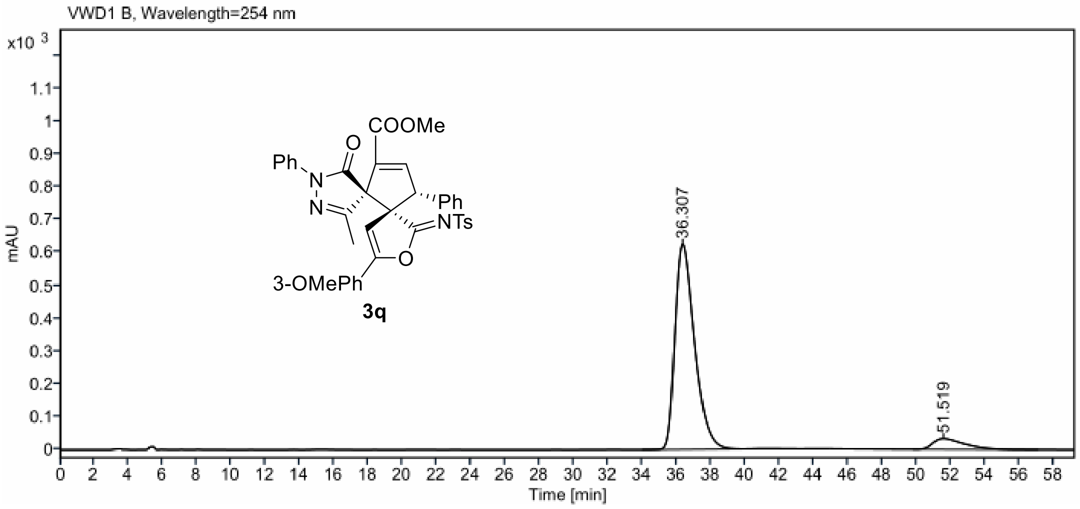

Signal: VWD1 B, Wavelength=254 nm

RT [min]	Type	Width [min]	Area	Height	Area%	Name
36.307	BB	1.2262	49810.2148	624.7051	92.3478	
51.519	BBA	1.8802	4127.4204	32.6186	7.6522	
		Sum	53937.6353			

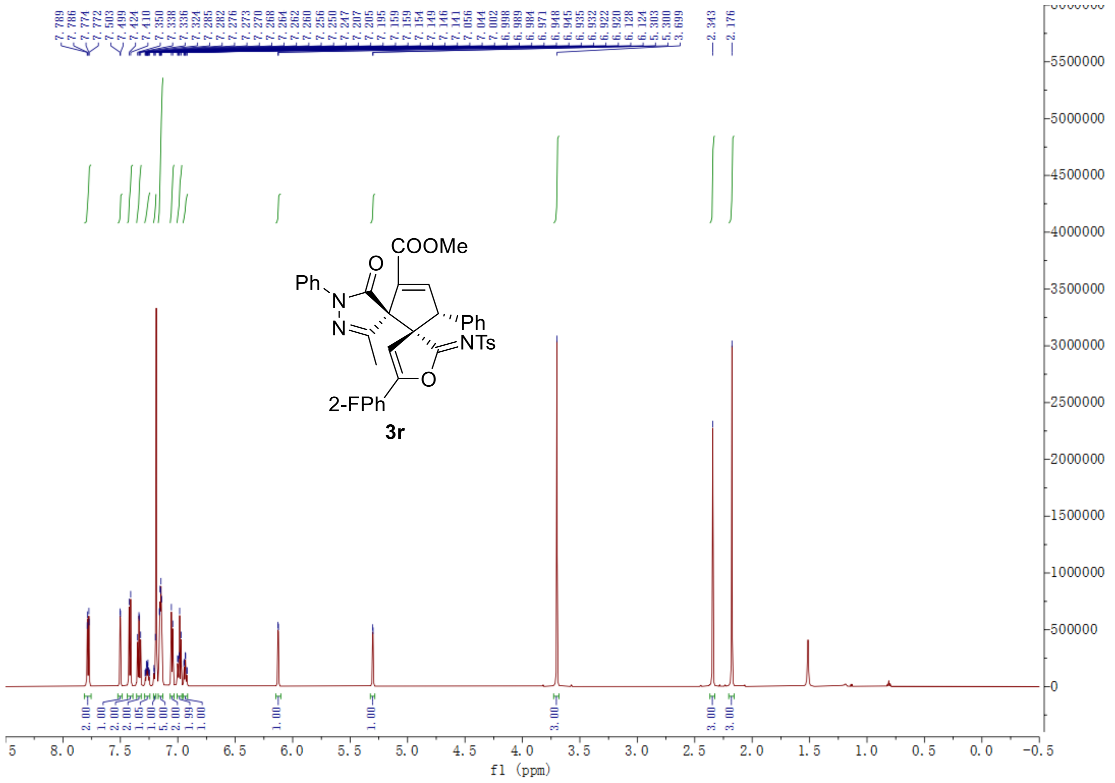

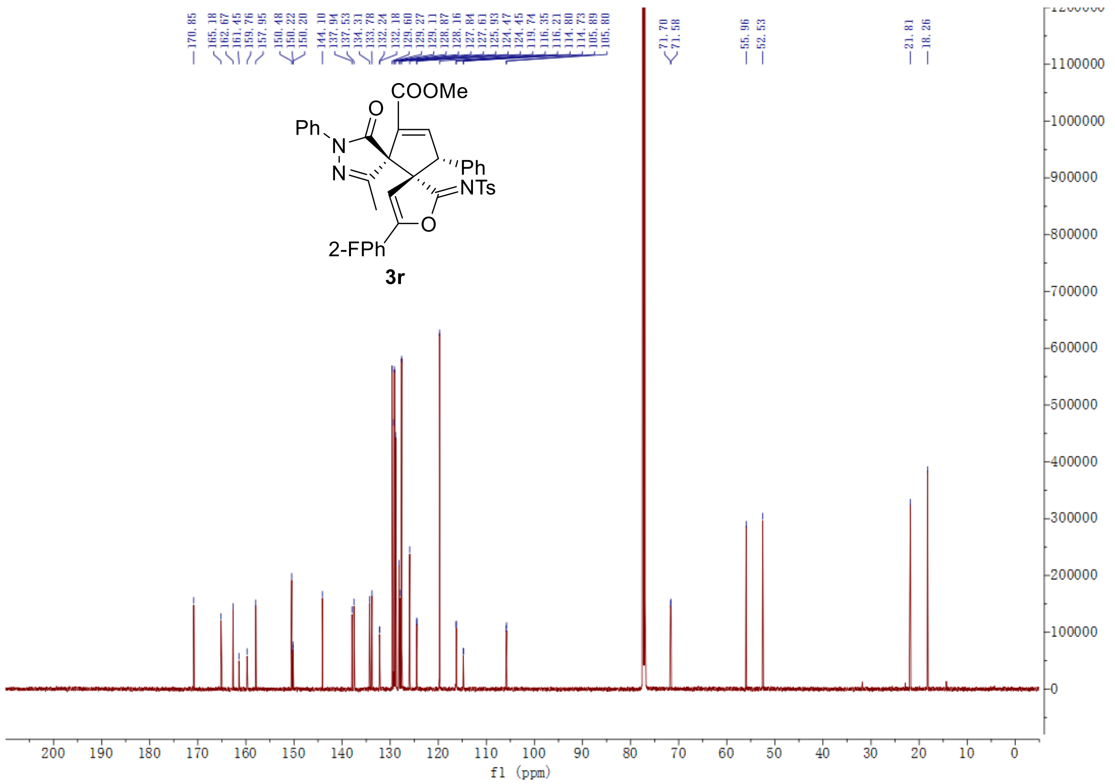

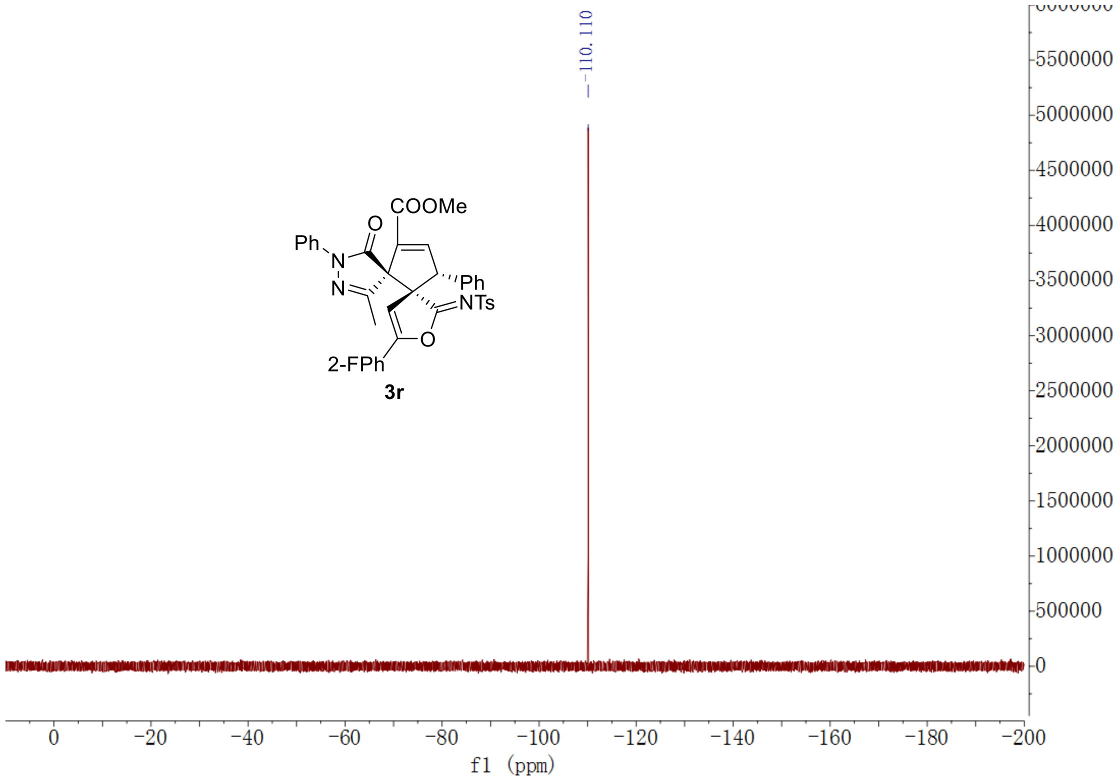

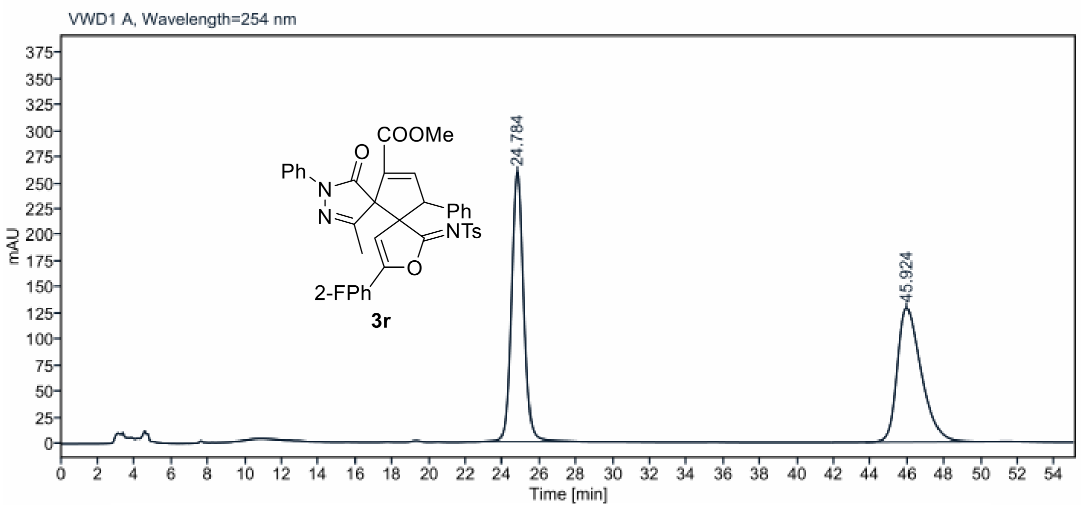

Signal: VWD1 A, Wavelength=254 nm

RT [min]	Type	Width [min]	Area	Height	Area%	Name
24.784	MM	0.7631	11857.4932	258.9883	50.4629	
45.924	MM	1.5075	11639.9736	128.6919	49.5371	
		Sum	23497.4668			

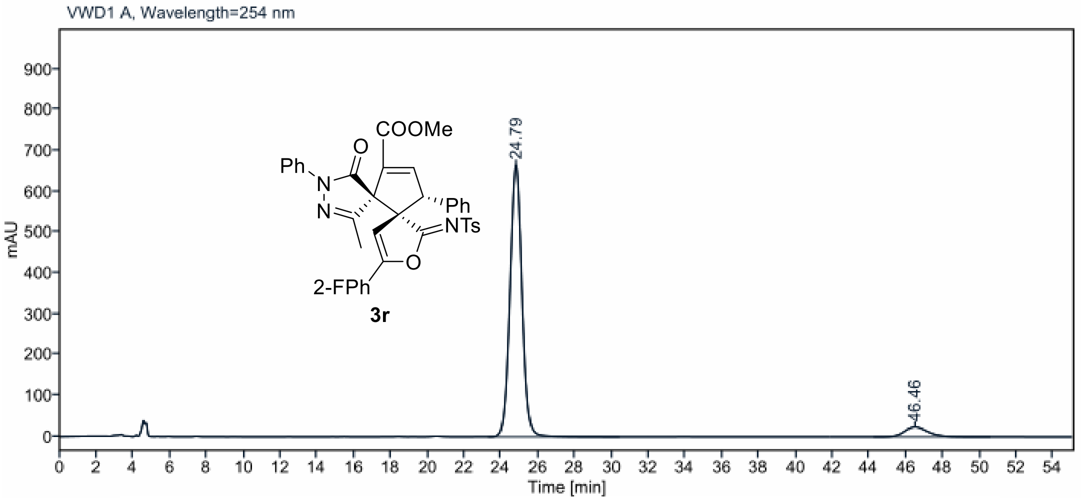

Signal: VWD1 A, Wavelength=254 nm

RT [min]	Type	Width [min]	Area	Height	Area%	Name
24.790	BB	0.7099	30442.3965	665.2166	93.5365	
46.460	BB	1.3159	2103.6213	23.7609	6.4635	
		Sum	32546.0178			

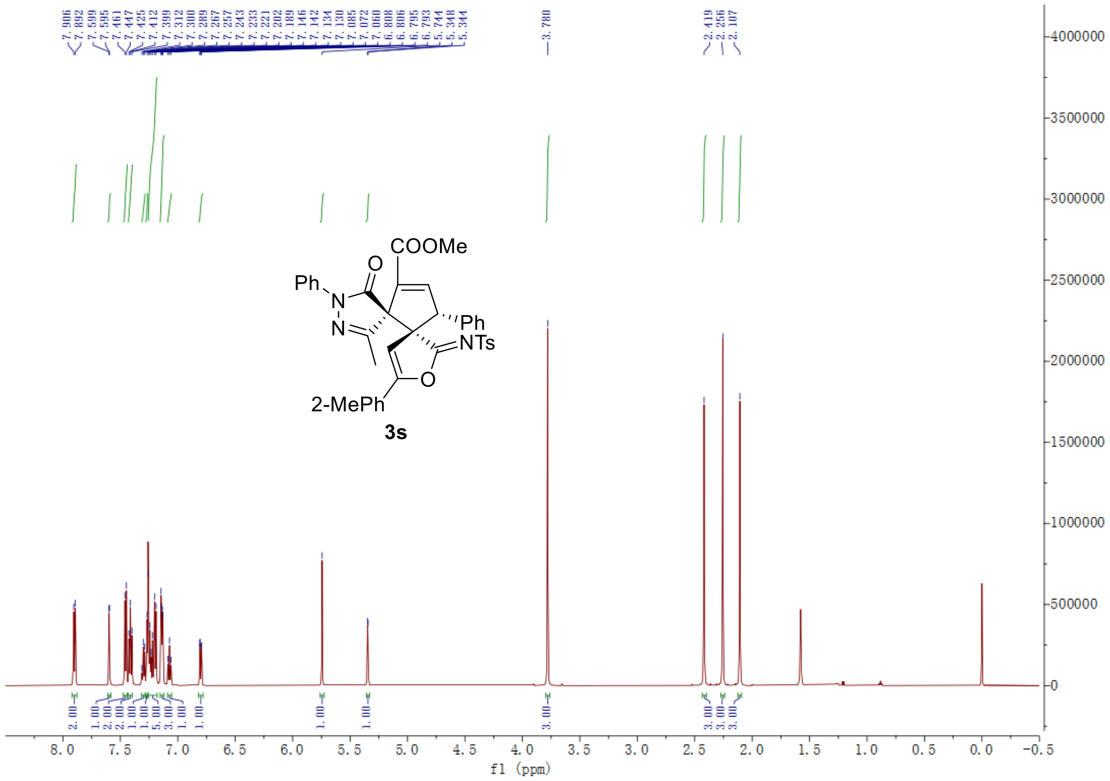

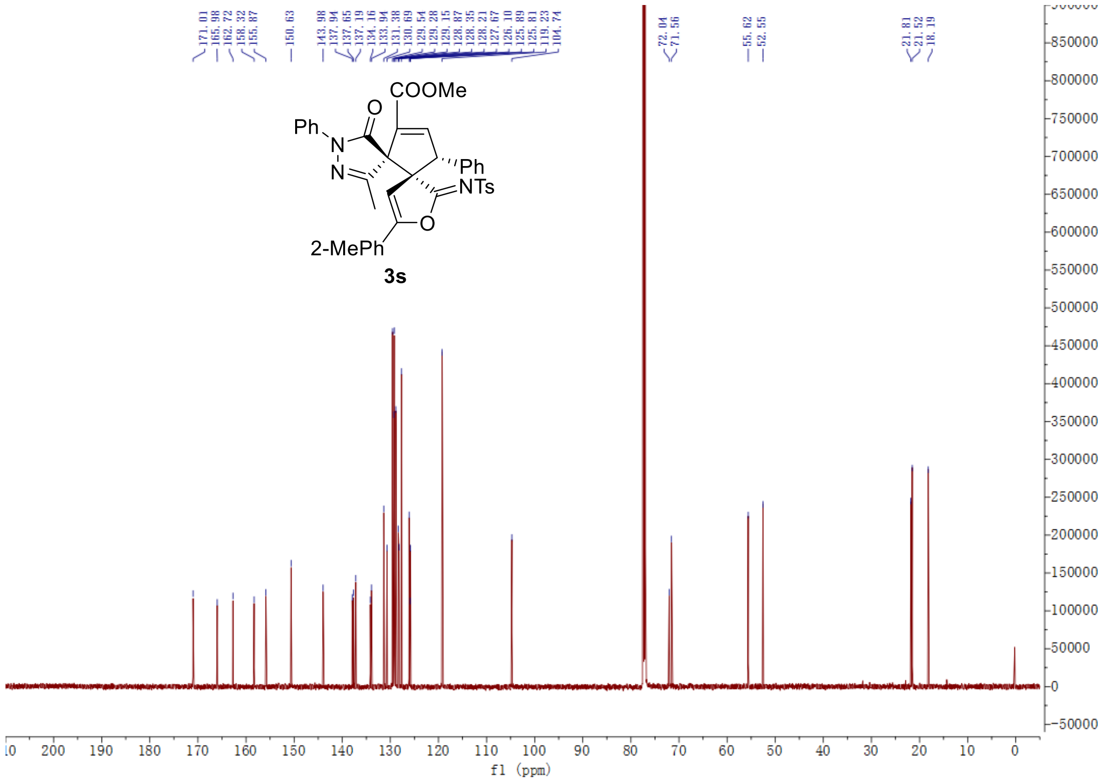

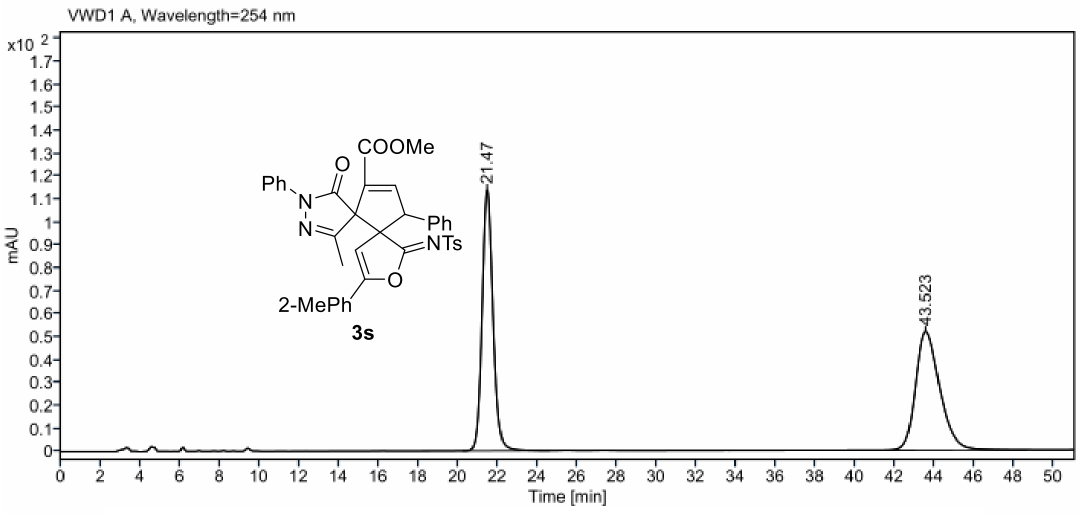

Signal: VWD1 A, Wavelength=254 nm

RT [min]	Type	Width [min]	Area	Height	Area%	Name
21.470	BB	0.5924	4399.2964	113.9513	50.1472	
43.523	BB	1.2827	4373.4644	51.7106	49.8528	
		Sum	8772.7607			

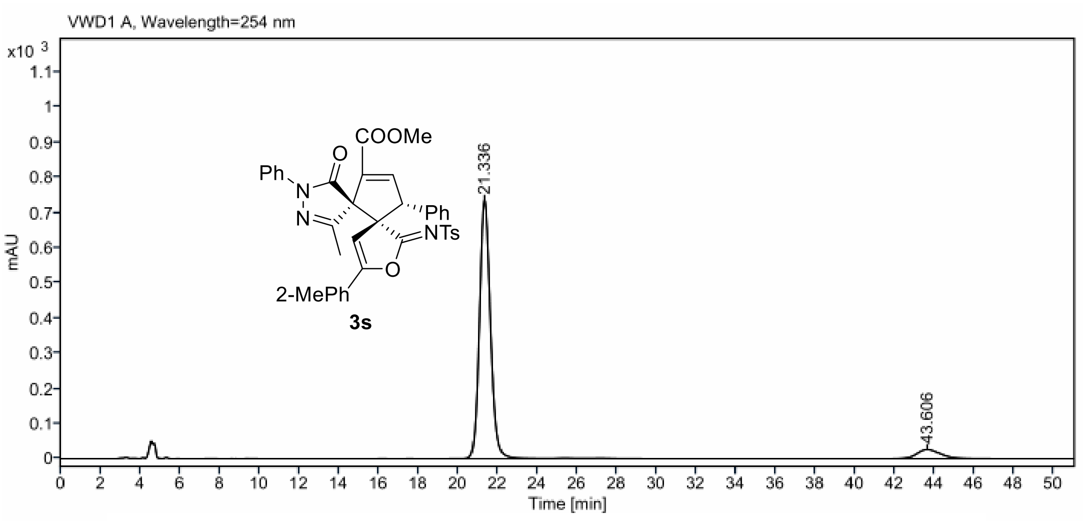

Signal: VWD1 A, Wavelength=254 nm

RT [min]	Type	Width [min]	Area	Height	Area%	Name
21.336	BB	0.5988	28553.7793	735.6356	92.7926	
43.606	MM	1.3946	2217.8367	26.5047	7.2074	
		Sum	30771.6160			

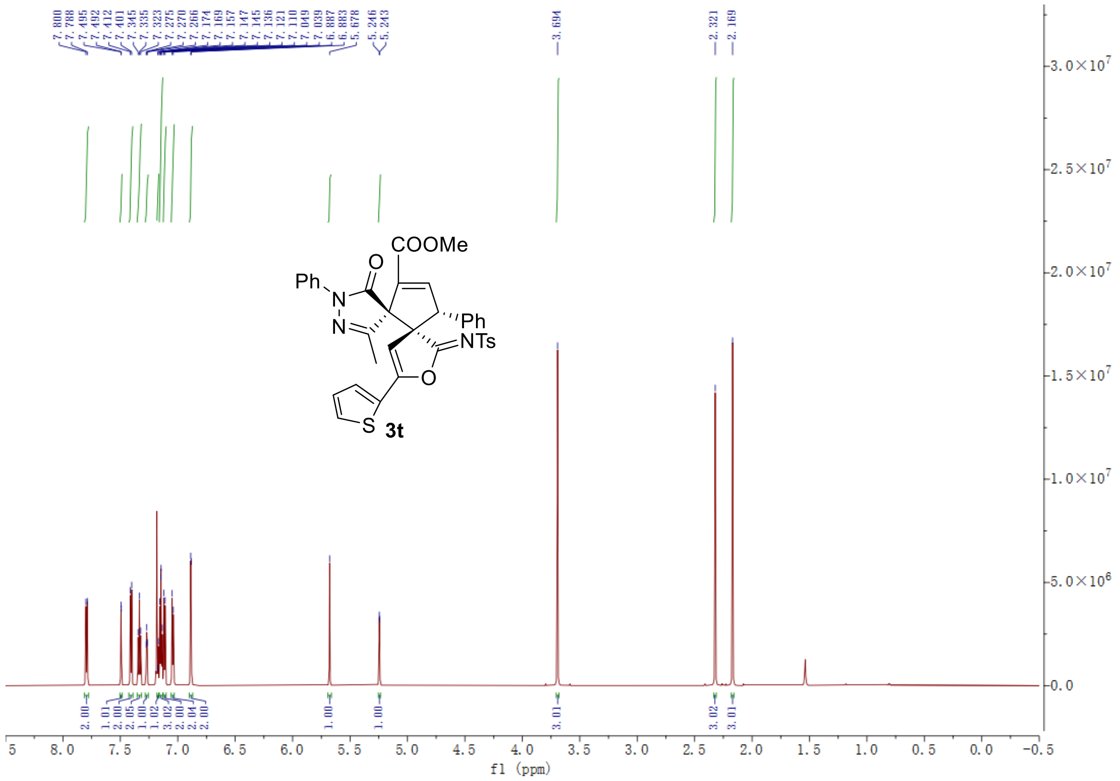

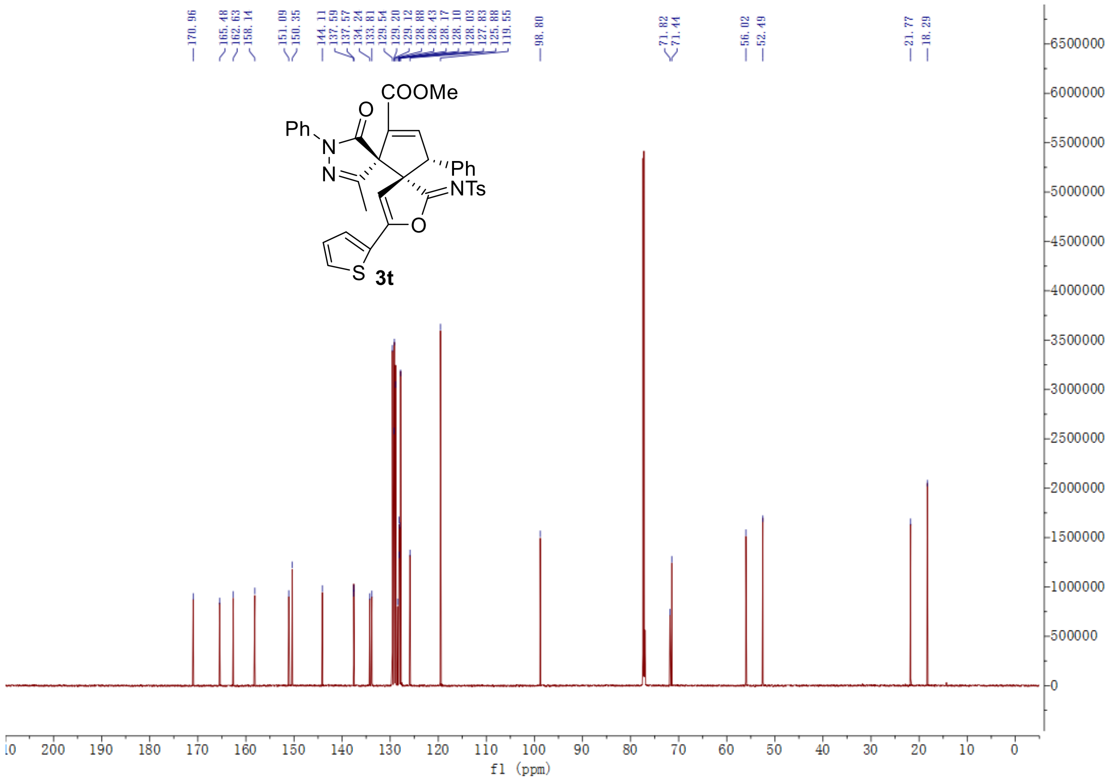

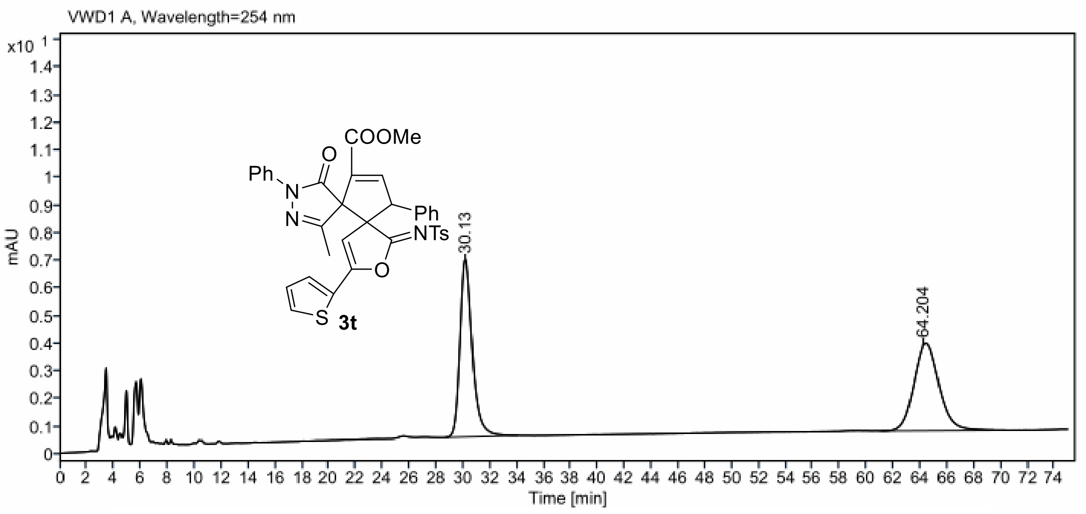

Signal: VWD1 A, Wavelength=254 nm

RT [min]	Type	Width [min]	Area	Height	Area%	Name
30.130	BB	0.9648	411.5309	6.4287	49.9884	
64.204	MM	2.1684	411.7217	3.1645	50.0116	
		Sum	823.2525			

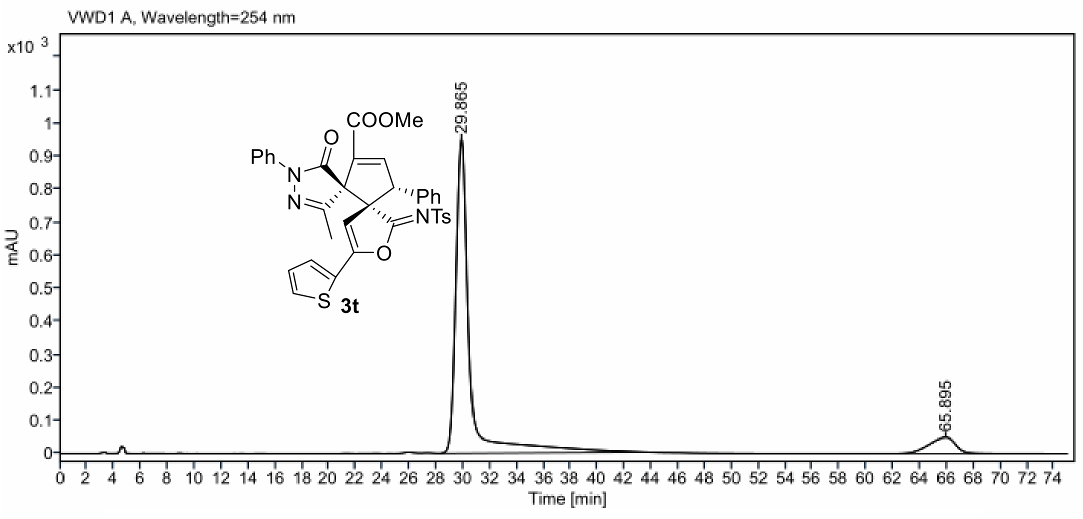

Signal: VWD1 A, Wavelength=254 nm

RT [min]	Type	Width [min]	Area	Height	Area%	Name
29.865	MM	1.2027	68701.2734	952.0609	91.1746	
65.895	MM	2.2632	6650.0513	48.9713	8.8254	
		Sum	75351.3247			

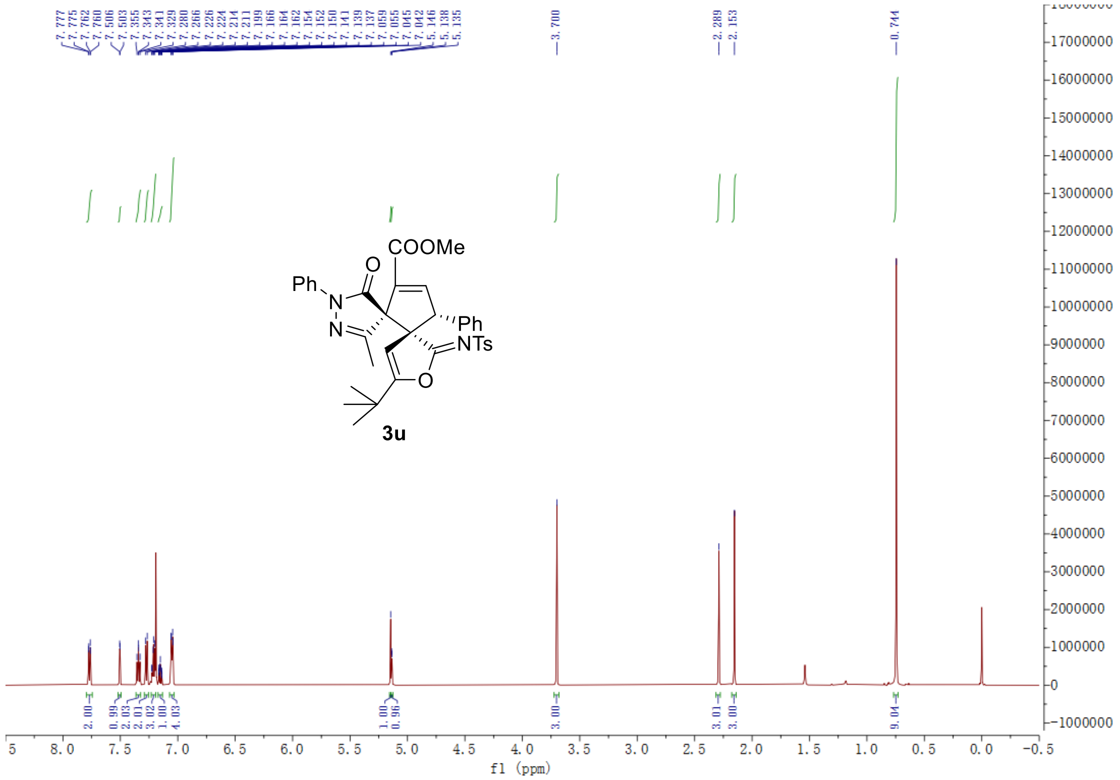

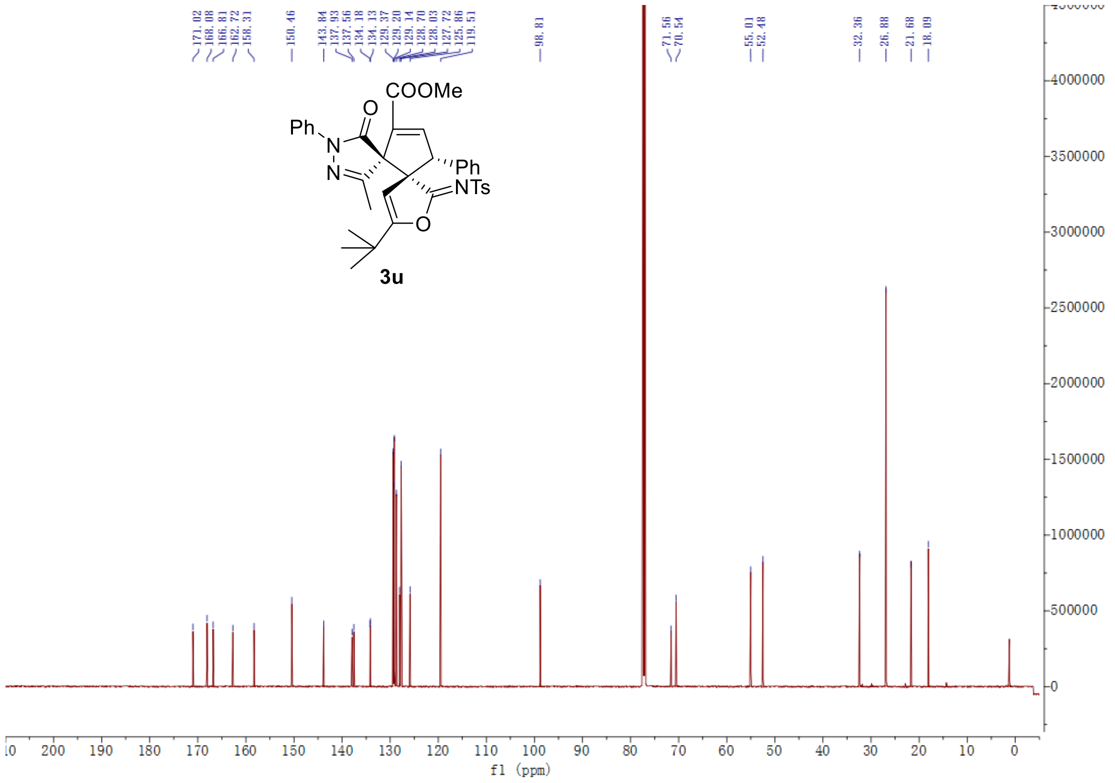

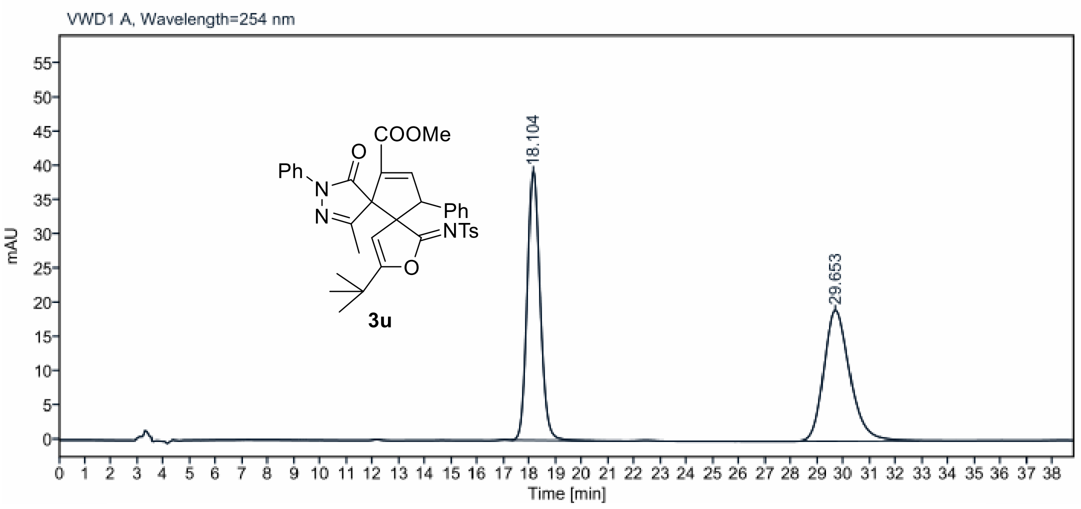

Signal:	VWD1 A, Wavelength=254 nm				
RT [min] Type	Width [min]	Area	Height	Area%	Name
18.104 BB	0.5234	1332.3268	39.2113	50.2210	
29.653 BB	1.0364	1320.5984	19.0811	49.7790	
	Sum	2652.9252			

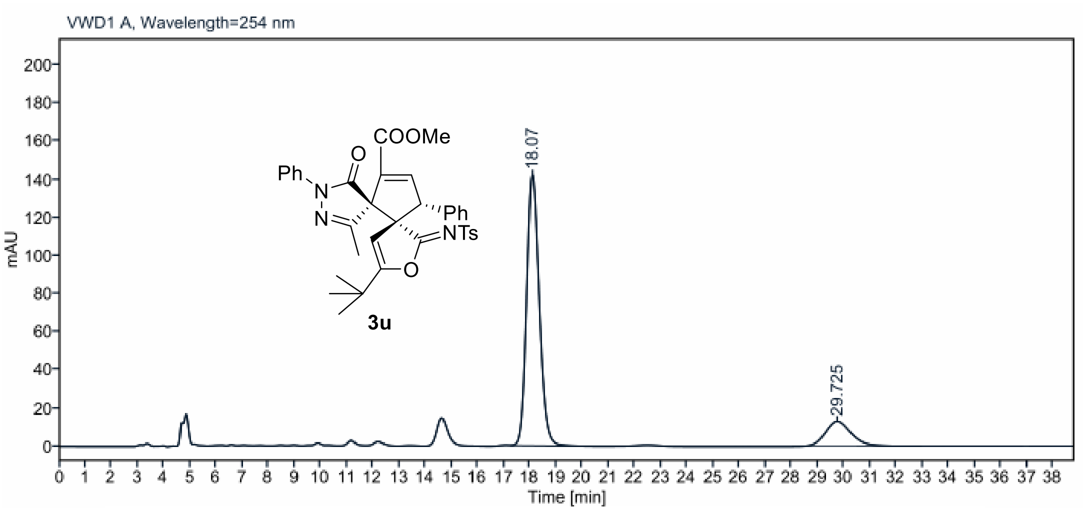

Signal: VWD1 A, Wavelength=254 nm

RT [min]	Type	Width [min]	Area	Height	Area%	Name
18.070	MM	0.5551	4733.2808	142.1118	83.6918	
29.725	BB	1.0593	922.3278	13.1185	16.3082	
		Sum	5655.6086			

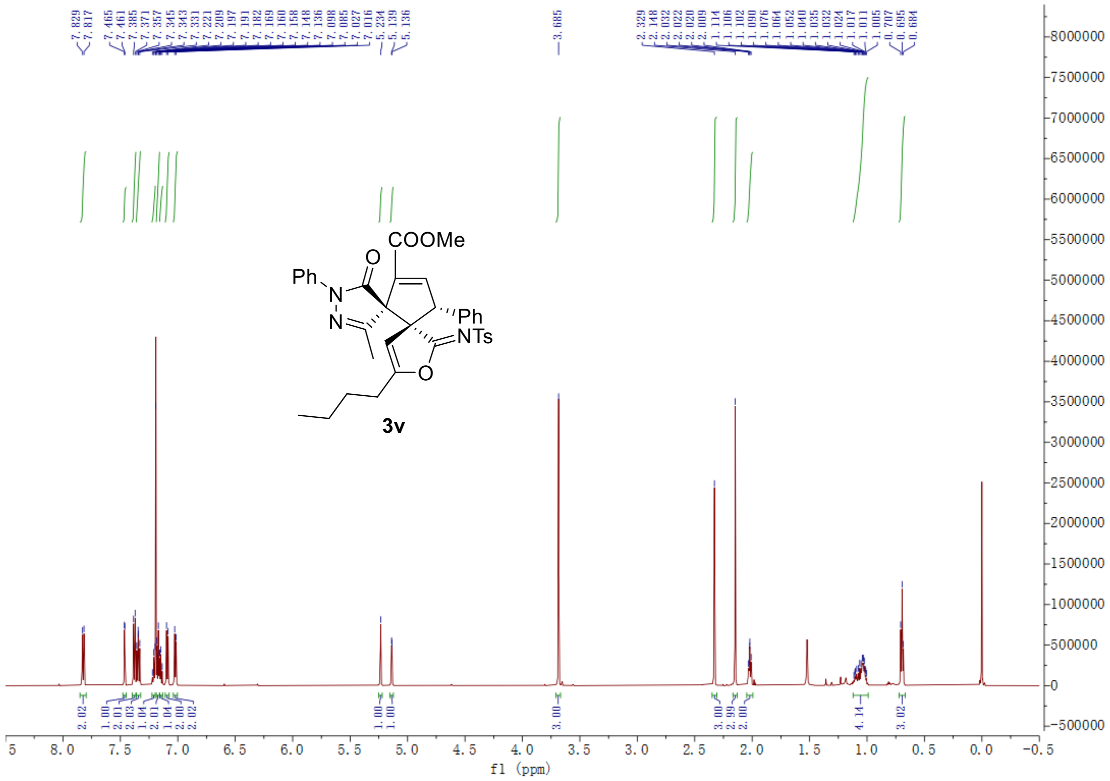

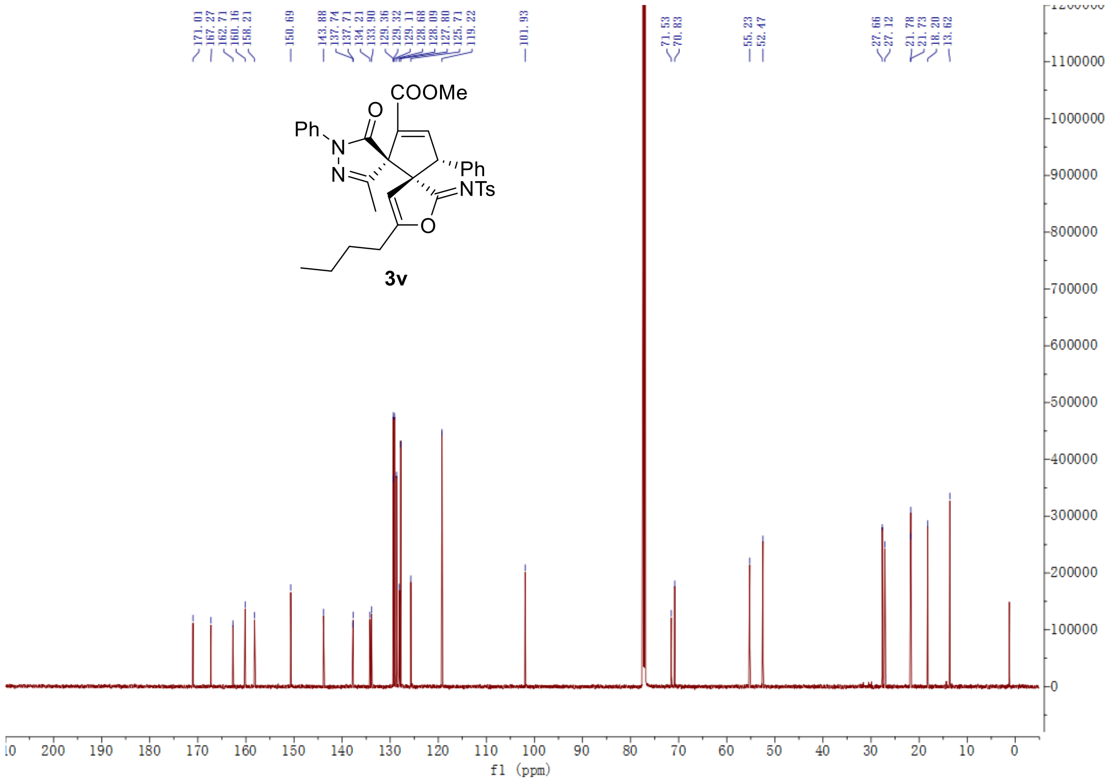

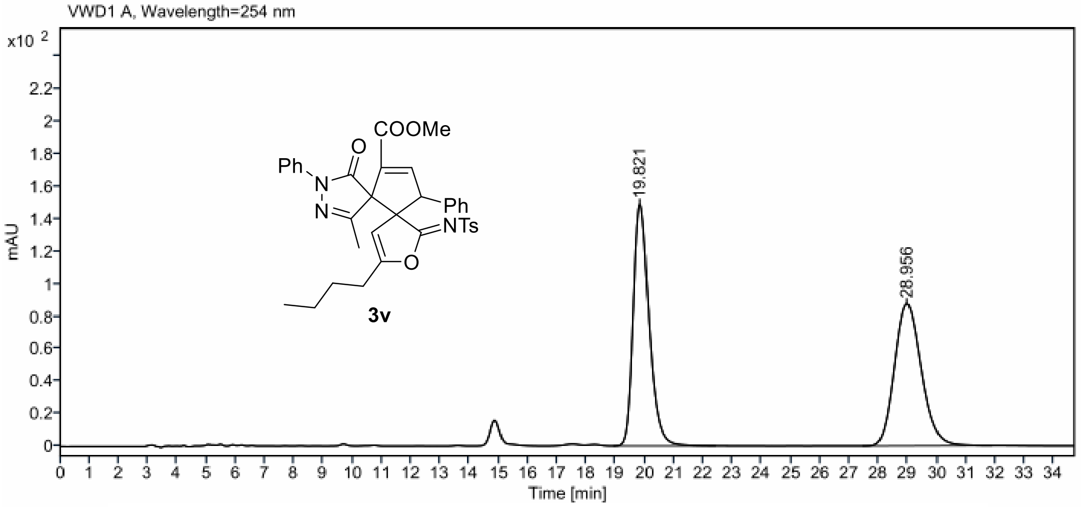

Signal: VWD1 A, Wavelength=254 nm

RT [min]	Type	Width [min]	Area	Height	Area%	Name
19.821	MM	0.6301	5615.2842	148.5261	50.1572	
28.956	MM	1.0650	5580.0967	87.3260	49.8428	
		Sum	11195.3809			

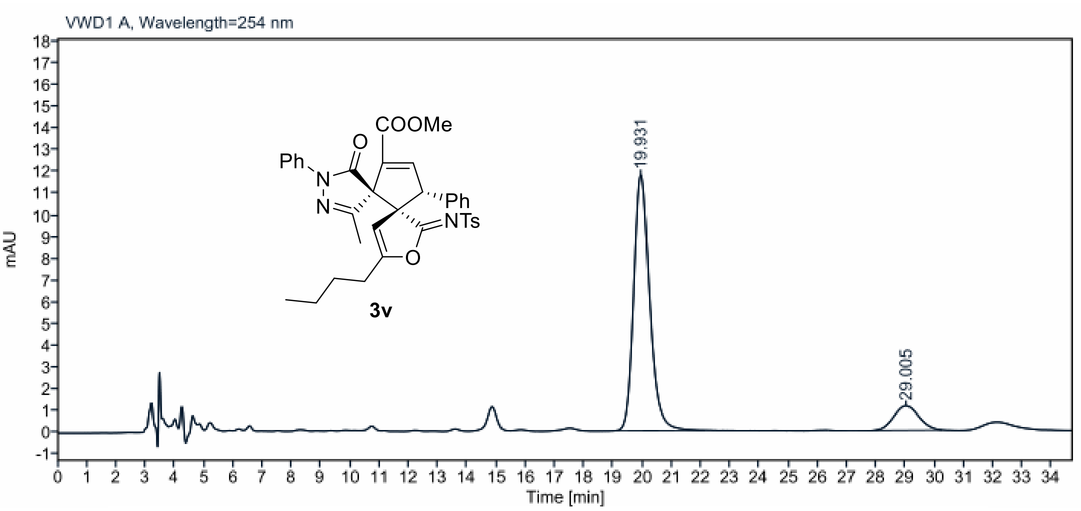

Signal: VWD1 A, Wavelength=254 nm

RT [min]	Type	Width [min]	Area	Height	Area%	Name
19.931	BB	0.5789	447.3774	11.7581	86.8747	
29.005	MM	0.9963	67.5914	1.1307	13.1253	
		Sum	514.9688			

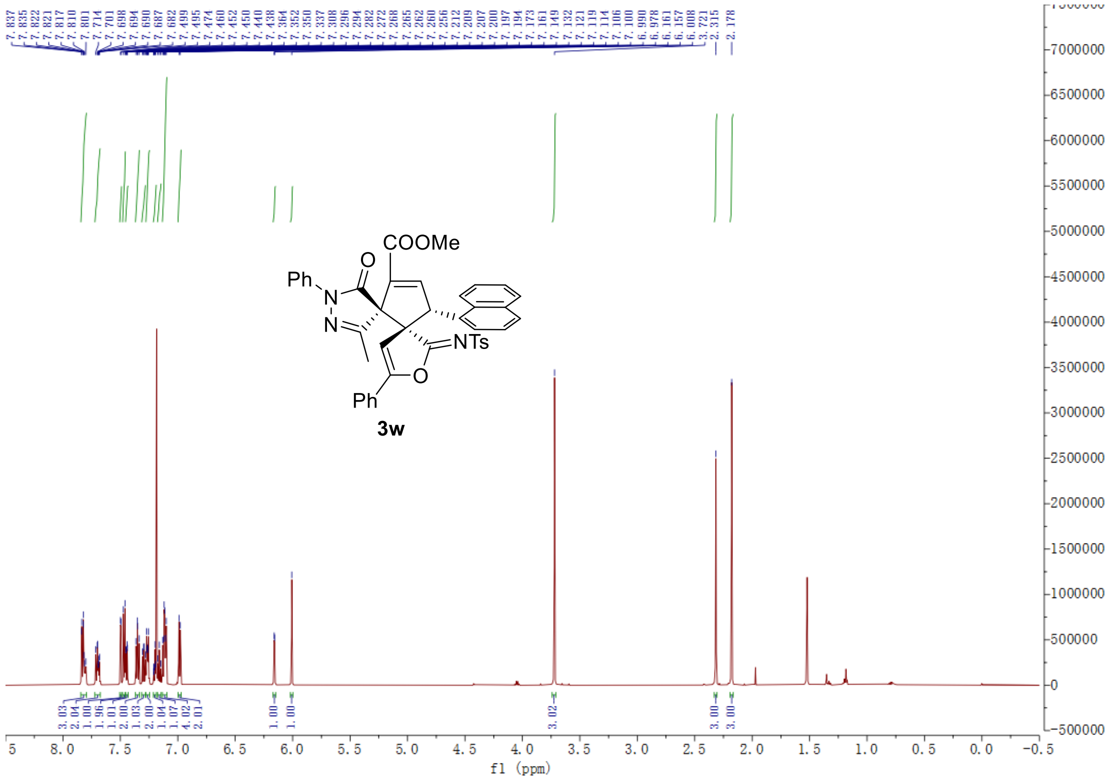

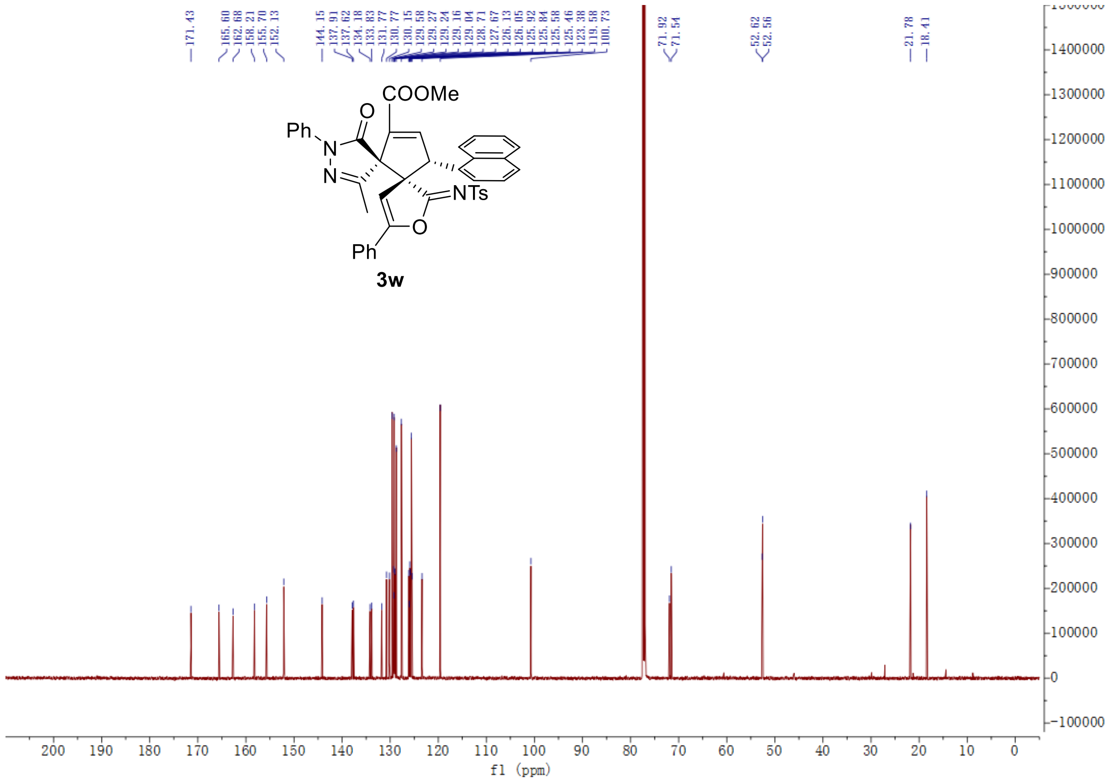

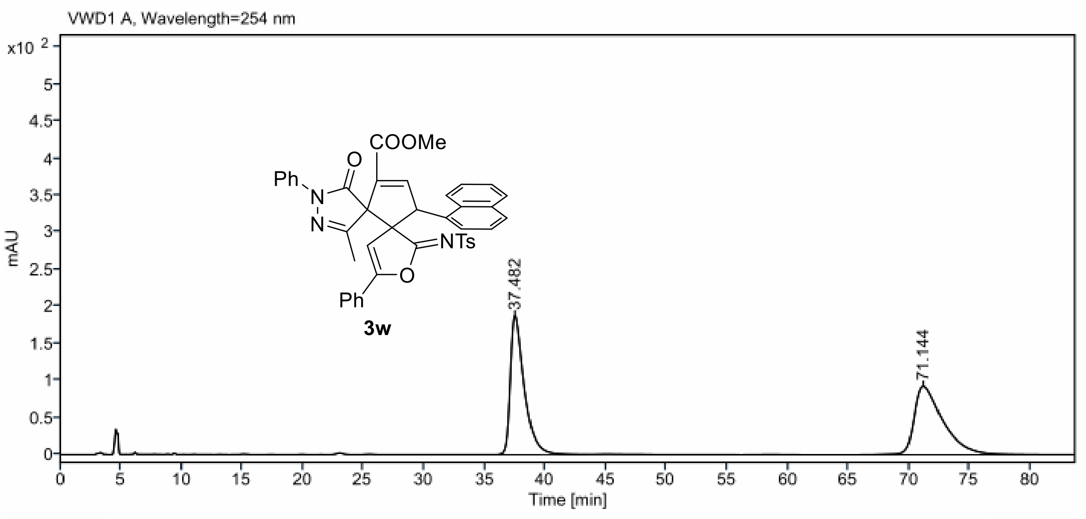

Signal: VWD1 A, Wavelength=254 nm

RT [min]	Type	Width [min]	Area	Height	Area%	Name
37.482	BB	1.1968	15198.4844	187.6736	50.0049	
71.144	MM	2.7395	15195.4893	92.4454	49.9951	
		Sum	30393.9736			

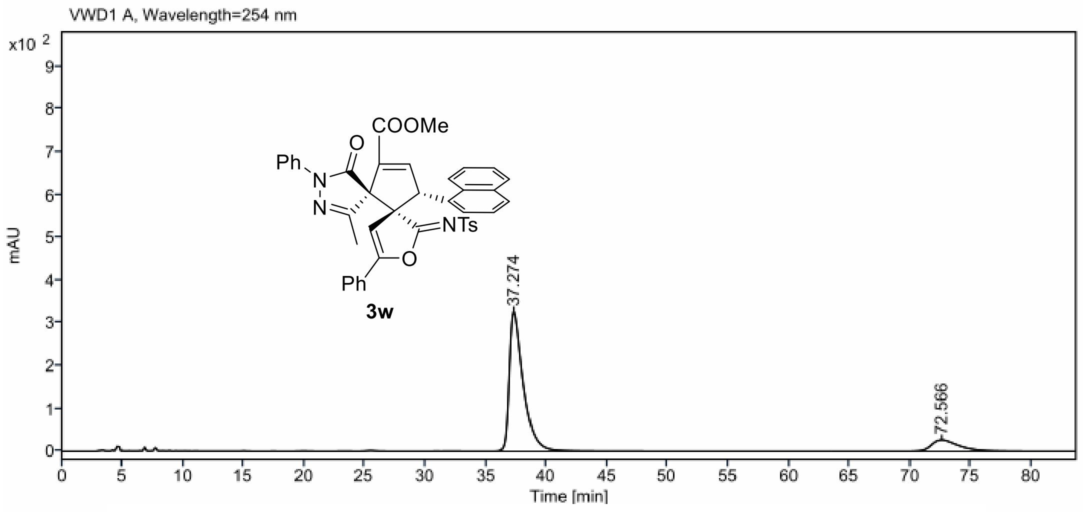

**Signal:**	VWD1 A, Wavelength=254 nm					
**RT [min]**	**Type**	**Width [min]**	**Area**	**Height**	**Area%**	**Name**
37.274	BB	1.1943	26511.4707	326.1614	86.3247	
72.566	MM	2.6409	4199.8794	26.5054	13.6753	
		Sum	30711.3501			

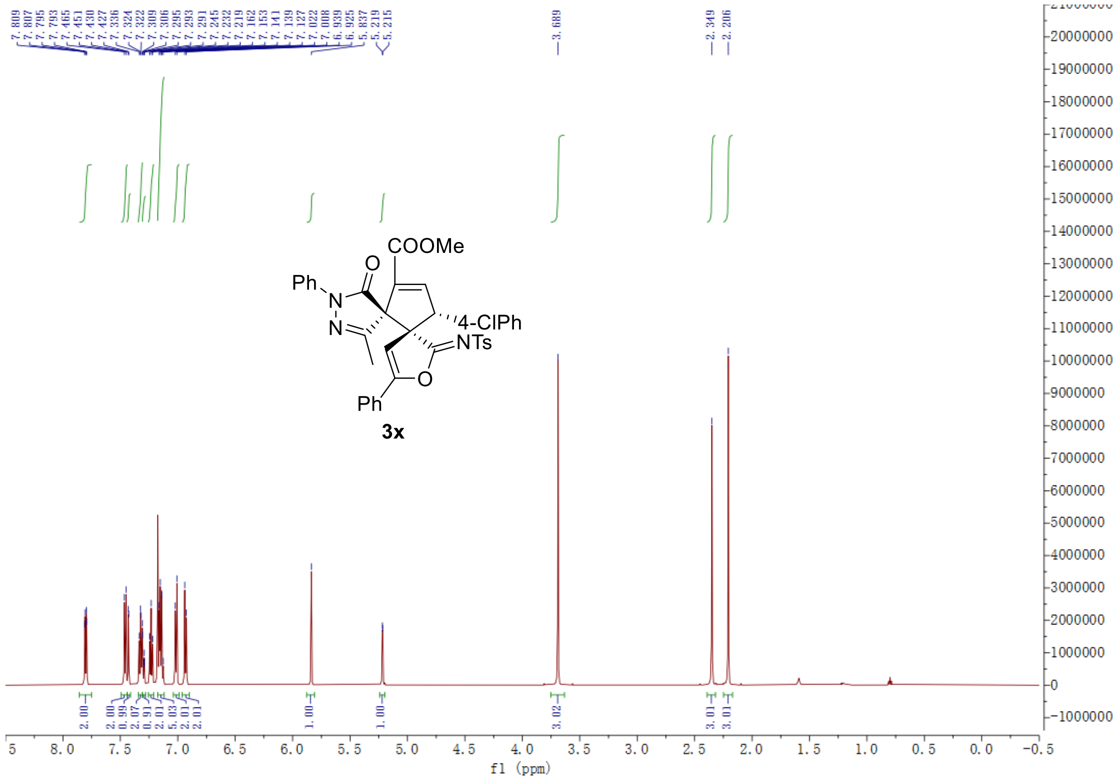

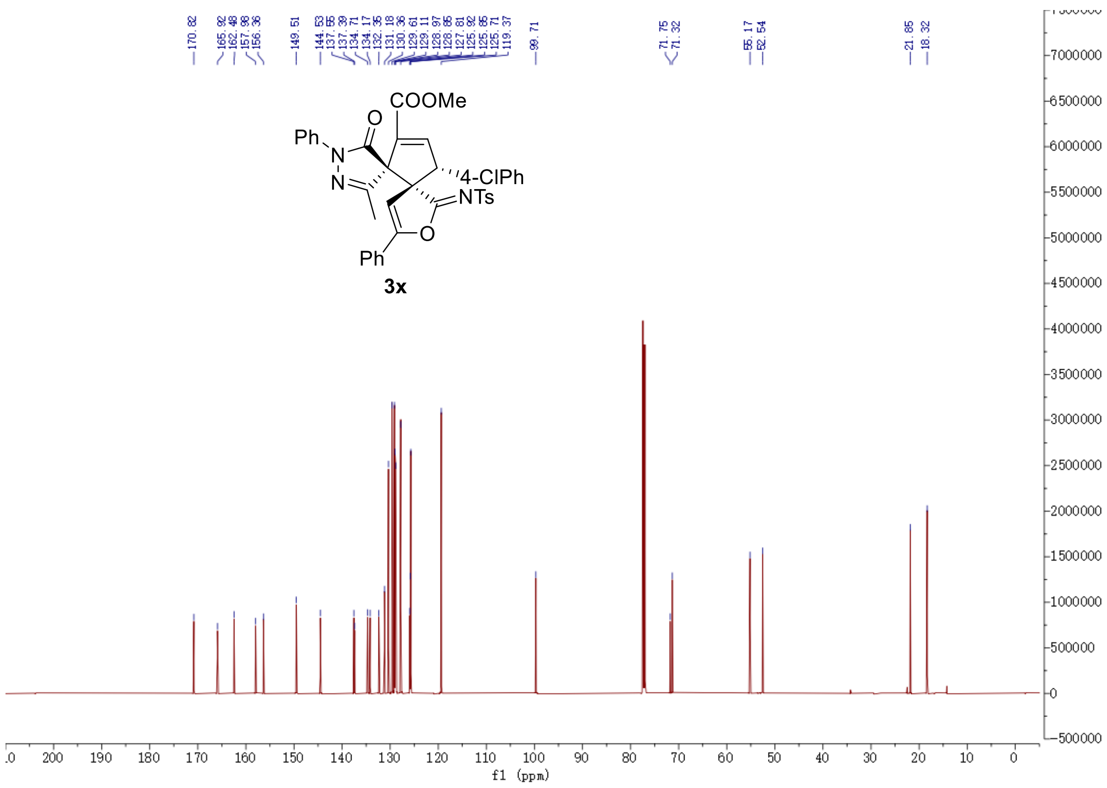

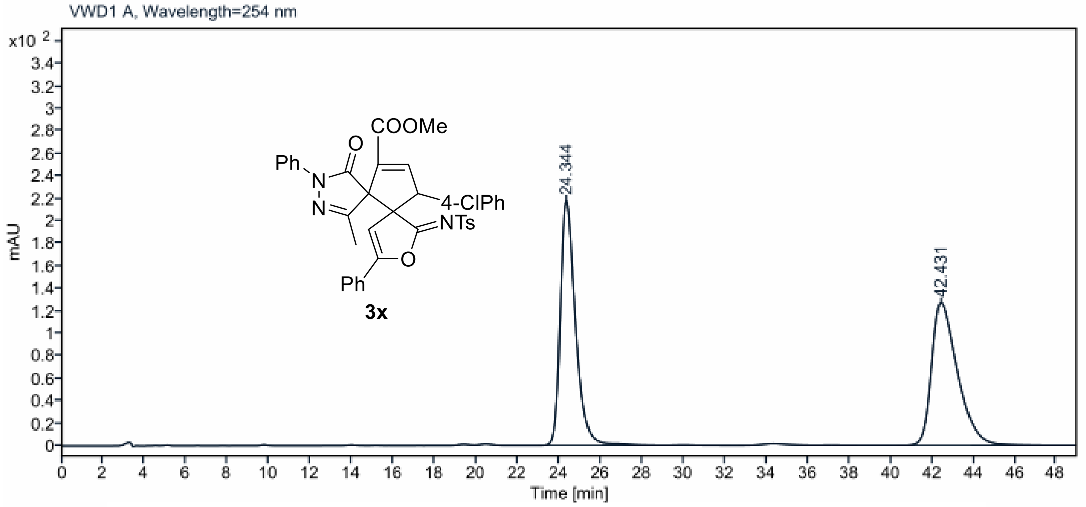

Signal: VWD1 A, Wavelength=254 nm

RT [min]	Type	Width [min]	Area	Height	Area%	Name
24.344	BB	0.7732	11139.2422	217.6086	50.2622	
42.431	BB	1.3124	11023.0137	126.7756	49.7378	
		Sum	22162.2559			

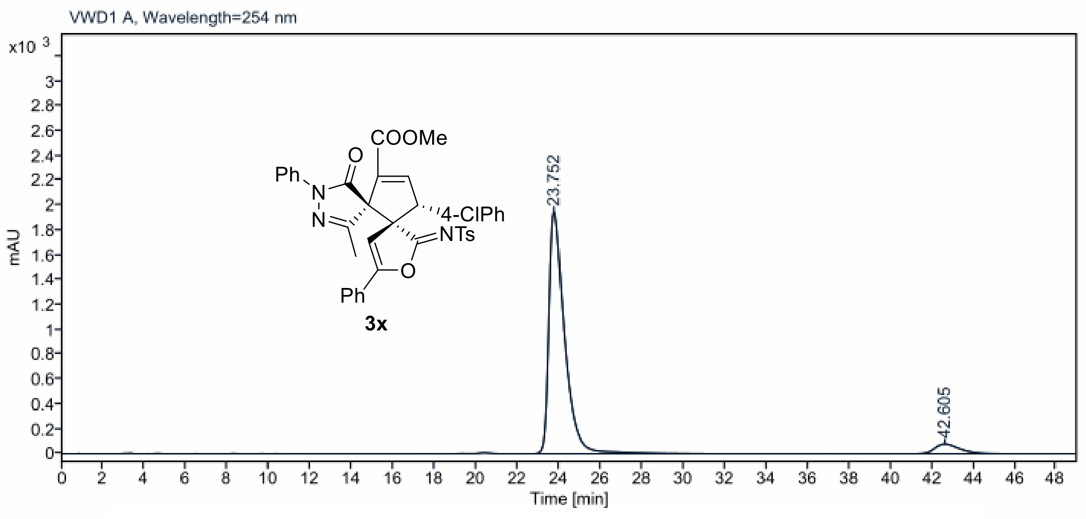

Signal: VWD1 A, Wavelength=254 nm

RT [min]	Type	Width [min]	Area	Height	Area%	Name
23.752	MM	0.8780	102656.0781	1948.6219	93.8611	
42.605	MM	1.4090	6714.0903	79.4209	6.1389	
		Sum	109370.1685			

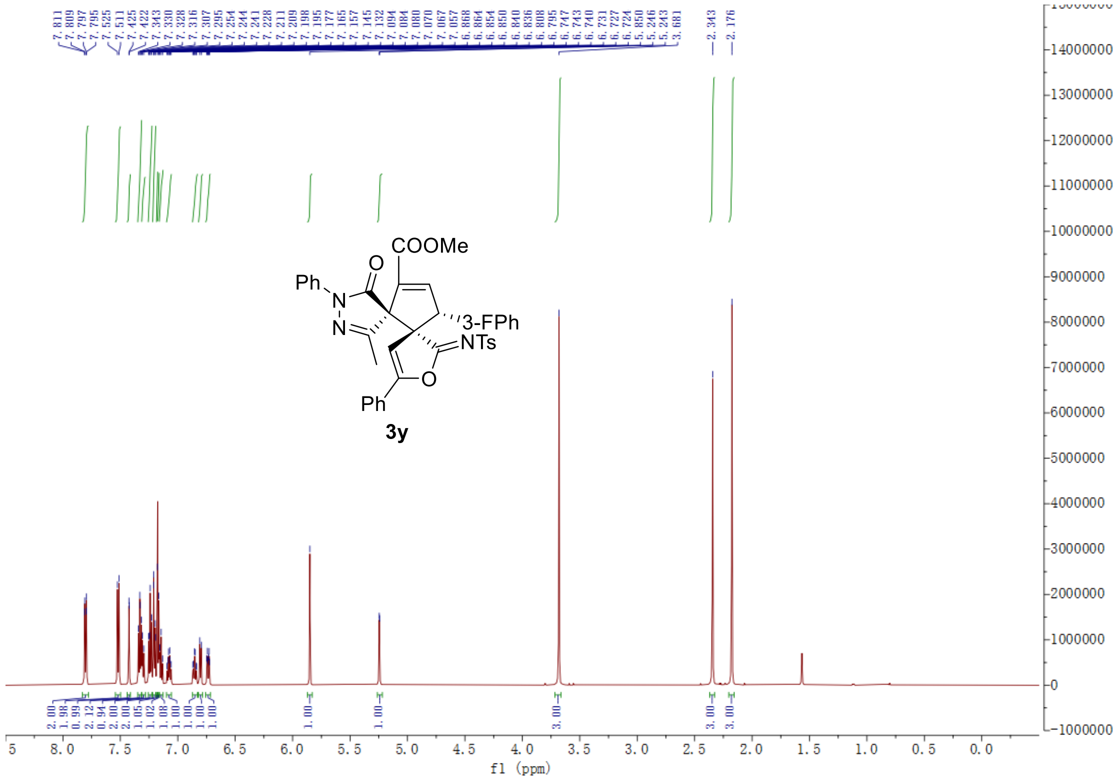

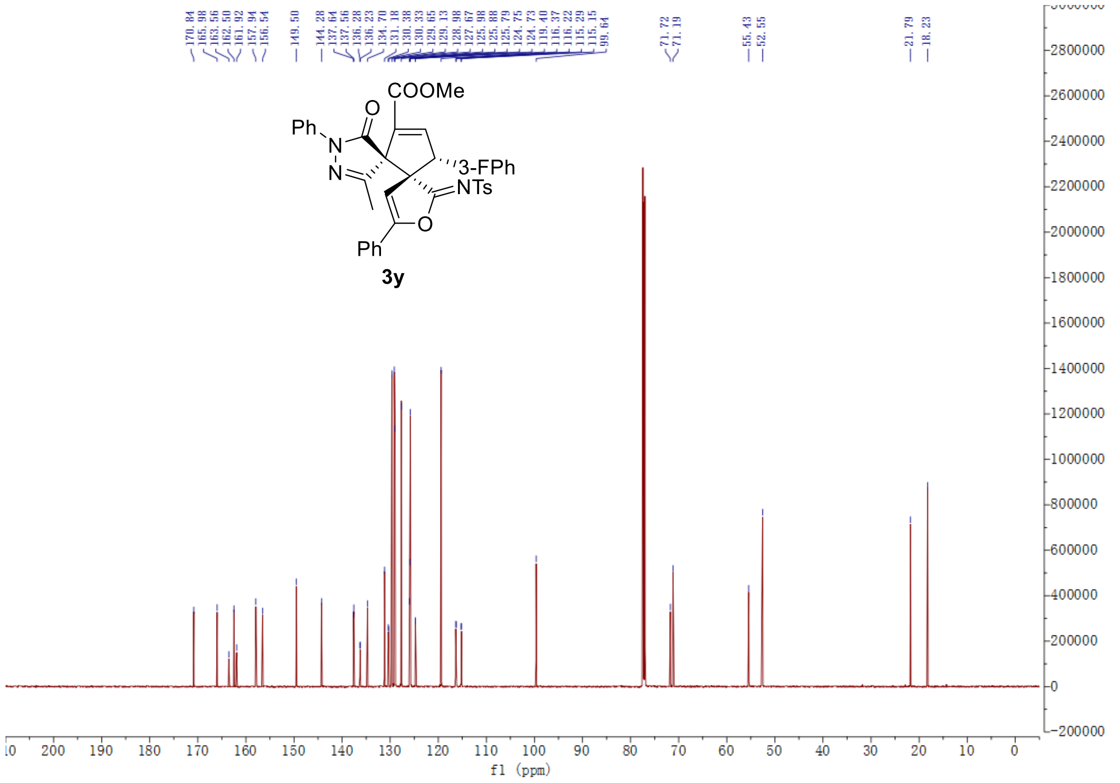
170.84
165.98
163.56
162.50
161.92
157.94
156.54
149.50
144.28
137.64
137.56
136.28
136.23
134.70
131.18
130.38
130.33
129.65
129.13
128.98
127.67
125.98
125.88
125.79
124.75
124.73
119.40
116.37
116.22
115.29
115.15
99.64
71.72
71.19
55.43
52.55
21.79
18.23
COOMe
Ph
N
N
O
3-FPh
NTs
O
Ph
3y
f1 (ppm)

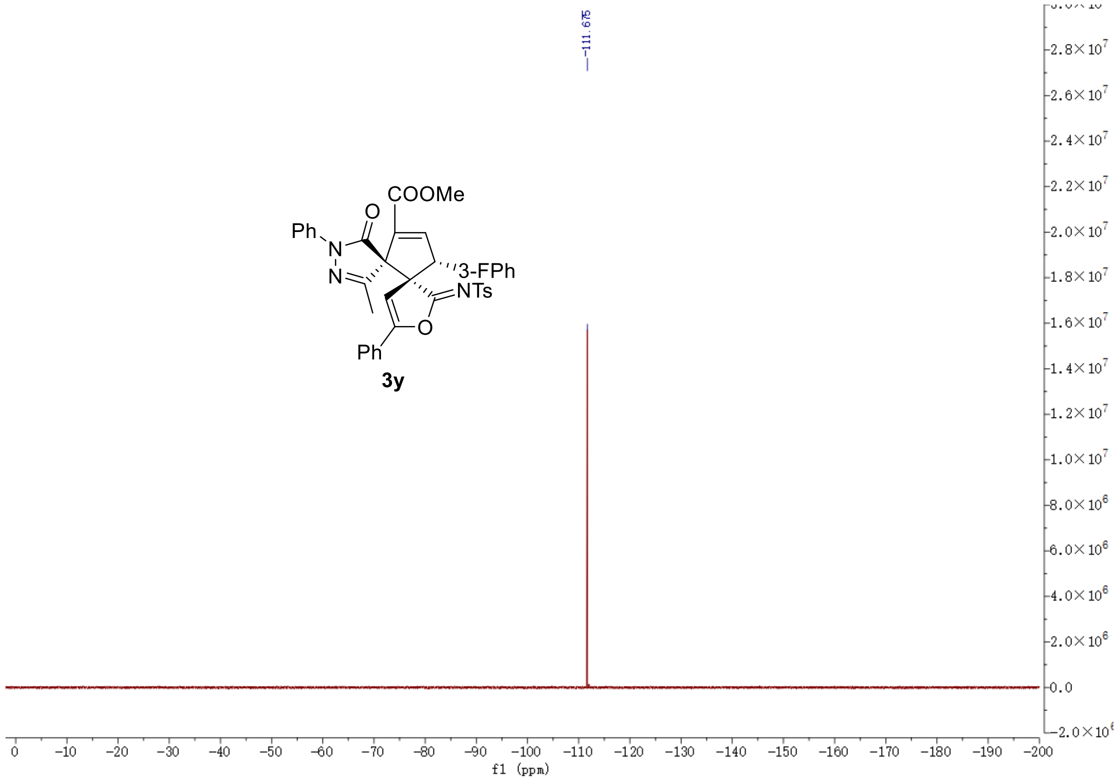
−111.675
COOMe
Ph
N
N
O
3-FPh
NTs
O
Ph
3y
f1 (ppm)

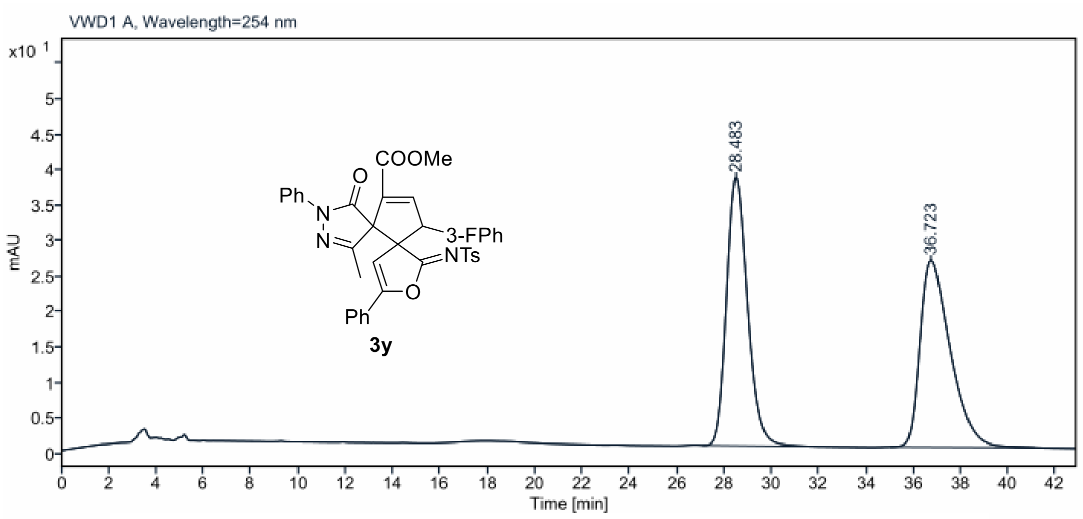

Signal: VWD1 A, Wavelength=254 nm

RT [min]	Type	Width [min]	Area	Height	Area%	Name
28.483	MM	1.0303	2336.2068	37.7912	49.9607	
36.723	MM	1.4815	2339.8777	26.3225	50.0393	
		Sum	4676.0845			

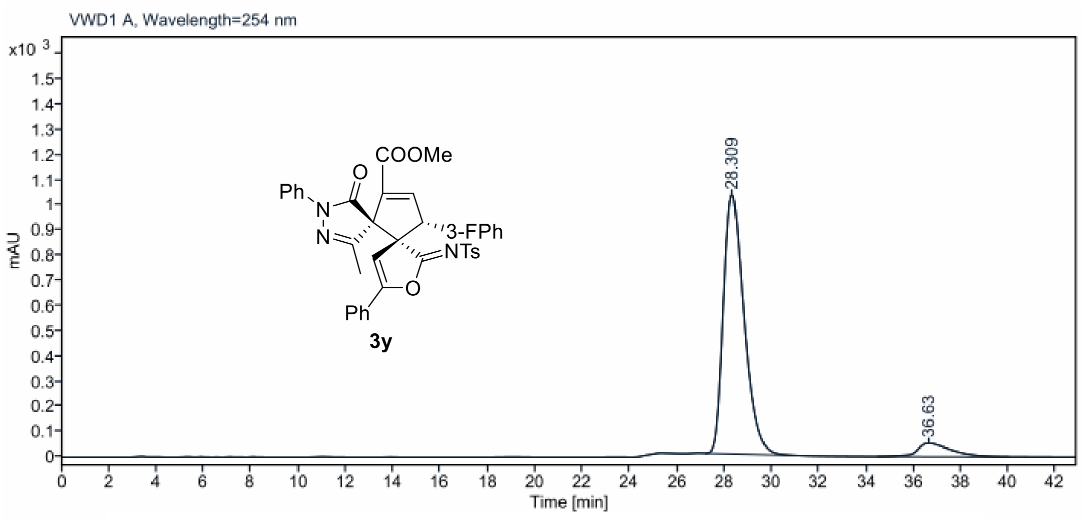

Signal: VWD1 A, Wavelength=254 nm

RT [min]	Type	Width [min]	Area	Height	Area%	Name
28.309	MM	1.0173	62826.5703	1029.2905	92.2043	
36.630	MM	1.6233	5311.8354	54.5361	7.7957	
		Sum	68138.4058			

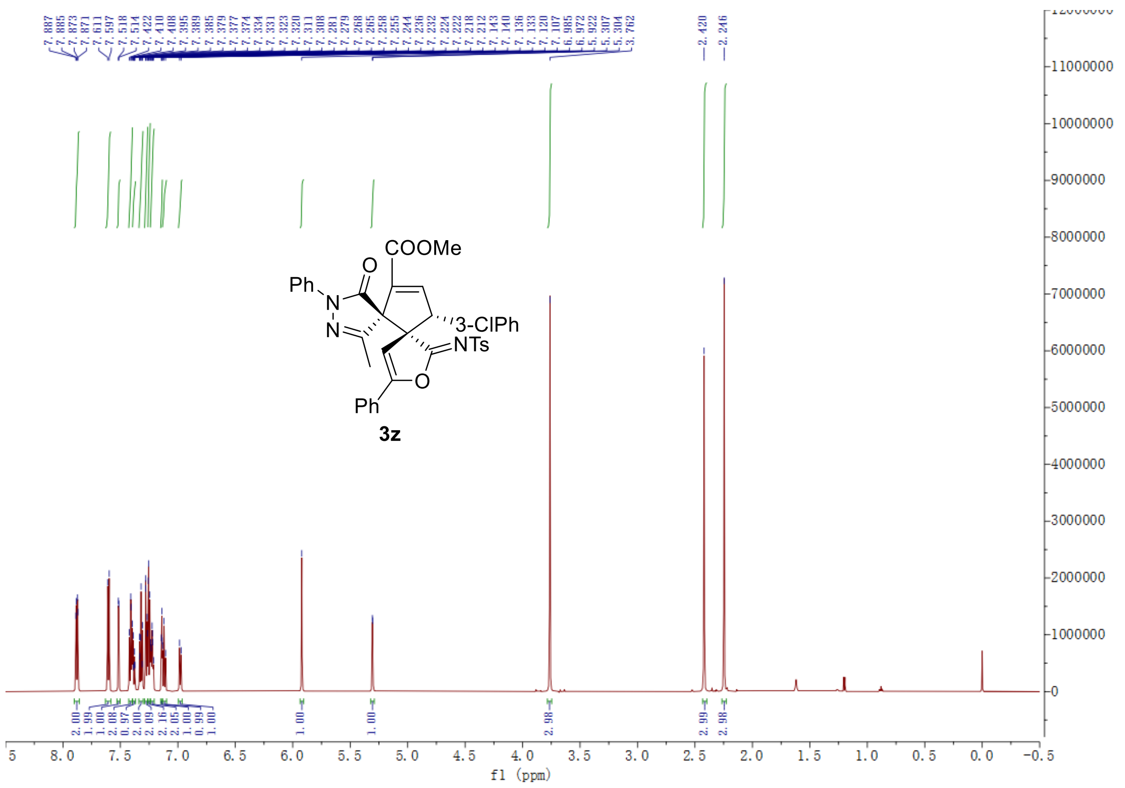
COOMe
Ph
N
N
O
3-ClPh
NTs
O
Ph
3z
f1 (ppm)

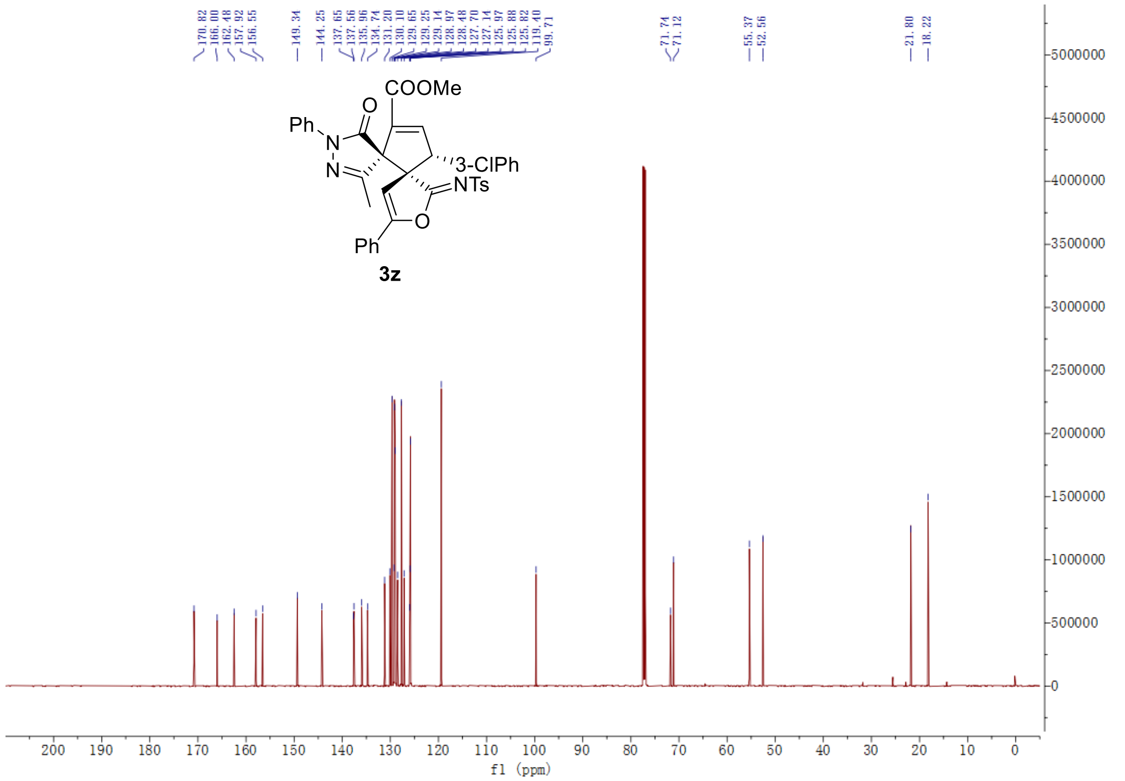
COOMe
Ph
N
N
O
3-ClPh
NTs
O
Ph
3z
f1 (ppm)

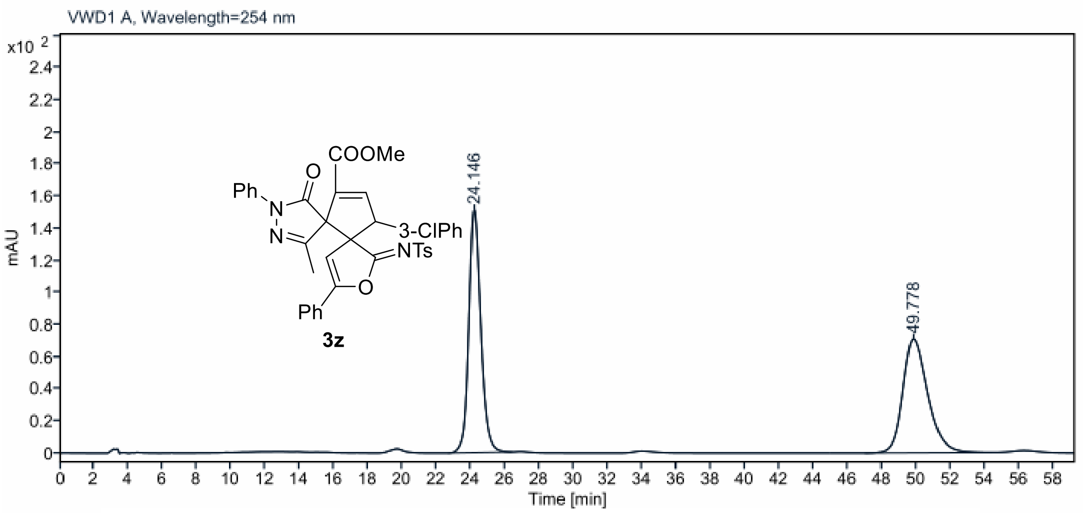

Signal: VWD1 A, Wavelength=254 nm

RT [min]	Type	Width [min]	Area	Height	Area%	Name
24.146	MM	0.8033	7299.7798	151.4472	50.3113	
49.778	MM	1.6912	7209.4316	71.0478	49.6887	
		Sum	14509.2114			

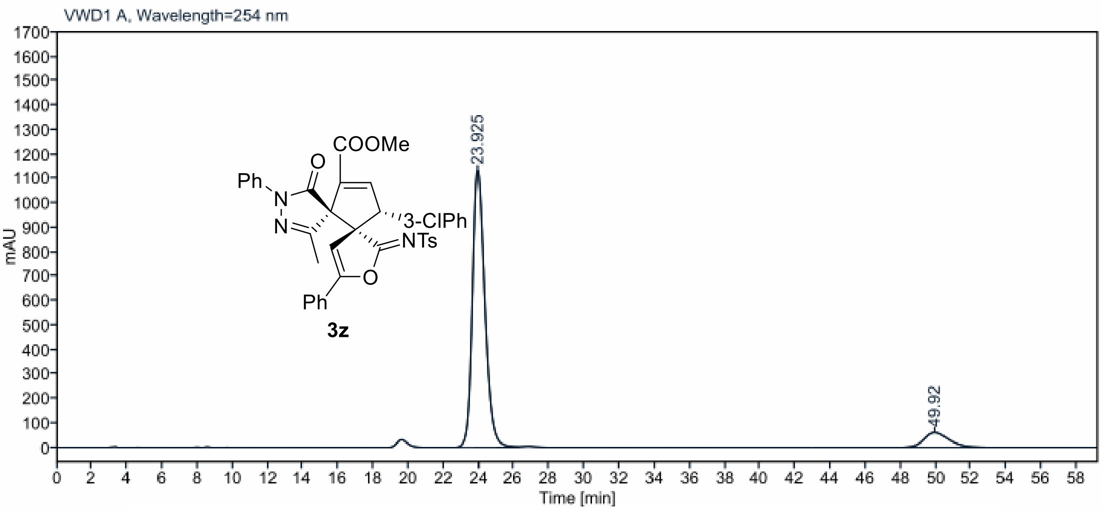

Signal: VWD1 A, Wavelength=254 nm

RT [min]	Type	Width [min]	Area	Height	Area%	Name
23.925	MM	0.8124	55324.6992	1134.9911	89.8520	
49.920	MM	1.6764	6248.4438	62.1221	10.1480	
		Sum	61573.1431			

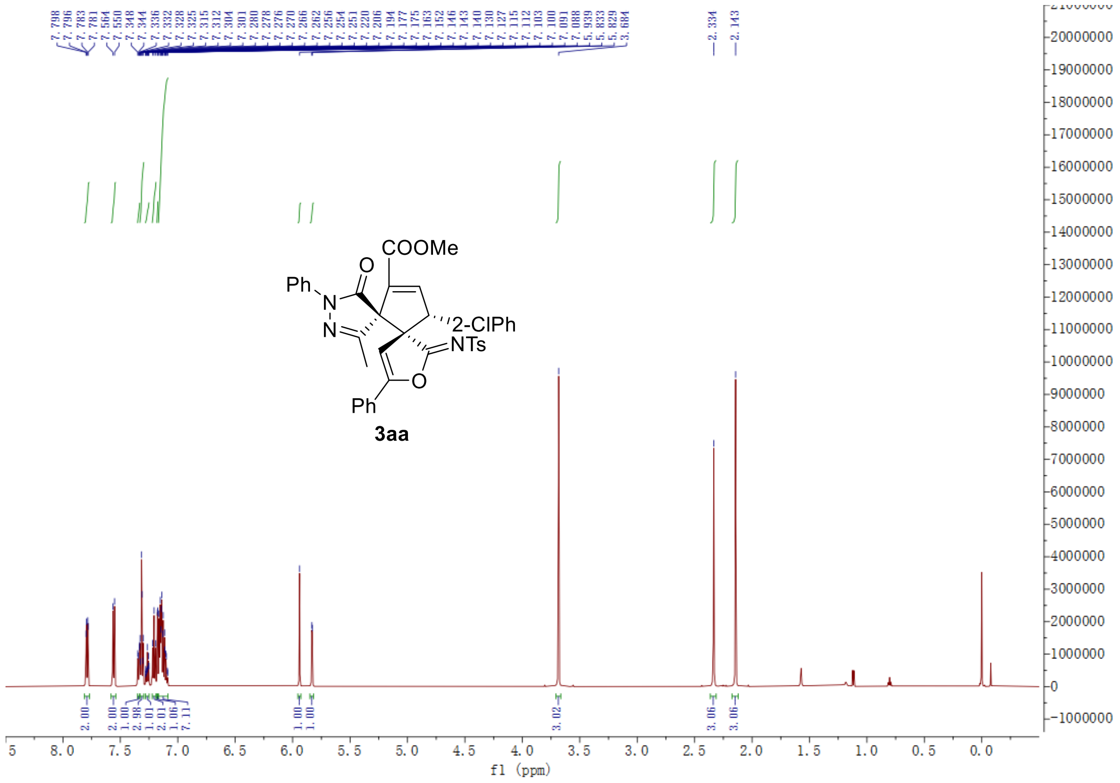
COOMe
O
Ph
N
N
2-ClPh
NTs
O
Ph
3aa
f1 (ppm)

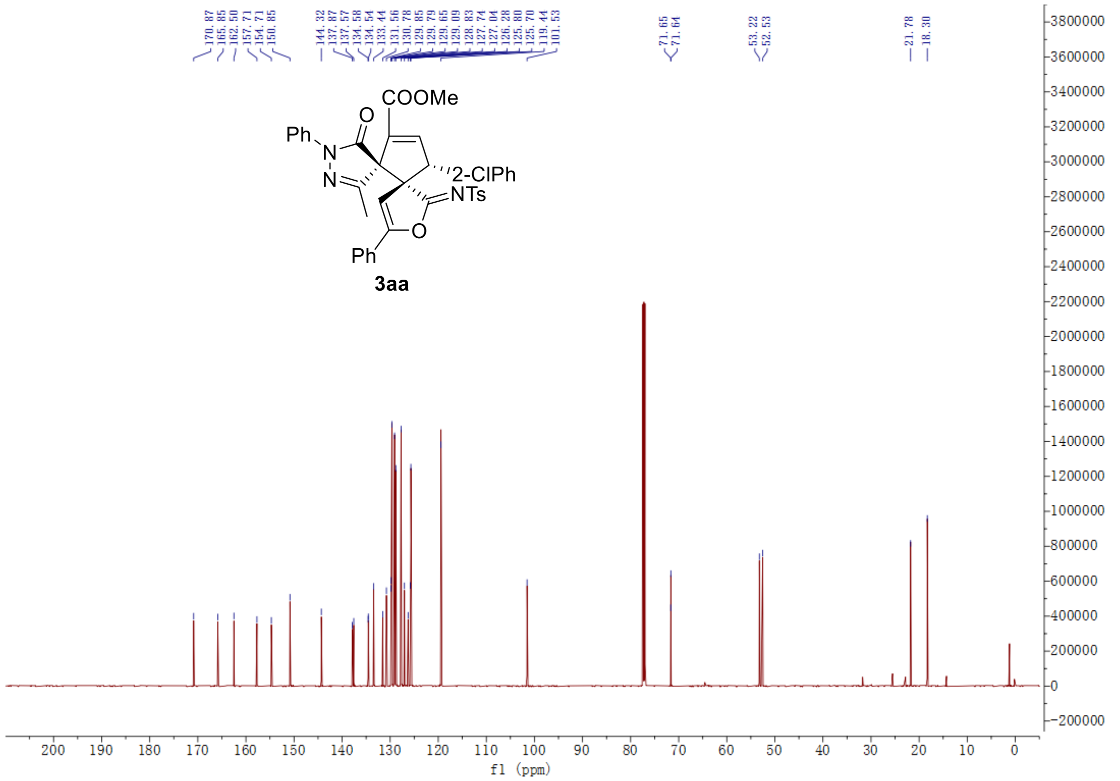
COOMe
O
Ph
N
N
2-ClPh
NTs
O
Ph
3aa
f1 (ppm)

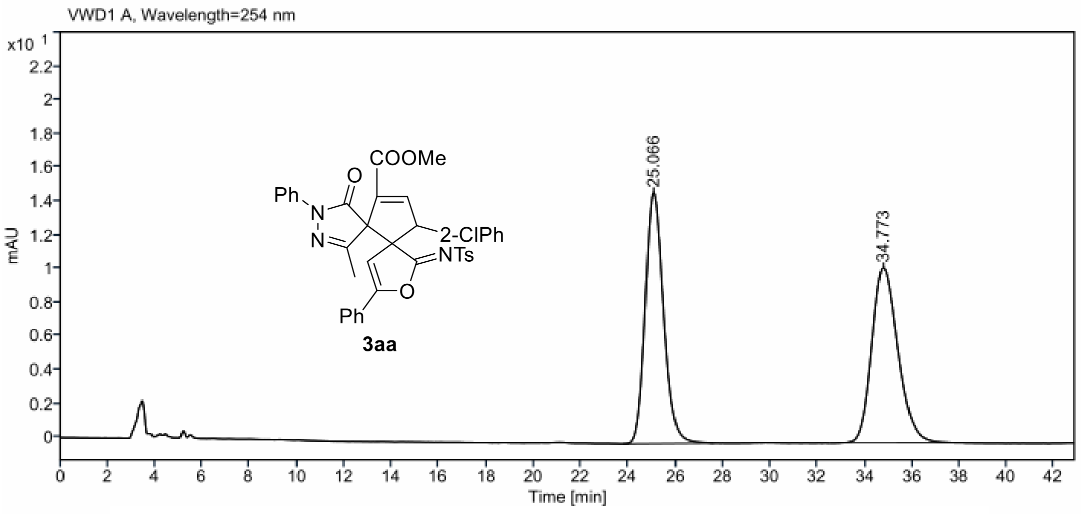

Signal: VWD1 A, Wavelength=254 nm

RT [min]	Type	Width [min]	Area	Height	Area%	Name
25.066	BB	0.8319	801.2975	14.8880	49.9465	
34.773	BB	1.1805	803.0134	10.4042	50.0535	
		Sum	1604.3110			

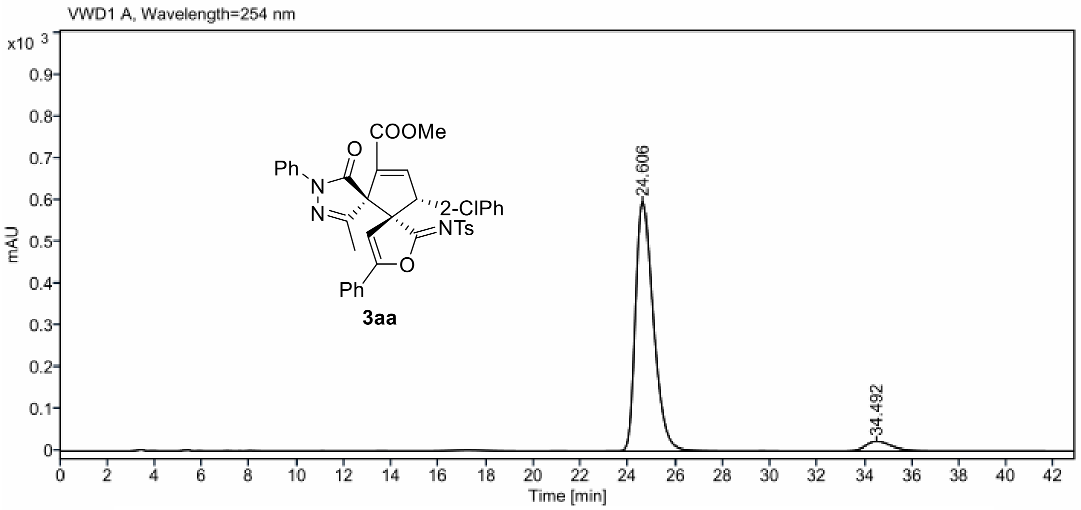

Signal: VWD1 A, Wavelength=254 nm

RT [min]	Type	Width [min]	Area	Height	Area%	Name
24.606	BB	0.8055	31286.0820	596.8994	94.8137	
34.492	BB	1.1617	1711.3424	22.6443	5.1863	
		Sum	32997.4244			

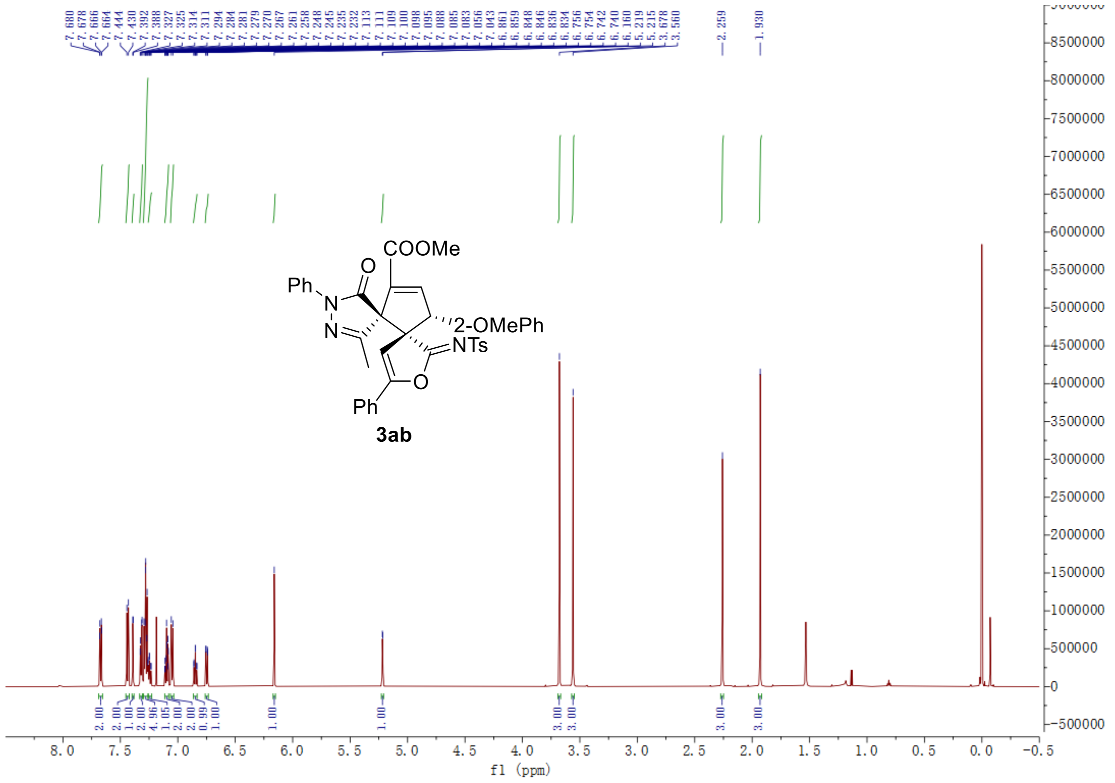
COOMe
Ph
N
N
O
2-OMePh
NTs
O
Ph
3ab
f1 (ppm)

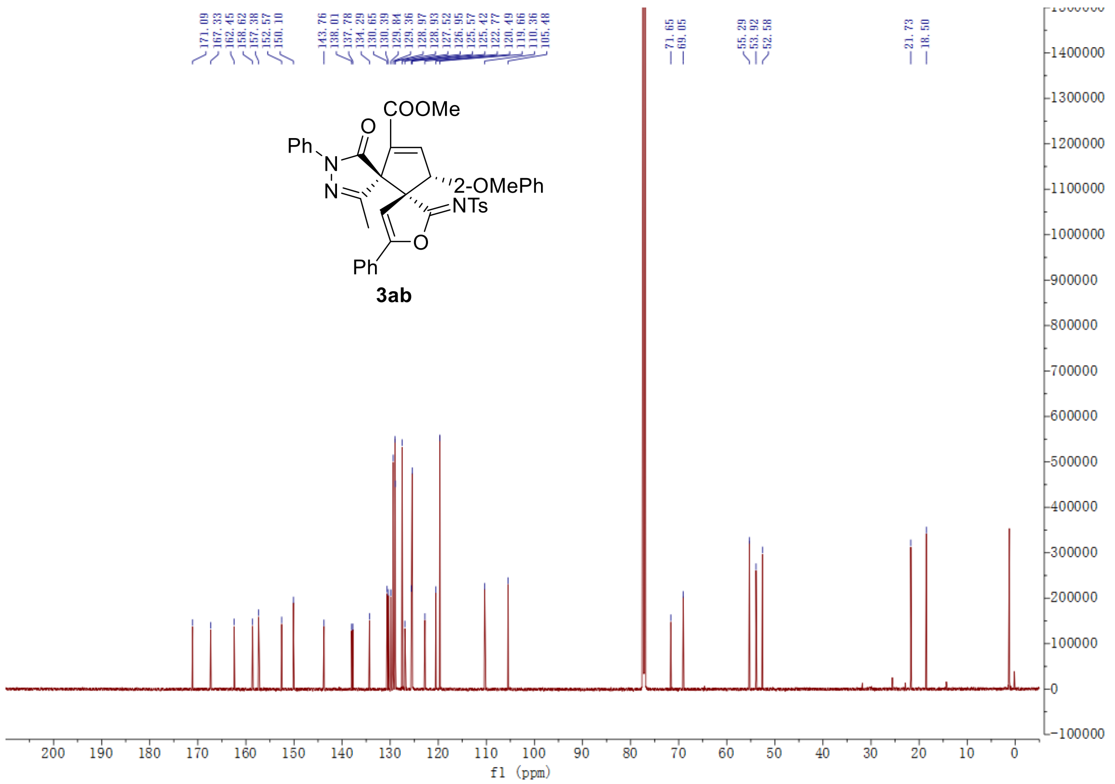
COOMe
Ph
N
N
O
2-OMePh
NTs
O
Ph
3ab
f1 (ppm)

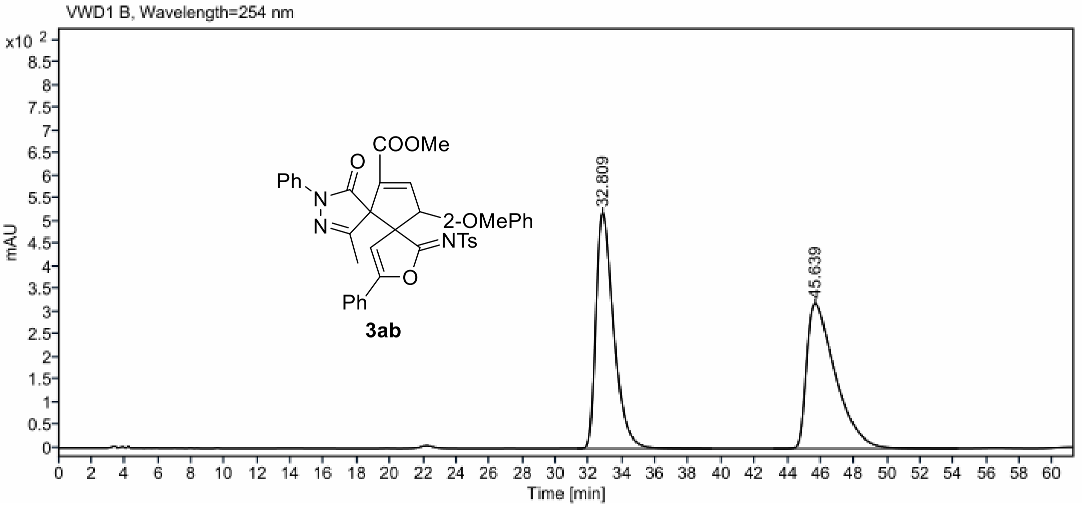

Signal: VWD1 B, Wavelength=254 nm

RT [min]	Type	Width [min]	Area	Height	Area%	Name
32.809	BB	1.1436	39001.5938	519.6496	50.2800	
45.639	BB	1.8060	38567.1641	319.5562	49.7200	
		Sum	77568.7578			

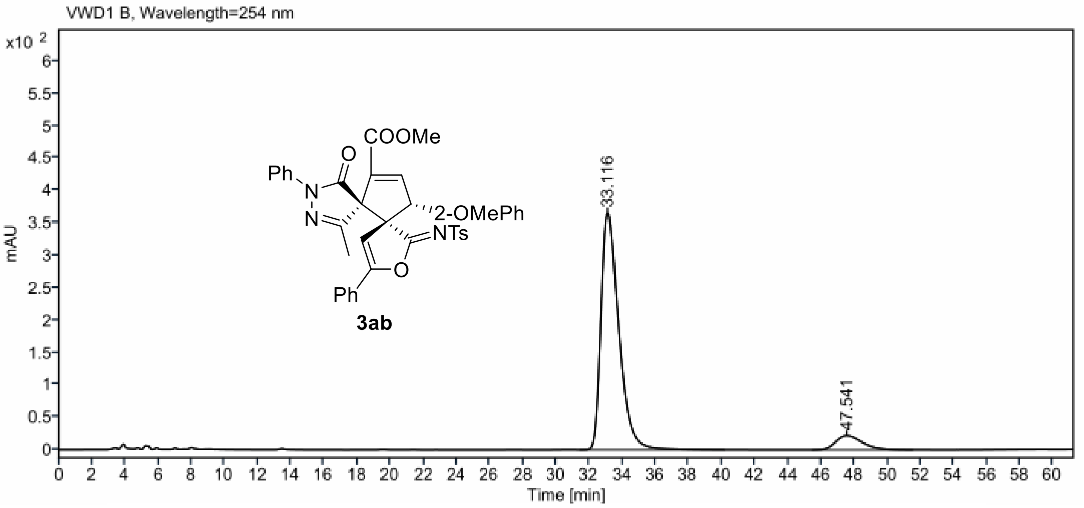

Signal: VWD1 B, Wavelength=254 nm

RT [min]	Type	Width [min]	Area	Height	Area%	Name
33.116	MM	1.2453	27309.1719	365.5074	91.8457	
47.541	MM	1.8786	2424.5938	21.5109	8.1543	
		Sum	29733.7656			

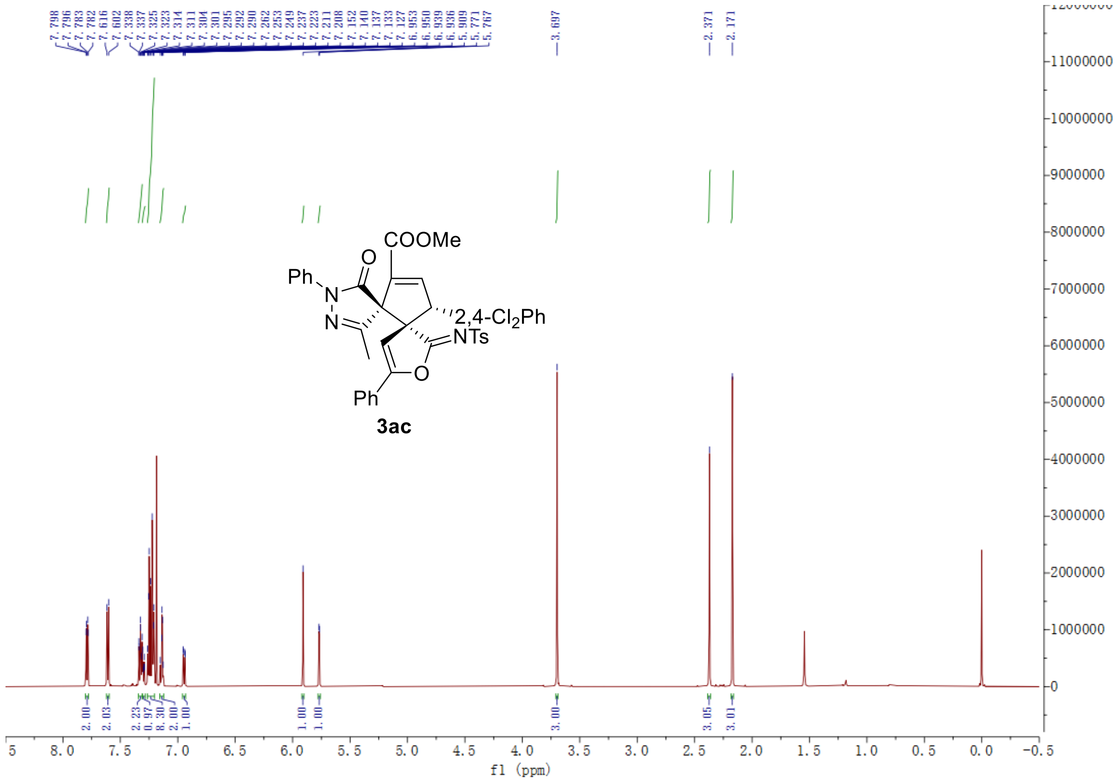

COOMe
Ph
N
N
O
2,4-Cl2Ph
NTs
O
Ph
3ac
f1 (ppm)

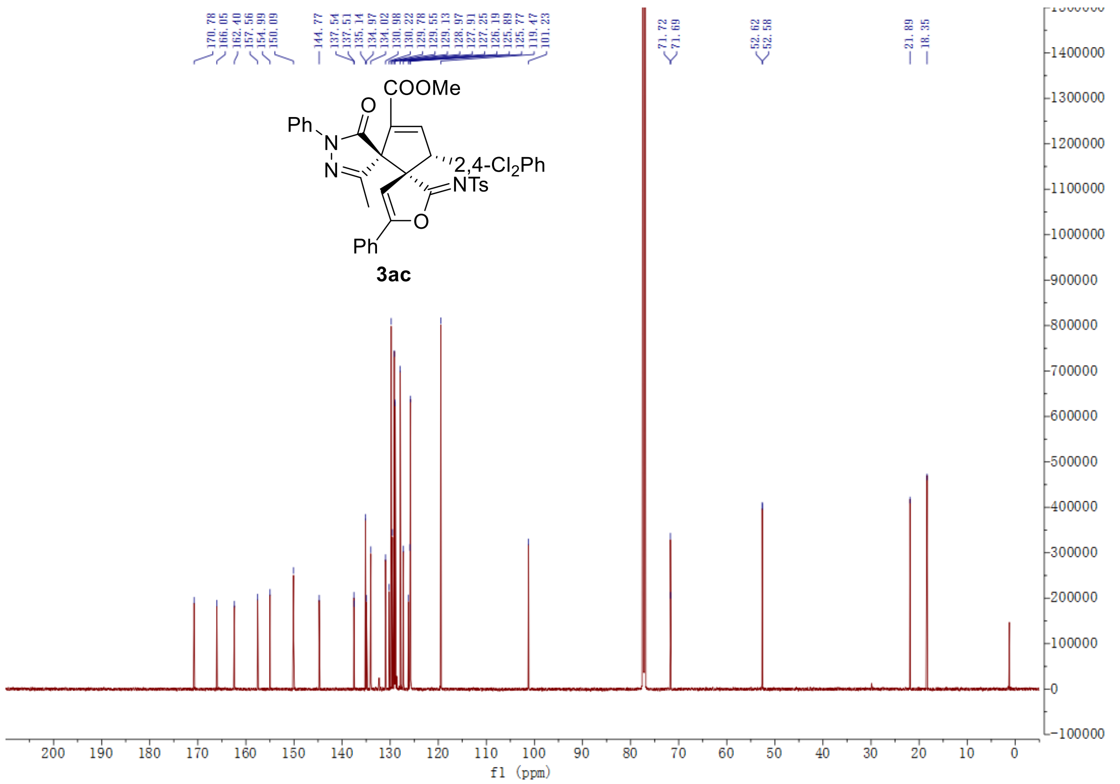

COOMe
Ph
N
N
O
2,4-Cl2Ph
NTs
O
Ph
3ac
f1 (ppm)

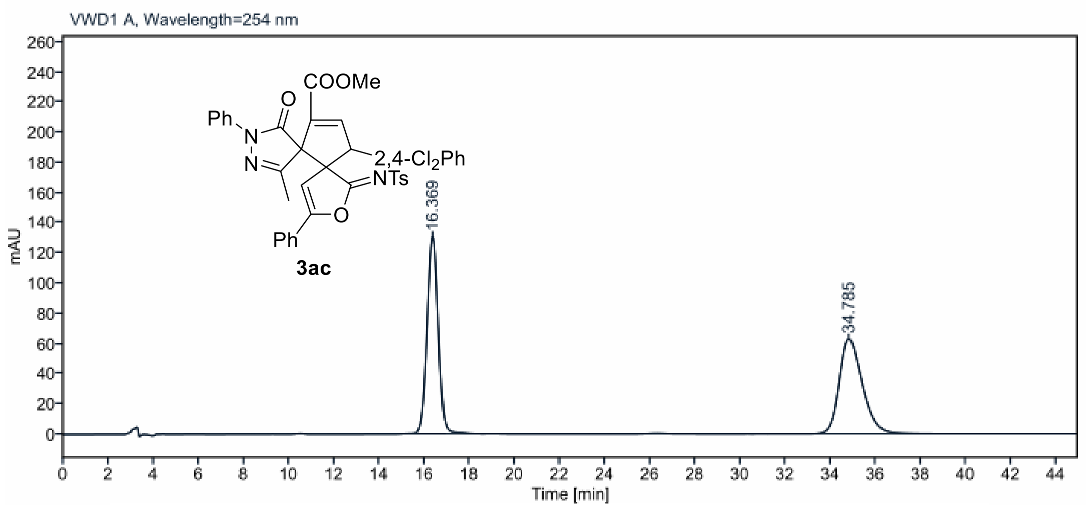

Signal: VWD1 A, Wavelength=254 nm

RT [min]	Type	Width [min]	Area	Height	Area%	Name
16.369	MM	0.5784	4531.0186	130.5613	50.1214	
34.785	BB	1.0938	4509.0615	63.0191	49.8786	
		Sum	9040.0801			

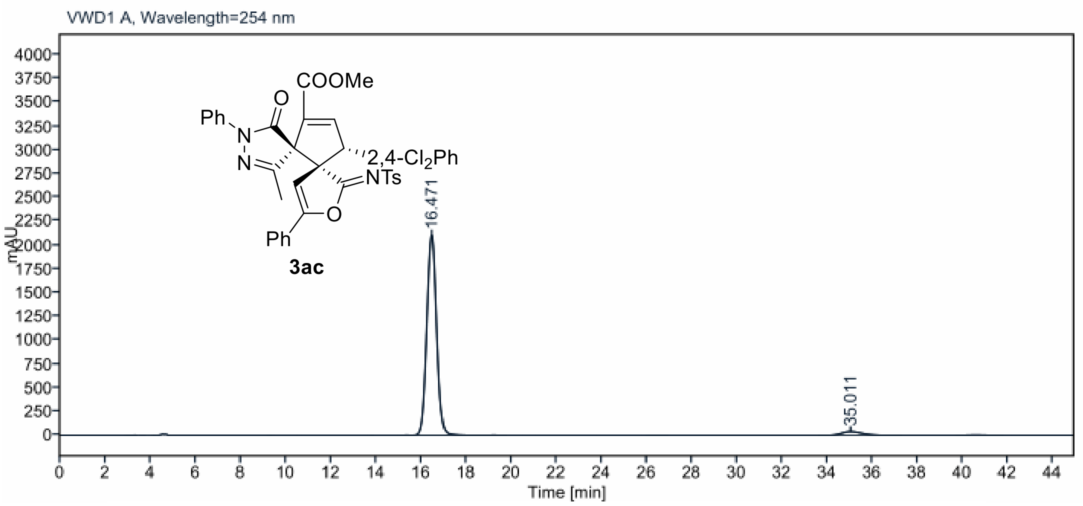

Signal: VWD1 A, Wavelength=254 nm

RT [min]	Type	Width [min]	Area	Height	Area%	Name
16.471	MM	0.5086	64274.5781	2106.1428	95.8674	
35.011	MM	1.1291	2770.6882	40.8974	4.1326	
		Sum	67045.2664			

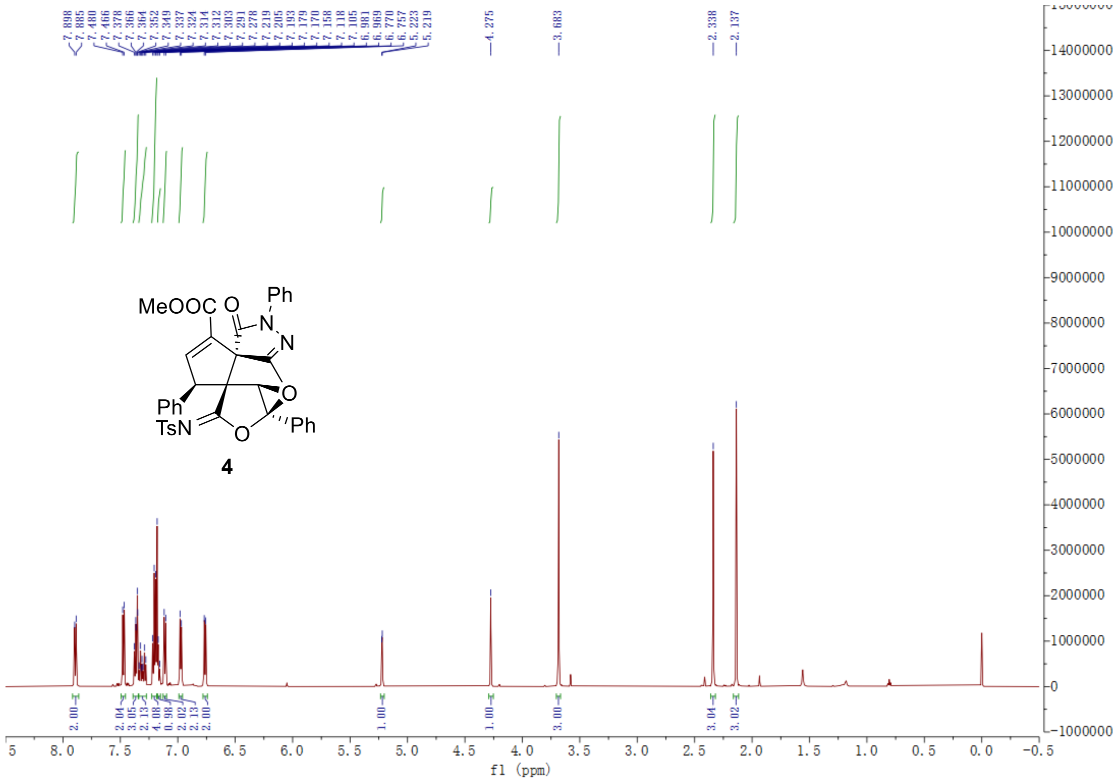

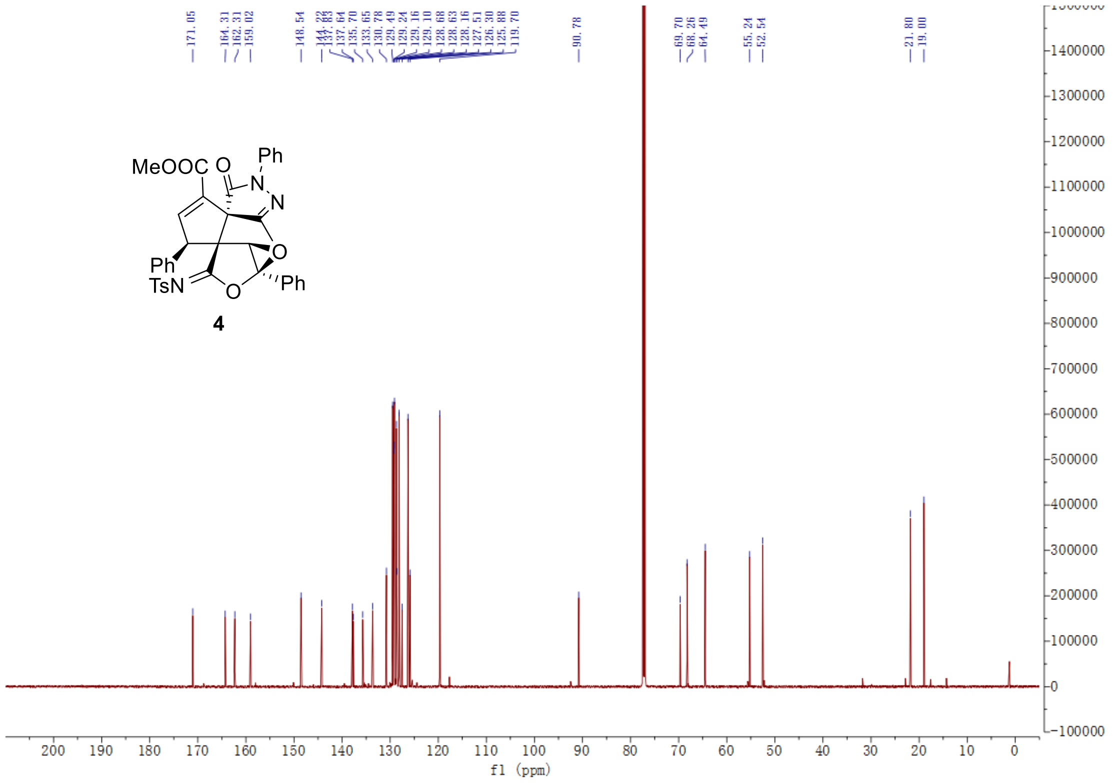

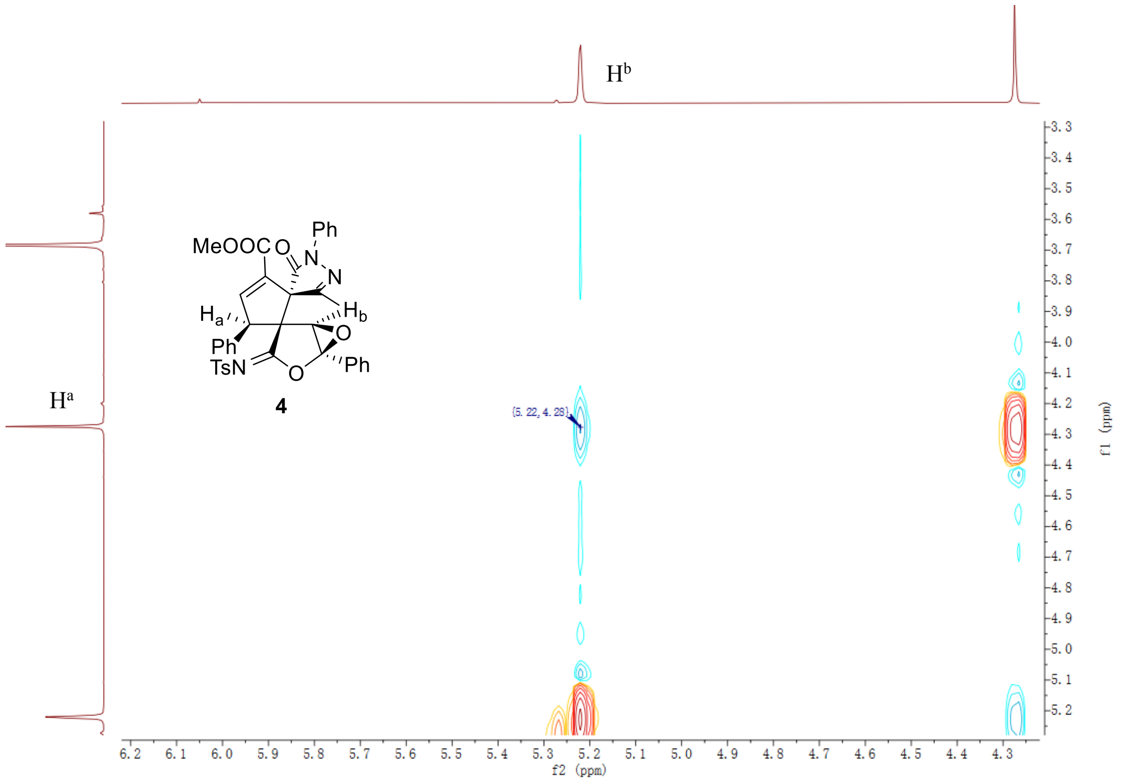

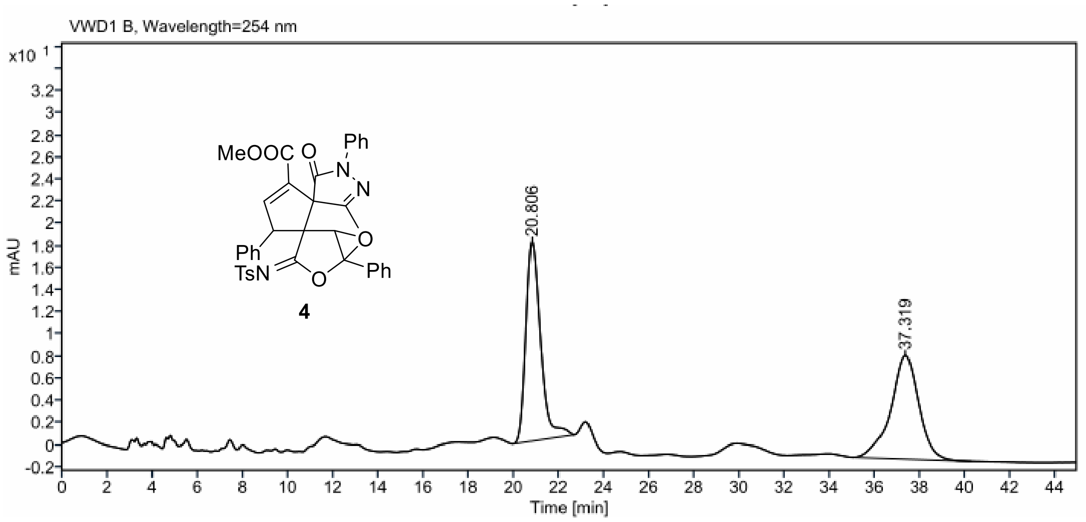

Signal: VWD1 B, Wavelength=254 nm

RT [min]	Type	Width [min]	Area	Height	Area%	Name
20.806	BB	0.6907	811.4865	17.9753	49.9241	
37.319	BB	1.2833	813.9531	9.3710	50.0759	
		Sum	1625.4395			

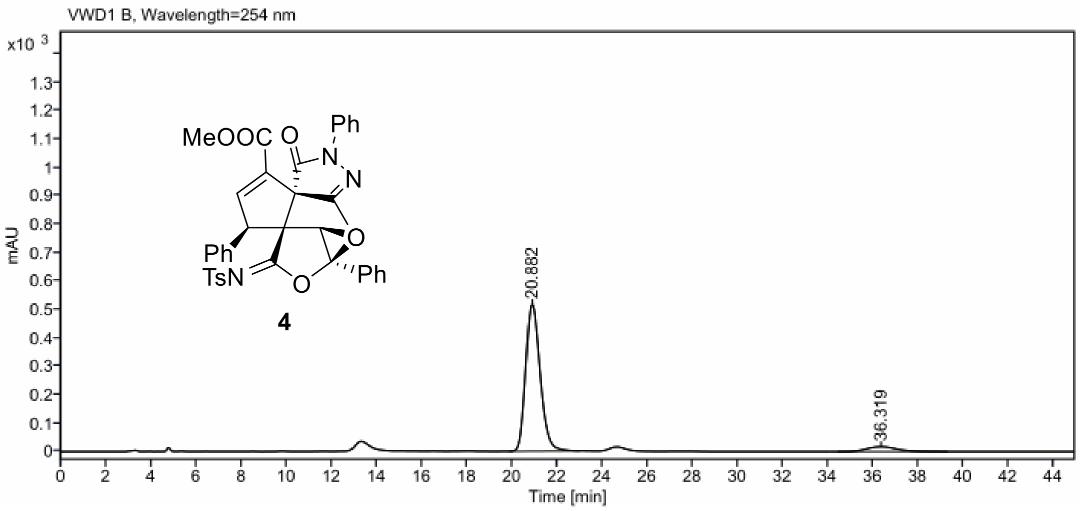

Signal: VWD1 B, Wavelength=254 nm

RT [min]	Type	Width [min]	Area	Height	Area%	Name
20.882	MM	0.7432	23067.5371	517.2722	93.4333	
36.319	MM	1.5574	1621.2286	17.3496	6.5667	
		Sum	24688.7657			

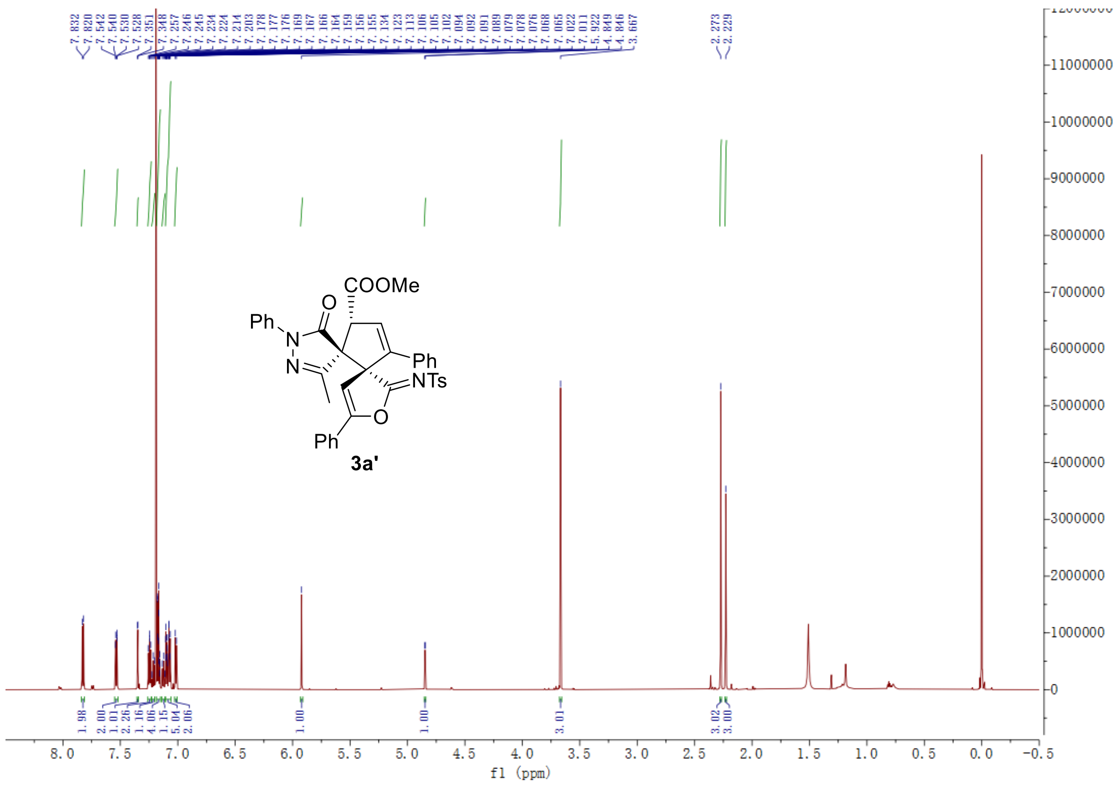

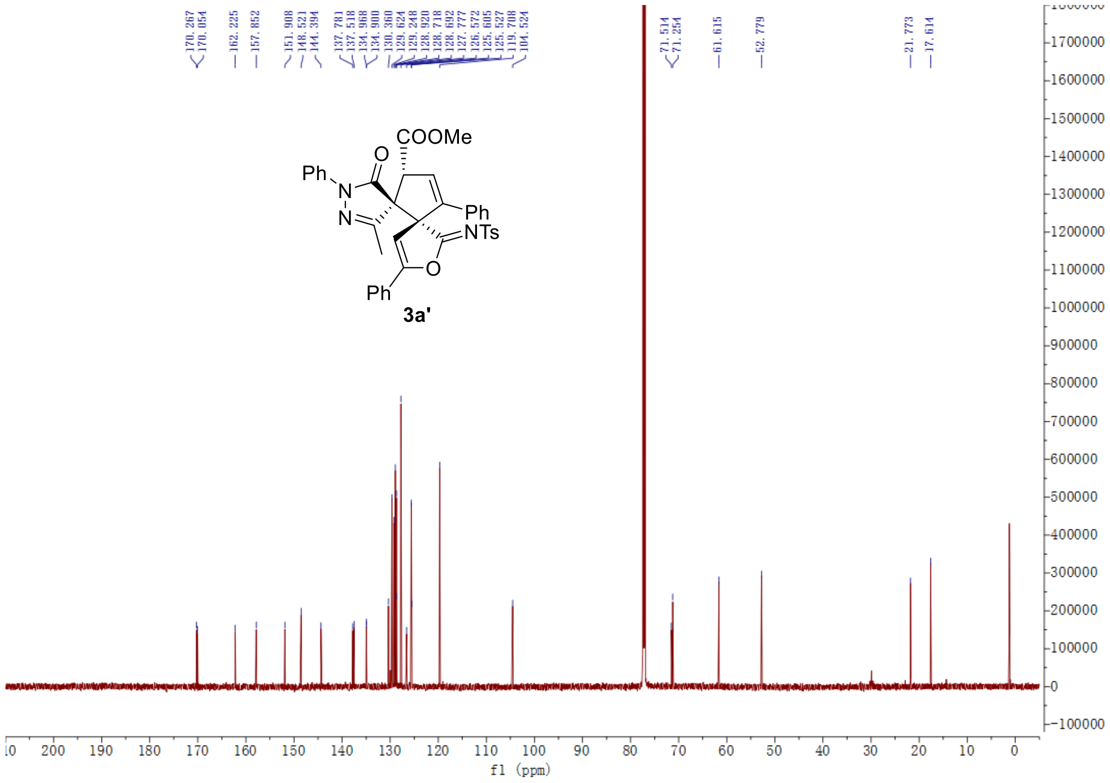

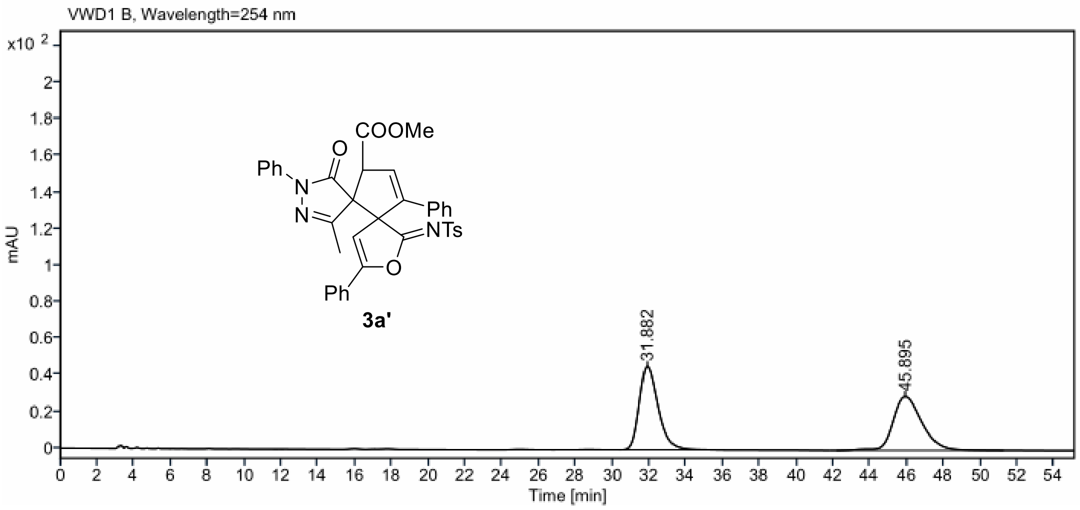

Signal: VWD1 B, Wavelength=254 nm

RT [min]	Type	Width [min]	Area	Height	Area%	Name
31.882	MM	1.1908	3259.2886	45.6180	50.2375	
45.895	MM	1.8263	3228.4766	29.4632	49.7625	
		Sum	6487.7651			

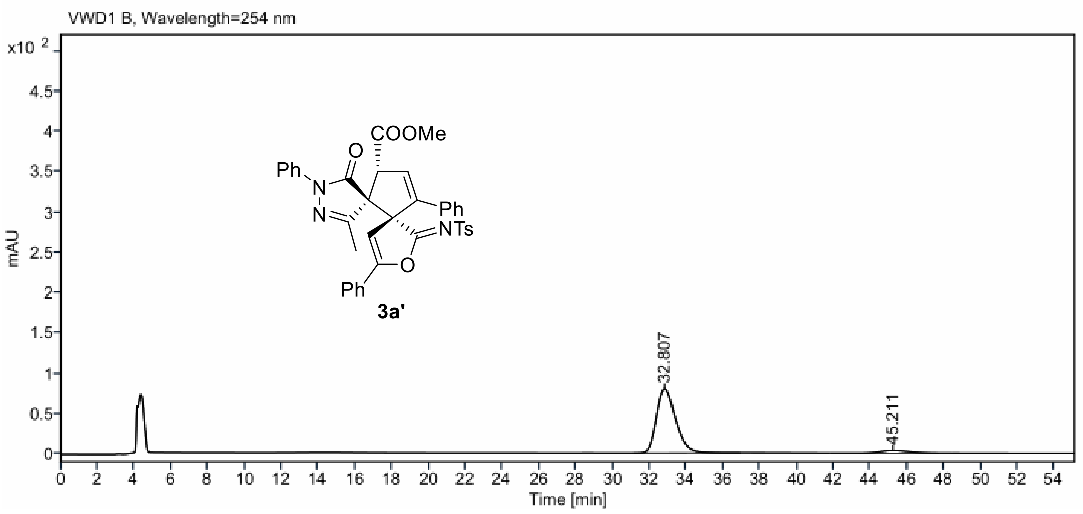

Signal: VWD1 B, Wavelength=254 nm

RT [min]	Type	Width [min]	Area	Height	Area%	Name
32.807	MM	1.2141	5817.6348	79.8653	93.7374	
45.211	MM	1.7773	388.6772	3.6449	6.2626	
		Sum	6206.3120			

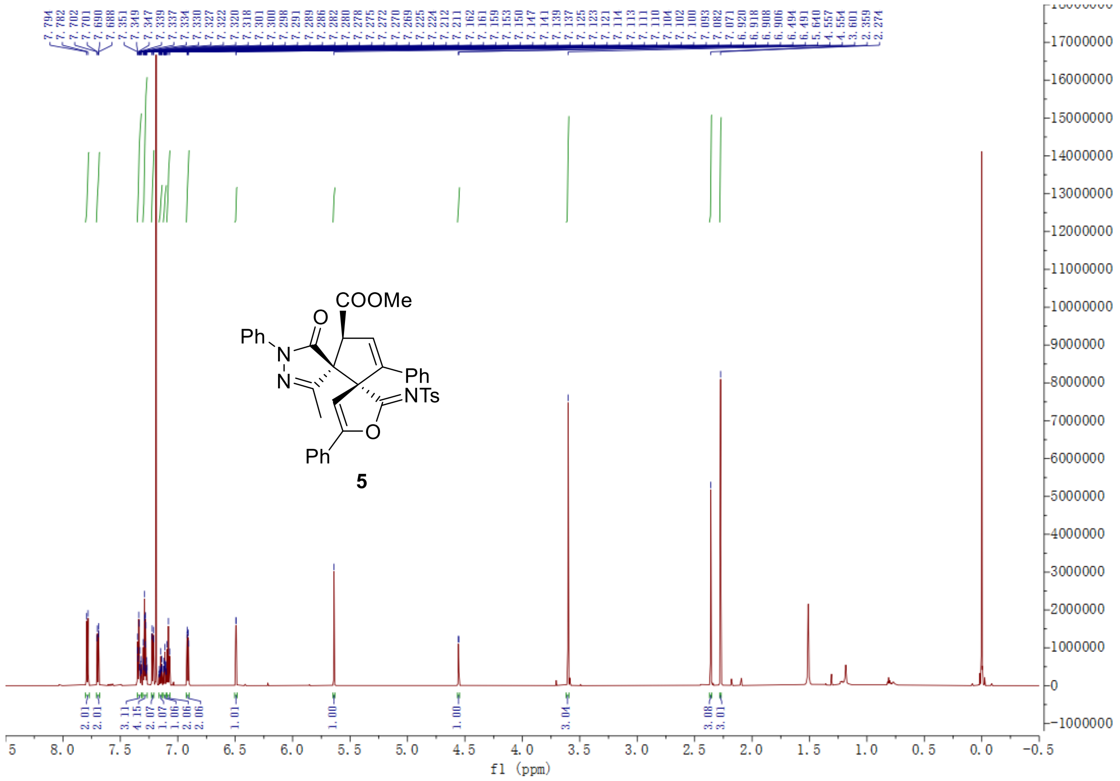

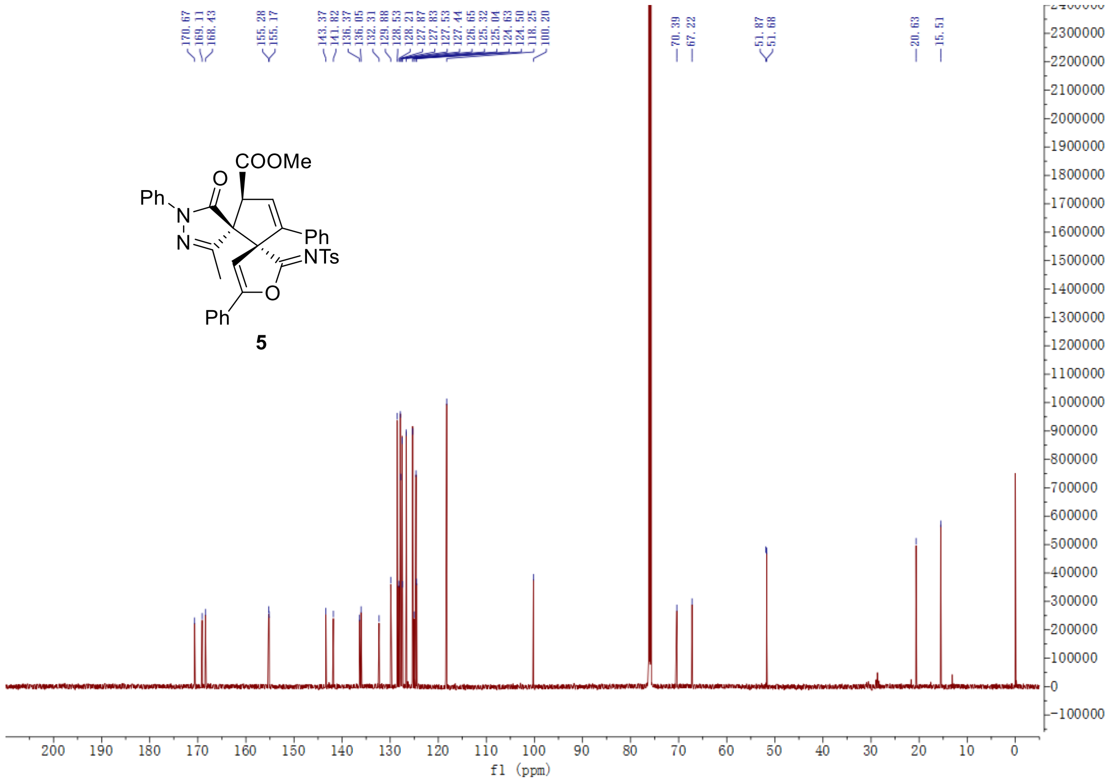
COOMe
Ph
N
N
O
Ph
NTs
O
Ph
5
f1 (ppm)

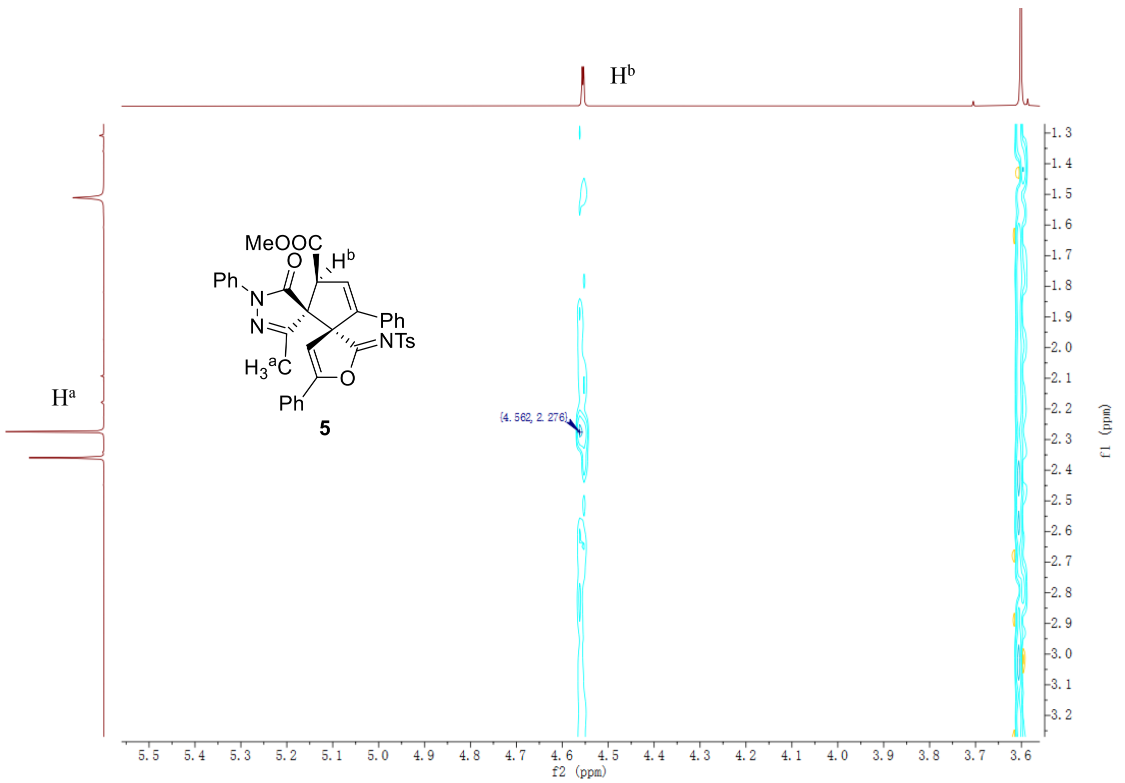
H^b
H^a
MeOOC
H^b
Ph
N
N
O
H3^aC
Ph
NTs
O
Ph
5
(4.562, 2.276)
f2 (ppm)
f1 (ppm)

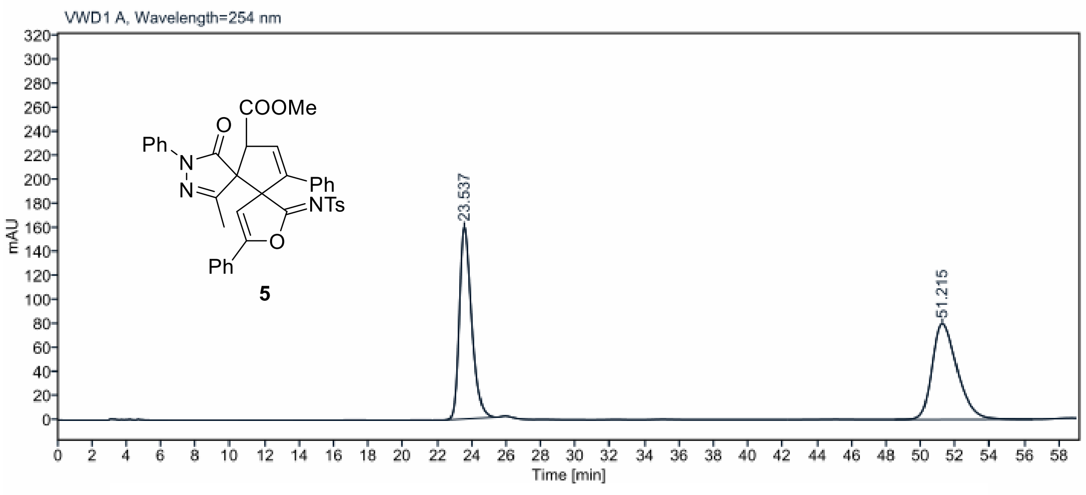

Signal: VWD1 A, Wavelength=254 nm

RT [min]	Type	Width [min]	Area	Height	Area%	Name
23.537	BB	0.7579	7987.9888	160.1406	49.6182	
51.215	BB	1.5489	8110.9175	79.9994	50.3818	
		Sum	16098.9063			

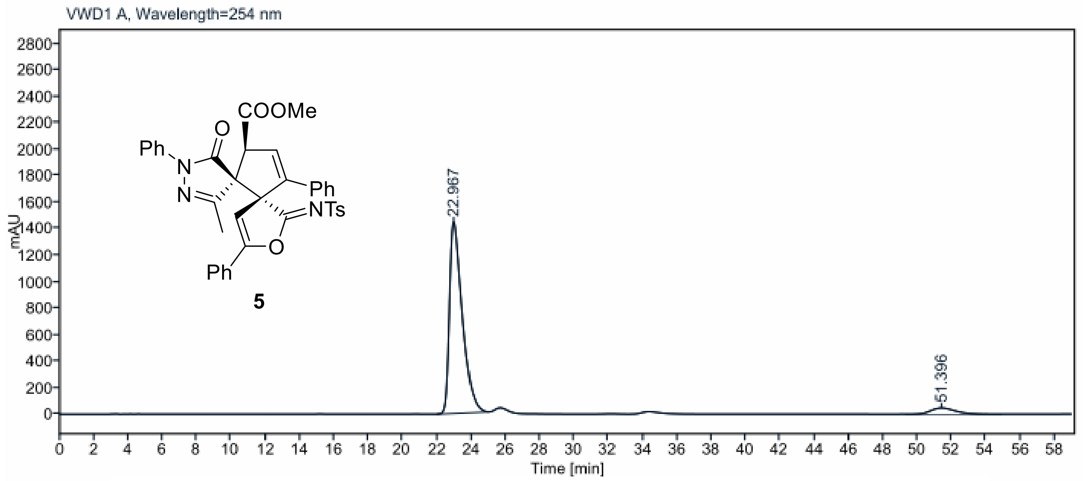

Signal: VWD1 A, Wavelength=254 nm

RT [min]	Type	Width [min]	Area	Height	Area%	Name
22.967	MM	0.8755	76043.0547	1447.5718	93.9949	
51.396	MM	1.6898	4858.1885	47.9163	6.0051	
		Sum	80901.2432			

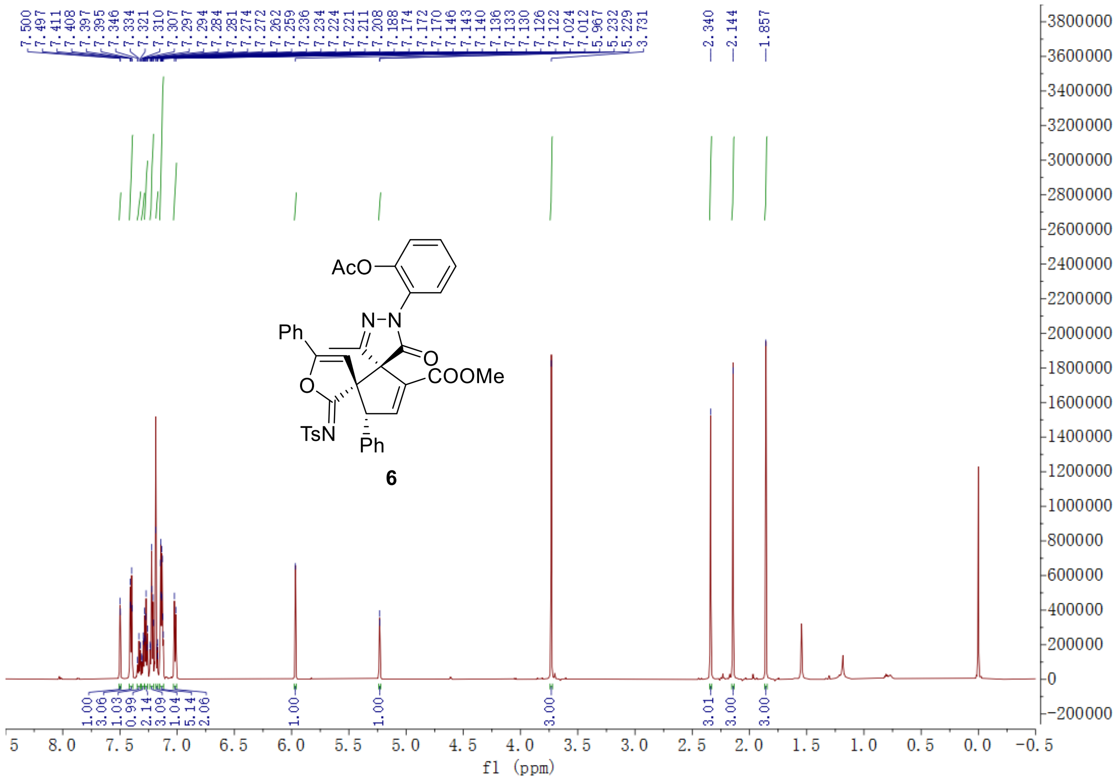
7.500 7.497 7.411 7.408 7.397 7.395 7.346 7.334 7.321 7.310 7.307 7.297 7.294 7.284 7.281 7.274 7.272 7.262 7.259 7.236 7.234 7.224 7.221 7.211 7.208 7.188 7.174 7.172 7.170 7.146 7.143 7.140 7.136 7.133 7.130 7.126 7.122 7.024 7.012 5.967 5.232 5.229 3.731
2.340
2.144
1.857
AcO
N–N
Ph
O
COOMe
O
TsN
Ph
6
1.00 3.06 1.03 0.99 2.14 3.09 1.04 5.14 2.06
1.00
1.00
3.00
3.01 3.00 3.00
f1 (ppm)

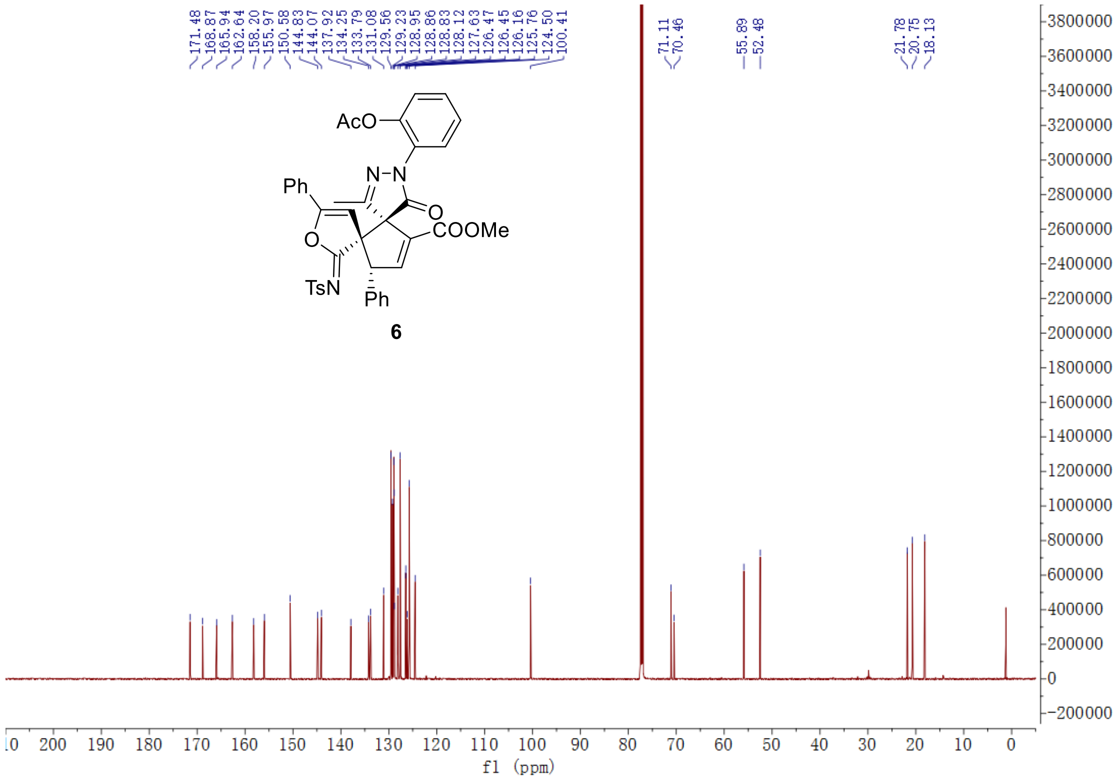
171.48 168.87 165.94 162.64 158.20 155.97 150.58 144.83 144.07 137.92 134.25 133.79 131.08 129.56 129.23 128.95 128.86 128.83 128.12 127.63 126.47 126.45 126.16 125.76 124.50 100.41
71.11 70.46
55.89 52.48
21.78 20.75 18.13
AcO
N–N
Ph
O
COOMe
O
TsN
Ph
6
f1 (ppm)

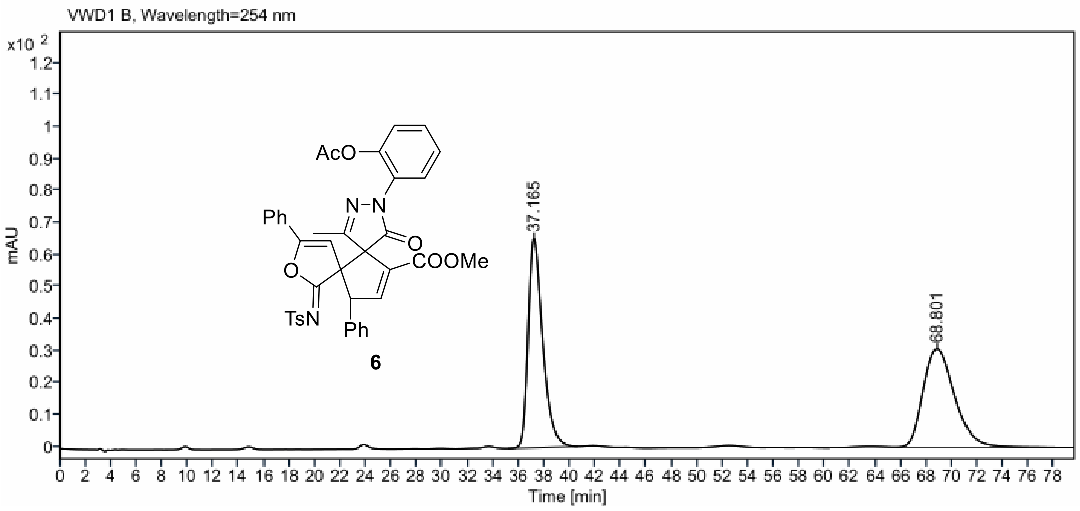

Signal: VWD1 B, Wavelength=254 nm

RT [min]	Type	Width [min]	Area	Height	Area%	Name
37.165	BB	1.1964	5209.6348	65.4637	49.6401	
68.801	MM	2.8609	5285.1714	30.7898	50.3599	
		Sum	10494.8062			

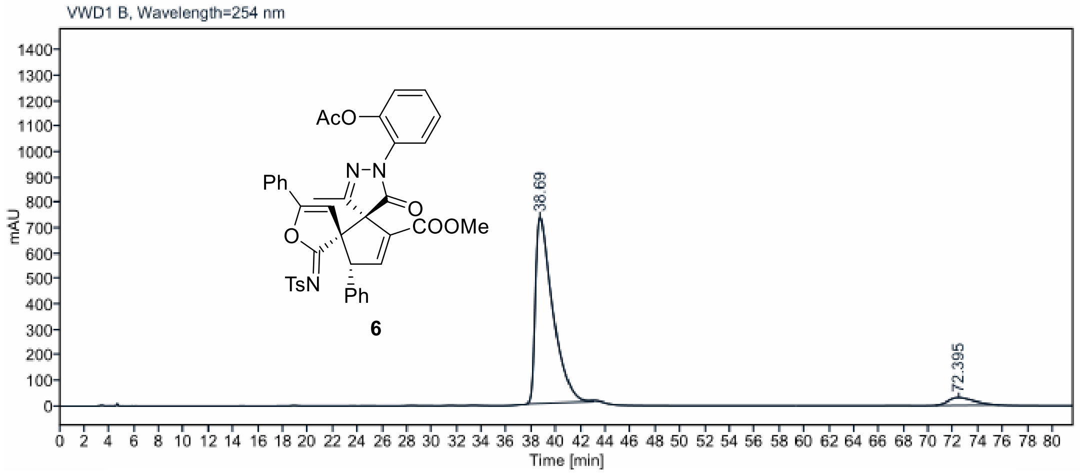

Signal: VWD1 B, Wavelength=254 nm

RT [min]	Type	Width [min]	Area	Height	Area%	Name
38.690	MM	1.6265	71548.7891	733.1597	94.2797	
72.395	MM	2.4281	4341.1387	29.7980	5.7203	
		Sum	75889.9277			

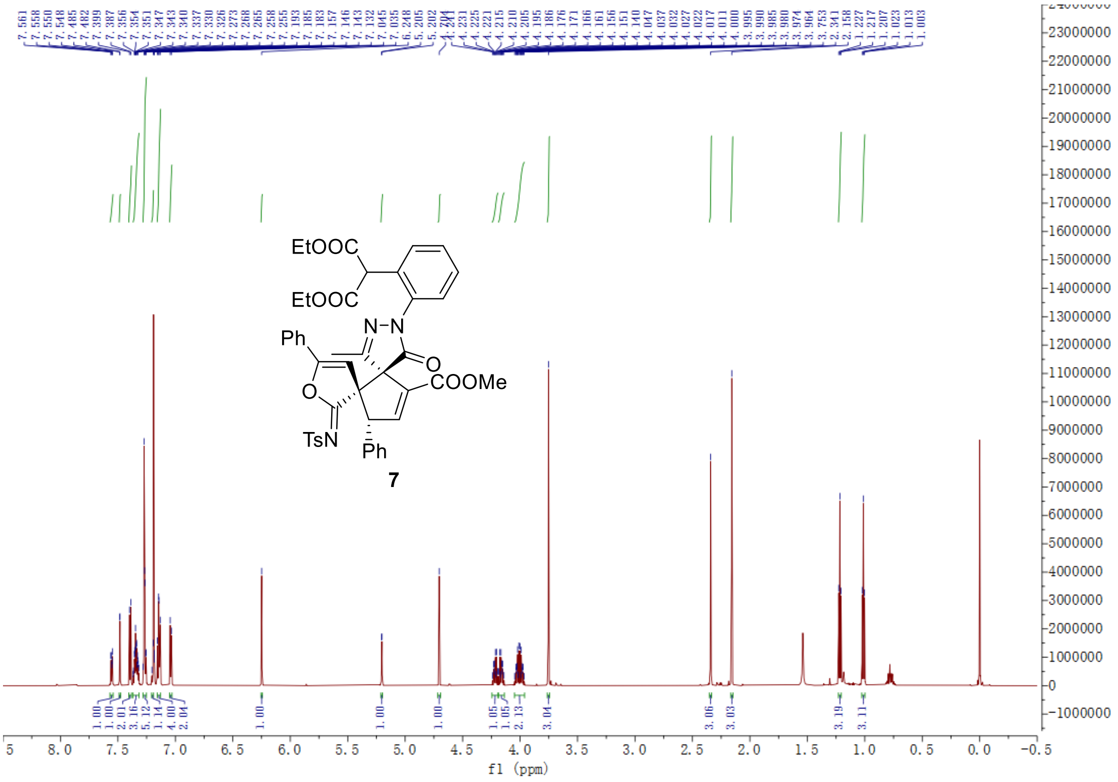
EtOOC
EtOOC
Ph
N–N
O
COOMe
O
TsN
Ph
7
f1 (ppm)

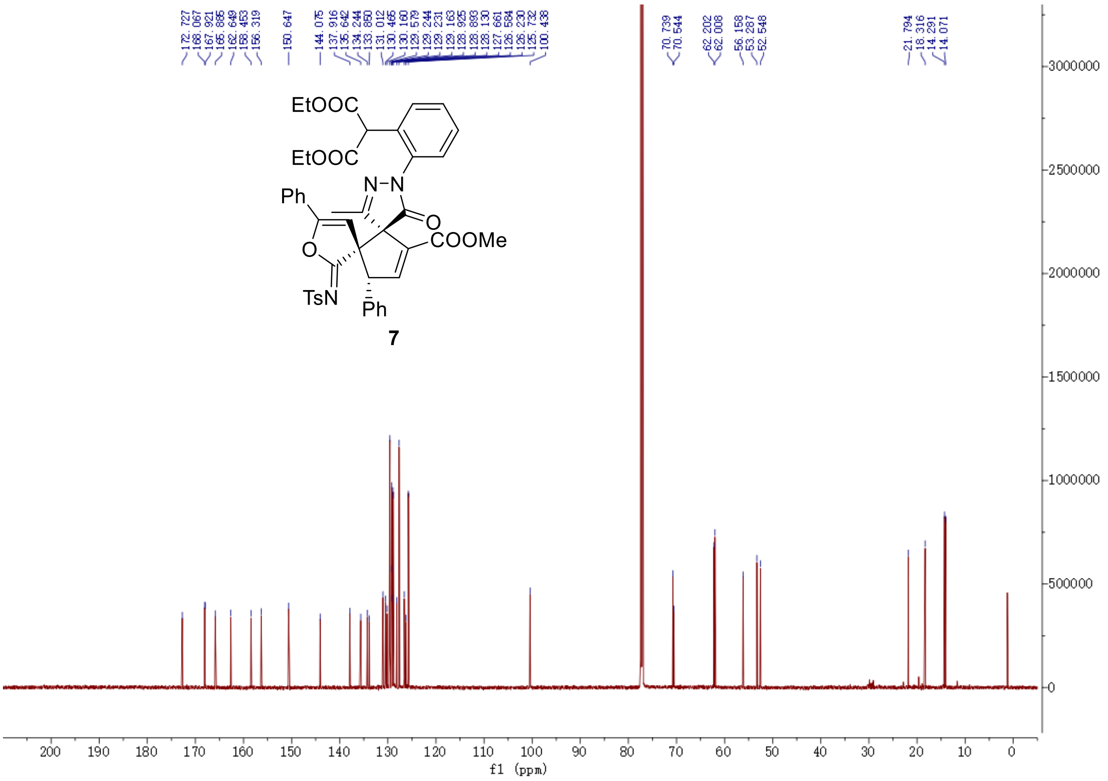
172.727
168.067
167.921
165.885
162.649
158.463
156.319
150.647
144.075
137.916
135.642
134.244
133.850
131.012
130.465
130.160
129.579
129.244
129.231
129.163
128.925
128.893
128.130
127.661
126.584
126.230
125.732
100.438
70.739
70.544
62.202
62.008
56.158
53.287
52.548
21.794
18.316
14.291
14.071
EtOOC
EtOOC
Ph
N–N
O
COOMe
O
TsN
Ph
7
f1 (ppm)

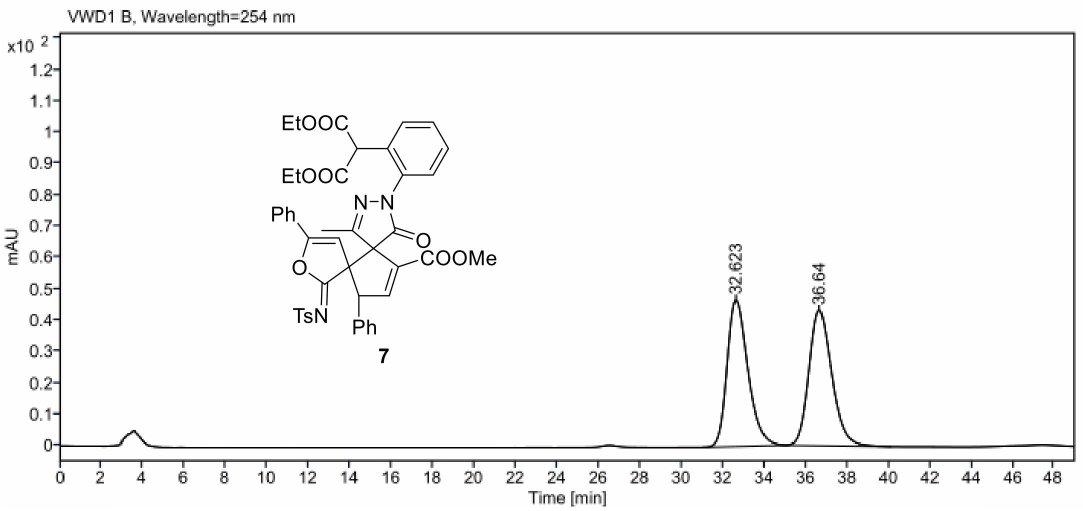

Signal: VWD1 B, Wavelength=254 nm

RT [min]	Type	Width [min]	Area	Height	Area%	Name
32.623	BB	1.0684	3260.0547	46.7032	49.4706	
36.640	BB	1.1850	3329.8350	43.2113	50.5294	
		Sum	6589.8896			

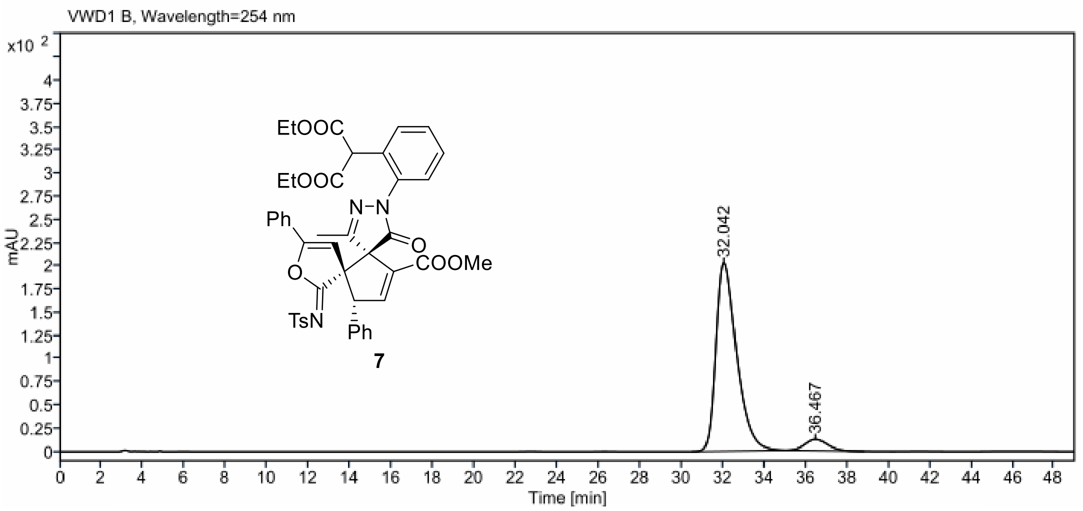

Signal: VWD1 B, Wavelength=254 nm

RT [min]	Type	Width [min]	Area	Height	Area%	Name
32.042	BB	1.0597	14185.1318	203.3809	93.7377	
36.467	BB	1.1898	947.6604	12.3143	6.2623	
		Sum	15132.7922			

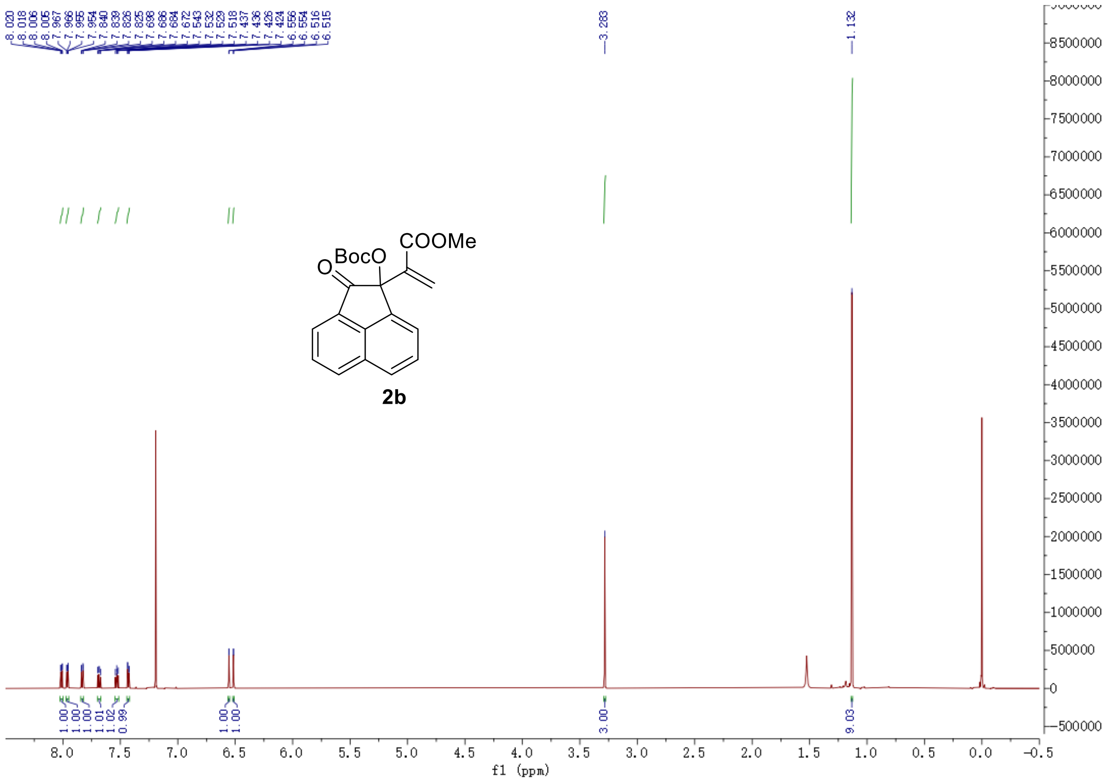
8.020
8.018
8.006
8.005
7.967
7.966
7.955
7.954
7.840
7.839
7.826
7.825
7.688
7.686
7.684
7.672
7.543
7.532
7.529
7.518
7.437
7.436
7.426
7.424
6.556
6.554
6.516
6.515
3.283
1.132
COOMe
BocO
O
2b
1.00
1.00
1.00
1.01
1.02
0.99
1.00
1.00
3.00
9.03
f1 (ppm)

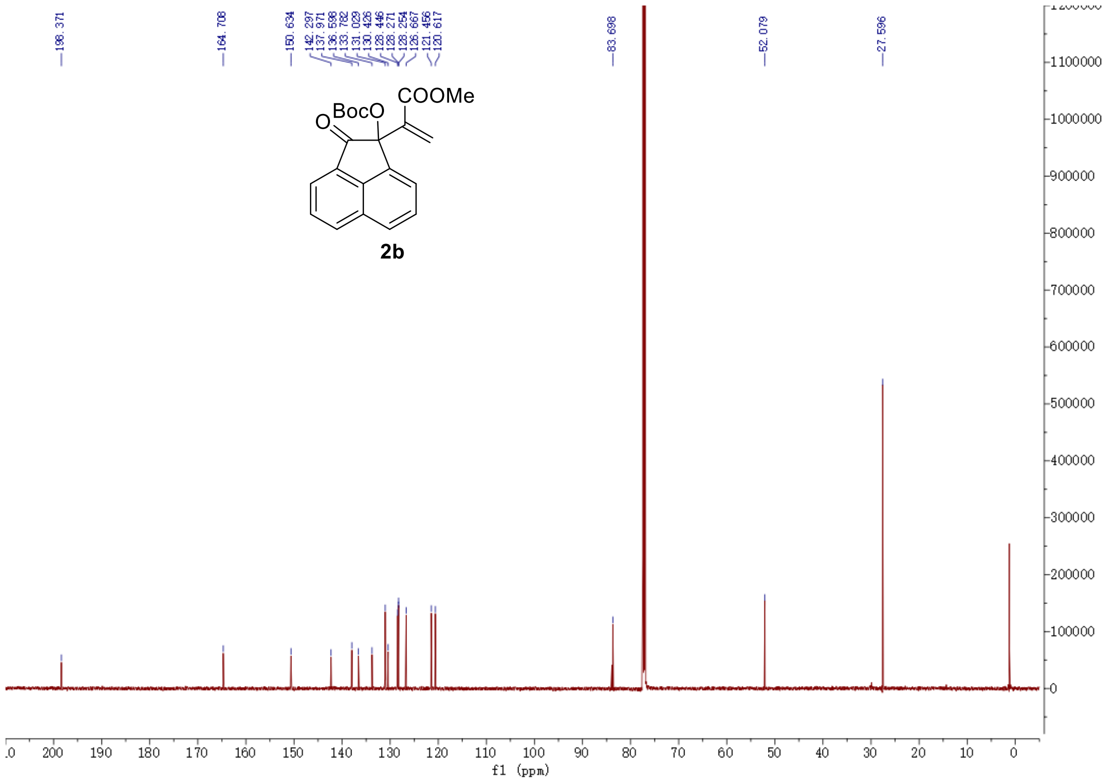
198.371
164.708
150.634
142.297
137.971
136.598
133.782
131.029
130.426
128.446
128.271
128.254
126.667
121.456
120.617
83.698
52.079
27.596
COOMe
BocO
O
2b
f1 (ppm)

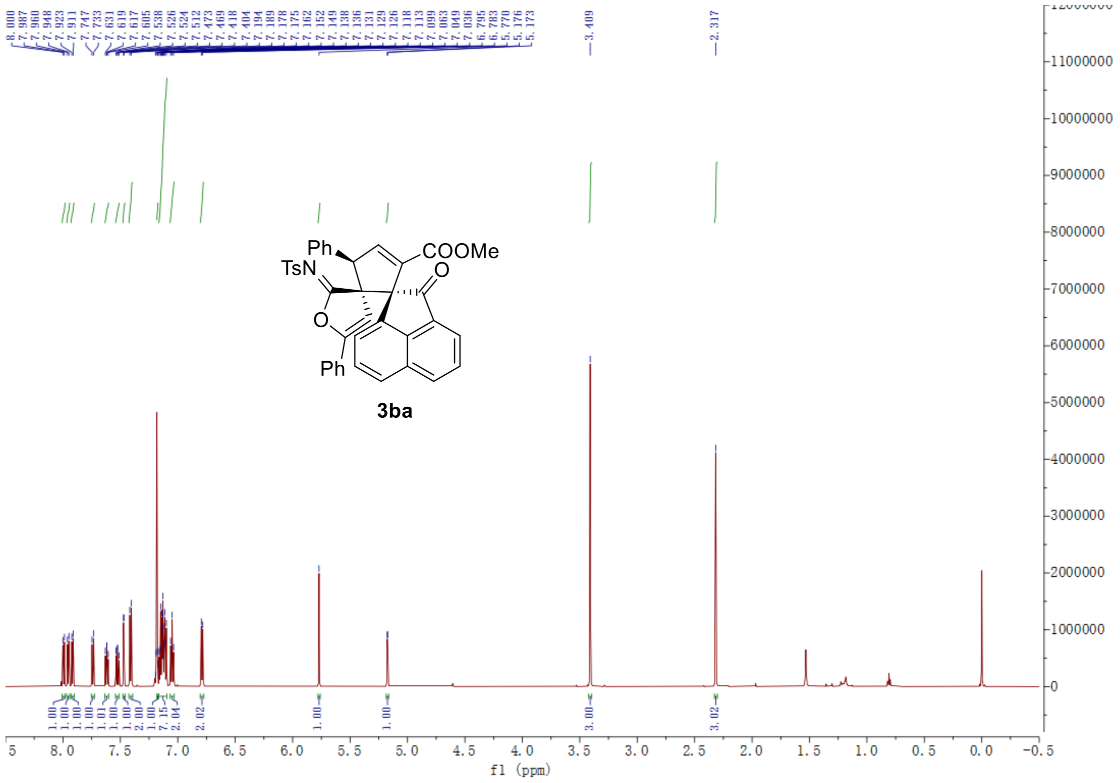
8.000 7.987 7.960 7.948 7.923 7.911 7.747 7.733 7.631 7.619 7.617 7.605 7.538 7.526 7.524 7.512 7.473 7.469 7.418 7.404 7.194 7.189 7.178 7.175 7.162 7.152 7.149 7.138 7.136 7.131 7.129 7.126 7.118 7.113 7.099 7.063 7.049 7.036 6.795 6.783 5.776 5.173
3.409
2.317
Ph
TsN
COOMe
O
O
Ph
3ba
1.00 1.00 1.00 1.00 1.01 1.00 1.00 2.00 1.00 7.15 2.04 2.02
1.00
1.00
3.00
3.02
fl (ppm)

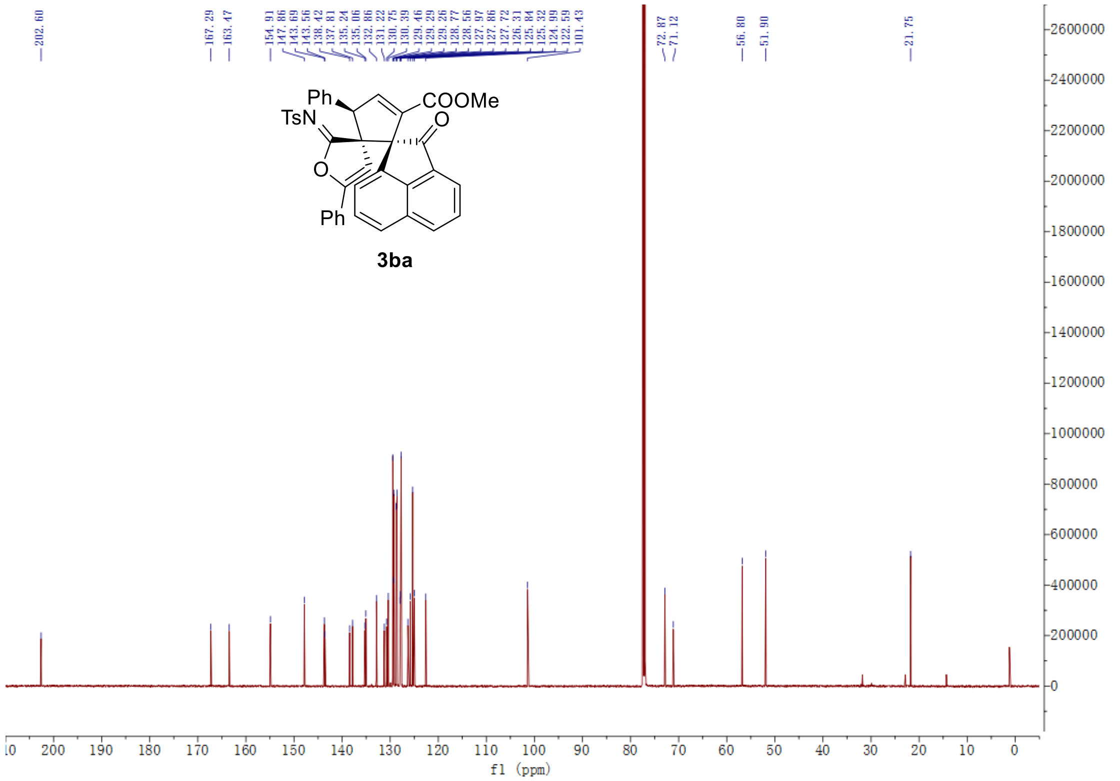
202.60
167.29
163.47
154.91 147.86 143.69 143.56 138.42 137.81 135.24 135.06 132.06 131.22 130.75 130.39 129.46 129.29 129.26 128.77 128.56 127.97 127.86 127.72 126.31 125.84 125.32 124.99 122.59 101.43
72.87 71.12
56.80
51.90
21.75
Ph
TsN
COOMe
O
O
Ph
3ba
fl (ppm)

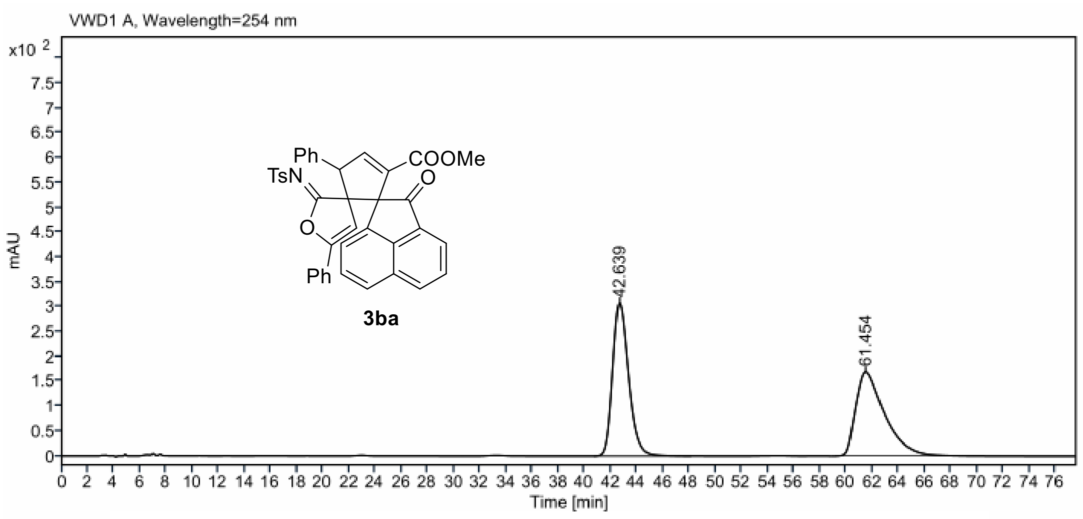

Signal: VWD1 A, Wavelength=254 nm

RT [min]	Type	Width [min]	Area	Height	Area%	Name
42.639	MM	1.4173	26196.7422	308.0658	50.0586	
61.454	MM	2.5715	26135.4570	169.3933	49.9414	
		Sum	52332.1992			

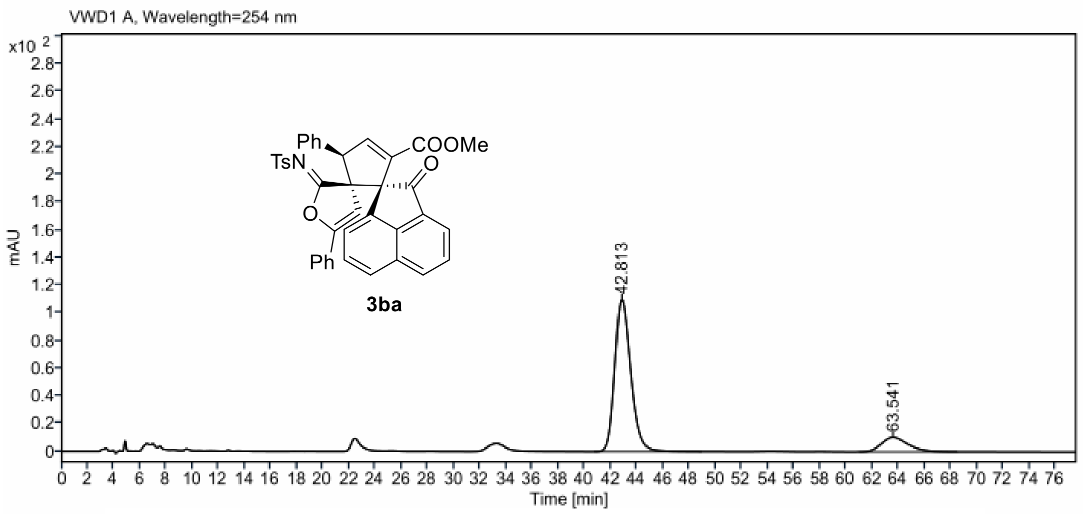

Signal: VWD1 A, Wavelength=254 nm

RT [min]	Type	Width [min]	Area	Height	Area%	Name
42.813	MM	1.4205	9358.8564	109.8056	86.3041	
63.541	MM	2.3670	1485.1934	10.4577	13.6959	
		Sum	10844.0498			
