# Supplementary material for: Silver/chiral pyrrolidinopyridine relay catalytic cycloisomerization/(2 + 3) cycloadditions of enynamides to asymmetrically synthesize bispirocyclopentenes as PDE1B inhibitors
Source: Commun Chem. 2023 Jun 19;6:128. doi: 10.1038/s42004-023-00921-6 (PMC10279699; doi:10.1038/s42004-023-00921-6)
Supplement: Supplementary file 7 — Supplementary Data 4 [file 42004_2023_921_MOESM7_ESM.pdf]

Supplementary Data 4

DFT calculations

1. Computational method

All density functional theory (DFT) calculations were performed using Gaussian 16 program software.[1] The SMD model[2] was employed to simulate the solvent effect of chloroform solution. Full geometry optimizations were operated to locate all of the stationary points, using M06 density functional theory method[3] with 6-31G(d,p) basis set[4] for all atoms under experimental temperature and pressure (313.15 K and 1 atm). In the meantime, the stability of the DFT wave-function of the auxiliary Kohn–Sham determinant was examined.[5] Harmonic vibrational frequency calculations were implemented at the same level of theory to characterize all stationary points. Herein, minima have zero imaginary frequencies, and transition states (TS) have one imaginary vibrational frequency. Intrinsic reaction coordinate (IRC) method[6] was implemented to track minimum energy paths connecting each transition state structure to two corresponding minima. To get more accurate energies, single-point energies of all stationary points were calculated with the same functional and at a larger basis set of def2-TZVP in the chloroform solution under experimental temperature and pressure (313.15 K and 1 atm). This theoretical level is denoted as M06/Def2-TZVP-SMD($CHCl_3$)//M06/6-31G(d,p)-SMD($CHCl_3$). Atomic charges were analyzed by natural population analysis (NPA) to investigate the electronic properties for key intermediate.[7]

2. Computational results

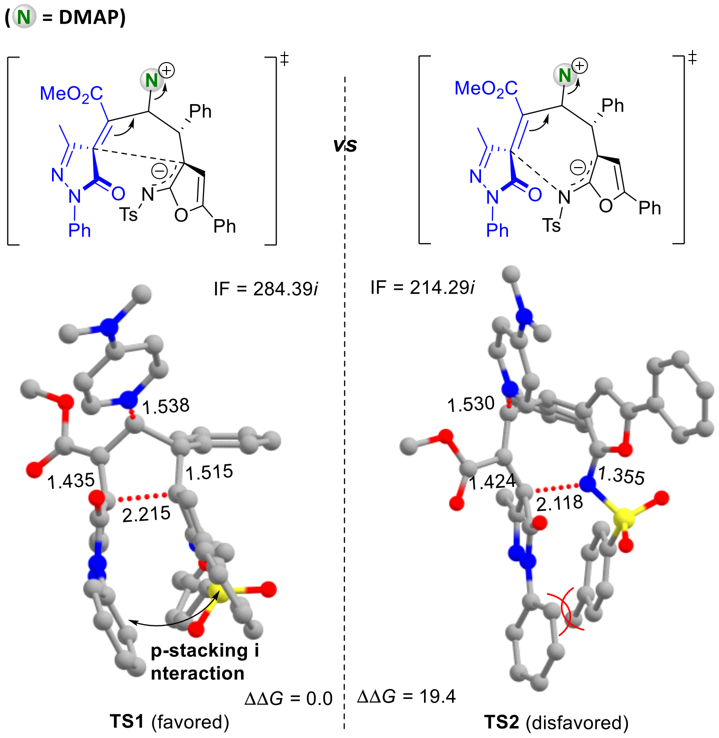

Figure S1. Optimized geometries of the regioselectivity-determining transition states. Energies and bond distances are given in kcal mol^{-1} and angstroms (Å), respectively. For clarity, hydrogen atoms on the geometries are not shown.

A computational investigation by density functional theory (DFT) was undertaken to better understand the observed regioselectivity of the reaction. The optimized geometries of the regioselectivity-determining transition states **TS1** and **TS2** are given in Figure 1. The relevant computational details and cartesian coordinates of optimized structures are provided in SI. Comparison of energies of **TS1** and **TS2** reveals that the (3 + 2) annulation is found to be more favored than the (4 + 3) annulation by 19.4 kcal mol^{-1}. It may origin from the fact that that the electron deficiency at the internal C2 position is more significant than that of terminal N4 position by using natural population analysis (NPA) (for detail, see Figure 2). Moreover, there is favorable π-stacking interaction between two aromatic moieties in the structure of **TS1**, while the steric hindrance is indeed observed between the Ph group of **2a** moiety and the Ts group in the structure of **TS2**.

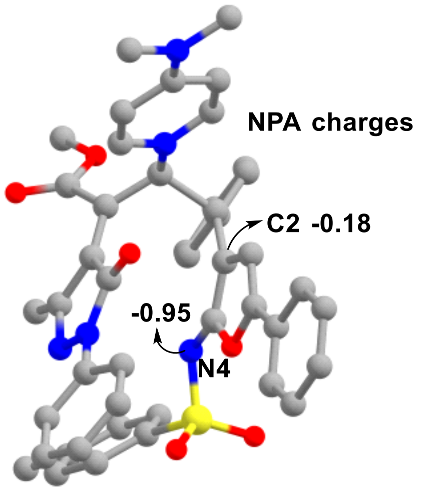

Figure S2. Natural population analysis (NPA) for the key intermediate **IM**.

3. Energies and cartesian coordinates of all stationary points

IM

Zero-point correction= 0.781884 (Hartree/Particle)
Thermal correction to Energy= 0.835767
Thermal correction to Enthalpy= 0.836758
Thermal correction to Gibbs Free Energy= 0.693919
Sum of electronic and zero-point Energies= -2860.279177
Sum of electronic and thermal Energies= -2860.225294
Sum of electronic and thermal Enthalpies= -2860.224303
Sum of electronic and thermal Free Energies= -2860.367142
SCF Done = -2861.93019047

Imaginary frequency: 0 (cm^{-1})

Standard orientation:

Center Number	Atomic Number	Atomic Type	Coordinates (Angstroms) X	Y	Z
1	16	0	-0.393571	-3.277240	2.571606
2	8	0	-0.827002	-4.637909	2.229761
3	8	0	-0.263530	-2.982588	4.008259
4	6	0	-3.236259	0.455134	1.037026
5	7	0	-1.191183	-2.079585	1.817557
6	6	0	1.208525	-3.032107	1.831852
7	6	0	-4.697620	-1.429070	1.849506
8	6	0	-3.401708	-0.931546	1.554112
9	6	0	-2.510130	-1.987502	1.873975
10	6	0	1.726200	-3.993135	0.972841
11	1	0	1.143206	-4.883711	0.751410
12	6	0	2.976436	-3.790094	0.399146
13	1	0	3.386953	-4.538658	-0.277655
14	6	0	3.716837	-2.637219	0.673330
15	6	0	5.073254	-2.440738	0.068936
16	1	0	5.842374	-2.978232	0.638840
17	1	0	5.358758	-1.383510	0.055360
18	1	0	5.114751	-2.819479	-0.958425
19	6	0	1.929179	-1.878711	2.127928
20	1	0	1.513940	-1.139676	2.811217
21	6	0	3.173447	-1.685573	1.542378
22	1	0	3.742369	-0.783940	1.767492
23	6	0	-4.578460	-2.708878	2.302010
24	6	0	-2.275173	-1.473627	-1.259347

25	6	0	-3.518758	0.630328	-0.527943
26	6	0	-2.004735	1.287942	1.344836
27	6	0	-2.212906	2.631859	1.672087
28	6	0	-0.690477	0.845420	1.185144
29	6	0	-1.150328	3.514775	1.823468
30	1	0	-3.233679	2.998109	1.796105
31	6	0	0.376656	1.726807	1.338415
32	1	0	-0.495702	-0.201214	0.968763
33	6	0	0.154630	3.063812	1.649284
34	1	0	-1.342631	4.554940	2.077874
35	1	0	1.392933	1.357636	1.206715
36	1	0	0.991376	3.749376	1.764073
37	6	0	-5.512907	-3.708402	2.760087
38	6	0	-5.066724	-4.986982	3.131985
39	6	0	-6.885395	-3.420943	2.838604
40	6	0	-5.972398	-5.942463	3.573935
41	1	0	-4.007003	-5.222627	3.060725
42	6	0	-7.781424	-4.381504	3.280498
43	1	0	-7.244351	-2.433902	2.551521
44	6	0	-7.330795	-5.648066	3.652103
45	1	0	-5.611096	-6.928158	3.859200
46	1	0	-8.841251	-4.142131	3.337676
47	1	0	-8.035650	-6.398886	4.001562
48	8	0	-3.250080	-3.047562	2.321251
49	1	0	-4.057805	1.010423	1.509056
50	6	0	-2.516599	-0.119144	-1.343150
51	6	0	-1.602334	0.704945	-2.175445
52	8	0	-1.079282	0.312465	-3.198557
53	8	0	-1.443886	1.953503	-1.710099
54	6	0	-0.494411	2.757316	-2.405623
55	1	0	-0.535289	3.742303	-1.938417
56	1	0	0.510480	2.333919	-2.297017
57	1	0	-0.740065	2.830677	-3.469201
58	1	0	-5.629490	-0.888272	1.720863
59	6	0	-1.000708	-2.157538	-1.434410
60	6	0	-3.224666	-2.574225	-0.915440
61	7	0	-2.420066	-3.709530	-0.870357
62	6	0	-2.808978	-5.057129	-0.718906
63	6	0	-4.055072	-5.396942	-0.186283
64	6	0	-1.924019	-6.063829	-1.116889
65	6	0	-4.408673	-6.737253	-0.076217
66	1	0	-4.735727	-4.619829	0.143892
67	6	0	-2.290679	-7.396175	-0.989051
68	1	0	-0.952454	-5.788985	-1.515887

69	6	0	-3.536772	-7.743829	-0.475772
70	1	0	-5.381454	-6.990374	0.340729
71	1	0	-1.591887	-8.170084	-1.299383
72	1	0	-3.821526	-8.789035	-0.382071
73	7	0	-1.106102	-3.420819	-1.140354
74	6	0	0.358219	-1.600184	-1.663414
75	1	0	1.097527	-2.357863	-1.384030
76	1	0	0.512719	-1.312653	-2.706938
77	1	0	0.531396	-0.702936	-1.052281
78	8	0	-4.447462	-2.573868	-0.841664
79	1	0	-3.373076	1.698817	-0.716733
80	6	0	-5.921064	1.092955	-0.237218
81	6	0	-5.316415	-0.337016	-1.971441
82	6	0	-7.240551	1.003970	-0.563842
83	1	0	-5.591747	1.744386	0.565578
84	6	0	-6.618065	-0.461061	-2.367182
85	1	0	-4.512335	-0.797663	-2.530202
86	6	0	-7.654439	0.185800	-1.651042
87	1	0	-7.949156	1.588915	0.010728
88	1	0	-6.826213	-1.070424	-3.238498
89	7	0	-4.954233	0.401514	-0.895579
90	7	0	-8.946465	0.061308	-1.994665
91	6	0	-9.319160	-0.745732	-3.146982
92	1	0	-9.000005	-1.787212	-3.021684
93	1	0	-10.403941	-0.732981	-3.254969
94	1	0	-8.878377	-0.351167	-4.070809
95	6	0	-9.980246	0.759612	-1.245793
96	1	0	-9.890744	1.848141	-1.352629
97	1	0	-10.957041	0.458408	-1.625053
98	1	0	-9.939601	0.503667	-0.180799

TS1

Zero-point correction= 0.777985 (Hartree/Particle)
Thermal correction to Energy= 0.832102
Thermal correction to Enthalpy= 0.833094
Thermal correction to Gibbs Free Energy= 0.688116
Sum of electronic and zero-point Energies= -2860.272827
Sum of electronic and thermal Energies= -2860.218709
Sum of electronic and thermal Enthalpies= -2860.217718
Sum of electronic and thermal Free Energies= -2860.362696

SCF Done = -2861.91852797

Imaginary frequency: 284.39*i* (cm^{-1})

Standard orientation:

Center Number	Atomic Number	Atomic Type	Coordinates (Angstroms) X	Y	Z
1	16	0	0.081354	-3.110566	2.459080
2	8	0	0.005138	-4.486934	1.968514
3	8	0	-0.138060	-2.872933	3.888459
4	6	0	-3.097388	0.044389	0.640520
5	7	0	-0.868558	-2.064164	1.587242
6	6	0	1.669463	-2.462242	2.013772
7	6	0	-4.389575	-2.084707	1.204459
8	6	0	-3.141147	-1.459542	0.816649
9	6	0	-2.137114	-2.274181	1.495962
10	6	0	2.607611	-3.293924	1.419871
11	1	0	2.353534	-4.329416	1.207094
12	6	0	3.858881	-2.776297	1.098514
13	1	0	4.602265	-3.419498	0.630768
14	6	0	4.175653	-1.443638	1.360951
15	6	0	5.515850	-0.880923	1.003935
16	1	0	5.995352	-0.412843	1.872173
17	1	0	5.426817	-0.103790	0.234391
18	1	0	6.189777	-1.653860	0.620881
19	6	0	1.959295	-1.128494	2.293846
20	1	0	1.205464	-0.488076	2.750477
21	6	0	3.207322	-0.628560	1.961863
22	1	0	3.442835	0.415433	2.168750
23	6	0	-4.114371	-3.225512	1.871545
24	6	0	-2.614979	-1.882206	-1.292506
25	6	0	-3.372259	0.442216	-0.837021
26	6	0	-1.870985	0.738714	1.207351
27	6	0	-1.844226	0.992656	2.581447
28	6	0	-0.786163	1.155787	0.434812
29	6	0	-0.773264	1.653523	3.170417
30	1	0	-2.680566	0.661329	3.198168
31	6	0	0.284404	1.826722	1.019031
32	1	0	-0.770555	0.955253	-0.634341
33	6	0	0.294990	2.082320	2.386227
34	1	0	-0.777302	1.841802	4.241668
35	1	0	1.118300	2.146339	0.397389
36	1	0	1.132362	2.607866	2.840820
37	6	0	-4.932719	-4.275153	2.438437

38	6	0	-4.390101	-5.200913	3.339508
39	6	0	-6.286225	-4.370979	2.085216
40	6	0	-5.199078	-6.180931	3.899289
41	1	0	-3.335033	-5.143608	3.596540
42	6	0	-7.087415	-5.350543	2.651290
43	1	0	-6.694790	-3.682648	1.346983
44	6	0	-6.547720	-6.255839	3.563407
45	1	0	-4.771986	-6.892479	4.602062
46	1	0	-8.136269	-5.417115	2.371558
47	1	0	-7.177413	-7.025305	4.004028
48	8	0	-2.741984	-3.364110	2.036620
49	1	0	-3.935091	0.410421	1.252104
50	6	0	-2.680552	-0.512754	-1.714614
51	6	0	-1.937842	-0.073983	-2.859171
52	8	0	-1.311578	-0.775312	-3.645587
53	8	0	-2.037015	1.280086	-3.060986
54	6	0	-1.383092	1.763145	-4.222166
55	1	0	-1.564022	2.840344	-4.248657
56	1	0	-0.304981	1.572103	-4.187412
57	1	0	-1.784657	1.300013	-5.130600
58	1	0	-5.376096	-1.716156	0.951146
59	6	0	-1.409081	-2.720611	-1.330935
60	6	0	-3.716475	-2.883831	-1.330251
61	7	0	-3.075409	-4.104040	-1.163541
62	6	0	-3.648007	-5.312314	-0.713941
63	6	0	-4.960700	-5.647482	-1.053177
64	6	0	-2.895842	-6.152591	0.110582
65	6	0	-5.513877	-6.821803	-0.555194
66	1	0	-5.536654	-4.985560	-1.690312
67	6	0	-3.458581	-7.331819	0.580888
68	1	0	-1.886028	-5.861192	0.387922
69	6	0	-4.769355	-7.671014	0.257039
70	1	0	-6.539231	-7.077104	-0.815356
71	1	0	-2.868721	-7.980710	1.224850
72	1	0	-5.207640	-8.590520	0.638625
73	7	0	-1.690282	-3.977071	-1.174538
74	6	0	0.002586	-2.269684	-1.387876
75	1	0	0.659500	-3.066079	-1.022593
76	1	0	0.282088	-2.009193	-2.413131
77	1	0	0.145811	-1.380312	-0.762161
78	8	0	-4.922810	-2.724687	-1.463556
79	1	0	-3.014194	1.462352	-1.012876
80	6	0	-5.604766	1.454372	-0.326335
81	6	0	-5.484441	-0.017173	-2.123345

82	6	0	-6.930870	1.688370	-0.546717
83	1	0	-5.065385	1.976466	0.458982
84	6	0	-6.809129	0.170377	-2.411306
85	1	0	-4.843859	-0.659766	-2.716923
86	6	0	-7.603008	1.026349	-1.610618
87	1	0	-7.440537	2.397860	0.094799
88	1	0	-7.222589	-0.347978	-3.268580
89	7	0	-4.885181	0.585284	-1.076165
90	7	0	-8.912446	1.220131	-1.852660
91	6	0	-9.552488	0.549555	-2.973475
92	1	0	-9.439750	-0.538669	-2.900355
93	1	0	-10.618148	0.780284	-2.965395
94	1	0	-9.136035	0.881067	-3.933444
95	6	0	-9.681812	2.149740	-1.041303
96	1	0	-9.301736	3.175144	-1.132196
97	1	0	-10.718607	2.141364	-1.378488
98	1	0	-9.669843	1.862021	0.016833

TS2

Zero-point correction= 0.780387 (Hartree/Particle)
Thermal correction to Energy= 0.833566
Thermal correction to Enthalpy= 0.834557
Thermal correction to Gibbs Free Energy= 0.694091
Sum of electronic and zero-point Energies= -2860.245886
Sum of electronic and thermal Energies= -2860.192708
Sum of electronic and thermal Enthalpies= -2860.191716
Sum of electronic and thermal Free Energies= -2860.332183
SCF Done = -2861.89361567

Imaginary frequency: 214.29*i* (cm^{-1})

Standard orientation:

Center Number	Atomic Number	Atomic Type	Coordinates (Angstroms) X	Y	Z
1	16	0	-2.320150	-6.584934	1.308438
2	8	0	-3.662103	-6.362689	0.766891
3	8	0	-2.143472	-6.453186	2.760278
4	6	0	1.425268	-3.868050	0.409245
5	7	0	-1.147091	-5.655326	0.580961
6	6	0	-1.826225	-8.227878	0.859840
7	6	0	-0.247793	-2.413362	1.762670

8	6	0	0.144590	-3.633476	1.149216
9	6	0	-0.990216	-4.415019	1.104096
10	6	0	-2.742249	-9.086192	0.269114
11	1	0	-3.741842	-8.732362	0.028415
12	6	0	-2.351650	-10.388902	-0.025583
13	1	0	-3.059956	-11.068099	-0.497727
14	6	0	-1.062461	-10.837595	0.262086
15	6	0	-0.635757	-12.234521	-0.066091
16	1	0	-0.372123	-12.792651	0.841055
17	1	0	0.253180	-12.237278	-0.708733
18	1	0	-1.427072	-12.787187	-0.582253
19	6	0	-0.542956	-8.658775	1.185165
20	1	0	0.155713	-7.969001	1.653026
21	6	0	-0.164719	-9.953641	0.874873
22	1	0	0.846794	-10.288394	1.106807
23	6	0	-1.581226	-2.507552	2.049181
24	6	0	-0.615402	-5.562390	-1.466684
25	6	0	1.165639	-3.603603	-1.102613
26	6	0	2.218403	-5.116148	0.735886
27	6	0	3.407504	-5.375086	0.046116
28	6	0	1.872687	-5.950819	1.799078
29	6	0	4.210735	-6.457406	0.383687
30	1	0	3.716391	-4.720970	-0.769451
31	6	0	2.680362	-7.032407	2.144655
32	1	0	0.961372	-5.750978	2.361204
33	6	0	3.846250	-7.296047	1.433727
34	1	0	5.127646	-6.643850	-0.171268
35	1	0	2.391726	-7.670088	2.978392
36	1	0	4.472797	-8.143307	1.702678
37	6	0	-2.541927	-1.585546	2.615567
38	6	0	-3.858456	-1.982656	2.887074
39	6	0	-2.169466	-0.256524	2.870804
40	6	0	-4.771994	-1.072664	3.402678
41	1	0	-4.157884	-3.008885	2.688388
42	6	0	-3.087625	0.645478	3.388348
43	1	0	-1.153424	0.068785	2.647968
44	6	0	-4.394648	0.242872	3.657308
45	1	0	-5.789991	-1.395981	3.608548
46	1	0	-2.783392	1.672264	3.581200
47	1	0	-5.113962	0.951460	4.060987
48	8	0	-2.040446	-3.726063	1.634919
49	1	0	2.093162	-3.040972	0.701911
50	6	0	0.099548	-4.382354	-1.819652
51	6	0	-0.176960	-4.018800	-3.201765

52	8	0	-0.888465	-4.635821	-3.984713
53	8	0	0.548497	-2.945769	-3.625572
54	6	0	0.411736	-2.637170	-5.006270
55	1	0	1.079399	-1.793808	-5.196054
56	1	0	0.697509	-3.486608	-5.635512
57	1	0	-0.618473	-2.357245	-5.249371
58	1	0	0.389660	-1.553871	1.944245
59	6	0	-0.157483	-6.948190	-1.598402
60	6	0	-2.066418	-5.632179	-1.908196
61	7	0	-2.266443	-6.976646	-2.179854
62	6	0	-3.436432	-7.612925	-2.637141
63	6	0	-4.685073	-7.022817	-2.418881
64	6	0	-3.347590	-8.853774	-3.272577
65	6	0	-5.832011	-7.679686	-2.847785
66	1	0	-4.741753	-6.068136	-1.907569
67	6	0	-4.506567	-9.498126	-3.686567
68	1	0	-2.372491	-9.307168	-3.422981
69	6	0	-5.754264	-8.916519	-3.480838
70	1	0	-6.800906	-7.216656	-2.673280
71	1	0	-4.428492	-10.465427	-4.178614
72	1	0	-6.658354	-9.425026	-3.807252
73	7	0	-1.088634	-7.704214	-2.088855
74	6	0	1.216061	-7.473483	-1.438214
75	1	0	1.248660	-8.509514	-1.789048
76	1	0	1.928005	-6.876416	-2.021758
77	1	0	1.538045	-7.443040	-0.391002
78	8	0	-2.880838	-4.733264	-1.901775
79	6	0	1.786021	-1.206723	-1.090680
80	6	0	-0.489793	-1.695065	-1.119350
81	6	0	1.543881	0.133437	-0.985510
82	1	0	2.798521	-1.601797	-1.136954
83	6	0	-0.811470	-0.369068	-1.027354
84	1	0	-1.250330	-2.469467	-1.197950
85	6	0	0.206412	0.611683	-0.928407
86	1	0	2.391110	0.808346	-0.955615
87	1	0	-1.861832	-0.100937	-1.018613
88	7	0	0.792537	-2.120358	-1.137507
89	7	0	-0.077411	1.920116	-0.793801
90	6	0	-1.462765	2.363580	-0.754510
91	1	0	-2.011450	1.878886	0.062222
92	1	0	-1.487920	3.440624	-0.586864
93	1	0	-1.981304	2.150547	-1.697863
94	6	0	0.994520	2.899958	-0.718314
95	1	0	1.590730	2.917917	-1.639556

96	1	0	0.562457	3.891112	-0.575631
97	1	0	1.661073	2.694940	0.128172
98	1	0	2.119012	-3.610679	-1.651837

----

4. Supplementary References

1. Friscourt, F., Fahrni, C. J. & Boons, G.-J. Fluorogenic Strain-Promoted Alkyne–Diazo Cycloadditions. *Chem. -Eur. J.* **21**, 13996-14001 (2015).
2. Frisch, M. J., Trucks, G. W., Schlegel, H. B., Scuseria, G. E., Robb, M. A., Cheeseman, J. R., Scalmani, G., Barone, V ., Petersson, G. A., Nakatsuji, H., X. Li, Caricato, M., Marenich, A. V ., Bloino, J., Janesko, B. G., Gomperts, R., Mennucci, B., Hratchian, H. P., Ortiz, J. V ., Izmaylov, A. F., Sonnenberg, J. L., Williams-Young, D., Ding, F., Lipparini, F., Egidi, F., Goings, J., Peng, B., Petrone, A., Henderson, T., Ranasinghe, D., Zakrzewski, V . G., Gao, J., Rega, N., Zheng, G., Liang, W., Hada, M., Ehara, M., Toyota, K., Fukuda, R., Hasegawa, J., Ishida, M., Nakajima, T., Honda, Y., Kitao, O., Nakai, H., Vreven, T., Throssell, K., Montgomery, J. A., Jr., Peralta, Ogliaro, J. E., F., Bearpark, M. J., Heyd, J. J., Brothers, E. N., Kudin, K. N., Staroverov, V . N., Keith, T. A., Kobayashi, R., Normand, J., Raghavachari, K., Rendell, A. P., Burant, J. C., Iyengar, S. S., Tomasi, J., Cossi, M., Millam, J. M., Klene, M., Adamo, C., Cammi, R., Ochterski, J. W., Martin, R. L., Morokuma, K., Farkas, O., Foresman, J. B., & Fox, D. J., Gaussian 16, Revision C.01, Gaussian, Inc., Wallingford CT (2019).
3. Marenich, A. V., Cramer, C. J. & Truhlar, D. G. Universal Solvation Model Based on Solute Electron Density and on a Continuum Model of the Solvent Defined by the Bulk Dielectric Constant and Atomic Surface Tensions. *J. Phys. Chem. B* **113**, 6378-6396 (2009).
4. Zhao, Y. & Truhlar, D. G. The M06 suite of density functionals for main group thermochemistry, thermochemical kinetics, noncovalent interactions, excited states, and transition elements: two new functionals and systematic testing of four M06-class functionals and 12 other functionals. *Theor. Chem. Acc.* **120**, 215-241 (2008).
5. Zhao, Y. & Truhlar, D. G. Density Functionals with Broad Applicability in Chemistry. *Acc. Chem. Res.* **41**, 157-167 (2008).
6. Krishnan, R., Binkley, J. S., Seeger, R. & Pople, J. A. Self‐consistent molecular orbital methods. XX. A basis set for correlated wave functions. *J. Chem. Phys.* **72**, 650-654 (1980).
7. KMcLean, A. D. & Chandler, G. S. Contracted Gaussian basis sets for molecular calculations. I. Second row atoms, Z=11–18. *J. Chem. Phys.* **72**, 5639-5648 (1980).
