# Supplementary material for: Silver/chiral pyrrolidinopyridine relay catalytic cycloisomerization/(2 + 3) cycloadditions of enynamides to asymmetrically synthesize bispirocyclopentenes as PDE1B inhibitors
Source: Commun Chem. 2023 Jun 19;6:128. doi: 10.1038/s42004-023-00921-6 (PMC10279699; doi:10.1038/s42004-023-00921-6)
Supplement: Supplementary file 8 — Supplementary Data 5 [file 42004_2023_921_MOESM8_ESM.pdf]

Supplementary Data 5

Experimental procedures of bioassays

Bioassay of phosphodiesterase PDE1 and other PDE subfamilies

The PDE1B protein was purified according to the protocols described in previous report. PDE activity was measured by a scintillation proximity assay using a fixed amount of enzyme and substrate concentrations. The phosphodiesterase (PDE) assays measure the conversion of H^3-cAMP for PDE 1A, 1B, 1C, 3A1, 4D2, 7A2, 8A2 and 10A1) or H^3-cGMP for PDE 2A, 5A1, 6C, 9A2 and 11A4, by the relevant PDE enzyme subtype. The scintillation proximity beads bind selectively to H^3-AMP or H^3-GMP, with the magnitude of radioactive counts being directly related to PDE enzymatic activity. In brief, 1 μL of test compound in dimethyl sulfoxide was added to each well. Enzyme solution was then added to each well in buffer (Trizma and $MgCl_2$) containing Brij 35 (0.01% (v/v)). For PDE1 subtype assays the buffer additionally included $CaCl_2$ (30 mM) and calmodulin (25 U ml^{-1}). Subsequently, 20 μL of H^3-cGMP (or 20 μL of H^3-cAMP) was added to each well to start the reaction and the plate was incubated for 30 minutes at 25 ^oC. Following an additional 8h incubation period the plates were read on a MicroBeta radioactive plate counter to determine radioactive counts per well.

Table S4. Inhibitory profiling of compound 3x across representative PDE subtypes.

PDE subtype	IC_{50} (nM)	Selectivity (fold)
PDE1A	31.37	2.02
PDE1B	15.54	-
PDE1C	21.19	1.36
PDE2A	>10,000	>643
PDE3A	>10,000	>643
PDE4D2	1,237	79.6
PDE5A1	976	62.8
PDE6C	>10,000	>643
PDE7A1	>10,000	>643
PDE8A2	>10,000	>643
PDE9A2	>10,000	>643
PDE10A1	>10,000	>643
PDE11A4	>10,000	>643

Computational procedures in molecular dynamics simulations

For each system, energy minimization and MD simulation were performed by using the Gromacs-2020.6 package. The AMBER99 and GAFF forcefield were utilized to build the topology of protein and ligand molecules, respectively. Prior to MD simulations, the entire system was subject to energy minimization in two stages to remove bad contacts between the complex and the solvent molecules. Firstly, the water molecules and counterions were minimized by freezing the solute using a harmonic constraint of a strength of 100 kcal $mol^{-1}Å^{-2}$. Secondly, the entire system was minimized without restriction. Each stage was consisted of a 5000-step steepest descent and a 5000-step conjugate gradient minimization. In MD simulations, Particle Mesh Ewald (PME) was employed to deal with the long-range electrostatic

interactions. The cutoff distances for the long-range electrostatic and van der Waals energy interaction were set to 10 Å. The SHAKE procedure was utilized, and the time step was set to 2 fs. The systems were gradually heated in the NVT ensemble from 0 to 300 K over 500ps and equilibrium in the NPT ensemble over 500ps. Then, 100-ns scale MD simulations were performed under the constant temperature of 300 K. During the sampling process, the coordinates were saved every 10ps and the conformations generated from the simulations were used for further binding free energy calculations and decomposition analysis.

Cell culture and western blotting

The HLF cells were purchased from Procell Life Science Technology and was cultured in F12K with 10% fetal bovine serum (FBS) and 1% penicillin/streptomycin (both from Gibco; Thermo Fisher Scientific, Inc.). All the cell lines were maintained at 37°C in a humidified incubator with 5% CO_2. The cells were harvested and lysed with RIPA (Beyotime Institute of Biotechnology). The protein concentration of each sample was measured using a Pierce™ Rapid Gold BCA Protein Assay kit (Thermo Fisher Scientific, Inc.) based on the manufacturer's guidelines. Total protein was separated using 12.5% SDS-PAGE, transferred to PVDF membranes, blocked with 5% skimmed milk at room temperature for 2 h, then incubated with the following primary antibodies on a shaker overnight at 4°C. Following which, the membranes were washed with TBS containing 0.1% Tween-20 three times and incubated with HRP-conjugated secondary antibodies (1:10,000 dilution; ProteinTech Group, Inc.) for 1 h at room temperature. The blotted proteins were observed using Immobilon ECL Ultra Western HRP Substrate (Merck KGaA), scanned with a Chemi-Doc System (Bio-Rad Laboratories, Inc.) and analyzed using ImageJ software (https://imagej.net).

Immunofluorescence (IF) assays

In total, about $1x10^5$ HLF cells administrated with TGFβ1 and/or compound **3x** were plated on coverslips, cultured overnight at 37°C, the coverslips were fixed with 4% pro-cooled paraformaldehyde for 20 min at room temperature, these cells were fixed in 3.7% formalin (Sigma-Aldrich), permeabilized in 0.25% Triton X-100 (Sigma-Aldrich), and blocked with 10% goat serum for 1 h. After overnight incubation with the primary antibody diluted with 10% goat serum, we added 250 μL of the fluorescent secondary antibody solution (1:100, diluted with 10% goat serum) and incubated at room temperature for 1 h in the dark, then briefly incubated with DAPI (Invitrogen; Thermo Fisher Scientific, Inc.) at room temperature for 5 min in the dark. Finally, the slides were sealed with neutral balsam and viewed using a confocal fluorescence microscope (Axiovert 200 M; Zeiss GmbH).

BLM-induced pulmonary damage rat model

All animal care and experimental protocols were in accordance with the "Guide for the Care and Use of Laboratory Animals" (National Institutes of Health Publication, revised 1996, No. 86-23, Bethesda, MD) and approved by the Institutional Ethical Committee for Animal Research of Chengdu University of Traditional Chinese Medicine (No. 2022-37). After a habituation period of 1 week, the animals were randomly assigned into four groups: control group, model group, **3x** (20 mg kg^{-1}) group and positive control group (PFD 150 mg kg^{-1}). The modeling method was implemented in the model and the **3x** group as follows: after the rats were anesthetized by an intraperitoneal injection of 4% pentobarbital sodium (10 mL kg^{-1}), the lower neck was incised aseptically, dissected bluntly, and well exposed; then, about 0.2 mL of bleomycin (5 mg kg^{-1}) was injected and the rats were immediately erected and rotated several times to make the liquid distribute evenly. After the wounds were sutured, states of the rats after recovery

were observed. In the meantime, the rats in the control group were injected with the same amount of normal saline into the trachea. After 28 days of administration, the respiratory level in each group was measured. Then, the rats were anesthetized by an intraperitoneal injection of 4% pentobarbital sodium, and left lower pulmonary lobes were harvested after the rats were euthanized; the tissues were immersed in 4% buffered paraformaldehyde at room temperature overnight and then embedded in paraffin wax. Pulmonary samples were stained by the H&E or Masson’s trichrome staining. An Olympus FV-3000 microscope was used to examine the stained pulmonary sections.

Underlying raw images of Western blot

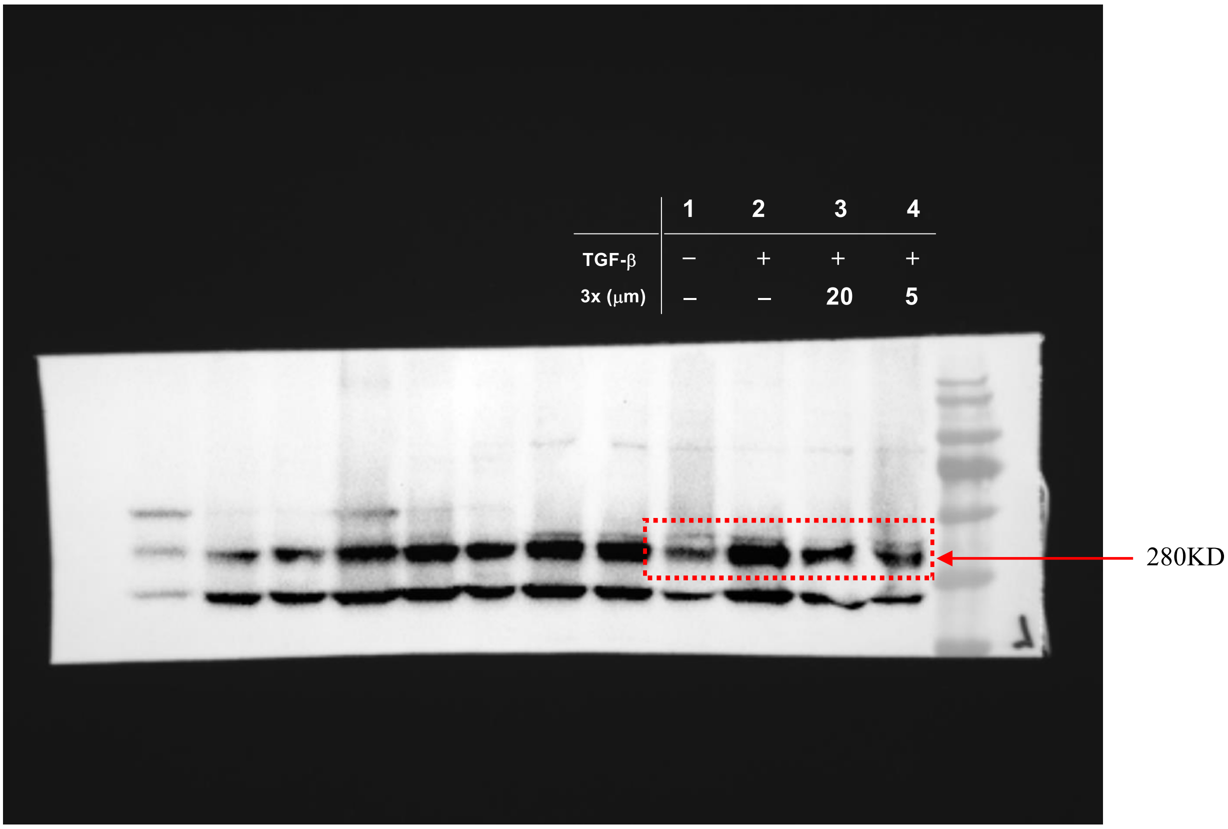

Figure S3. Western blot analysis of Fibronectin expression levels

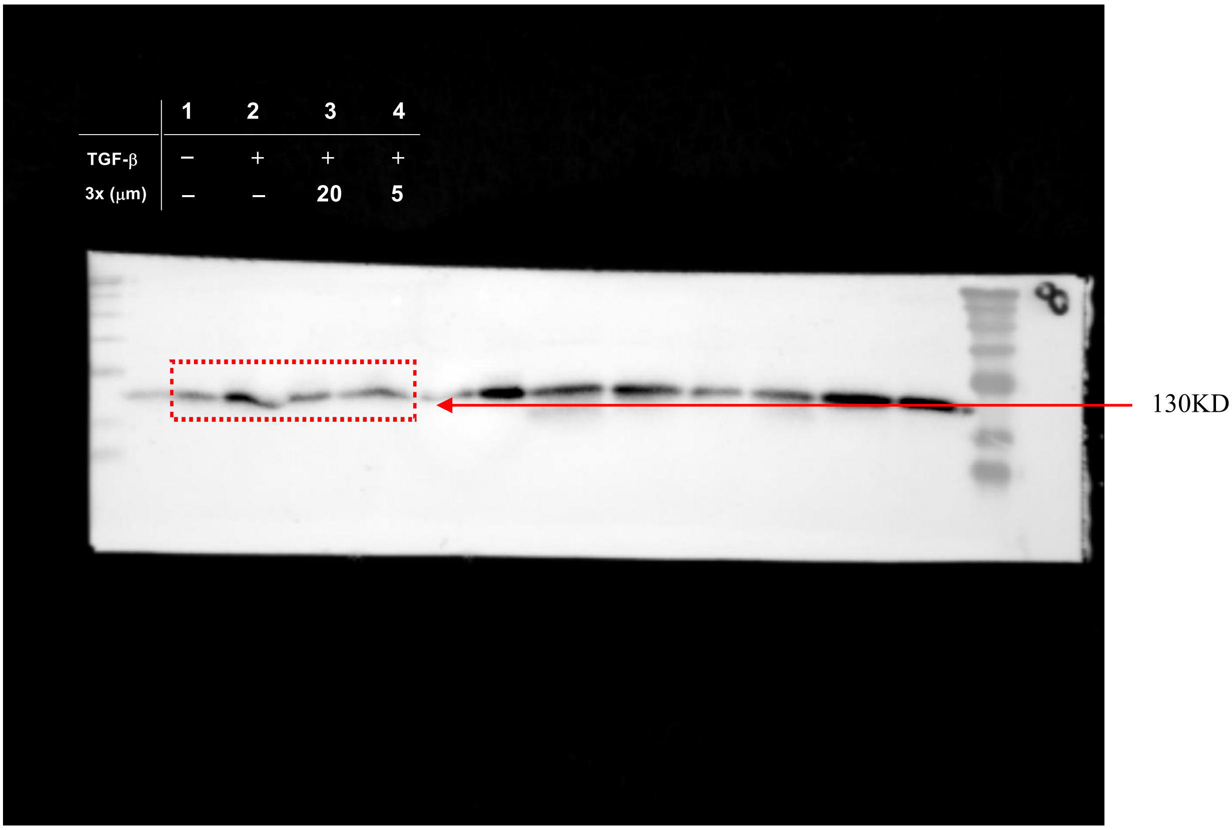

Figure S4. Western blot analysis of Collagen-I expression levels

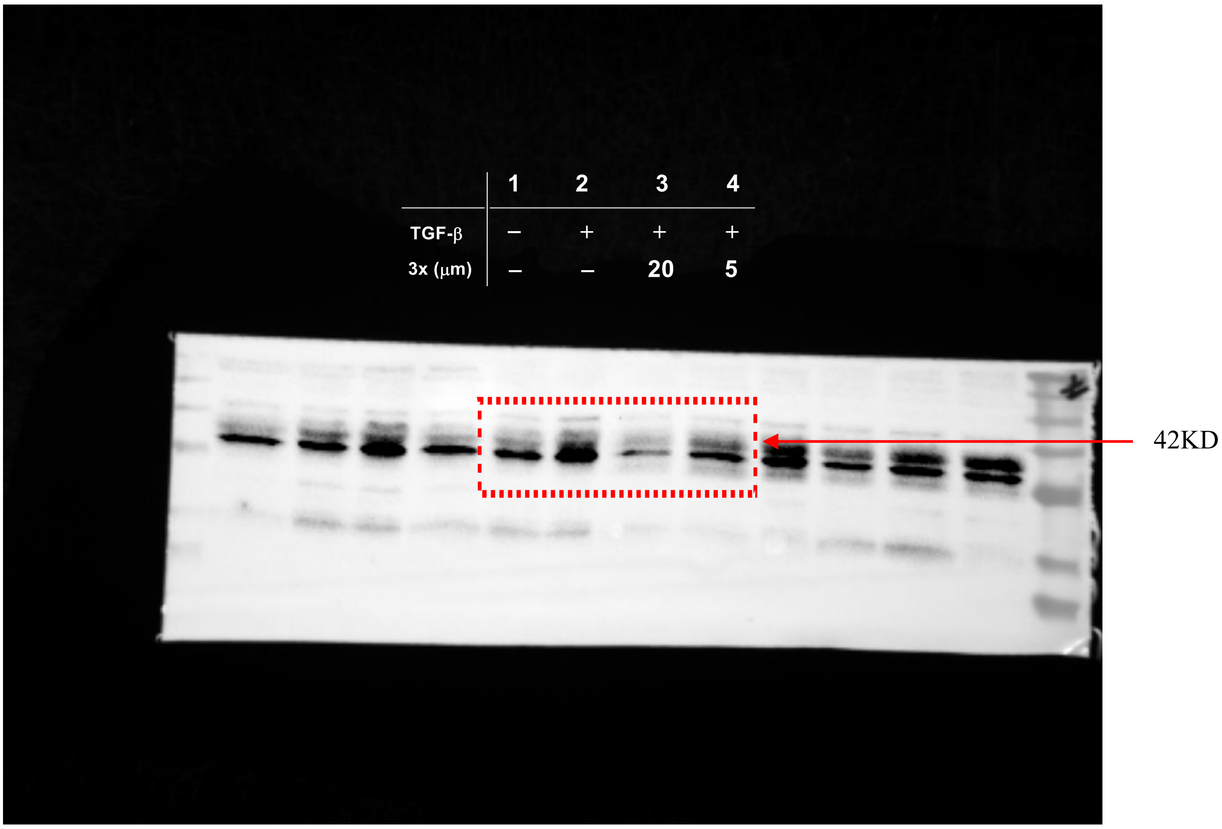

Figure S5. Western blot analysis of α-SMA expression levels

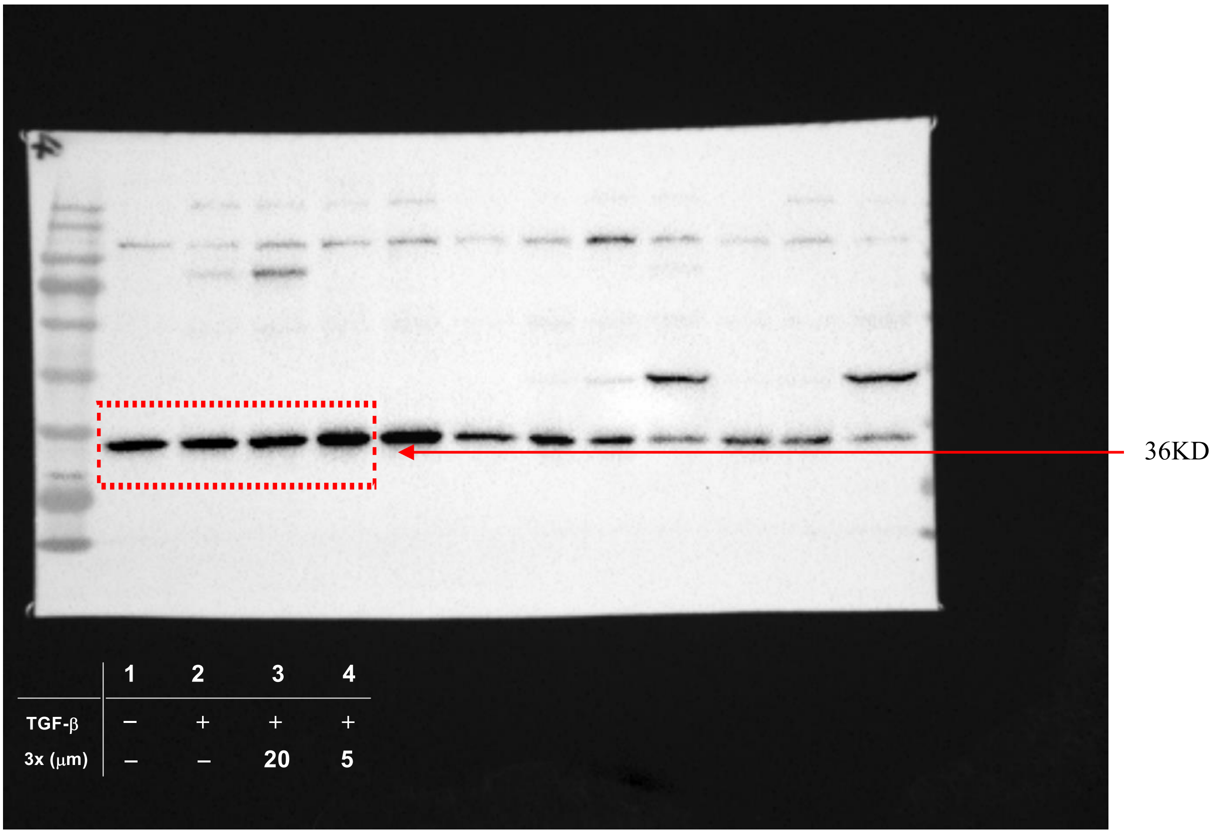

Figure S6. Western blot analysis of GAPDH expression levels
