# Supplementary material for: Silver/chiral pyrrolidinopyridine relay catalytic cycloisomerization/(2 + 3) cycloadditions of enynamides to asymmetrically synthesize bispirocyclopentenes as PDE1B inhibitors
Source: Commun Chem. 2023 Jun 19;6:128. doi: 10.1038/s42004-023-00921-6 (PMC10279699; doi:10.1038/s42004-023-00921-6)
Supplement: Supplementary file 9 — Reporting Summary [file 42004_2023_921_MOESM9_ESM.pdf]

nature portfolio

Corresponding author(s): Bo Han, Gu Zhan

Last updated by author(s): May 25, 2023

Reporting Summary

Nature Portfolio wishes to improve the reproducibility of the work that we publish. This form provides structure for consistency and transparency in reporting. For further information on Nature Portfolio policies, see our Editorial Policies and the Editorial Policy Checklist.

Statistics

For all statistical analyses, confirm that the following items are present in the figure legend, table legend, main text, or Methods section.

n/a	Confirmed	
☐	☒	The exact sample size (*n*) for each experimental group/condition, given as a discrete number and unit of measurement
☐	☒	A statement on whether measurements were taken from distinct samples or whether the same sample was measured repeatedly
☒	☐	The statistical test(s) used AND whether they are one- or two-sided<br>*Only common tests should be described solely by name; describe more complex techniques in the Methods section.*
☒	☐	A description of all covariates tested
☒	☐	A description of any assumptions or corrections, such as tests of normality and adjustment for multiple comparisons
☐	☒	A full description of the statistical parameters including central tendency (e.g. means) or other basic estimates (e.g. regression coefficient) AND variation (e.g. standard deviation) or associated estimates of uncertainty (e.g. confidence intervals)
☒	☐	For null hypothesis testing, the test statistic (e.g. *F*, *t*, *r*) with confidence intervals, effect sizes, degrees of freedom and *P* value noted<br>*Give P values as exact values whenever suitable.*
☒	☐	For Bayesian analysis, information on the choice of priors and Markov chain Monte Carlo settings
☒	☐	For hierarchical and complex designs, identification of the appropriate level for tests and full reporting of outcomes
☒	☐	Estimates of effect sizes (e.g. Cohen's *d*, Pearson's *r*), indicating how they were calculated

Our web collection on statistics for biologists contains articles on many of the points above.

Software and code

Policy information about availability of computer code

Data collection	No software was used.
Data analysis	No software was used.

For manuscripts utilizing custom algorithms or software that are central to the research but not yet described in published literature, software must be made available to editors and reviewers. We strongly encourage code deposition in a community repository (e.g. GitHub). See the Nature Portfolio guidelines for submitting code & software for further information.

Data

Policy information about availability of data

All manuscripts must include a data availability statement. This statement should provide the following information, where applicable:

- Accession codes, unique identifiers, or web links for publicly available datasets
- A description of any restrictions on data availability
- For clinical datasets or third party data, please ensure that the statement adheres to our policy

The authors confirm that the data supporting the findings of this study are available within the article or supplementary data.

Human research participants

Policy information about studies involving human research participants and Sex and Gender in Research.

Reporting on sex and gender	This information has not been collected.
Population characteristics	This information has not been collected.
Recruitment	This information has not been collected.
Ethics oversight	This information has not been collected.

Note that full information on the approval of the study protocol must also be provided in the manuscript.

Field-specific reporting

Please select the one below that is the best fit for your research. If you are not sure, read the appropriate sections before making your selection.

☒ Life sciences ☐ Behavioural & social sciences ☐ Ecological, evolutionary & environmental sciences

For a reference copy of the document with all sections, see nature.com/documents/nr-reporting-summary-flat.pdf

Life sciences study design

All studies must disclose on these points even when the disclosure is negative.

Sample size	There were three independent duplicated in the in vitro assays. And there were six mice in each group in the BLM stimulated pulmonary fibrosis models.
Data exclusions	No data were excluded from the analyses.
Replication	There were three independent duplicates in the in vitro assays.
Randomization	The animals were randomly allocated into each group after BLM stimulation.
Blinding	Blinding was not relevant to the study.

Reporting for specific materials, systems and methods

We require information from authors about some types of materials, experimental systems and methods used in many studies. Here, indicate whether each material, system or method listed is relevant to your study. If you are not sure if a list item applies to your research, read the appropriate section before selecting a response.

Materials & experimental systems

n/a	Involved in the study	
☐	☒	Antibodies
☐	☒	Eukaryotic cell lines
☒	☐	Palaeontology and archaeology
☐	☒	Animals and other organisms
☒	☐	Clinical data
☒	☐	Dual use research of concern

Methods

n/a	Involved in the study	
☒	☐	ChIP-seq
☒	☐	Flow cytometry
☒	☐	MRI-based neuroimaging

Antibodies

Antibodies used	Fibronectin (CST, #26836, E5H6X)<br>COL1A1 (CST, #72026, E8F4L) Rabbit mAb<br>α-Smooth Muscle Actin (CST, #19245, D4K9N)<br>GAPDH (Proteintech, 60004-1-Ig)
Validation	Fibronectin (Rabbit mAb, WB/IHC/IF/IP)<br>COL1A1 (Rabbit mAb, WB/IHC/IF)

α-Smooth Muscle Actin (Rabbit mAb, WB/IHC/IF/IP)
GAPDJ (Mouse mAb, WB/IF/IP/ELISA/FC)

Eukaryotic cell lines

Policy information about cell lines and Sex and Gender in Research

Cell line source(s)	Cells are preserved and provided by the State Key Laboratory of Biotherapy, Sichuan University
Authentication	We cultured the cells in DMEM supplemented with quantitative penicillin, streptomycin and 10% fetal bovine serum at 37 ? and 5% CO_2 atmosphere.
Mycoplasma contamination	Cell mycoplasma test negative.
Commonly misidentified lines (See ICLAC register)	Commonly misidentified cell lines were not used in the study.

Animals and other research organisms

Policy information about studies involving animals; ARRIVE guidelines recommended for reporting animal research, and Sex and Gender in Research

Laboratory animals	BALB/c mice (male and female, 6-8 weeks old) were provided by Beijing Vital River Laboratory Animal Technology Co., Ltd. (Beijing, China).
Wild animals	The study did not involve wild animals.
Reporting on sex	This information has not been collected.
Field-collected samples	In the experiment, all the animals are placed in sterilized mouse cages in an environment with a temperature of 22°C and a humidity of 50-60%.
Ethics oversight	All relevant animal care and experimental protocols were in accordance with the “Guide for the Care and Use of Laboratory Animals” (National Institutes of Health Publication, revised 1996, No. 86-23, Bethesda, MD) and approved by the Institutional Ethical Committee for Animal Research of Chengdu University of Traditional Chinese Medicine (No. 2022-37).

Note that full information on the approval of the study protocol must also be provided in the manuscript.
